# Epigenomic analysis detects aberrant super-enhancer DNA methylation in human cancer

**DOI:** 10.1186/s13059-016-0879-2

**Published:** 2016-01-26

**Authors:** Holger Heyn, Enrique Vidal, Humberto J. Ferreira, Miguel Vizoso, Sergi Sayols, Antonio Gomez, Sebastian Moran, Raquel Boque-Sastre, Sonia Guil, Anna Martinez-Cardus, Charles Y. Lin, Romina Royo, Jose V. Sanchez-Mut, Ramon Martinez, Marta Gut, David Torrents, Modesto Orozco, Ivo Gut, Richard A. Young, Manel Esteller

**Affiliations:** Cancer Epigenetics and Biology Program (PEBC), Bellvitge Biomedical Research Institute (IDIBELL), 08908 L’Hospitalet de Llobregat, Barcelona, Catalonia Spain; Whitehead Institute for Biomedical Research, 9 Cambridge Center, Cambridge, MA 02142 USA; Computational and Systems Biology Program, Massachusetts Institute of Technology, Cambridge, MA 02139 USA; Department of Medical Oncology, Dana-Farber Cancer Institute, 44 Binney Street, Boston, MA 02115 USA; Joint Biomedical Research Institute-Barcelona Supercomputing Center (IRB-BSC) Program in Computational Biology, Baldiri Reixac 10-12, 08028 Barcelona, Catalonia Spain; Department of Neurosurgery, University of Goettingen, Robert Koch. Str. 40, 37075 Goettingen, Germany; Centre Nacional d’Anàlisi Genòmica, Barcelona, Catalonia Spain; Institució Catalana de Recerca i Estudis Avançats (ICREA), 08010 Barcelona, Catalonia Spain; Institute for Research in Biomedicine (IRB Barcelona), Baldiri Reixac 10-12, 08028 Barcelona, Catalonia Spain; Department of Biochemistry and Molecular Biology, University of Barcelona, 08028 Barcelona, Catalonia Spain; Department of Biology, Massachusetts Institute of Technology, Cambridge, MA 02139 USA; Department of Physiological Sciences II, School of Medicine, University of Barcelona, 08036 Barcelona, Catalonia Spain

**Keywords:** Super-enhancer, DNA methylation, Epigenomics, Cancer

## Abstract

**Background:**

One of the hallmarks of cancer is the disruption of gene expression patterns. Many molecular lesions contribute to this phenotype, and the importance of aberrant DNA methylation profiles is increasingly recognized. Much of the research effort in this area has examined proximal promoter regions and epigenetic alterations at other loci are not well characterized.

**Results:**

Using whole genome bisulfite sequencing to examine uncharted regions of the epigenome, we identify a type of far-reaching DNA methylation alteration in cancer cells of the distal regulatory sequences described as super-enhancers. Human tumors undergo a shift in super-enhancer DNA methylation profiles that is associated with the transcriptional silencing or the overactivation of the corresponding target genes. Intriguingly, we observe locally active fractions of super-enhancers detectable through hypomethylated regions that suggest spatial variability within the large enhancer clusters. Functionally, the DNA methylomes obtained suggest that transcription factors contribute to this local activity of super-enhancers and that *trans*-acting factors modulate DNA methylation profiles with impact on transforming processes during carcinogenesis.

**Conclusions:**

We develop an extensive catalogue of human DNA methylomes at base resolution to better understand the regulatory functions of DNA methylation beyond those of proximal promoter gene regions. CpG methylation status in normal cells points to locally active regulatory sites at super-enhancers, which are targeted by specific aberrant DNA methylation events in cancer, with putative effects on the expression of downstream genes.

**Electronic supplementary material:**

The online version of this article (doi:10.1186/s13059-016-0879-2) contains supplementary material, which is available to authorized users.

## Background

The naked DNA sequence alone cannot explain the different cellular functions or phenotypes of cells and organisms with identical genetic sequences, such as the presence of different tissues within the same individual [1], monozygotic twins [2], and cloned animals [3]. This is even more pertinent when we try to explain the pathophysiology of the most common human diseases with their multifactorial causes. The existence of different chemical marks, such as DNA methylation and post-translational modifications of histones, that regulate gene activity in the epigenetic layers has taken center stage in biology and medicine [4]. However, many studies have taken a biased approach in examining the regulatory sequences nearest to the transcriptional start sites of the studied genes and, with rare exceptions [5–7], other potentially important regions have been neglected in attempts to address the role of epigenomics in tissue identity and disease. In this context, the existence of super-enhancers [8] or locus control regions [9, 10], large clusters of transcriptional enhancers that drive expression of genes that define cell identity, has been described. Most importantly, disease-associated variation is especially enriched in the super-enhancers of the corresponding cell types [11], and new super-enhancers for oncogenes and other transforming genes have been identified in cancer cells [12–15]. Herein, we present human DNA methylomes at single-nucleotide resolution of normal and cancer cells to identify epigenetic shifts in super-enhancers associated with these diseases.

## Results and discussion

We performed whole genome bisulfite sequencing (WGBS) to obtain unique DNA methylation data sets for five normal tissues and eight associated cancer samples (Table 1). Normal samples (n = 5) included brain, blood (CD19+), breast, lung and colon specimens. In order to enable the analysis of DNA methylation variance from different perspectives, we produced references data sets for cancer samples that involved both primary tumors (n = 2) and cancer cell lines (n = 6). These included a donor-matched primary colon triplet (normal tissue, primary cancer, liver metastasis) and matched primary and metastasis breast cancer cell lines, enabling us to analyze changes during tumor progression. The epigenetic peculiarities that could be present in cancer cell lines were addressed through replication experiments in an additional set of 78 normal tissue samples and 714 primary tumors using the HumanMethylation450 BeadChip (Table 2). The obtained data were also validated using the DNA methylation microarray profiles available for 208 normal samples and 675 primary tumor samples in The Cancer Genome Atlas (TCGA) projects (Table 2) [16–18].

**Table 1:** Whole genome bisulfite sequencing of 13 human samples

Sample ID	Status	Tissue	Origin	Total reads	Coverage genome	Coverage CpG	Average methylation	SE^a^	SE covered^b^
CD19	Normal	B cells	Primary	318714023	6.0	14.1	76.0	688	99.0 %
Brain	Normal	Brain (white matter)	Primary	557237398	11.1	7.0	77.1	1067	99.6 %
Breast	Normal	Breast	Primary	606872747	15.1	32.1	73.0	1099	99.5 %
Colon	Normal	Colon	Primary	609043678	13.7	24.3	69.6	1023	99.4 %
Lung	Normal	Lung	Primary	333333332	7.2	8.7	74.4	1286	99.1 %
Colon_P	Cancer	Colorectal cancer	Primary	670281443	16.7	24.6	66.5	1023	99.4 %
Colon_M	Cancer	Colorectal cancer metastasis	Primary	652566967	16.3	24.7	62.4	1023	99.4 %
MDA-MB-468PT	Cancer	Breast cancer	Cell line	626288553	15.4	37.6	57.1	1099	99.4 %
MDA-MB-468LN	Cancer	Breast cancer metastasis	Cell line	600134926	14.3	37.1	42.8	1099	99.5 %
U87MG	Cancer	Glioblastoma	Cell line	281524883	6.3	8.5	55.7	1067	99.6 %
H1437	Cancer	Lung adenocarcinoma	Cell line	333333332	7.9	10.3	48.1	1286	99.1 %
H1672	Cancer	Small cell lung cancer	Cell line	329691560	7.4	10.5	65.6	1286	99.1 %
H157	Cancer	Lung squamous cell cancer	Cell line	333333332	7.8	10.7	41.8	1286	99.2 %

^a^ *SE* is the number of super-enhancer regions determined in the respective normal tissue samples [11]

^b^SE covered is the percentage of super-enhancers covered by WGBS (>50 % of CpG sites)

**Table 2:** Genome-scale DNA methylation analysis of 78 normal tissue samples, 714 primary tumors and 24 metastasis samples (HumanMethylation450 BeadChip) and combined expression/DNA methylation analysis of 208 normal and 675 primary tumor samples (TCGA)

Cancer type	Status	Origin	Number of samples	Number of samples TCGA
Lung	Normal	Primary sample	26	57
Colon	Normal	Primary sample	18	41
Breast	Normal	Primary sample	19	110
Brain (white matter)	Normal	Primary sample	10	-
Blood (CD19+)	Normal	Primary sample	5	-
Lung adenocarcinoma	Cancer	Primary sample	321	216
Lung squamous cell carcinoma	Cancer	Primary sample	120	-
Colorectal cancer	Cancer	Primary sample	103	258
Colorectal cancer metastasis	Metastasis	Primary sample	24	-
Breast cancer	Cancer	Primary sample	66	201
Small cell lung cancer	Cancer	Primary sample	56	-
Glioblastoma	Cancer	Primary sample	48	-

**Fig. 1:**
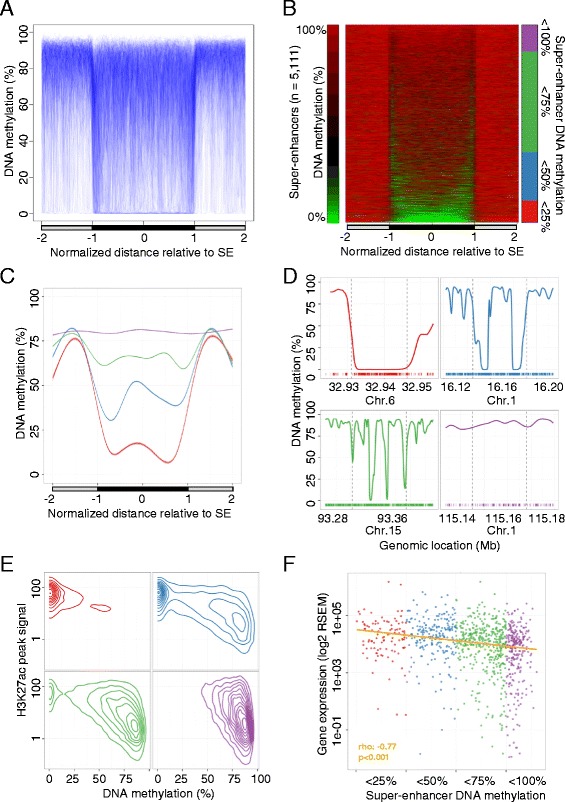
DNA methylation profile of super-enhancer regions derived from normal tissues determined by whole genome bisulfite sequencing (WGBS). **a** Scaled DNA methylation profile of 5111 super-enhancers (*SE*) in their respective normal tissues (n = 5). Each super-enhancer is represented by a single line (*blue*) and smoothed DNA methylation levels inside the super-enhancer (*black bar*) and equally sized flanking sequences (*gray bar*) are displayed. **b** DNA methylation levels of super-enhancers in their respective normal tissues (n = 5) in equally sized windows (*green*, 0 %; *red*, 100 %). Each horizontal line represents a single super-enhancer, ordered by average DNA methylation levels. Super-enhancers are grouped according to their average DNA methylation levels (*red*, <25 %; *blue*, <50 %; *green*, <75 %; *purple*, <100 %). **c** Smoothed average DNA methylation profile of all super-enhancers categorized into four groups on the basis of DNA methylation levels. **d** Examples of the DNA methylation profiles of breast super-enhancers representing the defined subgroups. Genomic locations of the super-enhancers (*dashed vertical lines*) and equally sized flanking regions are displayed and CpG dinucleotides locations are indicated (*bottom*, *colored bars*). **e** Association between DNA methylation levels and H3K27ac peak signals [11] in normal breast tissues and breast super-enhancers (n = 1091) displayed as averaged values (50-bp windows). Super-enhancers were classified into previously defined subgroups. **f** Gene expression levels of target transcripts in normal breast tissues. Scaled averaged expression levels of genes associated with breast super-enhancers (n = 1091) in normal breast tissue samples (n = 110; TCGA [16]). Super-enhancers were grouped according to their average DNA methylation levels. Significance of a Spearman’s correlation test is indicated. *RSEM* RNA-Sequencing by Expectation Maximization

Aligning uniquely mapping bisulfite sequencing reads (mean ~480 million reads per sample) of the original 13 samples undergoing whole genome single-nucleotide resolution analysis resulted in a median genomic coverage of 11.1× (14.1× CpG coverage) per sample. Consistent with previous reported results, apart from bimodal DNA methylation levels at promoter sites, the genomes presented high methylation levels, which were globally reduced in cancer samples (Table S1 and Figure S1 in Additional file 1) [5, 6]. To estimate the relationship between super-enhancers and DNA methylation levels, we determined DNA methylation profiles for enhancer regions within their respective tissue types. From the super-enhancers previously described in our normal tissue types through the histone modification H3K27ac (identified as a superior and sufficient mark for the identification of super-enhancers [11]), we could examine 99.3 % (5128 of 5163; >50 % CpGs covered; Table S1 in Additional file 1) using our WGBS data. We found significant enrichment of unmethylated DNA sequences within the super-enhancers compared with the flanking genomic regions (Fisher’s exact test, odds ratio (OR) 5.6, *p* < 0.001), supporting the relevance of the features in the here interrogated context. In particular, the edges of the enhancers were CpG-unmethylated, clearly marking the boundaries of the regulatory regions (Fig. 1a, b), a phenomenon that was consistent throughout the analyzed tissue types (Figure S2 in Additional file 1) and that could not be observed in traditional enhancers (Figure S3a, b in Additional file 1) [11]. Moreover, super-enhancers were significantly more hypomethylated than traditional enhancers (Fisher’s exact test, OR 1.8, *p* < 0.001), further supporting DNA methylation to specifically indicate functionality in this enhancer subtype.

The fact that super-enhancer edges show lower DNA methylation levels compared with their center could be related to an enrichment of transcription factor binding sites at the extreme parts of the regions (Fisher’s exact test, OR 5.33, *p* = 1.0 × 10^−11^; Figure S3c in Additional file 1) [19]. Indeed, DNA hypomethylation and transcription factor occupancy revealed a significant relationship (Fisher’s exact test, OR 11.3, *p* = 2.2 × 10^−16^; Figure S3d in Additional file 1), consistent with previous reports describing a co-dependency of both regulatory mechanisms [20, 21].

The extent of tissue-specific DNA methylation differences in the super-enhancer regions was low, with only 12.6 % (644 out of 5111) of them showing CpG methylation differences from different normal tissues (δ hypomethylated regions (HMRs) occupancy >10 %; Supplementary methods, Figure S4a and Table S2 in Additional file 1). We assessed variance in super-enhancer DNA methylation profiles by differential analysis of HMRs, focal sites of low DNA methylation levels that mark active regulatory loci [22–24], to account for the high heterogeneity at the large genomic regions represented by super-enhancers. Remarkably, tissue-specific HMRs at breast and blood super-enhancers were significantly enriched in specific transcription factor binding within the respective tissues, as measured by the occupancy of ten commonly profiled factors determined in CD19+ (GM12878; Fisher’s exact test, OR = 2.81, *p* < 0.001) and breast cells (MCF7; Fisher’s exact test, OR = 1.64, *p* = 0.007) [19]. Moreover, super-enhancers with tissue-specific DNA methylation levels in breast and brain samples were enriched at promoter regions compared with non-specific super-enhancers, in contrast to previous results that suggest tissue-specific DNA methylation to be enriched in *cis*-elements (Fisher’s exact test, OR 6.64, *p* < 0.001 and OR 1.74, *p* = 0.018, respectively; Figure S4b in Additional file 1) [1]. The sample with the greatest DNA methylation difference compared with normal tissues was that of the CD19+ cell-related super-enhancers (ANOVA, *p* < 0.001; Figure S4c in Additional file 1), which was the only representative of a non-solid tissue type. It is of note that the presence of tissue-specific DNA methylation in this minor fraction of super-enhancers could be validated by genome-scale analysis using DNA methylation microarrays (HumanMethylation450 BeadChip). Of the normal tissue-derived super-enhancers, 75.5 % (486 of 644) were represented by at least three probes, in a unique set of 78 normal samples (Table 2), representing the analyzed tissue types, of which 71.4 % (347 of 486) showed significant difference between the respective tissue types (Student’s *t*-test, false discovery rate (FDR) < 0.05; Figure S4d and Table S3 in in Additional file 1). As examples of super-enhancer tissue-specific DNA methylation we can cite the genes encoding the RNA-binding protein QKI (involved in myelinization and oligodendrocyte differentiation), which is unmethylated in white brain matter but heavily methylated in all other normal tissues (Figure S5a in Additional file 1), and lymphoblastic leukemia-associated hematopoiesis regulator 1 (LYL1; plays a role in blood vessel maturation and hematopoiesis), which is unmethylated in CD19+ cells but hypermethylated in all other normal tissues (Figure S5b in Additional file 1).

From the 5111 super-enhancers studied we established four categories based on their average DNA methylation levels (Fig. 1b, c). Remarkably, we determined striking differences between DNA methylation profiles at super-enhancers, ranging from fully hypermethylated to completely unmethylated (Fig. 1d). Moreover, focal hypomethylated regions pointed to spatial differences in DNA methylation within super-enhancers, suggesting local variability in their activity. Accordingly and in contrast to previous assumptions, the focal variability of the here studied epigenetic mark supports the action of independent regulatory units and challenges the conjoint activity of enhancer clusters for this subset of super-enhancer regions.

From an epigenetic perspective, the CpG unmethylated status was significantly correlated with H3K27ac occupancy (Spearman’s correlation test, rho 0.535, *p* < 0.001; Fig. 1e) and, to a lesser extent, with H3K4me1 (Spearman’s correlation test, rho 0.278, *p* < 0.001), further supporting the former mark as sufficiently bookmarking super-enhancer functionality. This association was independent of the local CpG density, suggesting a sequence-independent connection between the two epigenetic marks (multivariate linear model, *p* < 0.001; Figure S6 in Additional file 1). Most importantly, unmethylated status was significantly associated with increased transcriptional activity of the regulated target genes, indicating that DNA methylation levels at these sequences may be of value as surrogate marks of super-enhancer functionality (Spearman’s correlation test, rho −0.77, *p* < 0.001; Fig. 1f). Although, functional DNA methylation variance at enhancer sites has been reported previously [25–28], we observed a stronger effect of differential DNA methylation on gene expression levels of super-enhancer-related targets (Figure S7a in Additional file 1). It is of note that the increased correlation between DNA methylation and gene expression at super-enhancers compared with traditional enhancers was observed for enhancer sites overlapping promoter regions and those distal to the target gene transcription start site (TSS), suggesting an elevated effect of differential super-enhancer DNA methylation independent of the distance to its target (Figure S7a in Additional file 1). Moreover, DNA methylation levels at super-enhancers overlapping promoters showed significantly higher correlation at regions flanking the proximal (±2 kb of the TSS) promoter (Spearman’s correlation test, rho 0.26 versus 0.18), further suggesting that enhancer-specific dynamics drive gene regulation. It is noteworthy that we did not observe a correlation between super-enhancers and target promoter-related CpG island DNA methylation levels (Spearman’s correlation test, rho 0.0001, *p* = 0.99), although both genomic features independently correlated significantly with gene expression (Spearman’s correlation test, rho 0.31, *p* < 0.001 and rho 0.16, *p* < 0.001, respectively), suggesting an independent function of both regulatory elements. Furthermore, the effect of enhancers on gene expression was closely related to the enhancer size, with DNA methylation levels at super-enhancers presenting the highest correlation with target gene expression compared with smaller sized counterparts (Figure S7b in Additional file 1).

For *cis*-acting super-enhancers, we observed that the assignment of the closest gene as target resulted in better correlations between super-enhancer DNA methylation and gene expression than a chromatin conformation-based method (ChIA-PET Pol2 in MCF-7 cells, Spearman’s correlation test, rho −0.048, *p* = 0.4; Figure S7c in Additional file 1) [29]. However, both strategies clearly include falsely assigned enhancer–target pairs and more suitable methodologies have yet to be defined.

### Aberrant DNA methylation profiles of super-enhancers in human cancer

**Fig. 2:**
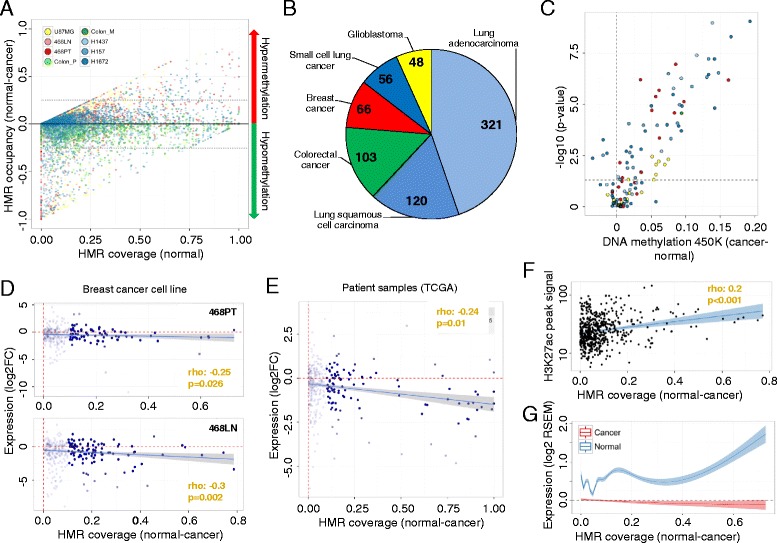
Cancer-specific alterations in DNA methylation within super-enhancer regions determined using WGBS. **a** Difference in DNA methylation levels (occupancy of hypomethylated regions (*HMRs*)) between cancer (n = 8) and normal (n = 5) samples paired within their respective tissue contexts (y-axis). HMR occupancy of normal tissues is indicated (x-axis) and cancer sample types are color-coded and the threshold indicated (*dotted line*; δ HMR occupancy 25 %). **b** Sample distribution of 714 cancer samples analyzed on the HumanMethylation450 BeadChip. **c** Validation of DNA hypermethylation at super-enhancers in 714 cancer samples using the HumanMethylation450 BeadChip (450 K). Significance was assessed by differential DNA methylation levels and the Student’s *t*-test (*p* value), comparing normal and cancer samples and averaging over the analyzed CpG (≥3) within a super-enhancer region (FDR < 0.05). The cancer samples are color-coded as defined in (**b**). **d** The association between HMR occupancy (WGBS) and target gene expression (RNA-seq) is assessed comparing normal breast (MCF10A) and the primary (468PT, *upper panel*) and metastatic (468LN, *lower panel*) breast cancer cell lines. Expression data are displayed as log transformed fold-change (*log2FC*) and significances of a Spearman’s correlation test are indicated. **e** Differences in HMR occupancy (WGBS) and target gene expression (RNA-seq, scaled log expression) are displayed comparing matched normal breast and primary carcinoma samples (TCGA [16], n = 25). **f** Association of H3K27ac signal (ChIP-seq) and differential HMR occupancy (WGBS) at hypermethylated super-enhancers. H3K27ac signals were retrieved from normal breast tissue [11]. **g** Smoothed (GAM) scaled log expression values of super-enhancer-related genes in matched normal and cancer samples (TCGA [16], n = 25) plotted against the difference in HMR occupancy (WGBS) for all super-enhancers gaining methylation in cancer. *GAM* Generalized Additive Model, *RSEM* RNA-Sequencing by Expectation Maximization

Considering the association between DNA methylation status and super-enhancer activity in normal tissues, we wondered whether the observed epigenetic pattern was significantly altered in human cancer. We observed that 14 % (727 out of 5111) of the super-enhancers studied underwent CpG methylation changes in their respective human tumor types, e.g., normal breast versus breast cancer cell lines (Fig. 2a). The most common DNA methylation shift was the loss of CpG methylation in the cancer sample, which was noted in 75.4 % (548 of 727) of cases, whilst 24.6 % (179 of 727) of super-enhancers gained DNA methylation across the eight tissue-matched cancer samples (δ HMR occupancy >25 %; Fig. 2a; Figure S8a and Tables S4 and S5 in Additional file 1). Interestingly, the hypomethylation events were rather unspecific, as they were associated with the global loss of DNA methylation usually observed in cancer samples (paired *t*-test, *p* > 0.05) [5, 6, 30], the only notable exception being colorectal tumors, in which they were significantly super-enhancer locus-specific (average flanking regions versus super-enhancer reduction 29.8 % [tumor] and 33.9 % [metastasis], paired *t*-test, *p* < 0.001; Figure S8b and Table S5 in Additional file 1). Thus, to determine functional epigenetic alterations, we decided to initially focus on the hypermethylated events, which were enriched in genes associated with transcriptional and metabolic processes and angiogenesis (FDR < 0.01; Table S6 in Additional file 1). Importantly, hypermethylation events were also replicated using DNA methylation microarray analyses in a unique cohort of 714 primary cancer samples (Table 2 and Fig. 2b), where 58.1 % (68 of 117) of the interrogated DNA hypermethylation events at super-enhancers were confirmed (Student’s *t*-test, FDR < 0.05; Fig. 2c; Table S7 in Additional file 1). These results further suggest that the hypermethylation events observed in the cancer cell line models are mirroring altered DNA methylation profiles at super-enhancer regions in primary tumors. Hypermethylated super-enhancers in cancer included genes previously related to cellular transformation (e.g., *CIC*, *FOXA2*, *FOXP1*, *RUNX1* and *TBX3*) [31]. Importantly, we excluded that copy number variations (CNVs) have confounded our analysis of the primary cancer samples by detecting significant differences in DNA methylation levels between normal and CNV samples in only a very minor fraction of the super-enhancers (4.3 %, 5/117; Student’s *t*-test, FDR < 0.05; Table S7 in Additional file 1).

It is of note that, using oxidative bisulfite (ox-BS) treatment coupled with DNA methylation microarray analyses, we could exclude the gain of DNA methylation observed in cancer to be due to an increase of 5-hydroxy methylation (5-hmC), a specific cytosine modification that confounds with 5-methylation (5-mC) in bisulfite (BS)-based analyses and found to be enriched in traditional enhancer regions (Figure S9 in Additional file 1) [32]. In order to test a significant contribution of the 5-hmC to the methylation gain in super-enhancers, we compared the methylation values obtained from BS-treated against ox-BS-treated cancer samples, enabling us to estimate the 5-hmC levels [33]. With the alternative hypothesis being that the ox-BS values were greater than 0, we did not observe a significant presence of 5-hmC in any cancer sample (paired one-tailed Wilcoxon test).

To further elucidate the functional consequences associated with the identified cancer-specific super-enhancer DNA methylation shifts, we investigated the impact of the tumor-associated gains of super-enhancer DNA methylation on gene expression. We first used a breast cancer model that included the paired breast cancer cell lines MDA-MB-468PT (derived from the primary tumor) and MDA-MB-468LN (derived from a lymph node metastasis) and the untransformed immortalized breast epithelial cell line MCF10A, associating differential gene expression (RNA sequencing, RNA-seq) with super-enhancer DNA methylation levels. As has been observed for the proximal regulatory gene regions, where a general repressive effect of DNA methylation is widely recognized [34], we found an association between DNA methylation gain in breast super-enhancer regions and gene repression of the associated genes for both MDA-MB-468PT (Spearman’s correlation test, rho −0.25, *p* = 0.026) and MDA-MB-468LN (Spearman’s correlation test, rho −0.3, *p* = 0.002; Fig. 2d) cell lines.

We extended these observations to primary breast tumors from the TCGA [16], whose expression patterns have also been determined by RNA-seq. We confirmed the significant association between the DNA methylation gains of super-enhancers identified in our breast cancer cell line data set and gene repression observed in the matched TCGA breast cancer samples (Spearman’s correlation test, rho −0.24, *p* = 0.01; Fig. 2e). Interestingly, the super-enhancers that became hypermethylated in breast cancer were those that, in normal breast epithelial cells, were the most enriched in the H3K27ac histone mark (Spearman’s correlation test, rho 0.2, *p* < 0.001; Fig. 2f), which defines these particular distal regulatory regions [8, 11, 13], and the H3K4me1 enhancer mark (Spearman’s correlation test, rho 0.2, *p* < 0.001). Remarkably, the most hypermethylated super-enhancers had also the highest level of expression for the respective associated genes in normal breast epithelial cells (linear slope 1.23, *p* < 0.001; Fig. 2g).

We were able to validate the link between cancer-specific super-enhancer hypermethylation and the transcriptional inactivation of the corresponding genes beyond the breast tumor type. In the lung tumorigenesis samples from the H1437 (lung adenocarcinoma) and H157 (lung squamous cell carcinoma) cancer cell lines, we found evidence that lung super-enhancer gain of DNA methylation was associated with the downregulation of the target genes (linear slope −3.06, *p* < 0.001 and −2.09, *p* = 0.004, respectively; Figure S10a, b in Additional file 1) determined by publically available expression microarrays [35]. We also extended these findings to primary lung adenocarcinoma and lung squamous cell carcinoma tumors from the TCGA [18], in which expression of the candidate genes originates from RNA-seq experiments. In this setting, we observed a significant association between lung super-enhancer hypermethylation identified in our lung cancer cell lines and gene downregulation found in the matched primary lung cancer samples (Spearman’s correlation test, rho −0.19, *p* = 0.012 and rho −0.25, *p* < 0.001, respectively; Figure S10c, d in Additional file 1). The significant association between cancer-specific DNA methylation of super-enhancers and gene repression was also noted in the glioblastoma cell line U87MG (Spearman correlation test, rho −0.26, *p* < 0.001; Figure S10e in Additional file 1), in which we performed an expression microarray experiment. Thus, the results overall suggest that a tumor-related gain of DNA methylation in super-enhancers has a transcriptionally repressive effect on the corresponding related genes.

**Fig. 3:**
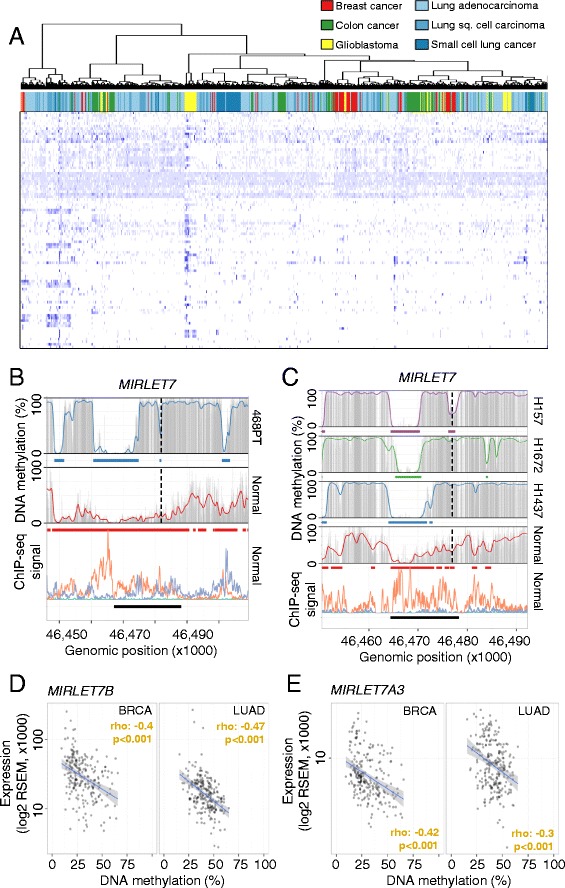
Cancer type-specific alterations of DNA methylation signatures at super-enhancer loci. **a** Hierarchical clustering of common hypomethylated super-enhancer regions in normal tissues (rows, <25 % average DNA methylation) in 714 cancer samples (columns). Average CpG methylation levels in common regions were clustered using Canberra distances and the Ward cluster method. DNA methylation levels are color-coded from 0 % (*light blue*) to 100 % (*dark blue*) and the different cancer types are color-coded. **b**, **c** DNA methylation profiles of the super-enhancer regions associated with *MIRLET7* in normal tissues and cell lines derived from breast (**b**) and lung cancer (**c**). Smoothed (*colored line*), raw (*gray bars*) CpG methylation levels, hypomethylated regions (*colored bars*) and super-enhancers (*black bars*) are indicated. The enhancer-related histone marks (*bottom panel*) H3K27ac (*orange*) and H3K4me1 (*purple*) are displayed as ChIP-seq signal intensities [11]. Transcription start sites are indicated (*broken line*). **d**, **e** Association of DNA methylation levels (TCGA, HumanMethylation450 BeadChip, averaged probe levels within the super-enhancer) and gene expression (TCGA, RNA-seq, absolute expression values) related to the *MIRLET7* super-enhancer and targeted microRNAs *MIRLET7B* (**d**) and *MIRLET7A3* (**e**) in breast (n = 201) and lung (n = 216) cancer samples. Significances of a Spearman’s correlation test are indicated. *RSEM* RNA-Sequencing by Expectation Maximization

We next considered the commonality among different tumor types within super-enhancer DNA methylation changes, and the type of genes and pathways affected by these aberrant epigenetic shifts. We first observed that within regions of commonly hypomethylated super-enhancers in normal contexts, the cancer samples (Table 2) clustered by tumor type (Fig. 3a), a phenomenon we previously identified for DNA methylation events in proximal promoters among distinct human tumors [36]. Interestingly, despite the clear presence of super-enhancer DNA methylation that is associated with the cancer type, there are hypermethylated super-enhancers shared by common epithelial tumors such as the breast and lung samples (Figure S11a in Additional file 1). This is the case for the super-enhancer of the tumor suppressor microRNA *MIRLET7*, where hypomethylation of the super-enhancer was diminished by a gain of CpG methylation in a fraction of the regulatory region (Fig. 3b, c; Figure S11b, c in Additional file 1). It is of note that the large highly hypomethylated super-enhancer regions displayed focal gains in DNA methylation in cancer, suggesting that distinct segments might exhibit specific functions in healthy and cancer contexts. Consistent with the suspected regulatory function, hypermethylation of the *MIRLET7*-associated super-enhancer region was associated with transcriptional silencing of *MIRLET7B* and *MIRLET7A3*, two family members coded within the affected pri-microRNA (Figure S11d in Additional file 1). Moreover, microRNAs *MIRLET7B* and *MIRLET7A3* were repressed in primary breast carcinomas (TCGA [16]; Wilcoxon test, *p* = 0.001 and *p* = 0.033, respectively) and lung adenocarcinomas (TCGA [18]; Wilcoxon test, *p* < 0.001 and *p* < 0.001, respectively) (Figure S11e, f in Additional file 1) and hypermethylation at super-enhancers was significantly correlated with microRNA repression in breast carcinomas (Spearman correlation test, rho −0.4 and −0.42, *p* < 0.001 and *p* < 0.001, respectively) and lung adenocarcinomas (Spearman correlation test, rho −0.47 and − 0.3, *p* < 0.001 and *p* < 0.001, respectively) (Fig. 3d, e).

### Cancer-specific super-enhancers coincide with regional hypomethylation

**Fig. 4:**
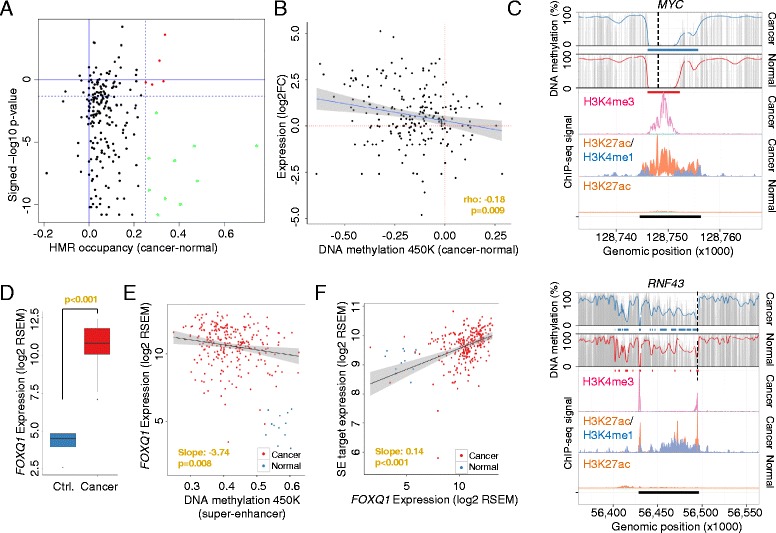
Hypomethylation at cancer-related super-enhancers in colorectal tumors. **a** Differential DNA methylation (occupancy of hypomethylated regions (*HMRs*)) at colorectal cancer-related super-enhancers between normal mucosa and primary colorectal cancer samples (WGBS, x-axis). Differentially methylated super-enhancers are indicated (*colored dots*, δ HMR occupancy >25 %). Results were validated in a cohort of matched normal and primary colorectal tumor samples (TCGA, n = 41, HumanMethylation450 BeadChip) and significant differences assessed by the Wilcoxon test (*green dots*, *p* < 0.05, y-axis). **b** Hypomethylation at super-enhancers was associated with increased target gene expression analyzed by HumanMethylation450 BeadChip (450 K, x-axis) and RNA-seq (y-axis) in matched primary colorectal cancer samples (n = 12, TCGA). Expression data are displayed as log transformed fold-change (*log2FC*). **c** DNA methylation profiles of the super-enhancer regions associated with *MYC* and *RNF43* in normal and colorectal cancer samples (WGBS). Smoothed (*colored line*), raw (*gray bars*) CpG methylation levels, hypomethylated regions (*colored bars*) and super-enhancers (*black bars*) are indicated. The enhancer-related histone marks H3K27ac (*orange*) and H3K4me1 (*blue*) and the promoter-related mark H3K4me3 (*pink*) are displayed as ChIP-seq signal intensities (*bottom panels*) [11]. The transcription start sites are indicated (*broken line*). **d** Gene expression levels of the transcription factor *FOXQ1* in normal (*blue*) and colorectal cancer (*red*) samples (TCGA). **e, f** Association of *FOXQ1* expression and DNA methylation levels (HumanMethylation450 BeadChip, 450 K) at hypomethylated super-enhancer regions (**e**) or expression levels of associated target genes (**f**) in colorectal cancer in normal (*blue*) and colorectal cancer (*red*) samples (TCGA). Significance was assessed from a linear regression model applied solely to the cancer samples. *RSEM* RNA-Sequencing by Expectation Maximization

Until now we have focused our attention on those sequences described as being super-enhancers that ensure cell and tissue identity in normal tissues [8, 11]. However, a new class of super-enhancer sequences has recently been described that only play this de novo regulatory role in transformed cells to drive the cancer phenotype and its associated hallmarks [11, 13, 37]. We examined the DNA methylation changes occurring in the super-enhancers of colorectal cancer (HCT-116, n = 387), in which we obtained 99 % coverage using our WGBS approach. We observed that these newly developed tumor-related super-enhancers were associated with DNA hypomethylation events (n = 23, δ HMR occupancy >25 %) at these sequences in the transformed cells compared with normal colorectal mucosa (Fig. 4a; Table S8 in Additional file 1). Most notably, the super-enhancer hypomethylation shift was independent of the global loss of DNA methylation generally found in cancer cells (paired *t*-test, *p* < 0.001) [5, 6, 30] and rather represented a focal DNA demethylation event within the super-enhancer regions (Figure S12 in Additional file 1). As we did with the aforementioned normal tissue super-enhancers, we validated the DNA hypomethylation changes in these de novo cancer super-enhancers using a cohort of matched normal colon and primary colorectal tumors (TCGA [17], n = 41) analyzed by DNA methylation microarrays (Fig. 4a; Table S8 in Additional file 1). Noteworthy, we again excluded potential biases included by CNV in these regions (Table S8 in Additional file 1). In this setting, we further confirmed that the loss of DNA methylation in these emerging cancer super-enhancers was significantly associated with an increase in expression of the corresponding regulated genes in the primary colon tumors in comparison with the matched normal colon mucosa (TCGA [17]; Spearman’s correlation test, rho −0.18, *p* = 0.009; Fig. 4b). Examples within the most hypomethylated cancer super-enhancers include those sequences regulating the *MYC* and *RNF43* [38] oncogenes (Fig. 4c; Figure S13a, b in Additional file 1), regions not affected by CNV in the primary colorectal cancer sample analyzed by WGBS (Table S8 in Additional file 1). Importantly, DNA methylation changes affected solely regions specifically marked by H3K27ac in colon cancer and widely excluded H3K4me3, further indicating that alterations in super-enhancers occur predominantly distal to the core promoter regions (Fig. 4c).

An interesting matter arising from these results is their value for identifying putative mechanisms that create such specific patterns of oncogenic super-enhancer hypomethylation. It has been proposed that the availability and binding of transcription factors (TFs) to regulatory regions might be able to impact on the DNA methylome and that it is not the transcriptional activity per se that alters the DNA methylation profile of regulatory elements [20, 21]. Herein, we have studied the putative enrichment of TF binding sites in these colorectal cancer-specific hypomethylated enhancers and we observed a significant enrichment for specific TF binding motifs (Figure S14a in Additional file 1). From these factors, specifically FOXQ1 (forkhead box Q1; *p* = 0.013), a member of the FOX gene family that is involved in tumorigenesis [39], was the most overexpressed TF in primary colorectal cancer samples and showed multiple binding sites (Table S8 in Additional file 1) and a significant enrichment at hypomethylated super-enhancer loci (Figure S14b in Additional file 1). In relation to this point, *FOXQ1* had a 73-fold greater expression in primary colorectal cancer samples than in matched control samples (TCGA [17]; Wilcoxon test, *p* < 0.001; Fig. 4d). Furthermore, the stronger *FOXQ1* expression was significantly associated with hypomethylation of the previously defined super-enhancers (linear slope −3.74, *p* = 0.008; Fig. 4e) and the activation of associated target genes (linear slope 0.14, *p* < 0.001; Fig. 4f), such as the well-known oncogenes *MYC* and *RNF43* (Figures. S15a, b and S16a, b in Additional file 1). Interestingly, the presence of cancer-specific super-enhancer hypomethylation and the tumorigenic effect mediated by the presence of FOXQ1 binding sites could be useful for identifying new candidate oncogenes, such as *GPRC5A* (G protein-coupled receptor, class C, group 5, member A; Figures. S13c, d, S15c and S16c in Additional file 1), which, by mediating between retinoid acid and G protein signaling pathways, has a role in epithelial cell differentiation [40].

Importantly, we experimentally validated the association between *FOXQ1* expression and target gene regulation in a colorectal cancer cell line model system (HCT116 and SW1116 cancer cell lines). Initially, we confirmed the occupancy of FOXQ1 at binding sites within the super-enhancer regions of the previous described target genes *MYC*, *RNF43* and *GPRC5A* (Figure S17a in Additional file 1). Furthermore, following small hairpin RNA (shRNA)-mediated knockdown of the TF, we observed significant downregulation of *MYC*, *RNF43* and *GPRC5A*, suggesting a direct regulatory role of FOXQ1 (Figure S17b in Additional file 1). In line with the oncogenic role of FOXQ1 targets in colorectal cancer settings, knockdown of the TF reduced cell proliferation of the colorectal cancer cell line (Figure S17c in Additional file 1). Remarkably, in addition to FOXQ1, we could also experimentally confirm the regulatory effect of other enriched TFs, whose expression correlated significantly with super-enhancer hypomethylation level (*p* < 0.05; Figure S14b in Additional file 1). Specifically, we experimentally confirmed the regulatory effect of the TFs *HNF4A* and *PPARG* on *RNF43* and *GPRC5A* expression (Figure S18a, b in Additional file 1). Herein, knockdown of the TFs repressed *RNF43* and *GPRC5A* expression (Figure S18c in Additional file 1) and resulted in reduced cell viability (Figure S18d in Additional file 1), further supporting the accuracy of the functional prediction based on super-enhancer DNA methylation levels (Figure S14b in Additional file 1).

Further, we were interested if disruption of the super-enhancer structure would interfere with the DNA methylation levels in the respective regions. Therefore, we treated the colorectal cancer cell lines HCT116 and SW1116 at sub-lethal concentrations with the BET-bromodomain inhibitor JQ1, a small molecule targeting BRD4, a key component of the secondary super-enhancer structure (Figure S19a, b in Additional file 1) [13]. Interestingly, although the treatment with JQ1 decreased the expression of super-enhancer gene targets, such as *MYC*, *RNF43* or *GPRC5A*, we could not detect an effect on DNA methylation levels at super-enhancer-related CpG sites (Figure S19c, d in Additional file 1). The lack of DNA methylation variance following JQ1 treatment suggests that the secondary super-enhancer structure per se is not a determinant of DNA methylation profiles, but that it is the binding of TFs to the DNA that locally establishes CpG methylation levels.

### Large-scale hypomethylation marks potential cancer drivers

**Fig. 5:**
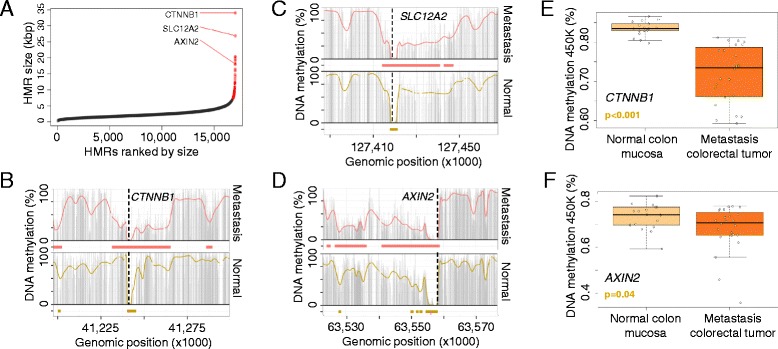
Large hypomethylated regions in colorectal metastasis. **a** HMRs derived from the metastatic colorectal cancer sample ranked by genomic size. Large HMRs are indicated (*red dots*). **b–d** DNA methylation profile of the HMRs spanning *CTNNB1* (**b**), *SLC12A2* (**c**) and *AXIN2* (**d**) in the normal (*yellow*) and metastatic (*red*) samples. Smoothed (*colored line*), raw (*gray bars*) CpG methylation levels and hypomethylated regions (*colored bars*) are indicated. **e, f** Validation of the large HMRs associated with *CTNNB1* (**e**) and *AXIN2* (**f**) using the HumanMethylation450 BeadChip. Displayed are average CpG methylation levels of *CTNNB1* and *AXIN2* for 18 normal colon mucosa and 24 colorectal metastasis samples. Significant differences were assessed using Student’s *t*-test

Finally, we wondered whether DNA methylation data obtained from WGBS could be used to identify new candidate cancer regulatory regions beyond the histone-based super-enhancer loci [8, 11]. In line, extended hypomethylated regions were previously established as important regulatory elements in hematopoietic cells with a function in leukemogenesis [41]. To test this hypothesis, we ranked all the de novo formed hypomethylated DNA regions (<20 % average DNA methylation) in our colorectal cancer samples by size, having shown above that HMRs in colorectal tumorigenesis presented locus-specific properties (Figure S8b and Table S5 in Additional file 1). In this setting, we did observe an unequal distribution of HMR sizes, as previously reported for the super-enhancer-defining mark H3K27ac (Fig. 5a). Importantly, these large HMRs were mutually exclusive to the presence of super-enhancers in the respective regions, suggesting they represent an independent epigenetic feature to histone defined regulatory elements. Intriguingly, large HMRs mainly spanned gene promoter regions (22/26; Table S9 in Additional file 1), a phenomenon previously described for genes activated in medulloblastoma patients, where an extensive expanded hypomethylation beyond the proximal promoter was observed, which might be a general feature of cancer-related gene activation [42]. Further, most of the HMRs that were present only in the metastatic cancer samples presented features suggesting a role in tumorigenesis. For example, the largest observed HMR (34.1 kb) in the metastatic colorectal cancer sample corresponded to beta-catenin (*CTNNB1*), a key component of the WNT pathway and driver of epithelial–mesenchymal transition (Fig. 5b) [43]. *AXIN2*, another key member of the WNT signaling pathway [44], was also among the top identified HMRs and is, together with an additional illustrative example, displayed in Fig. 5c, d. Importantly, these findings were validated in an independent cohort of colorectal metastasis samples (n = 24) using DNA methylation microarray analysis (Student’s *t*-test, *p* < 0.05; Fig. 5e, f). Thus, these findings suggest that large cancer-specific HMRs are likely candidate markers for identifying sequences that could act as de novo activators in a super-enhancer-like manner.

## Conclusions

Overall, our findings indicate that super-enhancers, regulatory regions critical for cell identity and function, are partially regulated by their CpG methylation status in normal cells, and that they are targeted by specific aberrant DNA methylation events in cancer, with putative effects for the expression of the downstream-controlled genes. Further, we determined spatial differences of healthy and transformed DNA methylation profiles within these large enhancer clusters, suggesting local differences in activity in super-enhancer regions.

We hypothesize that local changes in TF binding act on super-enhancer DNA methylation profiles with subsequent effects on target gene expression. Accordingly, super-enhancer DNA methylation levels indicate regulatory activity and, moreover, point to implicated TFs. In cancer, the perturbed expression of key TFs establishes novel super-enhancers that drive oncogene expression, a scenario that we partially delineated through the identification of FOXQ1 as a putative factor driving the differential DNA methylation at colorectal cancer-specific super-enhancers and the overexpression of key oncogenes, such as *MYC* and *RNF43*.

Our results also emphasize that developing more extensive catalogues of human DNA methylomes at base resolution would help us gain a better understanding of the regulatory functions of DNA methylation beyond those of the most widely studied proximal promoter gene regions.

## Materials and methods

### Whole genome bisulfite sequencing

Cancer cell lines were obtained from the American Type Culture Collection (ATCC) and cultivated according to the provider’s recommendations. All primary samples analyzed in this study were approved for research use by the respective ethics committees and were evaluated by trained personal before entering this study. DNA from cell lines or fresh frozen healthy and tumor samples was extracted using Phenol:Chloroform:Isoamylalcohol (Sigma).

We spiked genomic DNA (1 or 2 μg) with unmethylated λ DNA (5 ng of λ DNA per μg of genomic DNA) (Promega). We sheared DNA by sonication to 50–500 bp with a Covaris E220 and selected 150- to 300-bp fragments using AMPure XP beads (Agencourt Bioscience Corp.). We constructed genomic DNA libraries using the TruSeq Sample Preparation kit (Illumina Inc.) following Illumina’s standard protocol. After adaptor ligation, we treated DNA with sodium bisulfite using the EpiTect Bisulfite kit (Qiagen) following the manufacturer's instructions for formalin-fixed and paraffin-embedded tissue samples. We performed two rounds of conversion to achieve >99 % conversion. We enriched adaptor-ligated DNA through seven cycles of PCR using the PfuTurboCx Hotstart DNA polymerase (Stratagene). We monitored library quality using the Agilent 2100 BioAnalyzer and determined the concentration of viable sequencing fragments (molecules carrying adapters at both extremities) by quantitative PCR using the Library Quantification Kit from KAPA Biosystems. We performed paired-end DNA sequencing (two reads of 100 bp each) using the Illumina HiSeq 2000.

Sequencing quality was assessed using the Illumina Sequencing Analysis Viewer and FastQC software (http://www.bioinformatics.babraham.ac.uk/projects/fastqc). We ensured the raw reads used in subsequent analyses were within the standard parameters set by the Illumina protocol. Positional quality along the reads was confirmed to be QC > 30, and we excluded biases towards specific motifs or GC-enriched regions in the PCR amplification or hybridization. Sequence alignment and DNA methylation calling of WGBS reads were performed using Bismark V.0.7.4 software [45]. SAM/BAM and BED file handling was done using SAMtools [46], BEDtools [47] and Tabix [48]. Statistical analysis and graphical representation were performed with R [49] and multicore and ggplot2 libraries. We smoothed the DNA methylation profiles using a previously described method for processing WGBS data [50]. Briefly, the method assumes that the DNA methylation profile is defined by a varying function of the genomic location that can be estimated with a local likelihood smoother. We used hg19 as the reference genome and retrieved genomic information from Biomart [51] and GENCODE V.16 [52]. The TSS was considered to be the most upstream base of all the annotated transcript variants of the gene. The DNA methylation data sets for the two breast cancer cell lines (MDA-MB-468PT and MDA-MB-468LN) were previously published and are available under accession code GSE56763, Gene Expression Omnibus (GEO).

### Hypomethylated regions

HMRs were identified as previously described [22]. Briefly, the raw methylated and unmethylated read counts of each CpG site, modeled with a beta-binomial distribution, provided the input for a hidden Markov segmentation model with two states (high and low methylation). Subsequently, a score was computed for each identified hypomethylated region as the number of CpG sites minus the sum of their methylation values. Further, the resulting regions were filtered on the basis of the 99^th^ percentile of the score obtained by randomly permuting CpG sites. Differential DNA methylation in super-enhancers was calculated as difference (δ) in HMR occupancy (regions overlapping HMRs) between two samples.

In order to identify large HMRs, we followed a similar strategy to that described for identifying histone mark-defined super-enhancers [11], identifying regions that are substantially larger than their normal counterparts. We initially extracted HMRs with an average smoothed DNA methylation level of <0.2 and sorted the regions by genomic size. Secondly, we scaled the size and sorting index to map them to values over a 0–1 range. We then plotted the scaled region size (y axis) against the scaled region index (x axis) and examined a subset of the data (above the 90^th^ percentile of size, high-scaled region index) and fitted a linear model with the log of the scaled size as outcome and the logistically transformed scaled index as predictor. Using the fitted parameter values, we reverted the variable transformation and identified the region index for which the derivative of the curve was 1 (i.e., a line with slope of 1 was the tangent to the curve). HMRs above this point were defined as large HMRs. This procedure was performed for each sample separately.

### DNA methylation of super-enhancers

Super-enhancer coordinates were obtained from [11]. For the set of genomic regions defined as super-enhancers, we extended to each side by 50 % of the total length to include equally sized flanking regions in downstream analyses. Further, we scaled the position of each region to the center (0), the edges of the original region (−1 and 1), and the edges of the extended region (−2 and 2). We then retrieved the smoothed methylation information for each CpG inside the super-enhancers and flanking regions. Differential DNA methylation levels inside super-enhancers and flanking regions were analyzed by Fisher’s exact test, classifying CpGs as hypomethylated (<0.33 DNA methylation) or hypermethylated (>0.66 DNA methylation). Tissue-specificity of the DNA methylation profiles within super-enhancers was determined by assessing the tissue-matched DNA methylation profile, as described above, and their characteristics in an unmatched tissue context. Differences in DNA methylation (flanking region versus super-enhancer region) between tissues were analyzed by ANOVA.

WGBS-based tissue-specific hypomethylated super-enhancers were defined by identifying super-enhancers with an absolute HMR occupancy >20 % and a difference in HMR occupancy between the corresponding tissue and the remaining normal tissues >10 %. Each of these selected regions was considered as validated if the average beta value (HumanMethylation450 BeadChip) in the corresponding tissue samples was <33 % and the Student’s *t*-test FDR comparing the corresponding tissue samples against the remaining samples was <0.05.

ChIP-sequencing data of the histone mark H3K27ac were retrieved from [11]. We computed the H3K27ac signal (ChIP versus input) and averaged the smoothed DNA methylation values in 50-bp windows. To define associations between histone signals and DNA methylation, we performed a Wilcoxon rank-sum test for the H3K27ac signal between hypomethylated (average <0.33) and hypermethylated (average >0.66) windows. Subsequently, we fitted a multivariate linear model with H3K27ac signal as response variable, DNA methylation status (hypo/hyper) and CpG density as predictors to assess the impact of CpG density on the association.

Differential DNA methylation analysis in cancer was done by computing the proportion of super-enhancers covered by HMRs. For each cancer sample and super-enhancer, we calculated the difference in HMR occupancy (δ HMR; cancer versus corresponding normal tissue). In order to assess overall differences between normal and cancer samples in super-enhancers, we performed a paired *t*-test for the reduction in DNA methylation (DNA methylation flanking super-enhancers versus DNA methylation inside super-enhancers) between the normal and cancer samples.

### Expression analysis

The relationship between DNA methylation and gene expression was assessed using data obtained from RNA sequencing and public data sets. Raw RNA sequencing FASTQ reads from the breast cancer cell lines (MCF10A, MDA-MB-468PT and MDA-MB-468LN) were aligned against the human hg19 reference sequence using the TopHat read-mapping algorithm [53]. Conversion to BAM format was carried out using SAMtools [46]. Counts of alignments for each gene using BAM files were generated using BEDtools multicov [47]. In a subsequent analysis, the non-transformed cell line MCF10A was considered as control. Data from primary tumor samples were obtained from TCGA data portal (https://tcga-data.nci.nih.gov/tcga/). The analyzed samples included 110 normal breast samples and 30 matched invasive breast carcinomas (BRCAs), 12 normal colon and 258 adenocarcinomas (COADs), and 57 matched normal lung and adenocarcinomas (LUADs). To study the association of super-enhancer DNA methylation and gene expression, we obtained TCGA RNA-sequencing data (level 3) at the gene level and performed a Spearman’s correlation test. Correlation analysis of gene expression and differential DNA methylation (normal versus cancer, δ > 0.1) were performed using a Spearman’s correlation test. Alternatively, we assigned the super-enhancers to the closest gene TSS, excluding those super-enhancers without a TSS within 1 Mb. We fit a log-linear model with RNA-Sequencing by Expectation Maximization-normalized gene expression as the response variable and average super-enhancer DNA methylation as predictor. The association between differential super-enhancer DNA methylation and gene expression was determined by fitting a linear model with the log fold-change of gene expression (cancer versus normal) as response and the δ HMR occupancy for all the super-enhancers gaining DNA methylation (δ HMR occupancy >0 %) or by Spearman’s correlation test.

For microRNA quantification the Taqman microRNA Reverse Transcription kit and microRNA specific Taqman assays (Applied Biosytems) were used. The expression level was evaluated by real-time quantitative PCR using the 7900HT Fast Real-Time PCR System (Applied Biosystems). Expression values are reported as relative microRNA expression levels normalized to RNU6B expression.

### Infinium HumanMethylation450 BeadChip

DNA from fresh frozen healthy and tumor samples was extracted using phenol:chloroform:isoamylalcohol (Sigma). All DNA samples were assessed for integrity, quantity and purity by electrophoresis in a 1.3 % agarose gel, picogreen quantification, and nanodrop measurements. All samples were randomly distributed into 96-well plates. Bisulfite conversion of 500 ng of genomic DNA was done using the EZ DNA Methylation Kit (Zymo Research), following the manufacturer’s instructions. Bisulfite-converted DNA (200 ng) were used for hybridization on the HumanMethylation450 BeadChip (Illumina).

The HumanMethylation450 BeadChip data were processed using the Bioconductor minfi package [54]. We performed the “llumina” procedure that mimics the method of GenomeStudio (Illumina); specifically, it performs a background correction and a normalization taking as a reference the first array of the plate. We removed probes with one or more single nucleotide polymorphisms (SNPs) with a minor allele frequency (MAF) >1 % (1000 Genomes) in the first 10 bp of the interrogated CpG, based on [55]. In order to minimize batch effect, we used ComBat normalization [56]. The methylation level (β) for each of the 485,577 CpG sites was calculated as the ratio of methylated signal divided by the sum of methylated and unmethylated signals plus 100. After the normalization step, we removed probes related to X and Y chromosomes. All analyses were performed in human genome version 19 (hg19).

We identified HMRs within super-enhancer-overlapping probes (≥3) on the BeadChip and computed the average DNA methylation level for super-enhancers (HMR located probes) per sample (tissue-wise). Differences in DNA methylation levels at hypomethylated super-enhancer regions were determined using Student’s *t*-test (FDR < 0.05). Selected super-enhancers were hierarchically clustered using Manhattan distance and median clustering algorithms. Finally, we assessed the BeadChip-based CpG methylation levels of common differentially methylated super-enhancers and performed hierarchical clustering using Canberra distance and Ward clustering algorithms with CpG-level data. The DNA methylation data for lung adenocarcinomas and lung squamous cell carcinomas were previously published and are available under accession code GSE39279, Gene Expression Omnibus (GEO).

The DNA hypomethylation observed at cancer-related super-enhancers was validated using data obtained from TCGA data portal (https://tcga-data.nci.nih.gov/tcga/). The analyzed samples included 41 matched normal and colorectal cancer samples. We obtained TCGA DNA methylation data from the HumanMethylation450 BeadChip (level 3) and averaged DNA methylation levels per super-enhancer containing ≥3 probes in the hypomethylated region. Significant differences between normal and cancer samples were assessed using a Wilcoxon test, with values of *p* < 0.01 considered to be significant.

### CNV analysis

To test for biases in DNA methylation analysis due to CNV in cancer samples, we applied two independent approaches based on DNA methylation or SNP array data. For the 714 primary cancer samples analyzed using the HumanMethylation450 BeadChip, we performed a copy number analysis comparing cancer and normal samples using Bioconductor and the CopyNumber450K R package for CNV inference using the Illumina 450 k DNA methylation assay. We defined a region to be aberrant if >50 % of the region presented a significant copy number alteration as reported by the software (FDR < 0.05). Alternatively, for TCGA data set of colorectal adenocarcinomas [17], we used level 3 CNV data and defined a region to be aberrant if >50 % of the super-enhancer region presented copy numbers <1.5 or >2.5. For the WGBS cancer samples, we hybridized genomic DNA on the HumanOmni5 SNP array (Illumina) and performed a copy number analysis based on GenomeStudio software (V.2011.1) routines for the HumanOmni5-4v1_B chips.

### Ethics

The Clinical Research Ethics Committee of the Bellvitge University Hospital approved the current study under the reference PR055/10. All patients who supplied the primary tumor samples have given written informed consent. The experimental methods comply with the Helsinki Declaration.

### Availability of supporting data

The bisulfite sequencing data sets supporting the results of this article are available in the NCBI Sequence Read Archive (SRA; http://www.ncbi.nlm.nih.gov/sra) under accession number SRP033252 and to the NCBI Gene Expression Omnibus (GEO; http://www.ncbi.nlm.nih.gov/geo/) under accession number GSE52272). All HumanMethylation450 BeadChip data from this study are available in GEO under accession number GSE52272.

## Additional file

Additional file 1:
**A PDF document containing Supplementary methods, Figures. S1–S19 and Tables S1–S9.** (PDF 7013 kb)

## Abbreviations

5-hmC: 5-Hydroxy methylation
5-mC: 5-Methylation
BS: Bisulfite
ChIA-PET: Chromatin interaction analysis by paired-end tag
ChIP: Chromatin immunoprecipitation
CNV: Copy number variation
FDR: False discovery rate
GEO: Gene Expression Omnibus
HMR: Hypomethylated region
OR: Odds ratio
ox-BS: Oxidative bisulfite
PCR: Polymerase chain reaction
shRNA: Small hairpin RNA
SNP: Single nucleotide polymorphism
TCGA: The Cancer Genome Atlas
TF: Transcription factor
TSS: Transcription start site
WGBS: Whole genome bisulfite sequencing
