# Supplementary material for: Epigenomic analysis detects aberrant super-enhancer DNA methylation in human cancer
Source: Genome Biol. 2016 Jan 26;17:11. doi: 10.1186/s13059-016-0879-2 (PMC4728783; doi:10.1186/s13059-016-0879-2)
Supplement: Additional file 1: — A PDF document containing Supplementary methods, Figures. S1–S19 and Tables S1–S9. (PDF 7013 kb) [file 13059_2016_879_MOESM1_ESM.pdf]

SUPPLEMENTARY INFORMATION

SUPPLEMENTARY METHODS
SUPPLEMENTARY FIGURES 1-19
SUPPLEMENTARY TABLES 1-9

SUPPLEMENTARY METHODS

Functional enrichment analysis. Gene Ontology (GO) enrichment analysis was performed using the Database for Annotation, Visualization and Integrated Discovery (DAVID; v6.7) [1, 2]. The results were corrected for multiple hypotheses testing using the Bonferroni p-value adjustment method.

ChIA-PET Data Integration

We retrieved MCF7 Pol2 ChIA-PET data from [3] (4 replicates) and kept anchors that were present in >1 replicate and where any of the anchor pairs overlapped a gene promoter (consensus anchor regions). Then, SEs not overlapping promoters were assigned to consensus ChIA-PET anchor regions. Finally, we assessed the association between SE enhancer DNA methylation and target gene expression with a Spearman's correlation test using TCGA data from normal breast samples [4].

5-Hydroxy DNA Methylation Profiling

Genomic DNA was quantified using Quibit fluorometer (Qubit® dsDNA BR Assay Kit). 4 µg of DNA in 150µL were sheared using g-TUBE (Covaris, Inc.) to 10kbp fragments, by centrifugation at RT for 1 min at 6,000 rpm (Eppendorf 5424). g-TUBEs were then inverted in the centrifuge, and centrifuged again for 1 min at 6,000 rpm (RT). Sheared DNA was recovered from the screw-cap, and processed through GeneJET purification kit to reduce volume from 150µL to 40µL. GeneJET columns were prepared with 50 mM NaOH twice, followed by Binding Buffer. Once GeneJET columns were prepared, 150µL of sheared DNA were processed on GeneJET columns, followed by 3 washes with 80% acetonitrile. DNA was recovered from the columns using 40µL of pre-warmed ultra-pure water (65°C). Concentrated sheared DNA was then quantified using Qubit, and all samples were spiked-in with 0.5% (w/w) of Digestion Control according to the quantification of the DNA.

Each sample was divided in two aliquots of 20µL each, and were processed following TrueMethyl 24 Kit User Guide (Version 3.1 July 2013, CEGX, UK). The aliquot intended for the oxidative bisulfite conversion (OxBs), underwent a pre-cleaning step, using BioRad P6 Micro-Bio spin columns to remove possible contaminants that could interfere the oxidation reaction. Then both aliquots were denatured for 30 min at 37°C with Denaturing Solution provided in the kit, and immediately kept on ice. To each aliquot intended for OxBs, 1µL of Oxidant Solution was added, while the aliquots intended for normal bisulfite conversion (Bs) 1µL of water was added instead. Samples

were incubated for 30 min at 40°C, and centrifuge 10 min at 14,000 x g, to remove precipitates. Supernatant was then used for bisulfite conversion process by adding 5µL of Bisulfite Additive and 170µL of Bisulfite Reagent, and incubated 2 cycles in a PCR machine (5 min @ 95°C; 20 min @ 60°C; 5 min @ 95°C; 40 min @ 60°C; 5 min @ 95°C; 165 min @ 60°C).

Bisulfite converted DNA was clean-up by pelleting precipitated salts, then subjected to a desulfonation reaction and finally clean-up again using Amicon Ultra 0.5 30kDa filter columns as indicated on the protocol with the modification on step 4.15 to increase the centrifugation time to 65 min, as indicated on TrueMethyl workflow for 450k Analysis (CEGX Version 1.1 December 2013). Both aliquots were quantified using Qubit ssDNA Assay Kit and 160 ng of bisulfite converted DNA were processed following standard protocol for HumanMethylation450 microarray.

Raw methylation data (idats) were background corrected and normalized using Methylation module (v1.9.0) of Illumina GenomeStudio software (V2011.1) in order to compute β-values. Hydroxymethylation levels were computed by subtracting β-values resulting from OxBs aliquot (5hmC+5mC) to the β-values from Bs aliquot (5mC).

Transcription Factor Occupancy Analysis

We used the ENCODE transcription factor binding site (TFBS) data available at UCSC, comprising 91 cell lines and 188 antibodies [5]. Given a sample and its corresponding super-enhancer (SE), we extended each SE 50 % of its size on each direction and split the resulting regions in 60 windows according to the relative distance to the center of the SE. We then computed the average methylation and the proportion covered by any of the TFBS (occupancy) among the window for each window and SE. As the distribution of both methylation and TFBS occupancy was bimodal, we categorized these values using a cutoff of 50 % and performed a Fisher's exact test separately for the windows inside the SE.

Transcription Factor Enrichment Analysis

The statistically over-represented transcription factor binding sites (TFBS) in a set of sequences were compared with a background set using the CLOVER algorithm (Cis-eLement OVERrepresentation) [6] employing the following procedure: the JASPAR 2009 CORE collection of TFBS pattern matrices [7] were downloaded and converted to CLOVER format using PERL scripts, then the subset of sequences to search for over-represented TFBS and the background of sequences with which to compare them were specified. Specifically, we defined the hypomethylated colon cancer-related SE regions as the target set and the entire set of colon cancer-related SE loci as the background

set. CLOVER compares each motif in turn with the sequence set and calculates a raw score that indicates how well represented the motif is in the subset. CLOVER also determines the statistical significance of the raw scores. Therefore, for the background set, CLOVER repeatedly extracts random fragments matched by length to the target subset of sequences, and calculates raw scores for these fragments. The proportion of times that the raw score of a fragment set exceeds or equals the raw score of the target set, is taken as the probability, P, that the motif's presence in the target set can be explained by chance alone. For each motif, a separate probability is calculated for each background file. Values of $P<0.05$ were considered to be statistically significant.

Chromatin Immunoprecipitation (ChIP) to Assess FOXQ1 Occupancy at Binding Sites and Super-enhancers Regions

Previous to ChIP, FOXQ1 monoexonic cDNA (lacking UTR regions) was amplified from HCT116 genomic DNA using specific primers with end adaptors containing EcoRI and BamH1 sequences, a Kozak sequence, and the N-terminal flag-tag (DYKDDDDK). The PCR products were cloned in bacteria, polymorphisms and mutations verified by Sanger-sequencing, and ligated into plvx-IRES-zsgreen plasmid from Clontech using EcoRI and BamH1 restriction enzymes (refseq: NM_033260). 10 µg of each plasmid were mixed with 7.5 µg of PS-PAX2 and 2.5 µg of PMD2.G plasmid in 1 ml jetPRIME buffer and 50 µl of jetPRIME reagent were added (114-15, Polyplus transfection). After 20 min of RT incubation, the mix was diluted over a 10 cm disk containing 10 ml of DMEM and 293T cells at 80% confluence. After 48 h, medium containing viruses was recovered and 45-µm filtered. 3 ml of this medium plus polybrene (8 µM) was added to six-well plates containing the host cells at 80% confluence. After 48-72 h, cells were expanded and green positive cells were isolated by flow cytometry. Ectopic expression of FOXQ1 protein was evaluated by western blot using anti-Flag-HRP antibody (A8592, Sigma).

For ChIP, fresh cultures (1.5-2.0 x 10^7 cells) were cross-linked with 1% formaldehyde for 8 min and the reaction was blocked by adding glycine to a final concentration of 0.125 M. After washing twice with ice-cold PBS, cell pellets were resuspended in 1ml of Farnham lysis buffer (PIPES 5mM pH8.0, KCl 85mM, NP-40 0.5%) supplemented with protease inhibitor cocktail (Complete EDTA-free, Roche) and kept on ice for 10 min. The nuclear pellet was then resuspended in 1ml RIPA buffer (Tris-HCl 50 mM pH8.0, EDTA 20 mM, SDS 1%) supplemented with protease inhibitor cocktail (Complete EDTA-free, Roche) and kept on ice for 10 min. Samples were subsequently sonicated with S220 Covaris ultrasonicator for 18 min (peak incident power: 75W, duty factor: 10%, cycles per burst: 200). The chromatin size of the fragments obtained was 250-

500 bp. Samples were diluted with dilution buffer (SDS 0.01%, Triton X-100 1.1%, EDTA 1.2 mM, NaCl 165 mM, Tris-HCl 16.7 mM pH 8.1). Magnetic beads were used for pre-clearing diluted chromatin (overnight at 4ºC) and for incubation with anti-Flag antibody (F1804, Sigma). Non-related mouse IgG antibody (12-371B, Millipore) was used as a negative control. The bead-antibody complexes were then incubated with pre-cleared chromatin for 8 h at 4ºC with rotation. The immune complexes were washed at 4ºC in rotation: twice with low-salt buffer (Tris-HCl 50 mM pH 8.0, NaCl 150 mM, SDS 0.1%, NP-40 1%, EDTA 1 mM, deoxycholate Na 0.5%), twice with high-salt buffer (Tris-HCl 50 mM pH 8.0, NaCl 500 mM, SDS 0.1%, NP-40 1%, EDTA 1 mM, deoxycholate Na 0.5%), twice with LiCl buffer (Tris-HCl 50 mM pH 8.0, LiCl 250 mM, SDS 0.1%, NP-40 1%, EDTA 1 mM, deoxycholate Na 0.5%) and once with TE Buffer (Tris-HCl 10 mM pH 8.0, EDTA 0.25 mM), 2 min each. Cross-linked chromatin was then eluted from the magnetic beads by adding elution buffer ($NaHCO_3$ 100 mM, SDS 1%). Samples were de-crosslinked overnight at 65ºC and incubated with proteinase K at 50 µg/ml final concentration for 1 h. Finally, DNA was purified with a PCR purification kit (28106, Qiagen). The following SybrGreen gene-specific primer pairs were used: MYC forward, 5'- GGATTTTTCCAATGGACACG-3', MYC reverse: 5'-AAACAAAGCCAGACCTCAGC-3'; GPR forward: 5'- TCTTCTCATTCTGGGTCCACT-3', GPR reverse: 5'- GGAAGTCAAAGATTCCTCAAGCA-3'; RNF forward: 5'-GCTTCCGTTTCAGAAAGCCA-3', RNF reverse: 5'- TCCTCTTCTCTGCCCAATCA-3'. Primer amplification efficiency and primer dimer formation was tested previously to ChIP. Western blot from ChIP pull downs were run to evaluate the presence of FOXQ1. ChIP data are presented as percentage of input ± SEM, n ≥ 3. Significance of Student's *t*-tests (equal variance, one tail) is shown.

Experimental Validation of Transcription Factor Effects

For qRT-PCR experiments, total RNA from the colorectal cancer cell line SW1116 transfected with shRNAs against the respective transcription factors or a scrambled control was extracted using MAXwell (Promega) and retro-transcribed using the ThermoScript™ RT–PCR System (Invitrogen). Gene expression was determined by quantitative real-time PCR using SYBR Green (Applied Biosystems) according to the manufacturer's recommendations. Target gene expression levels were normalized to two housekeeping genes (PPIA and B2M).

Cell proliferation was determined for the colorectal cancer cell line SW1116 transfected with shRNAs against the respective transcription factors or a scrambled control by a 3-(4,5-dimethylthiazol-2-yl)-2,5-diphenyltetrazolium bromide (MTT) assay. The cell viability was quantified over 6 days, staining the cells with MTT for 3 hours and

blocking the reaction adding lysis buffer (HCl 20 mM, acetic acid 2.5%, SDS 20%, dimethylformamide 50%, pH 4.7). Measurements were performed at 560nm after overnight incubation at 37ºC.

Super-enhancer disruption with JQ1

After overnight incubation, the colorectal cancer cell lines HCT116 and SW1116 were treated with a 2-fold dilution series of JQ1 (A1910, Apexbio) or vehicle alone (DMSO at final concentration 0.2%). After 48h, cell viability was determined by a 3-(4,5-dimethylthiazol-2-yl)-2,5-diphenyltetrazolium bromide (MTT) assay. To determine sublethal concentrations, we calculate the EC50 for JQ1 for both cell lines using the mean of four replicates in respect to vehicle treated cells. Viability curves were generated using a sigmoidal dose-response model with variable slope (GraphPad Prism 5 software) from which the EC_{50} values were extracted. To investigate the effect of JQ1 treatment at the DNA methylation level, HCT116 and SW1116 cells were treated with a sub-lethal JQ1 concentration (10 µM) for 48h. After 24h, culture medium and drug were renewed and cells were harvested 24h later. RNA and DNA were extracted to evaluate mRNA expression level changes of *MYC*, *RNF43* and *GPRC5A* and for DNA methylation analysis on the Infinium HumanMethylation450 BeadChip, respectively.

SUPPLEMENTARY REFERENCES

1. Huang DW, Sherman BT, Lempicki RA: Systematic and integrative analysis of large gene lists using DAVID bioinformatics resources. *Nat Protoc* 2009, 4:44–57.

2. Huang DW, Sherman BT, Lempicki RA: Bioinformatics enrichment tools: paths toward the comprehensive functional analysis of large gene lists. *Nucleic Acids Res* 2009, 37:1–13.

3. Li G, Ruan X, Auerbach RK, Sandhu KS, Zheng M, Wang P, Poh HM, Goh Y, Lim J, Zhang J, Sim HS, Peh SQ, Mulawadi FH, Ong CT, Orlov YL, Hong S, Zhang Z, Landt S, Raha D, Euskirchen G, Wei C-L, Ge W, Wang H, Davis C, Fisher-Aylor KI, Mortazavi A, Gerstein M, Gingeras T, Wold B, Sun Y, et al.: Extensive promoter-centered chromatin interactions provide a topological basis for transcription regulation. *Cell* 2012, 148:84–98.

4. Cancer Genome Atlas Network: Comprehensive molecular portraits of human breast tumours. *Nature* 2012, 490:61–70.

5. Consortium TEP: An integrated encyclopedia of DNA elements in the human genome. *Nature* 2012, 489:57–74.

6. Frith MC, Fu Y, Yu L, Chen J-F, Hansen U, Weng Z: Detection of functional DNA motifs via statistical over-representation. *Nucleic Acids Res* 2004, 32:1372–1381.

7. Sandelin A, Alkema W, Engström P, Wasserman WW, Lenhard B: JASPAR: an open-access database for eukaryotic transcription factor binding profiles. *Nucleic Acids Res* 2004, 32(Database issue):D91–94.

8. Hnisz D, Abraham BJ, Lee TI, Lau A, Saint-André V, Sigova AA, Hoke HA, Young RA: Super-Enhancers in the Control of Cell Identity and Disease. *Cell* 2013.

9. Cancer Genome Atlas Research Network: Comprehensive molecular profiling of lung adenocarcinoma. *Nature* 2014, 511:543–550.

10. Cancer Genome Atlas Network: Comprehensive molecular characterization of human colon and rectal cancer. *Nature* 2012, 487:330–337.

SUPPLEMENTARY FIGURES

Figure S1. Genome-wide CpG methylation levels of 13 samples determined by whole genome bisulfite shotgun sequencing (WGBS). Displayed are CpG methylation levels at promoters (red), exons (blue), introns (green) and intergenic regions (purple). Samples are organized according to their tissue types.

Figure S2. DNA methylation profiles of super-enhancer regions derived from the displayed normal tissues [8] determined by whole genome bisulfite shotgun sequencing (WGBS). Scaled DNA methylation profile of 1,091 super-enhancers detected in normal breast (**a**) 1,237 in normal lung (**b**), 1,012 in normal colon (**c**), 1,060 in normal brain (**d**) tissue and 675 in normal CD19+ cells (**e**). DNA methylation levels in equally sized windows inside super-enhancers (black bar) and flanking sequences (grey bar) are shown. Each horizontal line represents a single super-enhancer ordered by average DNA methylation levels (0%, green; 100%, red). Super-enhancers are grouped according to their average DNA methylation levels (red, <25%; blue, <50%; green, <75%; purple, <100%).

Figure S3. DNA methylation profiles of super-enhancer regions reveal a specific epigenetic profile. (**a**) Scaled DNA methylation profiles of a random set of 1,091 traditional enhancers (determined by H3K27ac in Human Mammary Epithelial Cells, HMECs [8]) in normal breast tissue. Each enhancer is represented by a single line (blue) and smoothed DNA methylation levels inside the enhancer (black bar) and equally sized flanking sequences (grey bar) are displayed. (**b**) DNA methylation levels of a random set of 1,091 traditional enhancers enhancers (determined by H3K27ac in Human Mammary Epithelial Cells, HMECs [8]) in normal breast tissue in equally sized windows (0%, green; 100%, red). Each horizontal line represents a single enhancer, ordered by average DNA methylation levels. Enhancers are grouped according to their average DNA methylation levels (red, <25%; blue, <50%; green, <75%; purple, <100%). (**c**) Transcription factor (TF) occupancy at scaled 1,091 breast super-enhancers (TF determined in 91 cell lines [5]) in equally sized windows. Each horizontal line represents a single super-enhancer, ordered by previous defined average DNA methylation levels (Figure S1). The proportion of the windows covered by TFs is color-coded (100% TF occupancy, black; no TF binding detected, white). (**d**) Correlation analysis of DNA methylation levels and TF occupancy in scaled 1,091

breast super-enhancer regions and equally sized windows. The density of windows is displayed gradiently (high, red; low, blue).

Figure S4. DNA methylation profiles of super-enhancer regions derived from normal tissues that display tissue-specific DNA methylation patterns. (**a**) DNA methylation levels of super-enhancers determined by WGBS are displayed for related and foreign tissue contexts. Scaled DNA methylation profiles of normal brain, CD19+, breast, colon and lung tissues in equally sized windows inside super-enhancers and flanking sequences are shown. Each horizontal line represents a single super-enhancer ordered by average DNA methylation levels of the specific tissues (0%, green; 100%, red). (**b**) Enrichment analysis of tissue-specific super-enhancers located in promoter regions compared to non-specific super-enhancer locations. Significant enrichments are indicated (*,p<0.001; Fisher's exact test). (**c**) Average DNA methylation differences between outside and inside super-enhancer regions (points) and their corresponding 95% CIs (segments). Each panel corresponds to a set of normal tissue-derived super-enhancers and normal tissue samples are color-coded. (**d**) Hierarchical clustering of DNA methylation levels in tissue-specific super-enhancers (rows) of normal breast (n=20), normal lung (n=26), normal colon (n=18), normal brain (n=9) tissues and sorted CD19+ cells (n=5), analyzed using the Human DNA methylation BeadChip. Average DNA methylation levels of probes (≥5) located in hypomethylated regions within tissue-specific super-enhancers are displayed (0%, white; 100%, blue).

Figure S5. DNA methylation profiles of the brain-specific super-enhancer region associated with *QKI* (**a**) and the CD19+ specific region related to *LYL1* (**b**). Smoothed (colored line) and raw (colored bars) CpG methylation levels are indicated for normal breast (red), normal brain (orange), normal lung (blue), normal colon (green) and sorted CD19+ (purple) cells. Hypomethylated regions (HMRs, colored bars) and super-enhancers (black bar) are indicated. The respective transcription start sites are highlighted (broken lines).

Figure S6. Hypomethylation level at the H3K27ac peak was independent of CpG density. CpG density of super-enhancers (x-axis) and the associated H3K27ac peak signal (y-axis) in 50-bp windows. Super-enhancer windows are split into high (>66%, red) and low (<33%, blue) DNA methylation levels. The significant differences of peak signals between high and low DNA methylation levels in super-enhancer windows were assessed with a multivariate linear model, adjusting for CpG density (p<0.001).

Figure S7. Super-enhancer DNA methylation level correlate with gene expression of the nearest gene. (**a**) Correlation analysis of enhancer HMR coverage levels and gene expression of the closest target gene. Enhancers are split in traditional enhancers (H3K27ac, red) and super-enhancer (blue) and *cis*-acting (no promoter overlap, upper panel) and promoter overlapping (lower panel). Expression data was log transformed and significances of a Spearman's correlation test are indicated. (**b**) Correlation analysis of enhancer HMR coverage levels and gene expression considering the enhancer size. Enhancers are split in short (red, 355-1,512 bp), tall (blue, 1,513-2,931 bp), grande (green, 2,932-58,999 bp) and super-enhancers (purple, 2,932-119,310 bp). Expression data was log transformed and significances of a Spearman's correlation test are indicated. (**c**) Correlation analysis of HMR coverage in *cis*-acting (no promoter overlap) super-enhancer regions and the expression of putative target genes defined by ChIA-PET analysis of polymerase II binding in MCF7 cells [3]. Expression data was log transformed and significances of a Spearman's correlation test are indicated.

Figure S8. Cancer-specific alterations in DNA methylation within super-enhancer regions determined using WGBS. (**a**) Super-enhancers of normal tissue gaining DNA methylation in a cancer context. Difference in DNA methylation levels (coverage of hypomethylated regions, HMRs) between cancer (n=8) and normal (n=5) samples paired within their respective tissue context (y axis). HMR coverage of normal tissues is indicated (x axis) and one plot per normal–tumor pair is displayed. (**b**) DNA methylation levels (coverage of hypomethylated regions, HMRs) of hypomethylated super-enhancers and the indicated cancer samples (δ HMR occupancy >25%) inside (red) and flanking (black) the super-enhancer regions.

Figure S9. Absence of 5-hydroxy CpG methylation (5-hmC) at hypermethylated super-enhancer loci in cancer samples. We compared the methylation values obtained from bisulfite treated cancer samples against oxidative bisulfite treated samples to assess 5-hmC levels and to distinguish between 5-hmC (red) and 5-mC (blue) levels. Significant contribution of the 5-hmC was excluded for CpG sites (450K probes) in hypermethylated super-enhancer regions within their respective cancer samples (Table S5) comparing 5-hmC and 5-mC levels (paired one tailed Wilcoxon test).

Figure S10. Association between DNA methylation levels of hypermethylated super-enhancers and gene expression of target genes. (**a,b**) Differential HMR coverage (x-axis, WGBS) and target gene expression (y-axis) are displayed, comparing normal lung samples and the primary lung adenocarcinoma (H1437, **a**) and lung squamous cell

carcinoma (H157, **b**) cancer cell lines. Significance of the linear regression model is indicated. (**c,d**) Associations of hypermethylated super-enhancers and target gene expression using primary lung adenocarcinoma (**c**) and lung squamous cell carcinoma. (**d**) samples (TCGA). Differential HMR coverage (x-axis, WGBS) and target gene expression (y-axis, RNAseq, scaled log expression) are shown, comparing matched normal lung and primary carcinoma samples. Significances of Spearman's correlation test are indicated. (**e**) Differential HMR coverage (x-axis, WGBS) and target gene expression (y-axis) are displayed, comparing normal brain (white matter) samples and the glioblastoma cell line. Significance of a Spearman's correlation test is indicated.

Figure S11. (**a**) Recurrently affected genes associated with hypermethylated super-enhancers in cancer tissue. Target genes of different samples within tissue types were merged with respect to the origin of the tumor. (**b,c**) Hierarchical clustering of CpG methylation levels (HumanMethylation450 BeadChip) within the super-enhancer regions associated with breast (**b**) and lung (**c**) tissue in 66 primary breast and 321 lung adenocarcinoma samples. CpG methylation levels were clustered using Euclidian distances and the Complete cluster method. DNA methylation levels are color-coded from 0% (white) to 100% (dark blue) and normal (blue) and cancer (red) samples are color-coded. (**d**) Repression of *MIRLET7B* and *MIRLET7A3* in breast and lung cancer cell lines (indicated). Expression levels were determined by quantitative real-time PCR and are displayed relative to the respective normal controls. (**e,f**) Repression of *MIRLET7B* and *MIRLET7A3* in primary in (**e**) breast (TCGA [4]) and (**f**) lung (TCGA [9]) cancer samples. Significances of a Wilcoxon test are indicated.

Figure S12. Focal DNA demethylation events within colorectal super-enhancer regions. Scaled 23 hypomethylated super-enhancers and flanking regions in equally spaced windows. Differential average CpG methylation levels (primary tumor - normal sample) are displayed for the respective windows with colors indicating hypo- (green) and hypermethylation (red).

Figure S13. (**a-c**) Association between transcriptional activity (y-axis) of *MYC* (**a**), *RNF43* (**b**) and *GPRC5A* (**c**) and hypomethylation (x-axis, HumanMethylation450 BeadChip, 450K) of their related super-enhancer in normal (blue, n=12) and colorectal cancer (red, n=258) samples (TCGA [10]). Significance was assessed using a linear regression model applied solely to the cancer samples. (**d**) DNA methylation profiles of the super-enhancer region associated with *GPRC5A* in normal mucosa (red) and the colorectal cancer sample (blue). Smoothed (colored line), raw (grey bars) CpG

methylation levels, hypomethylated regions (colored bars) and the super-enhancer (black bars) are indicated. The enhancer-related histone marks H3K27ac (orange) and H3K4me1 (blue) and the promoter-related mark H3K4me3 (pink) are displayed as ChIP-seq signal intensities (bottom panels) [8]. The transcription start site is indicated (broken line).

Figure S14. *FOXQ1* is overexpressed in primary colorectal tumor samples and correlates with the loss of DNA methylation in super-enhancers. (**a**) Gene expression levels of transcription factors with binding motifs significantly enriched ($p \leq 0.05$) at hypomethylated super-enhancers in the primary colorectal cancer sample (Table S6). Displayed are log fold-change (FC) expression levels comparing matched (black) unmatched (blue) primary colorectal cancer samples (COAD, TCGA). (**b**) Correlation analysis between DNA methylation levels in hypomethylated super-enhancer regions (**Table S5**) and gene expression of enriched transcription factors. Significant associations were assessed using a linear regression model on the cancer samples (red). Slopes and p-values are displayed blow the annotation of the tested transcription factors. Matched DNA methylation was determined by averaging over the DNA methylation array probes (TCGA) falling into the hypomethylated regions of the super-enhancers.

Figure S15. Association of *FOXQ1* expression levels and DNA methylation levels at previously defined hypomethylated super-enhancer regions of *MYC* (**a**), *RNF43* (**b**) and *GPRC5A* (**c**) in colorectal cancer in normal (blue) and colorectal cancer (red) samples (TCGA). Significance was assessed using a linear regression model applied solely to the cancer samples. Matched DNA methylation was determined by averaging over the DNA methylation array probes (TCGA) falling into the hypomethylated regions of the respective super-enhancers.

Figure S16. Association of *FOXQ1* expression levels and expression levels of genes associated with hypomethylated cancer-related super-enhancers in colorectal cancer, specifically, *MYC* (**a**), *RNF43* (**b**) and *GPRC5A* (**c**), in colorectal cancer in normal (blue) and colorectal cancer (red) samples (TCGA). Significance was assessed using a linear regression model applied solely to the cancer samples. Matched DNA methylation was determined by averaging over the DNA methylation array probes (TCGA) falling into the hypomethylated regions of the respective super-enhancers.

Figure S17. Functional validation of the effect of FOXQ1 on their predicted target genes *MYC*, *RNF43* and *GPRC5A*. (**a**) Chromatin immunoprecipitation (IP) experiments for FOXQ1 (flag-tagged) on predicted binding site in the super-enhancer regions related to *MYC*, *RNF43* and *GPRC5A* in HCT116 colorectal cancer cells. Significant enrichments were assigned using a Student's *t*-test (*). Enrichments were assessed comparing IP results of FOXQ1-flag expressing to untransfected cells or to unspecific antibody binding (IgG). (**b**) Small hairpin RNA (shRNA) mediated knockdown of *FOXQ1* led to a reduced expression of the predicted target genes *MYC*, *RNF43* and *GPRC5A* in SW1116 colorectal cancer cells. Significant differences in expression levels were assigned using a Student's *t*-test (*). (**c**) Small hairpin RNA (shRNA) mediated knockdown of *FOXQ1* in SW1116 resulted in a reduced viability of the colorectal cancer cells assessed using an MTT viability assays and analyzed over six consecutive days. Significant differences were assigned using a Student's *t*-test (*).

Figure S18. Validation of the effect of *HNF4A* and *PPARG* on their predicted target genes *RNF43* and *GPRC5A*. Association of TF expression levels and expression levels of genes associated with hypomethylated cancer-related super-enhancers in colorectal cancer, specifically, *RNF43* (**a**) and *GPRC5A* (**b**), in colorectal cancer in normal (blue) and colorectal cancer (red) samples (TCGA). Significance was assessed using a linear regression model applied solely to the cancer samples. Matched DNA methylation was determined by averaging over the DNA methylation array probes (TCGA) falling into the hypomethylated regions of the respective super-enhancers. (**c**) Small hairpin RNA (shRNA) mediated knockdown of *HNF4A* and *PPARG* led to a reduced expression of the predicted target genes *RNF43* and *GPRC5A* in SW1116 colorectal cancer cells. Significant differences were assigned using a Student's *t*-test (*). (**d**) Small hairpin RNA (shRNA) mediated knockdown of *HNF4A* and *PPARG* resulted in a reduced viability of SW1116 colorectal cancer cells assessed using an MTT viability assays and analyzed over six consecutive days. Significant differences were assigned using a Student's *t*-test (*).

Figure S19. Super-enhancer disruption with the BRD4 inhibitor JQ1 does not affect DNA methylation profiles. (**a**) Phenotypic alterations of colorectal cancer cells after JQ1 treatment. HCT116 and SW1116 cells after 48h of treatment with DMSO (vehicle control) or 10µM, 50µM and 100µM of JQ1. Cell viability decreased at 10µM and showed high toxicity at concentration $\geq$50µM. (**b**) Cell viability assay after treatment with crescent concentrations of JQ1 for 48h to determine drug EC50 values (indicated). Each data point corresponds to the mean of four replicates and represents the

percentage of JQ1-treated cells in respect to vehicle treated cells. Error bars ±SD are displayed. Viability curves were generated using a sigmoidal dose-response model with a variable slope. (**c**) DNA methylation levels of CpG sites within normal colon (up) or colorectal (down) super-enhancers comparing JQ1 (y-axis) and vehicle (x-axis) treated cells. Experiments were performed in HCT116 (left) and SW1116 (right) cancer cell lines and DNA methylation was determined using the Infinium HumanMethylation450 BeadChip. Similarity was assessed using a Spearman's correlation test (indicated). (**d**) Expression levels of *MYC*, *RNF43* and *GPRC5A* in HCT116 and SW1116 cells determined by qRT-PCR following 48h treatment with JQ1 or vehicle control (DMSO).

Figure S1

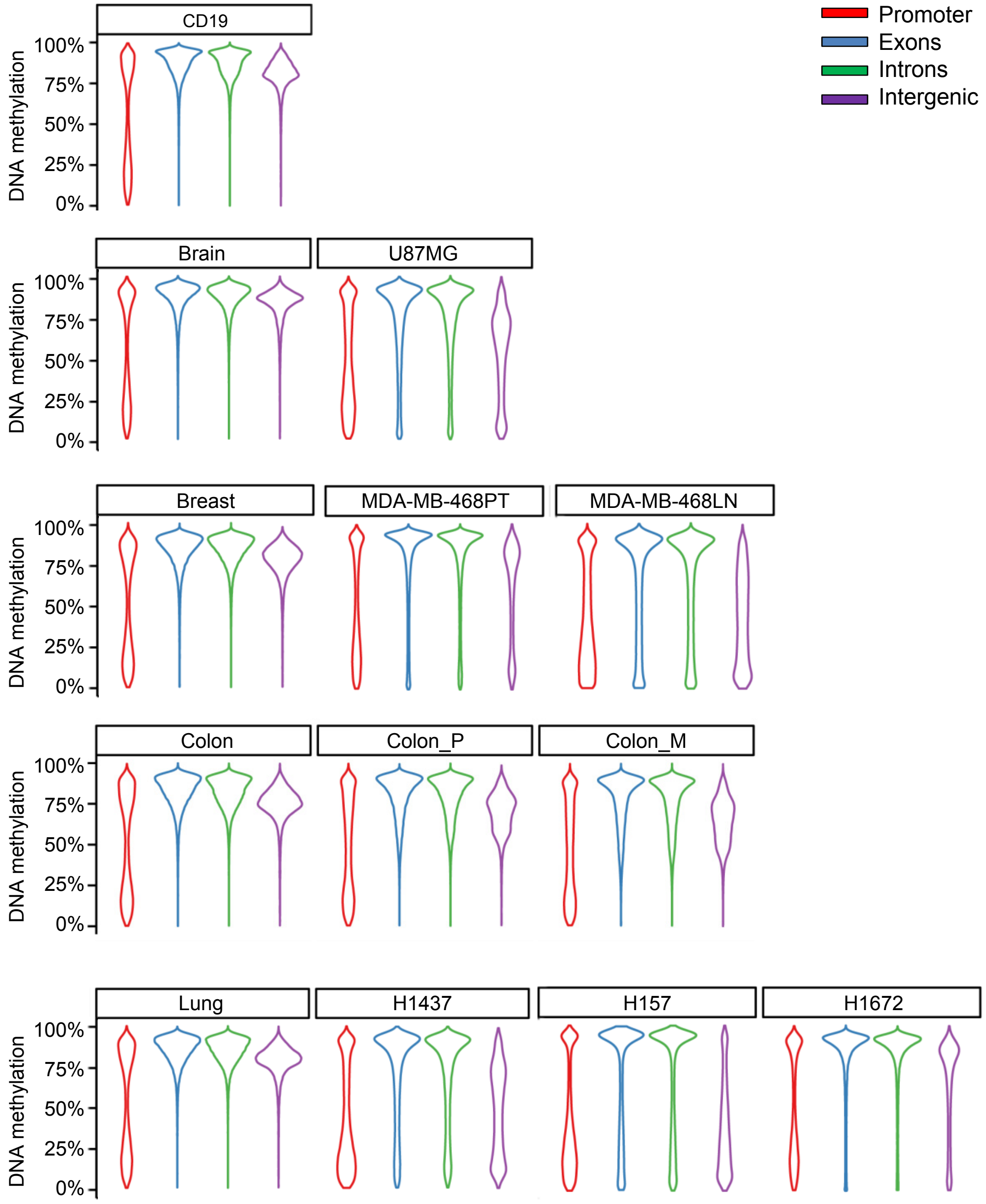

Promoter
Exons
Introns
Intergenic
CD19
Brain
U87MG
Breast
MDA-MB-468PT
MDA-MB-468LN
Colon
Colon_P
Colon_M
Lung
H1437
H157
H1672
DNA methylation
100%
75%
50%
25%
0%

Figure S2

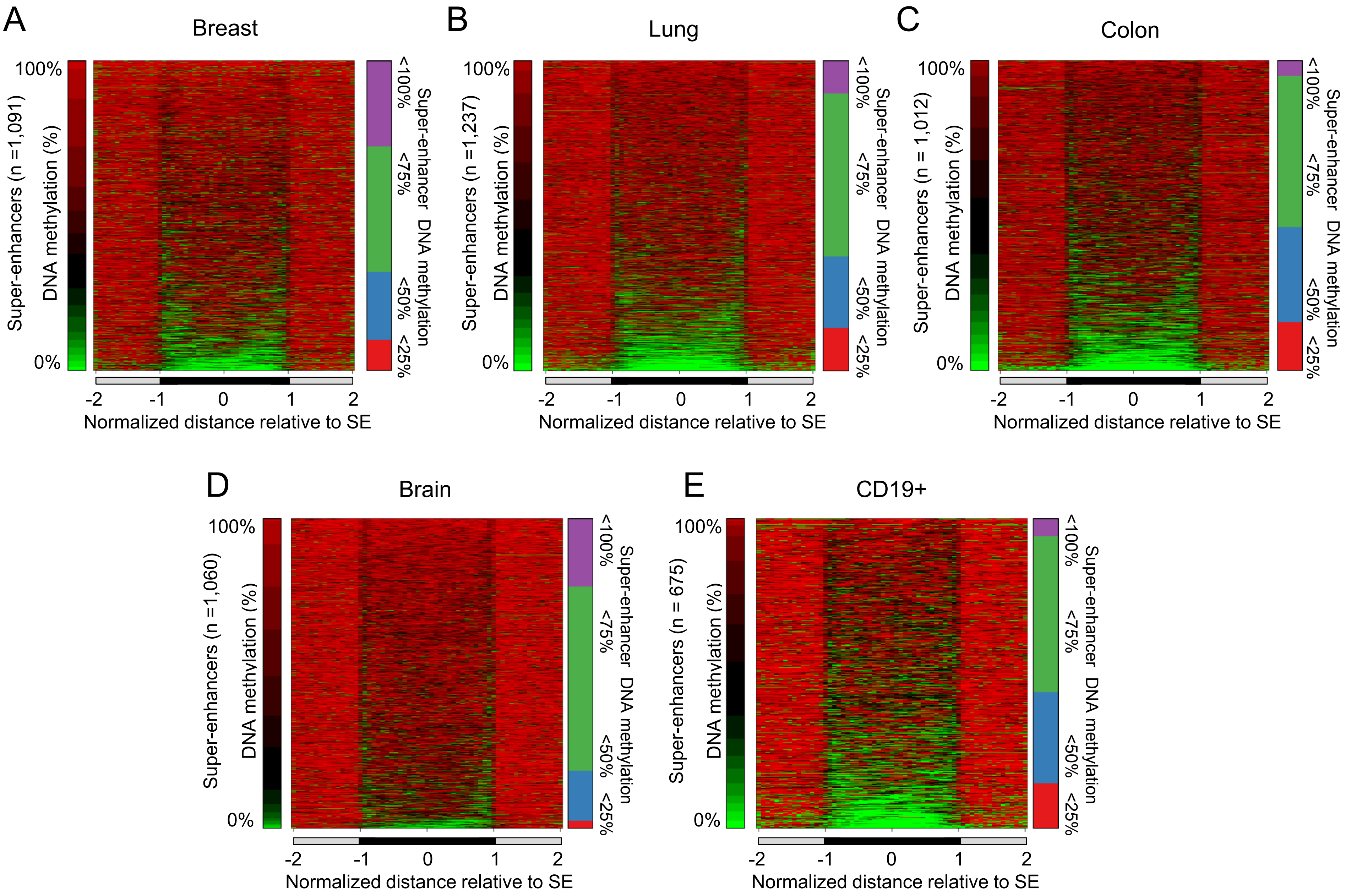
A
Breast
100%
0%
Super-enhancers (n =1,091)
DNA methylation (%)
<100%
<75%
<50%
<25%
Super-enhancer DNA methylation
-2
-1
0
1
2
Normalized distance relative to SE
B
Lung
Super-enhancers (n =1,237)
C
Colon
Super-enhancers (n = 1,012)
D
Brain
Super-enhancers (n =1,060)
E
CD19+
Super-enhancers (n = 675)

Figure S3

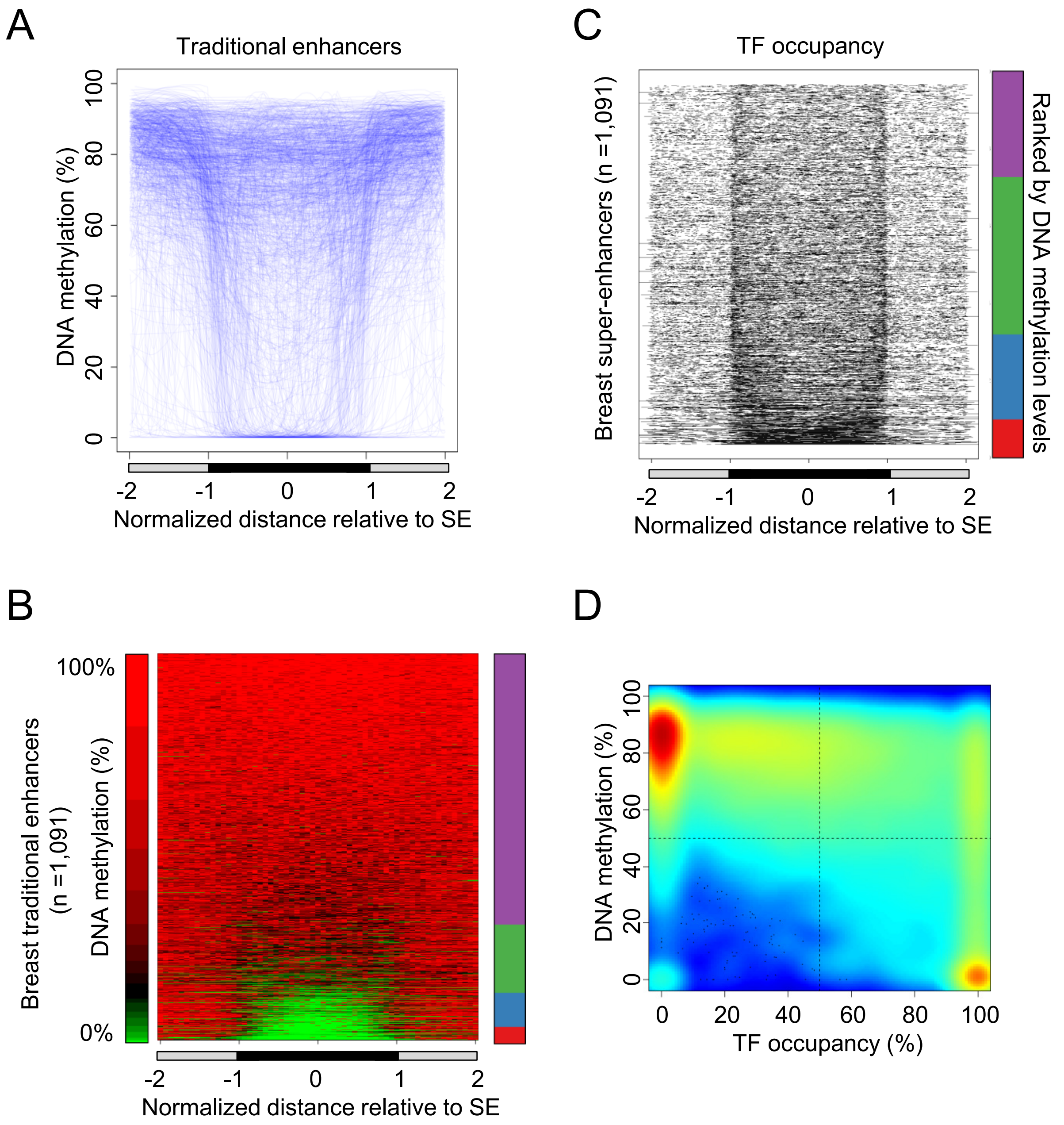
A
Traditional enhancers
DNA methylation (%)
0
20
40
60
80
100
-2
-1
0
1
2
Normalized distance relative to SE
C
TF occupancy
Breast super-enhancers (n =1,091)
Ranked by DNA methylation levels
-2
-1
0
1
2
Normalized distance relative to SE
B
100%
0%
Breast traditional enhancers
(n =1,091)
DNA methylation (%)
-2
-1
0
1
2
Normalized distance relative to SE
D
DNA methylation (%)
0
20
40
60
80
100
TF occupancy (%)

Figure S4

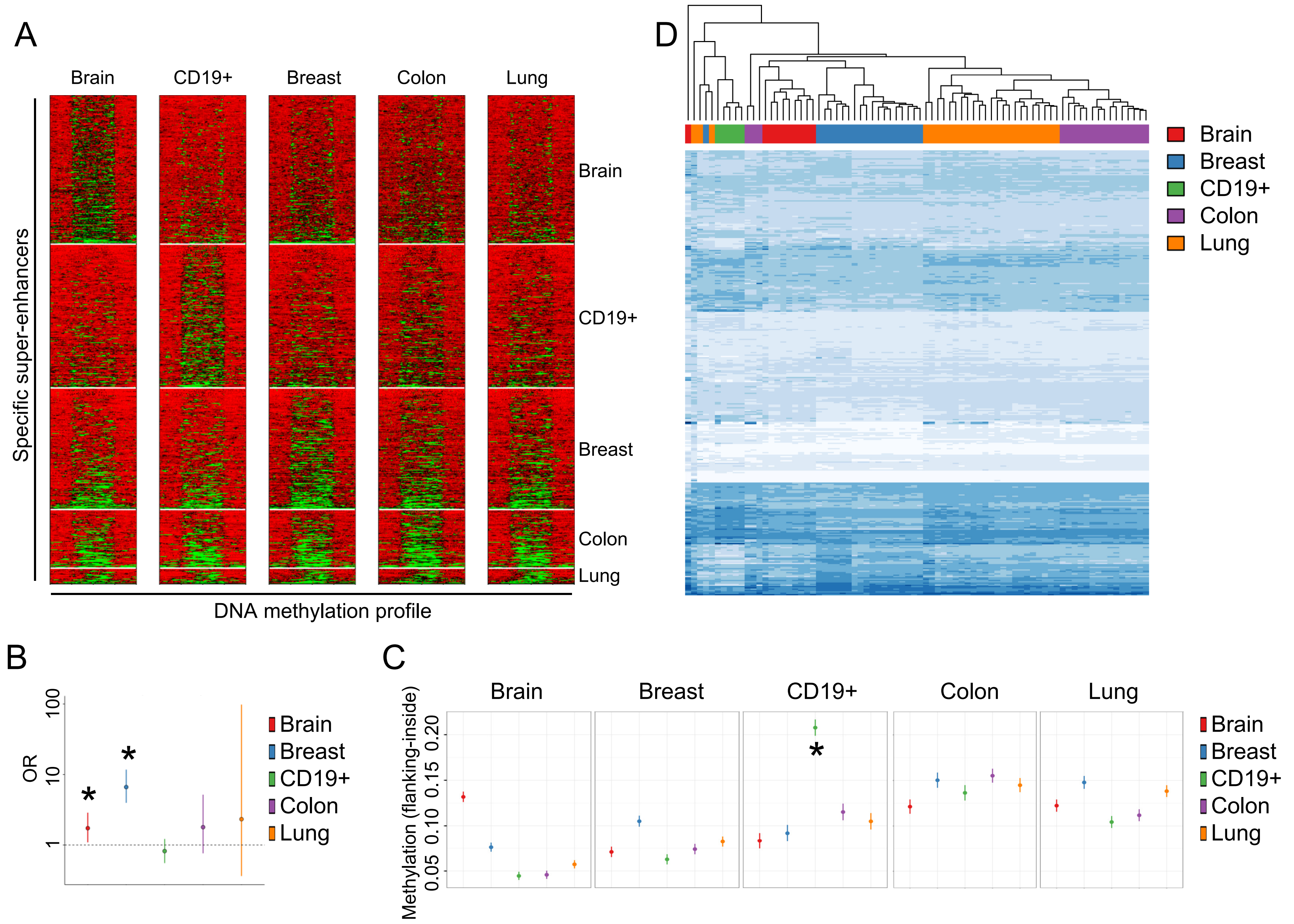

A
Brain
CD19+
Breast
Colon
Lung
Specific super-enhancers
Brain
CD19+
Breast
Colon
Lung
DNA methylation profile
D
Brain
Breast
CD19+
Colon
Lung
B
OR
100
10
1
*
*
Brain
Breast
CD19+
Colon
Lung
C
Brain
Breast
CD19+
Colon
Lung
Methylation (flanking-inside)
0.05
0.10
0.15
0.20
*
Brain
Breast
CD19+
Colon
Lung

Figure S5

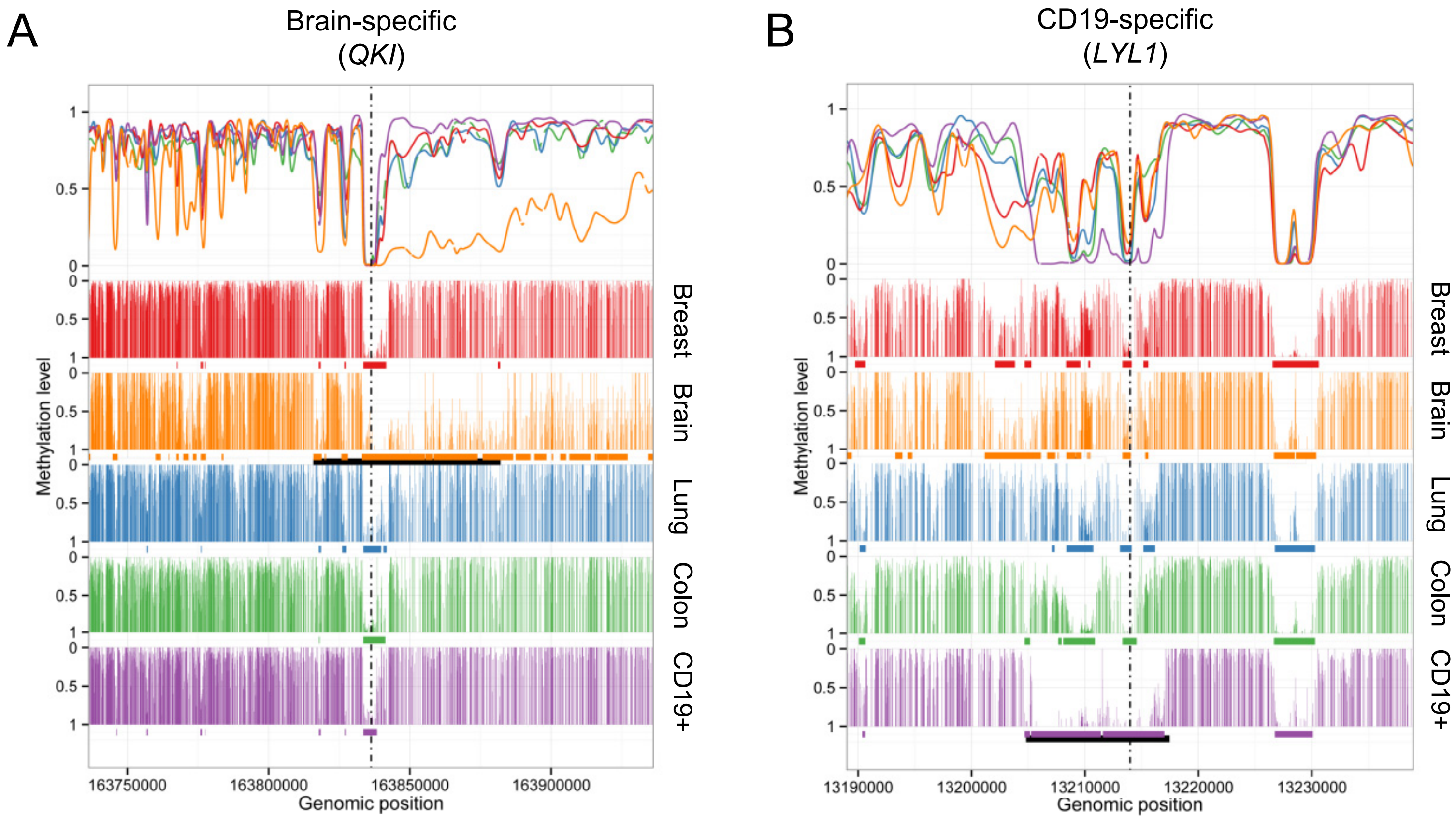
A
Brain-specific
(QKI)
Methylation level
Breast
Brain
Lung
Colon
CD19+
163750000
163800000
163850000
163900000
Genomic position
B
CD19-specific
(LYL1)
13190000
13200000
13210000
13220000
13230000

Figure S6

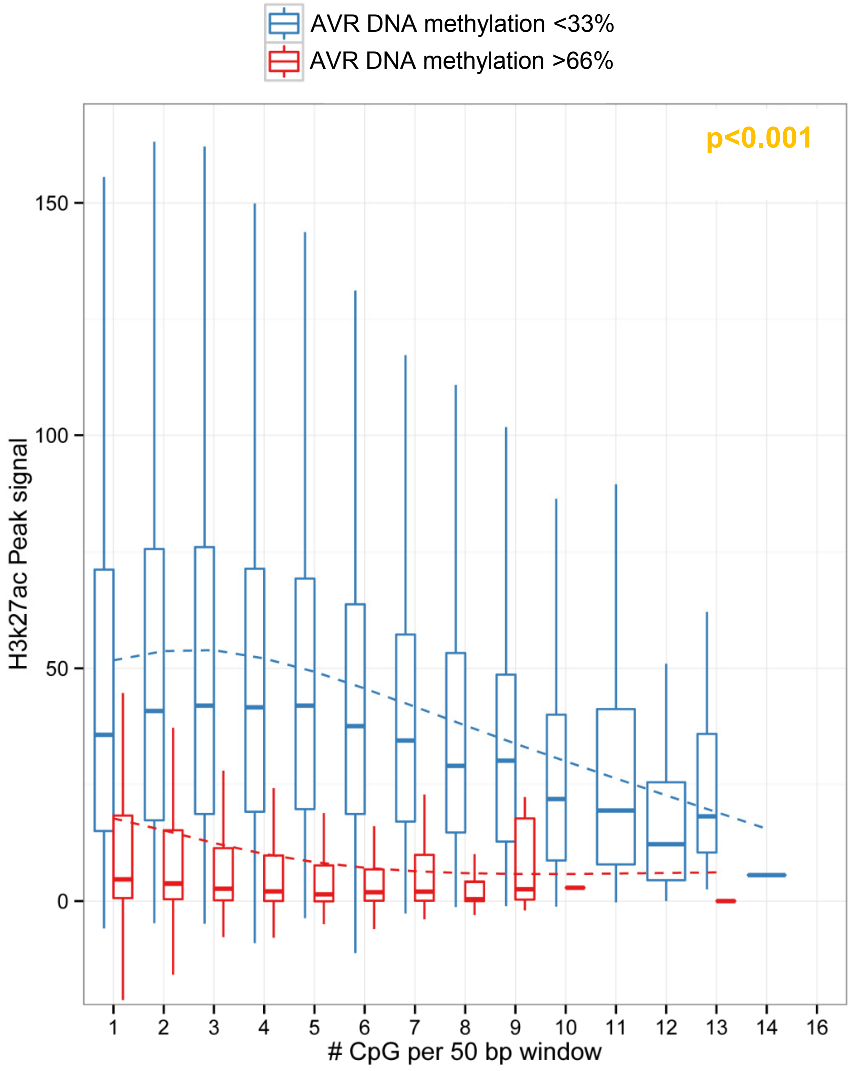

AVR DNA methylation <33%
AVR DNA methylation >66%
p<0.001
H3k27ac Peak signal
150
100
50
0
1
2
3
4
5
6
7
8
9
10
11
12
13
14
16
CpG per 50 bp window

Figure S7

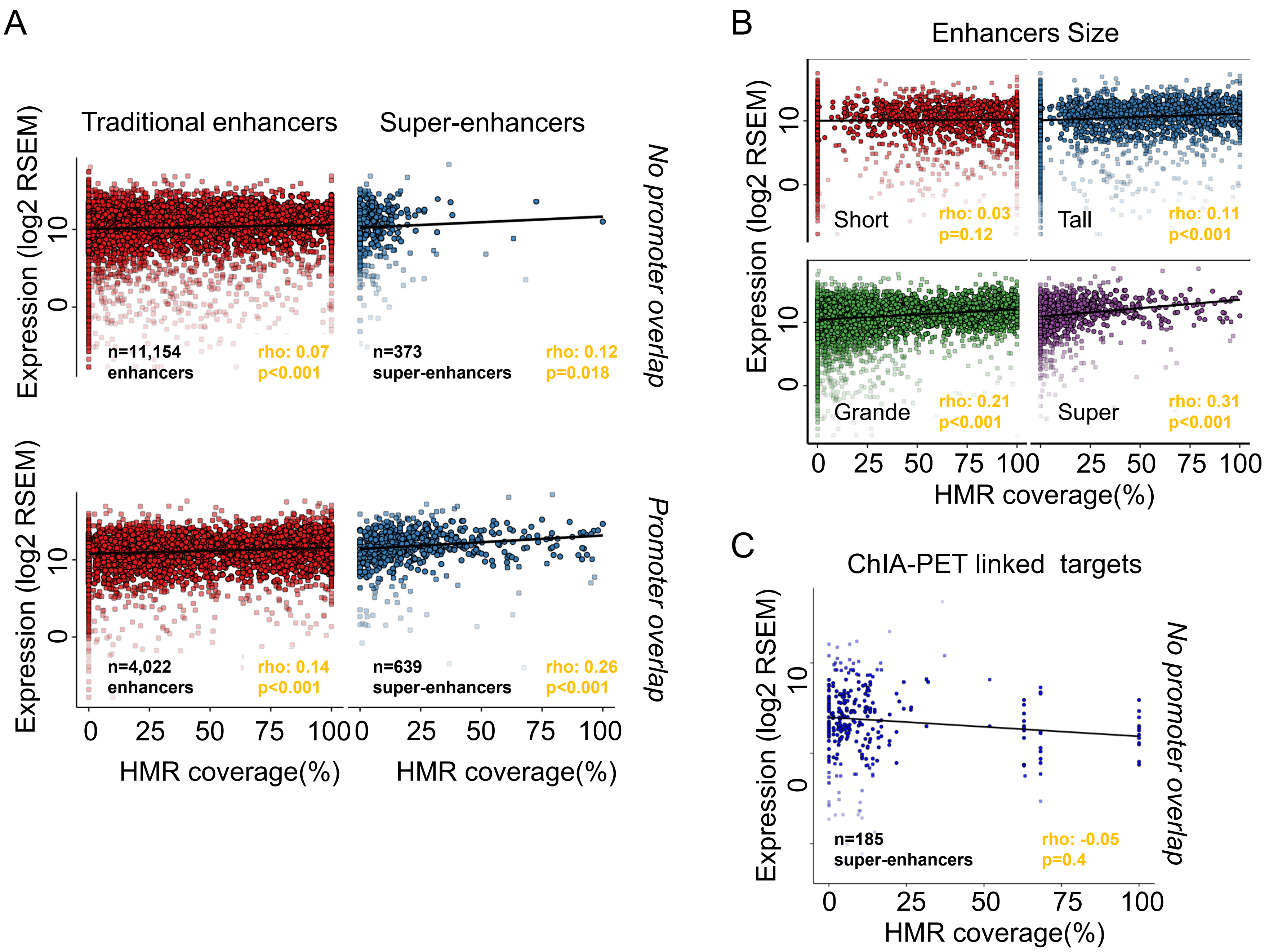

A
Traditional enhancers
Super-enhancers
Expression (log2 RSEM)
n=11,154
enhancers
rho: 0.07
p<0.001
n=373
super-enhancers
rho: 0.12
p=0.018
No promoter overlap
n=4,022
enhancers
rho: 0.14
p<0.001
n=639
super-enhancers
rho: 0.26
p<0.001
Promoter overlap
HMR coverage(%)
HMR coverage(%)
B
Enhancers Size
Short
rho: 0.03
p=0.12
Tall
rho: 0.11
p<0.001
Grande
rho: 0.21
p<0.001
Super
rho: 0.31
p<0.001
HMR coverage(%)
C
ChIA-PET linked targets
n=185
super-enhancers
rho: -0.05
p=0.4
No promoter overlap
HMR coverage(%)

Figure S8

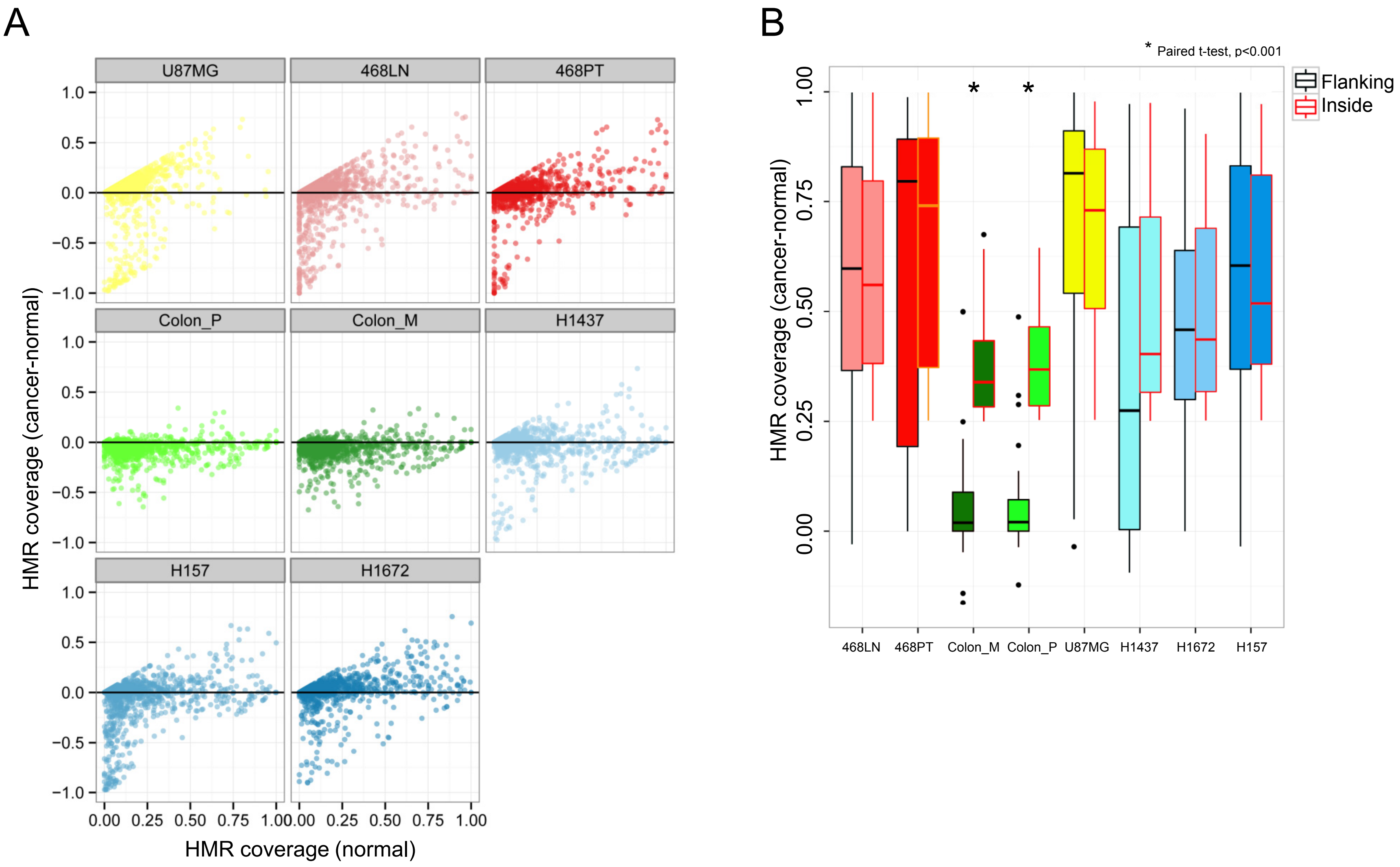
A
U87MG
468LN
468PT
Colon_P
Colon_M
H1437
H157
H1672
HMR coverage (cancer-normal)
HMR coverage (normal)
B
* Paired t-test, p<0.001
Flanking
Inside
468LN
468PT
Colon_M
Colon_P
U87MG
H1437
H1672
H157

Figure S9

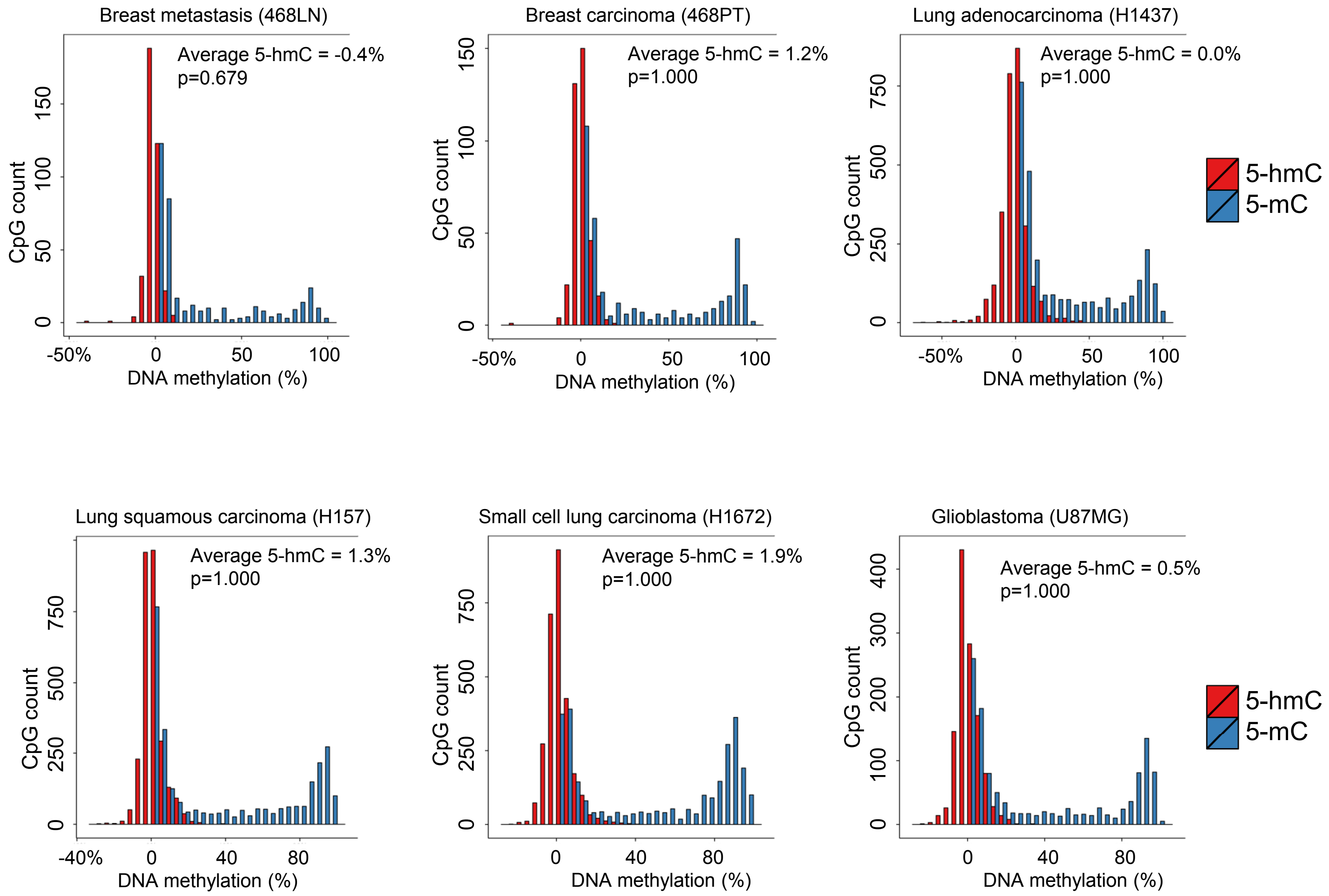
Breast metastasis (468LN)
Average 5-hmC = -0.4%
p=0.679
CpG count
DNA methylation (%)
Breast carcinoma (468PT)
Average 5-hmC = 1.2%
p=1.000
CpG count
DNA methylation (%)
Lung adenocarcinoma (H1437)
Average 5-hmC = 0.0%
p=1.000
CpG count
DNA methylation (%)
5-hmC
5-mC
Lung squamous carcinoma (H157)
Average 5-hmC = 1.3%
p=1.000
CpG count
DNA methylation (%)
Small cell lung carcinoma (H1672)
Average 5-hmC = 1.9%
p=1.000
CpG count
DNA methylation (%)
Glioblastoma (U87MG)
Average 5-hmC = 0.5%
p=1.000
CpG count
DNA methylation (%)
5-hmC
5-mC

Figure S10

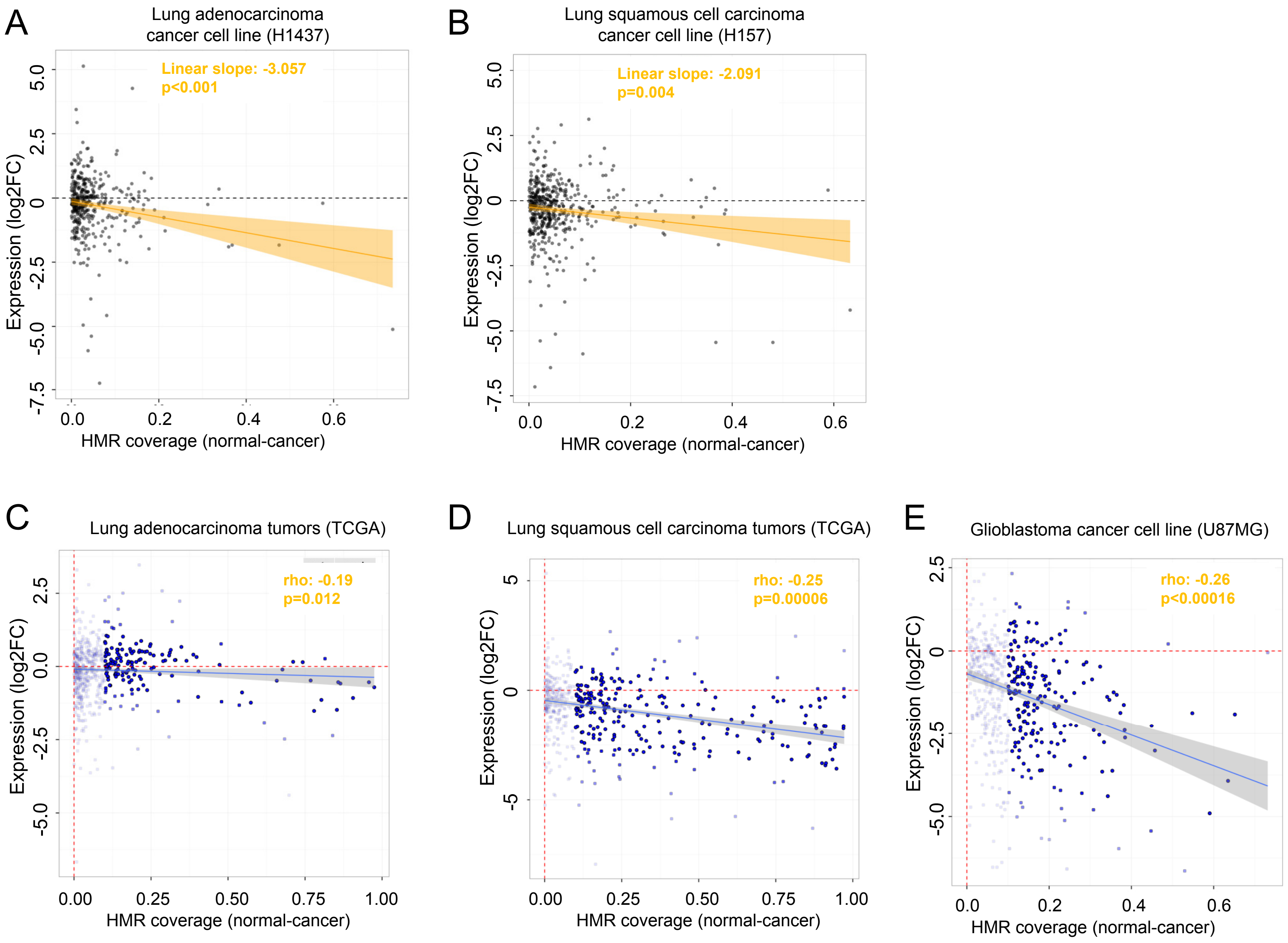
A
Lung adenocarcinoma cancer cell line (H1437)
Linear slope: -3.057
p<0.001
Expression (log2FC)
HMR coverage (normal-cancer)
B
Lung squamous cell carcinoma cancer cell line (H157)
Linear slope: -2.091
p=0.004
C
Lung adenocarcinoma tumors (TCGA)
rho: -0.19
p=0.012
D
Lung squamous cell carcinoma tumors (TCGA)
rho: -0.25
p=0.00006
E
Glioblastoma cancer cell line (U87MG)
rho: -0.26
p<0.00016

Figure S11

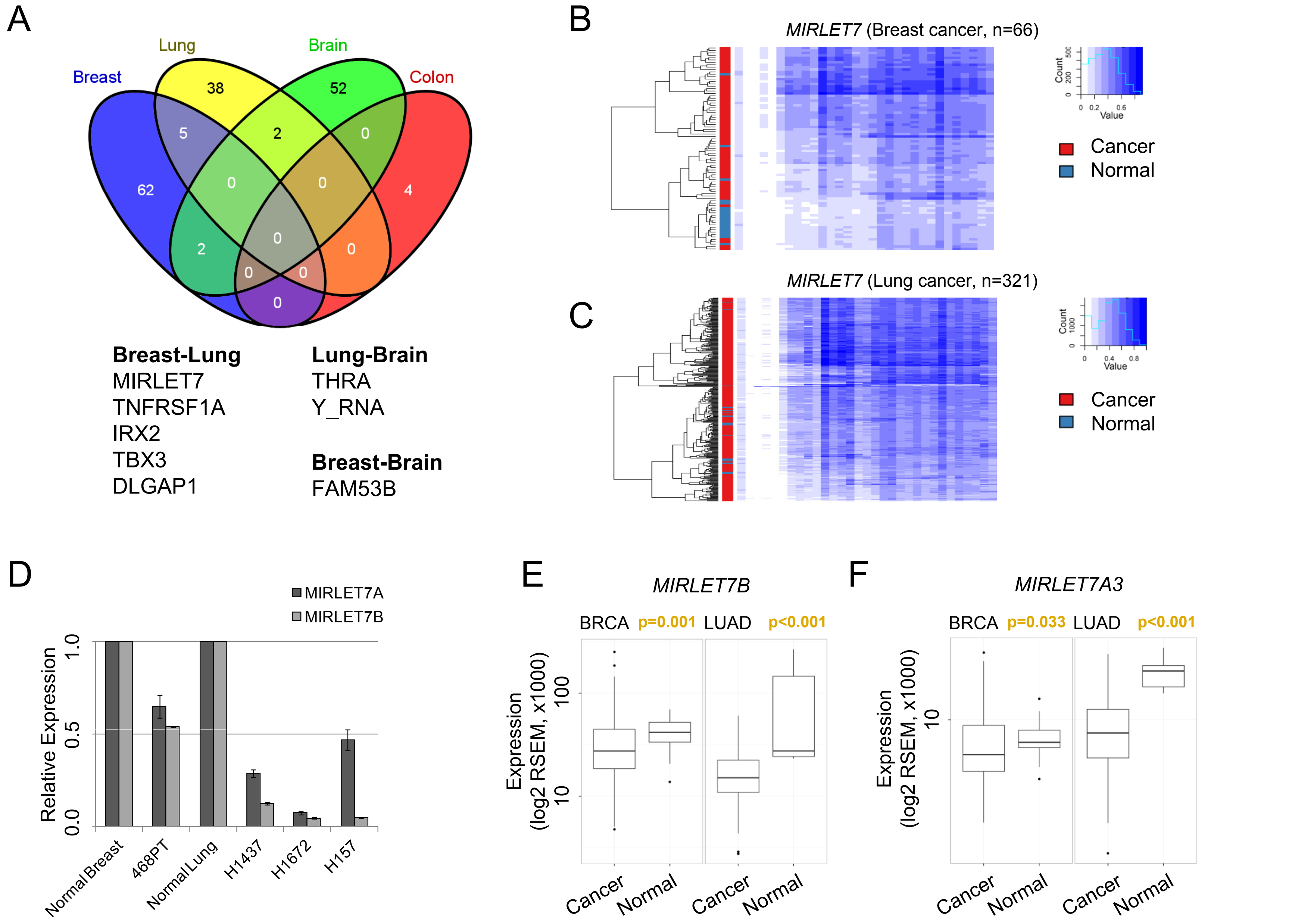

A
Breast
Lung
Brain
Colon
38
52
5
2
0
62
0
0
4
2
0
0
0
0
0
Breast-Lung
MIRLET7
TNFRSF1A
IRX2
TBX3
DLGAP1
Lung-Brain
THRA
Y_RNA
Breast-Brain
FAM53B
B
MIRLET7 (Breast cancer, n=66)
Count
Value
Cancer
Normal
C
MIRLET7 (Lung cancer, n=321)
Count
Value
Cancer
Normal
D
MIRLET7A
MIRLET7B
Relative Expression
1.0
0.5
0.0
Normal Breast
468PT
Normal Lung
H1437
H1672
H157
E
MIRLET7B
BRCA p=0.001
LUAD p<0.001
Expression (log2 RSEM, x1000)
100
10
Cancer
Normal
Cancer
Normal
F
MIRLET7A3
BRCA p=0.033
LUAD p<0.001
Expression (log2 RSEM, x1000)
10
Cancer
Normal
Cancer
Normal

Figure S12

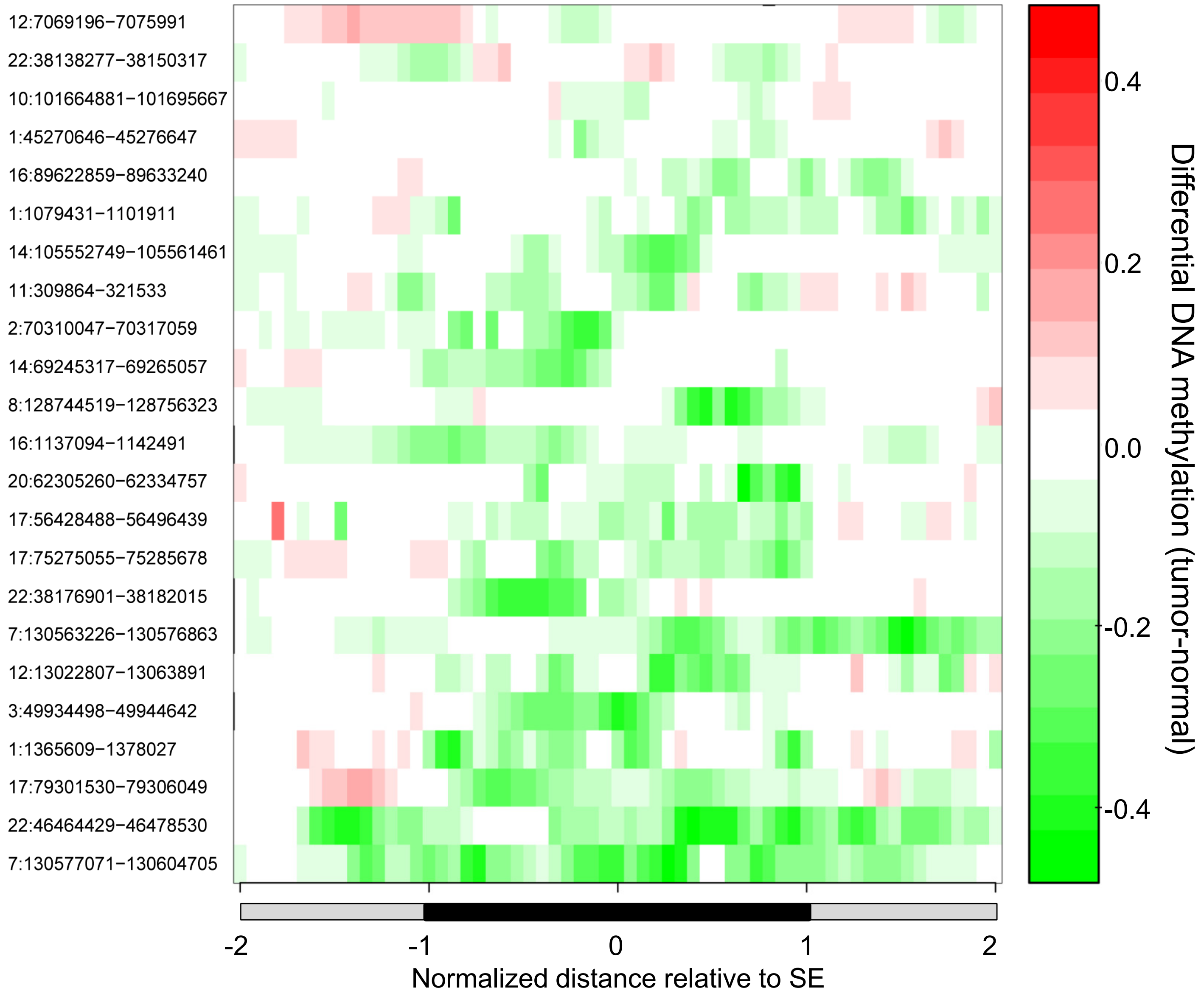

12:7069196−7075991
22:38138277−38150317
10:101664881−101695667
1:45270646−45276647
16:89622859−89633240
1:1079431−1101911
14:105552749−105561461
11:309864−321533
2:70310047−70317059
14:69245317−69265057
8:128744519−128756323
16:1137094−1142491
20:62305260−62334757
17:56428488−56496439
17:75275055−75285678
22:38176901−38182015
7:130563226−130576863
12:13022807−13063891
3:49934498−49944642
1:1365609−1378027
17:79301530−79306049
22:46464429−46478530
7:130577071−130604705
0.4
0.2
0.0
-0.2
-0.4
Differential DNA methylation (tumor-normal)
-2
-1
0
1
2
Normalized distance relative to SE

Figure S13

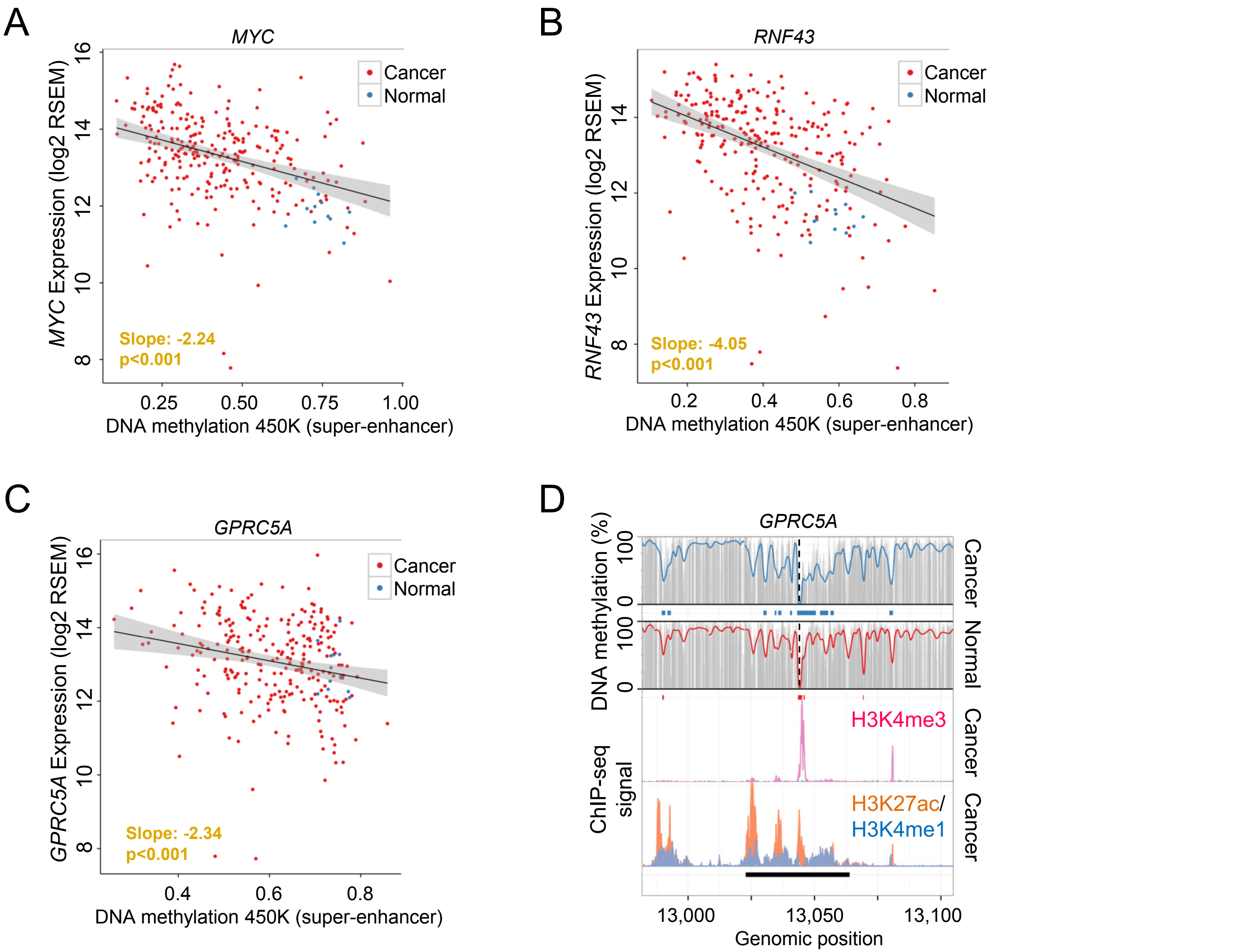
A
MYC
Cancer
Normal
MYC Expression (log2 RSEM)
Slope: -2.24
p<0.001
DNA methylation 450K (super-enhancer)
B
RNF43
Cancer
Normal
RNF43 Expression (log2 RSEM)
Slope: -4.05
p<0.001
DNA methylation 450K (super-enhancer)
C
GPRC5A
Cancer
Normal
GPRC5A Expression (log2 RSEM)
Slope: -2.34
p<0.001
DNA methylation 450K (super-enhancer)
D
GPRC5A
DNA methylation (%)
Cancer
Normal
ChIP-seq signal
H3K4me3
Cancer
H3K27ac/
H3K4me1
Cancer
13,000
13,050
13,100
Genomic position

Figure S14

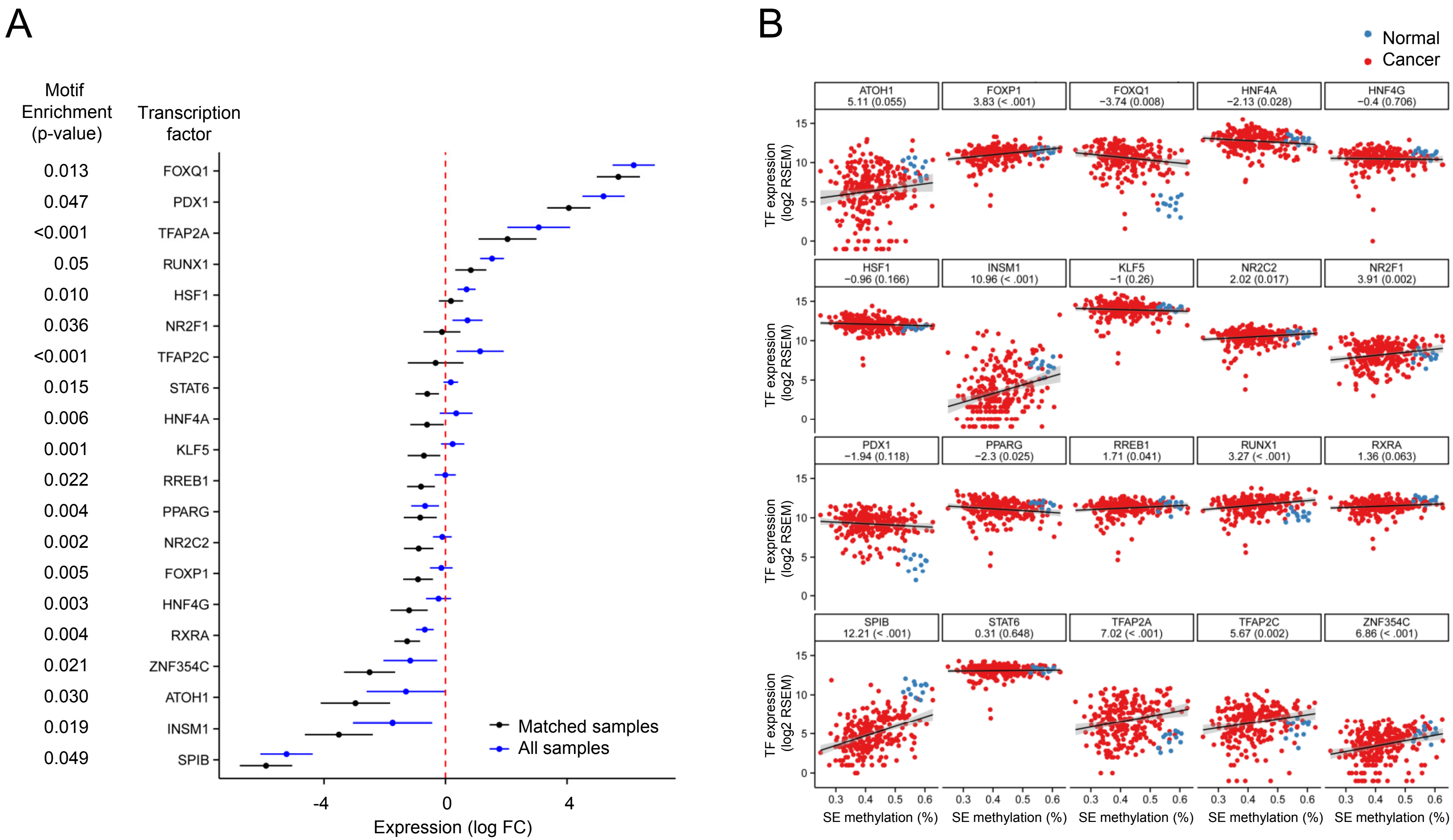

A
Motif Enrichment (p-value)
Transcription factor
0.013 FOXQ1
0.047 PDX1
<0.001 TFAP2A
0.05 RUNX1
0.010 HSF1
0.036 NR2F1
<0.001 TFAP2C
0.015 STAT6
0.006 HNF4A
0.001 KLF5
0.022 RREB1
0.004 PPARG
0.002 NR2C2
0.005 FOXP1
0.003 HNF4G
0.004 RXRA
0.021 ZNF354C
0.030 ATOH1
0.019 INSM1
0.049 SPIB
-4
0
4
Expression (log FC)
Matched samples
All samples
B
Normal
Cancer
ATOH1 5.11 (0.055)
FOXP1 3.83 (< .001)
FOXQ1 −3.74 (0.008)
HNF4A −2.13 (0.028)
HNF4G −0.4 (0.706)
HSF1 −0.96 (0.166)
INSM1 10.96 (< .001)
KLF5 −1 (0.26)
NR2C2 2.02 (0.017)
NR2F1 3.91 (0.002)
PDX1 −1.94 (0.118)
PPARG −2.3 (0.025)
RREB1 1.71 (0.041)
RUNX1 3.27 (< .001)
RXRA 1.36 (0.063)
SPIB 12.21 (< .001)
STAT6 0.31 (0.648)
TFAP2A 7.02 (< .001)
TFAP2C 5.67 (0.002)
ZNF354C 6.86 (< .001)
TF expression (log2 RSEM)
0
5
10
15
0.3 0.4 0.5 0.6
SE methylation (%)

Figure S15

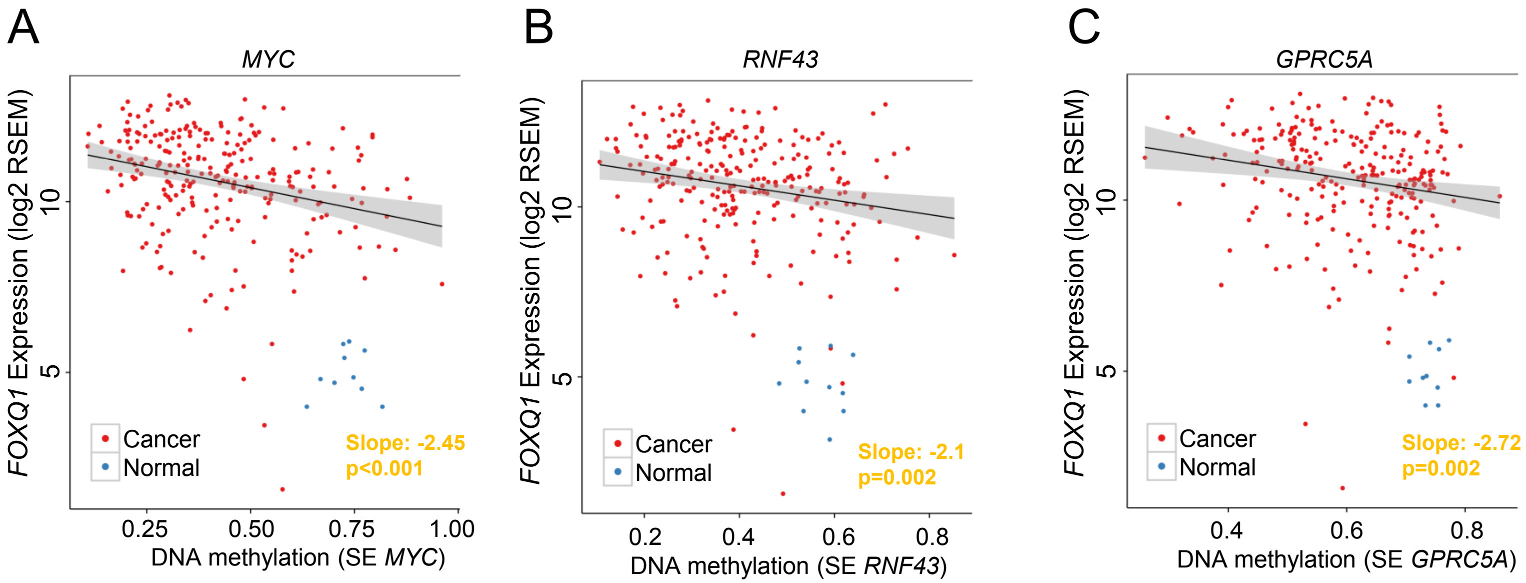
A
MYC
FOXQ1 Expression (log2 RSEM)
10
5
Cancer
Normal
Slope: -2.45
p<0.001
0.25
0.50
0.75
1.00
DNA methylation (SE MYC)
B
RNF43
FOXQ1 Expression (log2 RSEM)
10
5
Cancer
Normal
Slope: -2.1
p=0.002
0.2
0.4
0.6
0.8
DNA methylation (SE RNF43)
C
GPRC5A
FOXQ1 Expression (log2 RSEM)
10
5
Cancer
Normal
Slope: -2.72
p=0.002
0.4
0.6
0.8
DNA methylation (SE GPRC5A)

Figure S16

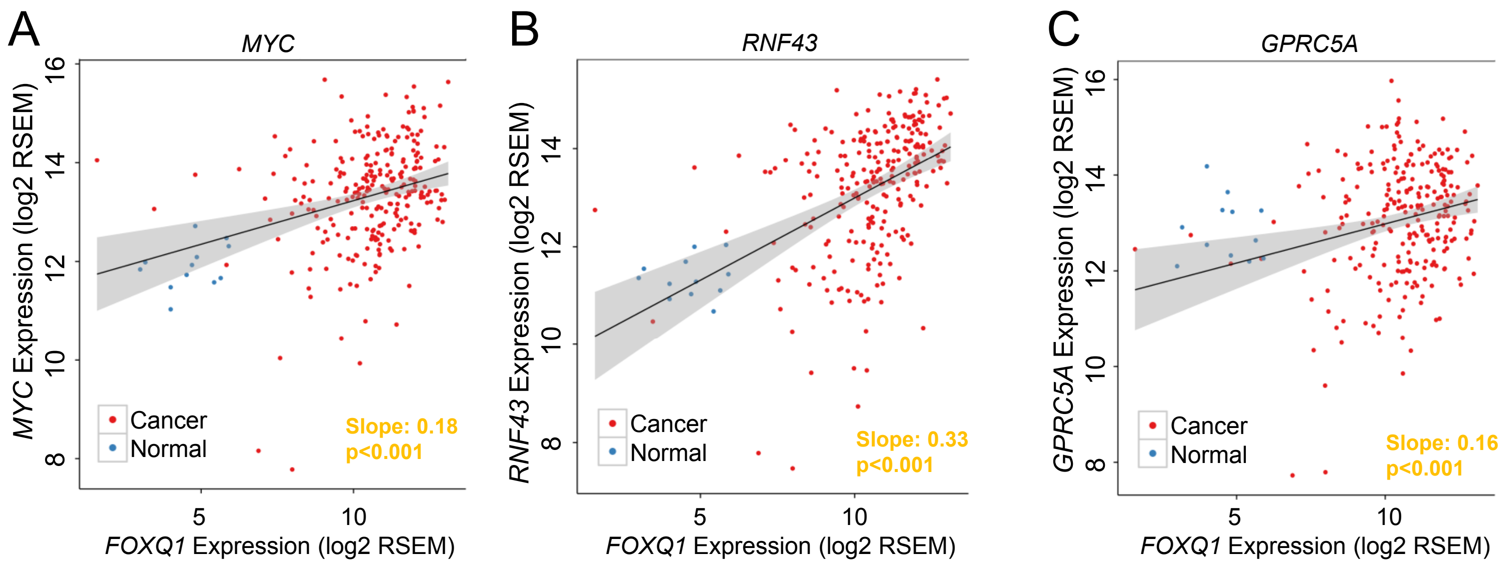
A
MYC
MYC Expression (log2 RSEM)
16
14
12
10
8
5
10
FOXQ1 Expression (log2 RSEM)
Cancer
Normal
Slope: 0.18
p<0.001
B
RNF43
RNF43 Expression (log2 RSEM)
14
12
10
8
5
10
FOXQ1 Expression (log2 RSEM)
Cancer
Normal
Slope: 0.33
p<0.001
C
GPRC5A
GPRC5A Expression (log2 RSEM)
16
14
12
10
8
5
10
FOXQ1 Expression (log2 RSEM)
Cancer
Normal
Slope: 0.16
p<0.001

Figure S17

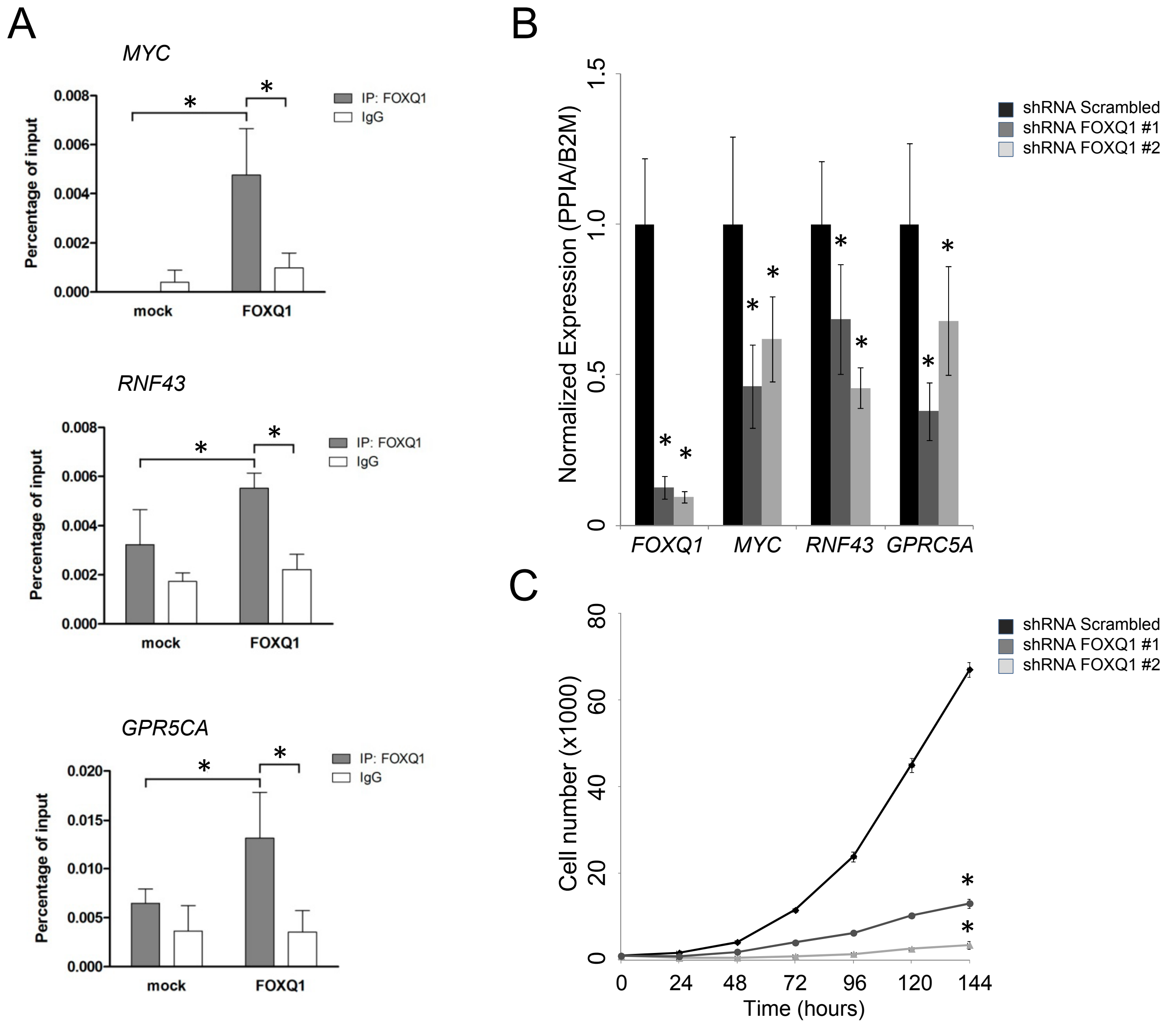
A
MYC
Percentage of input
0.008
0.006
0.004
0.002
0.000
mock
FOXQ1
IP: FOXQ1
IgG
RNF43
GPR5CA
0.020
0.015
0.010
0.005
B
Normalized Expression (PPIA/B2M)
1.5
1.0
0.5
0
FOXQ1
MYC
RNF43
GPRC5A
shRNA Scrambled
shRNA FOXQ1 #1
shRNA FOXQ1 #2
C
Cell number (x1000)
80
60
40
20
0
24
48
72
96
120
144
Time (hours)

Figure S18

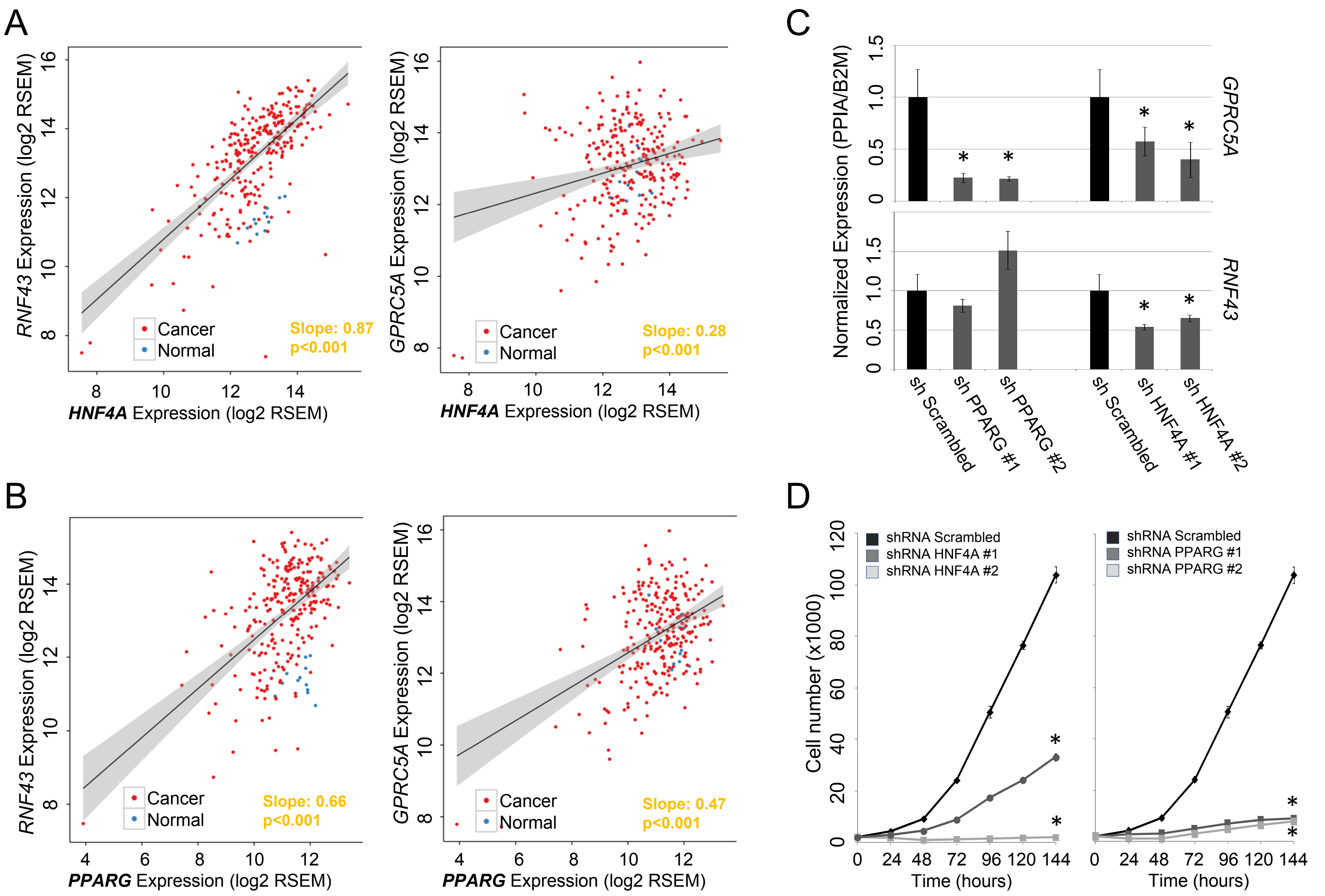

A
RNF43 Expression (log2 RSEM)
HNF4A Expression (log2 RSEM)
Cancer
Normal
Slope: 0.87
p<0.001
GPRC5A Expression (log2 RSEM)
HNF4A Expression (log2 RSEM)
Cancer
Normal
Slope: 0.28
p<0.001
B
RNF43 Expression (log2 RSEM)
PPARG Expression (log2 RSEM)
Cancer
Normal
Slope: 0.66
p<0.001
GPRC5A Expression (log2 RSEM)
PPARG Expression (log2 RSEM)
Cancer
Normal
Slope: 0.47
p<0.001
C
Normalized Expression (PPIA/B2M)
GPRC5A
RNF43
sh Scrambled
sh PPARG #1
sh PPARG #2
sh Scrambled
sh HNF4A #1
sh HNF4A #2
D
Cell number (x1000)
shRNA Scrambled
shRNA HNF4A #1
shRNA HNF4A #2
shRNA Scrambled
shRNA PPARG #1
shRNA PPARG #2
Time (hours)
Time (hours)

Figure S19

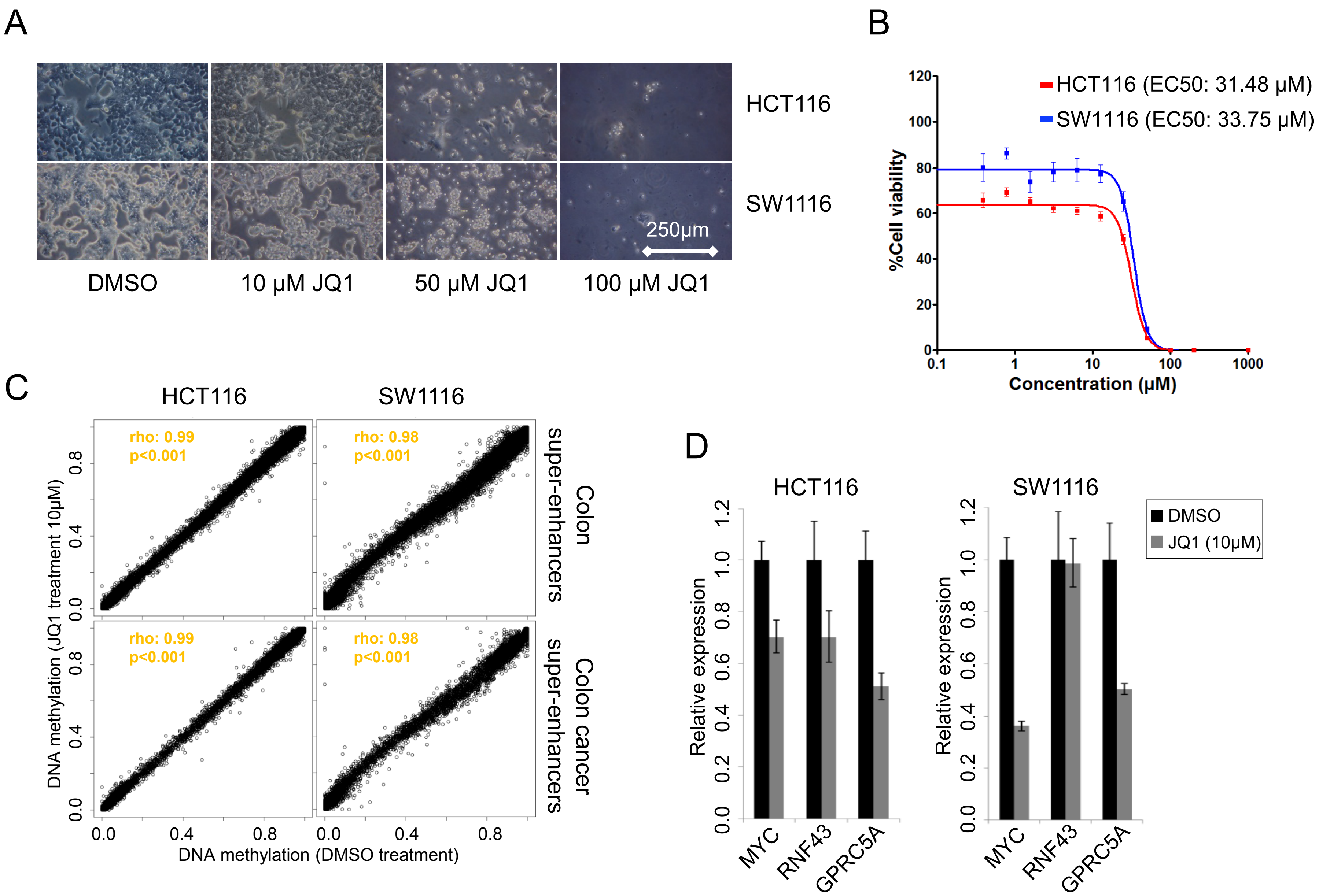
A
HCT116
SW1116
250μm
DMSO
10 μM JQ1
50 μM JQ1
100 μM JQ1
B
HCT116 (EC50: 31.48 μM)
SW1116 (EC50: 33.75 μM)
%Cell viability
Concentration (μM)
C
HCT116
SW1116
rho: 0.99
p<0.001
rho: 0.98
p<0.001
Colon super-enhancers
Colon cancer super-enhancers
DNA methylation (JQ1 treatment 10μM)
DNA methylation (DMSO treatment)
D
HCT116
SW1116
Relative expression
DMSO
JQ1 (10μM)
MYC
RNF43
GPRC5A

Supplementary Table 1: Whole genome bisulfite sequencing of 13 human samples.

	Average DNA methylation (SD)				Super-enhancers covered by WGBS			
	Promoters	Exons	Introns	Intergenic	>5% CpGs	>20% CpGs	>50% CpGs	Total
Colon	51.4 (30.62)	81.7 (11.69)	81.51 (10.5)	74.78 (10.35)	99.8%	99.5%	99.4%	1023
Colon_P	49.82 (29.4)	78.88 (14.38)	78.67 (13.3)	69.32 (12.37)	99.8%	99.5%	99.4%	1023
Colon_M	47.47 (29.07)	75.94 (16.75)	75.61 (15.69)	64.13 (14)	99.8%	99.5%	99.4%	1023
Breast	50.92 (31.57)	82.47 (11.87)	82.92 (10.27)	76.95 (10.75)	100.0%	99.8%	99.5%	1099
468LN	42.3 (29.93)	62.81 (31.32)	62.77 (30.88)	41.86 (26.92)	100.0%	99.8%	99.5%	1099
468PT	49.14 (31.9)	75.33 (26.65)	74.67 (26.78)	59.45 (28.91)	99.8%	99.6%	99.4%	1099
Lung	52.98 (31.36)	84.14 (11.08)	84.13 (9.71)	78.3 (10.19)	99.8%	99.6%	99.1%	1286
H1437	42.08 (30.82)	68.65 (28.49)	67.85 (28.34)	47.52 (26.64)	99.8%	99.5%	99.1%	1286
H157	40.94 (31.33)	65.85 (33.87)	65.69 (33.54)	37.96 (26.85)	99.8%	99.5%	99.2%	1286
H1672	50.36 (32.54)	79.44 (22.5)	79.97 (21.54)	63.67 (27.99)	99.8%	99.6%	99.1%	1286
Brain	54.51 (32.2)	85.58 (12.2)	85.82 (10.44)	82.14 (10.89)	100.0%	99.9%	99.6%	1067
U87MG	47.28 (29.61)	71.43 (25.9)	71.73 (24.88)	52.85 (26.19)	100.0%	99.9%	99.6%	1067
CD19	55.14 (31.8)	86.13 (11.6)	85.86 (10.18)	80.14 (10.38)	100.0%	99.6%	99.0%	688

Supplementary Table 2: Tissue-specific DNA methylation at super-enhancers in normal tissue types.

	Super-Enhancer					HMR coverage (%)		
Tissue	Chr.	Start	End	Transcript ID	Gene	Tissue	Others	Difference
Brain	6	163815822	163882069	ENST00000537883.1	QKI	0.79	0.13	0.66
Brain	7	29516606	29530680	ENST00000410098.1	CHN2	0.63	0.04	0.59
Brain	1	161160323	161173936	ENST00000478866.1	NDUFS2	0.80	0.21	0.59
Brain	13	78291692	78329377	ENST00000358679.2	SLAIN1	0.59	0.00	0.59
Brain	12	110152218	110181906	ENST00000358906.3	FAM222A	0.66	0.08	0.58
Brain	17	79097124	79111085	ENST00000575363.1	AATK	0.59	0.03	0.56
Brain	22	46467856	46491390	ENST00000360737.3	MIRLET7	0.95	0.42	0.54
Brain	18	74684916	74780601	ENST00000583266.1	MBP	0.53	0.04	0.49
Brain	9	131142171	131157912	ENST00000483206.1	URM1	0.49	0.01	0.48
Brain	11	61520065	61534843	ENST00000265460.5	MYRF	0.53	0.06	0.47
Brain	7	124359038	124407316	ENST00000303921.2	GPR37	0.51	0.05	0.46
Brain	18	13610336	13640017	ENST00000435606.1	LDLRAD4	0.53	0.08	0.45
Brain	22	46457577	46466999	ENST00000396008.2	MIRLET7	0.94	0.50	0.44
Brain	22	46457577	46466999	ENST00000333761.1	MIRLET7	0.94	0.50	0.44
Brain	9	130697475	130743184	ENST00000494606.1	FAM102A	0.61	0.17	0.44
Brain	10	14577374	14624047	ENST00000495292.1	FAM107B	0.46	0.02	0.44
Brain	13	67782754	67806323	ENST00000377861.3	PCDH9	0.59	0.20	0.39
Brain	3	33685210	33704614	ENST00000486796.1	CLASP2	0.45	0.06	0.39
Brain	5	36599886	36615091	ENST00000509272.1	SLC1A3	0.50	0.11	0.38
Brain	21	17441409	17461338	ENST00000478932.1	USP25	0.43	0.04	0.38
Brain	8	120648911	120687423	ENST00000427067.2	ENPP2	0.41	0.04	0.37
Brain	7	22212818	22261220	ENST00000420196.1	RAPGEF5	0.37	0.00	0.37
Brain	16	1013186	1042763	ENST00000566034.1	SOX8	0.55	0.19	0.36
Brain	22	38361166	38471689	ENST00000494434.1	PICK1	0.39	0.03	0.36
Brain	9	140081775	140094696	ENST00000477345.1	TPRN	0.56	0.20	0.35
Brain	18	60417206	60470491	ENST00000497351.1	PHLPP1	0.35	0.00	0.35
Brain	8	26427962	26519962	ENST00000474808.1	DPYSL2	0.45	0.10	0.35
Brain	1	198871424	198907549	ENST00000491302.1	PTPRC	0.48	0.13	0.35
Brain	17	36570707	36622668	ENST00000443378.1	ARHGAP23	0.54	0.20	0.34
Brain	3	72424289	72464834	ENST00000477973.1	RYBP	0.34	0.00	0.34
Brain	6	134488737	134505092	ENST00000525700.1	SGK1	0.60	0.27	0.34
Brain	10	81137824	81212605	ENST00000372333.3	ZCCHC24	0.43	0.09	0.33
Brain	4	96452170	96474433	ENST00000504962.1	UNC5C	0.48	0.15	0.33
Brain	16	75266933	75302422	ENST00000393422.2	BCAR1	0.51	0.18	0.33
Brain	3	39487852	39573984	ENST00000479860.1	MOBP	0.34	0.02	0.32
Brain	4	154142334	154219000	ENST00000433687.1	TRIM2	0.38	0.05	0.32
Brain	6	163659272	163687676	ENST00000419182.1	AL078585.1	0.36	0.04	0.32
Brain	10	88421359	88444547	ENST00000542786.1	LDB3	0.33	0.02	0.31
Brain	12	48167031	48182319	ENST00000599515.1	AC004466.1	0.40	0.10	0.31
Brain	2	145129528	145283099	ENST00000475115.1	ZEB2	0.39	0.08	0.30
Brain	4	134065738	134085565	ENST00000511112.1	PCDH10	0.73	0.42	0.30
Brain	1	160068777	160077945	ENST00000448417.1	IGSF8	0.44	0.14	0.30
Brain	2	127806748	127912517	ENST00000409400.1	BIN1	0.36	0.06	0.30
Brain	3	181403893	181455295	ENST00000325404.1	SOX2	0.84	0.56	0.28
Brain	16	19841311	19898686	ENST00000564449.1	GPRC5B	0.35	0.06	0.28
Brain	6	30844750	30864481	ENST00000514434.1	DDR1	0.45	0.18	0.27
Brain	4	115547254	115584118	ENST00000394511.3	UGT8	0.27	0.00	0.27
Brain	14	77765872	77789479	ENST00000555093.1	GSTZ1	0.39	0.12	0.27
Brain	10	650150	686281	ENST00000441152.2	PRR26	0.31	0.03	0.27
Brain	8	53318133	53342376	ENST00000520716.1	ST18	0.29	0.02	0.27
Brain	2	220142124	220157631	ENST00000460801.1	PTPRN	0.43	0.16	0.27
Brain	3	170135527	170167574	ENST00000488989.1	CLDN11	0.34	0.07	0.27
Brain	17	68152783	68183190	ENST00000243457.3	KCNJ2	0.38	0.11	0.27
Brain	21	48018786	48058971	ENST00000440086.1	PRMT2	0.35	0.09	0.26
Brain	19	13093539	13211813	ENST00000588680.1	NFIX	0.47	0.21	0.26
Brain	14	50976072	51001976	ENST00000441560.2	ATL1	0.36	0.11	0.26
Brain	5	173313778	173354104	ENST00000519467.1	CPEB4	0.34	0.08	0.25
Brain	3	134072004	134096230	ENST00000502491.1	AMOTL2	0.44	0.19	0.25
Brain	22	37940770	37968154	ENST00000434728.1	CDC42EP1	0.50	0.25	0.25
Brain	2	164566271	164594418	ENST00000482917.1	FIGN	0.35	0.11	0.25
Brain	15	64974641	64996542	ENST00000559665.2	OAZ2	0.36	0.11	0.24
Brain	1	65712720	65745163	ENST00000483402.1	DNAJC6	0.34	0.10	0.24

Brain	13	101281681	101326566	ENST00000480433.1	TMTC4	0.26	0.02	0.24
Brain	11	67173218	67188540	ENST00000531388.1	CARNS1	0.45	0.21	0.24
Brain	2	158160059	158191916	ENST00000419116.2	ERMN	0.24	0.00	0.24
Brain	11	111845328	111865086	ENST00000530645.1	DIXDC1	0.33	0.09	0.24
Brain	5	126625320	126648566	ENST00000274473.6	MEGF10	0.30	0.06	0.24
Brain	1	221879013	221917508	ENST00000477026.1	DUSP10	0.32	0.09	0.23
Brain	10	22858862	22973344	ENST00000432610.1	PIP4K2A	0.25	0.02	0.23
Brain	7	28724048	28800494	ENST00000468813.1	CREB5	0.27	0.04	0.23
Brain	2	105022517	105068208	ENST00000361360.2	POU3F3	0.24	0.01	0.23
Brain	22	39853661	39873571	ENST00000418314.1	MGAT3	0.34	0.11	0.23
Brain	9	97831315	97861405	ENST00000468164.1	C9orf3	0.25	0.02	0.23
Brain	1	202090299	202115217	ENST00000272217.2	ARL8A	0.39	0.17	0.23
Brain	16	56277138	56298622	ENST00000563440.1	GNAO1	0.24	0.02	0.22
Brain	5	132105879	132116081	ENST00000492490.1	8-Sep	0.43	0.21	0.22
Brain	13	107142225	107162158	ENST00000245323.4	EFNB2	0.29	0.07	0.22
Brain	2	102312024	102465976	ENST00000421882.1	MAP4K4	0.26	0.04	0.22
Brain	10	73722545	73806874	ENST00000373115.4	CHST3	0.28	0.06	0.22
Brain	4	48622532	48657738	ENST00000302806.5	FRYL	0.22	0.00	0.21
Brain	1	160047565	160068437	ENST00000460351.1	IGSF8	0.35	0.14	0.21
Brain	10	126400866	126432679	ENST00000392754.3	FAM53B	0.40	0.19	0.21
Brain	9	133698900	133750533	ENST00000318560.5	ABL1	0.29	0.08	0.21
Brain	17	40114067	40162277	ENST00000591153.1	DNAJC7	0.27	0.07	0.20
Brain	16	57277838	57336233	ENST00000569059.1	PLLP	0.30	0.10	0.20
Brain	10	64127809	64167185	ENST00000421210.1	ZNF365	0.23	0.03	0.20
Brain	3	133460192	133484110	ENST00000498622.1	TF	0.24	0.04	0.20
Brain	1	109889550	109945440	ENST00000471996.1	SORT1	0.26	0.05	0.20
Brain	14	65146034	65196928	ENST00000556801.1	PLEKHG3	0.31	0.11	0.20
Brain	10	128242317	128261039	ENST00000368674.1	C10orf90	0.24	0.04	0.20
Brain	17	72226877	72248627	ENST00000526858.1	TTYH2	0.23	0.03	0.20
Brain	22	45603349	45638976	ENST00000417906.1	KIAA0930	0.27	0.08	0.20
Brain	1	205461954	205494873	ENST00000505932.1	CDK18	0.26	0.06	0.20
Brain	2	74591442	74620177	ENST00000440727.1	DCTN1	0.27	0.07	0.20
Brain	10	124216441	124276238	ENST00000420892.1	HTRA1	0.27	0.07	0.20
Brain	19	54921528	54950915	ENST00000467939.1	TTYH1	0.38	0.18	0.20
Brain	20	35445944	35496093	ENST00000357779.3	SOGA1	0.30	0.11	0.20
Brain	6	41649397	41702285	ENST00000433032.1	TFEB	0.30	0.10	0.20
Brain	2	95678307	95745847	ENST00000349807.3	MAL	0.27	0.08	0.19
Brain	20	31025912	31074699	ENST00000326071.4	C20orf112	0.35	0.16	0.19
Brain	15	45655307	45688573	ENST00000558118.1	GATM	0.24	0.05	0.19
Brain	4	113802567	113832361	ENST00000503271.1	ANK2	0.21	0.02	0.19
Brain	4	113802567	113832361	ENST00000503423.1	ANK2	0.21	0.02	0.19
Brain	4	113802567	113832361	ENST00000506722.1	ANK2	0.21	0.02	0.19
Brain	1	214156400	214205391	ENST00000261454.4	PROX1	0.32	0.13	0.19
Brain	10	126679464	126714232	ENST00000486955.2	CTBP2	0.23	0.04	0.19
Brain	1	109816196	109827024	ENST00000471740.1	PSRC1	0.36	0.17	0.19
Brain	1	66414430	66466650	ENST00000526666.1	PDE4B	0.20	0.02	0.18
Brain	1	90285126	90311060	ENST00000527156.1	LRRC8D	0.33	0.14	0.18
Brain	11	124785353	124817909	ENST00000528971.1	HEPACAM	0.20	0.02	0.18
Brain	1	230932874	231004412	ENST00000366663.5	C1orf198	0.21	0.03	0.18
Brain	1	204796255	204866707	ENST00000514644.1	NFASC	0.21	0.03	0.18
Brain	7	30174536	30225450	ENST00000455738.1	C7orf41	0.23	0.06	0.18
Brain	17	79345155	79376776	ENST00000307745.7	BAHCC1	0.54	0.37	0.17
Brain	17	73682578	73702695	ENST00000579005.1	SAP30BP	0.22	0.05	0.17
Brain	5	124064380	124097267	ENST00000512940.1	ZNF608	0.41	0.24	0.17
Brain	19	50985631	51019282	ENST00000598657.1	ASPDH	0.28	0.11	0.17
Brain	7	32078425	32118455	ENST00000464881.1	PDE1C	0.23	0.06	0.17
Brain	12	123317747	123395518	ENST00000371248.3	VPS37B	0.30	0.13	0.17
Brain	9	135976307	136024639	ENST00000372062.3	RALGDS	0.33	0.16	0.17
Brain	17	56589712	56620235	ENST00000582976.1	4-Sep	0.36	0.19	0.17
Brain	11	63801514	63883871	ENST00000246841.3	FLRT1	0.21	0.05	0.17
Brain	6	69337682	69367340	ENST00000370598.1	BAI3	0.33	0.16	0.17
Brain	12	26265040	26280746	ENST00000534829.1	SSPN	0.64	0.47	0.17
Brain	20	45187487	45213480	ENST00000464518.1	SLC13A3	0.22	0.05	0.17
Brain	3	11024732	11080079	ENST00000495636.1	SLC6A1	0.23	0.06	0.17
Brain	1	160161970	160191690	ENST00000481831.1	DCAF8	0.25	0.09	0.17
Brain	1	160019557	160042341	ENST00000368089.3	KCNJ10	0.23	0.07	0.16
Brain	18	13548488	13588929	ENST00000592657.1	LDLRAD4	0.21	0.05	0.16

Brain	9	8840393	8880342	ENST00000481079.1	PTPRD	0.24	0.08	0.16
Brain	7	141373362	141403139	ENST00000482493.1	KIAA1147	0.22	0.06	0.16
Brain	1	149967151	149985599	ENST00000417191.1	OTUD7B	0.27	0.11	0.16
Brain	4	77099124	77138142	ENST00000512895.1	FAM47E	0.30	0.14	0.16
Brain	22	46445235	46456789	ENST00000381051.2	MIRLET7	0.63	0.47	0.16
Brain	10	699249	735394	ENST00000280886.6	DIP2C	0.21	0.05	0.16
Brain	10	73574974	73621362	ENST00000394934.1	PSAP	0.21	0.05	0.15
Brain	17	27071068	27095625	ENST00000584059.1	FAM222B	0.29	0.14	0.15
Brain	11	134254603	134298147	ENST00000531510.1	B3GAT1	0.26	0.11	0.15
Brain	13	97862565	97936838	ENST00000445661.2	MBNL2	0.24	0.09	0.15
Brain	14	90845063	90876551	ENST00000556721.1	CALM1	0.35	0.20	0.15
Brain	2	193010308	193063844	ENST00000409056.3	TMEFF2	0.21	0.06	0.15
Brain	3	156845617	156856684	ENST00000467849.1	CCNL1	0.30	0.15	0.15
Brain	1	155828272	155864174	ENST00000539162.1	SYT11	0.21	0.06	0.15
Brain	7	45111554	45132881	ENST00000490531.2	NACAD	0.22	0.07	0.15
Brain	6	166689003	166721330	ENST00000322583.3	PRR18	0.20	0.05	0.15
Brain	1	205626881	205650899	ENST00000367145.3	SLC45A3	0.29	0.14	0.15
Brain	9	14299740	14327190	ENST00000493697.1	NFIB	0.57	0.42	0.15
Brain	17	38216903	38234316	ENST00000577486.1	THRA	0.63	0.48	0.15
Brain	9	124022263	124092639	ENST00000477553.1	GSN	0.26	0.12	0.15
Brain	13	114786201	114844487	ENST00000389544.4	RASA3	0.21	0.06	0.15
Brain	11	6400761	6441884	ENST00000533407.1	APBB1	0.21	0.07	0.15
Brain	1	205195003	205257756	ENST00000481950.1	TMCC2	0.23	0.09	0.14
Brain	1	33789227	33859751	ENST00000468406.1	PHC2	0.25	0.11	0.14
Brain	6	52225169	52265846	ENST00000360726.3	PAQR8	0.21	0.07	0.14
Brain	5	115856316	115912087	ENST00000512156.1	SEMA6A	0.23	0.08	0.14
Brain	17	17638844	17657613	ENST00000395774.1	RAI1	0.30	0.16	0.14
Brain	5	139012918	139146609	ENST00000505812.1	CXXC5	0.36	0.22	0.14
Brain	16	15234031	15244187	ENST00000448014.2	NPIPP1	0.27	0.13	0.14
Brain	16	15234031	15244187	ENST00000358815.3	NPIPP1	0.27	0.13	0.14
Brain	17	76857381	76931557	ENST00000262768.7	TIMP2	0.23	0.09	0.14
Brain	17	57405868	57485233	ENST00000582813.1	YPEL2	0.20	0.06	0.14
Brain	19	51033029	51070726	ENST00000599957.1	LRRC4B	0.30	0.17	0.14
Brain	17	42161429	42202224	ENST00000587135.1	HDAC5	0.23	0.10	0.14
Brain	1	110071934	110092553	ENST00000369851.4	GNAI3	0.31	0.18	0.13
Brain	12	49579740	49630368	ENST00000552125.1	TUBA1C	0.23	0.09	0.13
Brain	1	61541625	61624014	ENST00000496712.1	NFIA	0.24	0.11	0.13
Brain	14	51524492	51564122	ENST00000360392.4	TRIM9	0.20	0.07	0.13
Brain	3	123128250	123170404	ENST00000462833.1	ADCY5	0.20	0.08	0.13
Brain	11	118463235	118534958	ENST00000528823.1	PHLDB1	0.23	0.10	0.13
Brain	10	102752997	102775915	ENST00000454422.1	LZTS2	0.44	0.32	0.13
Brain	11	73357627	73375282	ENST00000544282.1	PLEKHB1	0.20	0.08	0.13
Brain	7	86272604	86301105	ENST00000421579.1	GRM3	0.22	0.09	0.13
Brain	1	32792421	32824727	ENST00000574315.1	TSSK3	0.27	0.14	0.12
Brain	15	37371561	37406086	ENST00000559129.1	MEIS2	0.46	0.33	0.12
Brain	15	43800300	43824728	ENST00000300231.5	MAP1A	0.28	0.16	0.12
Brain	5	171829492	171881486	ENST00000519643.1	SH3PXD2B	0.21	0.09	0.12
Brain	11	119211319	119250680	ENST00000527843.1	USP2	0.22	0.10	0.12
Brain	5	646895	697805	ENST00000360578.5	TPPP	0.23	0.12	0.12
Brain	2	232526039	232554844	ENST00000466801.1	PTMA	0.34	0.23	0.12
Brain	10	72971329	73044360	ENST00000373192.4	UNC5B	0.21	0.09	0.12
Brain	10	24989499	25014855	ENST00000463892.1	ARHGAP21	0.33	0.22	0.12
Brain	2	9768796	9795405	ENST00000460093.1	YWHAQ	0.21	0.09	0.11
Brain	16	1064221	1098971	ENST00000397547.2	SSTR5	0.24	0.13	0.11
Brain	19	17858364	17882158	ENST00000600393.1	FCHO1	0.22	0.11	0.11
Brain	12	6639174	6665533	ENST00000396830.2	IFFO1	0.40	0.29	0.11
Brain	10	111967550	111992938	ENST00000369612.1	MXI1	0.40	0.29	0.11
Brain	17	48914258	48946511	ENST00000509385.1	TOB1	0.28	0.17	0.11
Brain	17	38258962	38270871	ENST00000246672.3	NR1D1	0.42	0.31	0.11
Brain	9	139405020	139447486	ENST00000277541.6	NOTCH1	0.32	0.21	0.11
Brain	6	15245470	15269892	ENST00000397311.3	JARID2	0.36	0.25	0.11
Brain	12	1752842	1776949	ENST00000545747.1	WNT5B	0.24	0.13	0.11
Brain	6	128798636	128843173	ENST00000368202.4	PTPRK	0.20	0.09	0.11
Brain	10	88108134	88126246	ENST00000327946.7	GRID1	0.29	0.19	0.11
Brain	1	156353833	156405678	ENST00000495000.1	C1orf61	0.29	0.18	0.11
Brain	21	34399873	34453980	ENST00000498799.1	OLIG1	0.28	0.18	0.11
Brain	20	45922895	45992027	ENST00000441977.1	ZMYND8	0.24	0.13	0.11

Brain	21	43639119	43683392	ENST00000340588.4	ABCG1	0.22	0.11	0.11
Brain	17	76703832	76732967	ENST00000590300.1	CYTH1	0.24	0.14	0.10
Brain	3	69130585	69168856	ENST00000485444.1	ARL6IP5	0.22	0.12	0.10
Brain	19	30713867	30786928	ENST00000591488.1	ZNF536	0.21	0.11	0.10
Brain	12	48125991	48140168	ENST00000395360.2	RAPGEF3	0.20	0.10	0.10
Breast	22	46467114	46488203	ENST00000360737.3	MIRLET7	1.00	0.47	0.53
Breast	5	131593100	131603174	ENST00000418373.1	PDLIM4	0.60	0.08	0.51
Breast	11	8828385	8837469	ENST00000531237.1	ST5	0.63	0.12	0.51
Breast	22	46448058	46467049	ENST00000381051.2	MIRLET7	1.00	0.50	0.50
Breast	9	33157786	33167403	ENST00000379731.4	B4GALT1	0.92	0.42	0.50
Breast	12	10868156	10877012	ENST00000540747.1	CSDA	0.79	0.29	0.50
Breast	1	157964217	157991843	ENST00000416935.2	KIRREL	0.50	0.04	0.47
Breast	12	6479020	6487672	ENST00000539925.1	LTBR	0.57	0.12	0.46
Breast	3	29321550	29334286	ENST00000456853.1	RBMS3	0.65	0.21	0.45
Breast	5	142773722	142785691	ENST00000502500.1	NR3C1	0.95	0.51	0.44
Breast	1	153579895	153590703	ENST00000469571.1	S100A14	0.49	0.05	0.44
Breast	15	42562799	42569003	ENST00000567421.1	GANC	0.91	0.51	0.40
Breast	4	146653244	146658422	ENST00000510096.1	C4orf51	0.68	0.29	0.39
Breast	1	45270631	45276266	ENST00000482715.1	BTBD19	0.65	0.27	0.38
Breast	1	156065471	156100930	ENST00000392353.3	LMNA	0.56	0.18	0.38
Breast	2	239193425	239200323	ENST00000431832.1	PER2	0.73	0.36	0.37
Breast	2	36579464	36605312	ENST00000473403.1	CRIM1	0.55	0.19	0.35
Breast	18	3622231	3626476	ENST00000486430.1	DLGAP1	0.52	0.17	0.35
Breast	21	36252652	36264673	ENST00000399237.2	RUNX1	0.93	0.58	0.35
Breast	1	33792562	33815439	ENST00000473158.1	PHC2	0.49	0.16	0.33
Breast	10	24737918	24757794	ENST00000376451.2	KIAA1217	0.37	0.04	0.33
Breast	8	26465475	26470176	ENST00000523690.1	DPYSL2	0.73	0.40	0.32
Breast	3	141083866	141089271	ENST00000507657.1	ZBTB38	0.39	0.08	0.32
Breast	1	154941228	154949531	ENST00000473344.1	CKS1B	0.82	0.52	0.31
Breast	7	115993193	115998892	ENST00000465451.1	CAV2	0.37	0.07	0.30
Breast	14	77489392	77513614	ENST00000238647.3	IRF2BPL	0.67	0.37	0.29
Breast	14	69402525	69445588	ENST00000553659.1	ACTN1	0.35	0.06	0.29
Breast	20	51581498	51597746	ENST00000371497.4	TSHZ2	0.45	0.16	0.29
Breast	7	55085115	55095372	ENST00000463948.1	EGFR	0.50	0.21	0.29
Breast	9	14298144	14323961	ENST00000493697.1	NFIB	0.71	0.42	0.28
Breast	1	27830256	27900464	ENST00000487743.2	AHDC1	0.37	0.09	0.28
Breast	8	145008274	145031982	ENST00000527816.1	PLEC	0.62	0.34	0.27
Breast	11	62306073	62328249	ENST00000531324.1	AHNAK	0.61	0.34	0.27
Breast	7	27134577	27144793	ENST00000222718.5	HOXA2	0.95	0.68	0.27
Breast	20	10634437	10656669	ENST00000254958.4	JAG1	0.49	0.22	0.27
Breast	15	93425946	93432559	ENST00000555520.1	CHD2	0.87	0.61	0.26
Breast	11	6339234	6344196	ENST00000530979.1	PRKCDBP	0.61	0.34	0.26
Breast	8	116659885	116682409	ENST00000451156.1	TRPS1	0.42	0.17	0.26
Breast	1	59245181	59252471	ENST00000371222.2	JUN	0.86	0.61	0.25
Breast	12	120661774	120703620	ENST00000547772.1	PXN	0.36	0.10	0.25
Breast	11	114162169	114180527	ENST00000545255.1	NNMT	0.26	0.03	0.24
Breast	10	122913683	122920721	ENST00000478567.1	WDR11	0.38	0.16	0.22
Breast	4	77506454	77513253	ENST00000484236.1	SHROOM3	0.31	0.09	0.22
Breast	12	59300121	59315174	ENST00000548968.1	LRIG3	0.39	0.18	0.21
Breast	1	100109973	100129273	ENST00000263174.4	PALMD	0.24	0.03	0.21
Breast	1	60135366	60141479	ENST00000471169.1	FGGY	0.24	0.03	0.21
Breast	5	121515651	121520476	ENST00000514925.1	ZNF474	0.26	0.06	0.21
Breast	6	74224616	74234296	ENST00000455918.1	EEF1A1	0.79	0.59	0.21
Breast	11	9586185	9600407	ENST00000524612.1	WEE1	0.46	0.26	0.21
Breast	20	43963799	43990114	ENST00000537976.1	SDC4	0.33	0.13	0.21
Breast	19	13123147	13172690	ENST00000358552.3	NFIX	0.50	0.29	0.20
Breast	14	105432978	105441663	ENST00000555122.1	AHNAK2	0.43	0.23	0.20
Breast	17	2294949	2311496	ENST00000571836.2	MNT	0.62	0.42	0.20
Breast	11	57528367	57568939	ENST00000534647.1	CTNND1	0.31	0.11	0.20
Breast	1	144706472	144710218	ENST00000338347.4	NBPF9	0.32	0.12	0.20
Breast	1	144706472	144710218	ENST00000440491.2	NBPF9	0.32	0.12	0.20
Breast	11	65238595	65276583	ENST00000309775.7	AP000769.1	0.63	0.43	0.20
Breast	14	55568260	55575863	ENST00000553493.1	LGALS3	0.32	0.13	0.19
Breast	6	121756401	121769000	ENST00000282561.3	GJA1	0.38	0.18	0.19
Breast	22	30792614	30822843	ENST00000407550.3	MTFP1	0.36	0.17	0.19
Breast	12	6432600	6452223	ENST00000538363.1	TNFRSF1A	0.50	0.30	0.19
Breast	2	112249648	112262948	ENST00000371162.4	AC108463.3	0.27	0.08	0.19

Breast	1	181055984	181089589	ENST00000367577.4	IER5	0.32	0.13	0.19
Breast	16	56638741	56649586	ENST00000567300.1	MT2A	0.46	0.27	0.19
Breast	5	1881509	1890183	ENST00000513692.1	IRX4	0.97	0.78	0.19
Breast	3	187453456	187468930	ENST00000496823.1	BCL6	0.81	0.63	0.19
Breast	1	207190041	207209398	ENST00000461135.2	C1orf116	0.22	0.03	0.19
Breast	6	111906089	111928358	ENST00000528599.1	TRAF3IP2	0.33	0.15	0.18
Breast	22	27868674	27873807	ENST00000497225.1	MN1	0.23	0.04	0.18
Breast	22	36718824	36814131	ENST00000216181.5	MYH9	0.34	0.16	0.18
Breast	19	49375182	49379790	ENST00000600406.1	PPP1R15A	0.78	0.60	0.18
Breast	3	193849125	193860857	ENST00000476918.1	HES1	0.79	0.61	0.18
Breast	11	118779500	118810391	ENST00000534788.1	UPK2	0.63	0.45	0.18
Breast	19	1154329	1184542	ENST00000587655.1	SBNO2	0.28	0.10	0.18
Breast	18	3245538	3270102	ENST00000584539.1	MYL12B	0.43	0.25	0.18
Breast	14	74207241	74227902	ENST00000421708.1	ELMSAN1	0.58	0.41	0.18
Breast	14	23292133	23323062	ENST00000547596.1	MMP14	0.33	0.15	0.17
Breast	10	95184875	95242959	ENST00000488645.1	MYOF	0.21	0.04	0.17
Breast	14	55537362	55546494	ENST00000553976.1	MAPK1IP1L	0.24	0.07	0.17
Breast	20	52443340	52447648	ENST00000422805.1	BCAS1	0.63	0.46	0.17
Breast	17	46122636	46152031	ENST00000581319.1	NFE2L1	0.28	0.11	0.17
Breast	11	66821179	66830989	ENST00000532559.1	RHOD	0.33	0.16	0.17
Breast	20	34326330	34331841	ENST00000493853.1	RBM39	0.91	0.74	0.17
Breast	13	48971911	48988013	ENST00000482024.1	LPAR6	0.24	0.07	0.17
Breast	8	62622640	62634995	ENST00000379449.6	ASPH	0.38	0.21	0.17
Breast	2	12841318	12863362	ENST00000405331.3	TRIB2	0.35	0.19	0.17
Breast	5	67510759	67574308	ENST00000520675.1	PIK3R1	0.27	0.10	0.16
Breast	19	16177568	16192210	ENST00000588483.1	TPM4	0.43	0.26	0.16
Breast	10	112252889	112273435	ENST00000468749.1	DUSP5	0.37	0.21	0.16
Breast	19	47598365	47618174	ENST00000594526.1	SAE1	0.46	0.30	0.16
Breast	20	17537085	17560729	ENST00000449141.2	DSTN	0.27	0.12	0.16
Breast	17	38627225	38717027	ENST00000254051.6	TNS4	0.21	0.05	0.16
Breast	11	65678179	65686589	ENST00000530188.1	C11orf68	0.30	0.14	0.16
Breast	17	80831491	80848486	ENST00000572984.1	TBCD	0.21	0.05	0.16
Breast	1	27320120	27340181	ENST00000289166.5	FAM46B	0.33	0.18	0.16
Breast	1	19247868	19283180	ENST00000416166.1	IFFO2	0.29	0.14	0.15
Breast	10	121410843	121447500	ENST00000450186.1	BAG3	0.21	0.06	0.15
Breast	7	42257859	42278946	ENST00000428534.1	GLI3	0.33	0.17	0.15
Breast	7	869464	876882	ENST00000469755.1	SUN1	0.22	0.07	0.15
Breast	15	50644604	50649357	ENST00000560896.1	GABPB1	0.96	0.80	0.15
Breast	17	7736868	7749068	ENST00000570632.1	KDM6B	0.92	0.77	0.15
Breast	18	46449301	46483832	ENST00000586093.1	SMAD7	0.47	0.32	0.15
Breast	17	75274996	75288850	ENST00000587237.1	9-Sep	0.30	0.15	0.15
Breast	12	109225745	109251401	ENST00000548522.1	SSH1	0.24	0.09	0.15
Breast	12	52608969	52643707	ENST00000544024.1	KRT86	0.21	0.06	0.15
Breast	2	114081148	114089147	ENST00000429538.3	PAX8	0.30	0.16	0.15
Breast	9	124030410	124052489	ENST00000545652.1	GSN	0.40	0.26	0.15
Breast	17	75387047	75426957	ENST00000586521.1	9-Sep	0.22	0.08	0.15
Breast	11	61732568	61749363	ENST00000601917.1	AP003733.1	0.38	0.24	0.14
Breast	7	41734099	41745805	ENST00000442711.1	INHBA	0.40	0.26	0.14
Breast	6	138172161	138196002	ENST00000433680.1	TNFAIP3	0.29	0.14	0.14
Breast	1	16274460	16281222	ENST00000494020.1	ZBTB17	0.21	0.07	0.14
Breast	19	41220087	41229010	ENST00000263370.2	ITPKC	0.69	0.55	0.14
Breast	15	68548590	68582156	ENST00000566739.1	FEM1B	0.25	0.12	0.13
Breast	2	106009478	106028025	ENST00000447958.1	FHL2	0.21	0.08	0.13
Breast	10	74051120	74097380	ENST00000473051.1	DNAJB12	0.27	0.14	0.13
Breast	19	42771661	42788152	ENST00000575839.1	CIC	0.57	0.44	0.13
Breast	6	10398835	10421638	ENST00000486038.1	TFAP2A	0.81	0.68	0.13
Breast	11	57045039	57094324	ENST00000527207.1	TNKS1BP1	0.23	0.09	0.13
Breast	3	71104592	71120542	ENST00000497553.1	FOXP1	0.41	0.28	0.13
Breast	20	19954385	20001261	ENST00000481837.1	NAA20	0.22	0.09	0.13
Breast	19	13948994	13959464	ENST00000591727.1	NANOS3	0.68	0.56	0.13
Breast	16	23152633	23161835	ENST00000219689.7	USP31	0.37	0.24	0.12
Breast	1	16156416	16179578	ENST00000375759.3	SPEN	0.49	0.37	0.12
Breast	4	169752417	169771288	ENST00000507735.1	PALLD	0.28	0.16	0.12
Breast	3	9436165	9466747	ENST00000468208.1	SETD5	0.30	0.18	0.12
Breast	1	207221596	207227576	ENST00000367079.2	PFKFB2	0.61	0.48	0.12
Breast	2	43444985	43456496	ENST00000282388.3	ZFP36L2	1.00	0.88	0.12
Breast	1	33207559	33241755	ENST00000373480.1	KIAA1522	0.22	0.10	0.12

Breast	4	95366945	95386196	ENST00000511767.1	PDLIM5	0.24	0.11	0.12
Breast	7	36426266	36435508	ENST00000418118.1	ANLN	0.36	0.23	0.12
Breast	5	124075983	124085579	ENST00000512940.1	ZNF608	0.90	0.78	0.12
Breast	1	155097310	155110100	ENST00000484027.1	SLC50A1	0.63	0.51	0.12
Breast	1	201404474	201474025	ENST00000532460.1	CSRP1	0.22	0.10	0.12
Breast	18	3446318	3460082	ENST00000472042.1	TGIF1	0.68	0.56	0.12
Breast	6	136605683	136612552	ENST00000528229.1	BCLAF1	0.54	0.42	0.12
Breast	1	6637843	6670728	ENST00000496707.1	KLHL21	0.28	0.17	0.12
Breast	11	65185034	65198304	ENST00000531151.1	FRMD8	1.00	0.88	0.12
Breast	5	140887653	140909039	ENST00000468119.1	DIAPH1	0.23	0.11	0.12
Breast	10	106080462	106112294	ENST00000458723.1	ITPRIP	0.31	0.19	0.12
Breast	4	103736345	103751180	ENST00000503282.1	UBE2D3	0.48	0.36	0.11
Breast	5	2739262	2759651	ENST00000502957.1	IRX2	0.92	0.80	0.11
Breast	15	75930589	75963120	ENST00000569152.1	SNX33	0.27	0.16	0.11
Breast	2	87744763	87759323	ENST00000359481.4	PLGLB2	0.21	0.09	0.11
Breast	10	82213990	82238676	ENST00000372156.1	TSPAN14	0.25	0.14	0.11
Breast	1	144981791	144985538	ENST00000596396.1	AL590452.1	0.28	0.17	0.11
Breast	12	109082915	109097018	ENST00000547170.1	CORO1C	0.31	0.21	0.11
Breast	10	73723394	73770620	ENST00000373115.4	CHST3	0.25	0.15	0.11
Breast	12	125388867	125425378	ENST00000542416.1	UBC	0.39	0.28	0.11
Breast	1	27183345	27192842	ENST00000339276.4	SFN	0.33	0.22	0.11
Breast	17	17723596	17746027	ENST00000435530.2	SREBF1	0.36	0.25	0.11
Breast	14	69241271	69268143	ENST00000408913.2	ZFP36L1	0.42	0.32	0.10
Breast	10	33226394	33252825	ENST00000439974.3	ITGB1	0.30	0.19	0.10
Breast	16	15735275	15740014	ENST00000396353.2	NDE1	0.64	0.54	0.10
Breast	17	17578482	17616584	ENST00000471135.2	RAI1	0.26	0.15	0.10
Breast	11	12694725	12726400	ENST00000334310.6	TEAD1	0.27	0.17	0.10
Breast	1	8062975	8088860	ENST00000487559.1	ERRFI1	0.25	0.14	0.10
Breast	9	130280649	130347137	ENST00000373314.3	FAM129B	0.20	0.10	0.10
Breast	10	74002370	74038384	ENST00000471240.1	DDIT4	0.27	0.17	0.10
Breast	17	27134005	27140821	ENST00000582059.1	FAM222B	0.45	0.35	0.10
Breast	7	115849775	115868787	ENST00000485009.1	TES	0.21	0.11	0.10
Breast	2	46523740	46563334	ENST00000475822.1	EPAS1	0.23	0.13	0.10
CD19	19	13204800	13217457	ENST00000590120.1	LYL1	0.94	0.34	0.60
CD19	14	106313303	106330974	ENST00000482999.1	KIAA0125	0.51	0.00	0.51
CD19	17	73289760	73295202	ENST00000580273.1	SLC25A19	0.51	0.00	0.51
CD19	8	134509931	134514822	ENST00000520020.1	ST3GAL1	0.60	0.12	0.48
CD19	12	57869667	57876031	ENST00000552249.1	ARHGAP9	0.56	0.14	0.42
CD19	2	232255961	232260646	ENST00000287590.5	B3GNT7	0.54	0.13	0.41
CD19	22	50627294	50632183	ENST00000395829.1	TRABD	0.69	0.28	0.41
CD19	2	182168501	182179942	ENST00000476089.1	ITGA4	0.40	0.00	0.40
CD19	6	31547188	31556914	ENST00000464044.1	LST1	0.57	0.18	0.40
CD19	17	43299369	43312992	ENST00000591434.1	FMNL1	0.55	0.16	0.39
CD19	1	26609716	26620076	ENST00000319041.6	SH3BGRL3	0.40	0.03	0.37
CD19	17	47814002	47827686	ENST00000515240.1	FAM117A	0.35	0.00	0.35
CD19	19	16693911	16706114	ENST00000598492.1	MED26	0.52	0.17	0.35
CD19	7	50344148	50370755	ENST00000426121.1	IKZF1	0.41	0.07	0.35
CD19	4	26883290	26890121	ENST00000478049.1	STIM2	0.34	0.00	0.34
CD19	2	197013308	197048452	ENST00000449152.1	STK17B	0.42	0.07	0.34
CD19	21	45660565	45668530	ENST00000400377.3	ICOSLG	0.72	0.38	0.34
CD19	14	64700800	64703816	ENST00000554928.1	SYNE2	0.42	0.08	0.34
CD19	9	71251305	71262079	ENST00000541509.1	PIP5K1B	0.33	0.00	0.33
CD19	9	134593692	134613095	ENST00000372189.3	RAPGEF1	0.40	0.08	0.32
CD19	11	9708104	9716296	ENST00000524817.1	SWAP70	0.39	0.07	0.32
CD19	20	42307887	42312191	ENST00000217026.4	MYBL2	0.32	0.00	0.32
CD19	15	81576278	81610588	ENST00000559953.1	IL16	0.33	0.01	0.32
CD19	11	58340596	58348101	ENST00000389919.4	ZFP91-CNTF	0.83	0.52	0.31
CD19	17	56394652	56423683	ENST00000583624.1	BZRAP1	0.46	0.16	0.31
CD19	8	56751994	56758932	ENST00000519728.1	LYN	0.54	0.24	0.30
CD19	1	111401311	111424334	ENST00000476408.1	CD53	0.29	0.00	0.29
CD19	14	61937696	61947119	ENST00000536400.1	PRKCH	0.33	0.04	0.29
CD19	1	167584400	167603373	ENST00000537350.1	RCSD1	0.37	0.08	0.29
CD19	2	31436778	31445756	ENST00000403897.3	CAPN14	0.28	0.00	0.28
CD19	5	133262873	133277639	ENST00000509913.1	C5orf15	0.28	0.00	0.28
CD19	7	86840957	86850205	ENST00000455575.1	TMEM243	0.59	0.31	0.28
CD19	19	35694706	35707859	ENST00000324675.3	FAM187B	0.34	0.06	0.28
CD19	6	13258837	13278907	ENST00000481706.1	PHACTR1	0.29	0.01	0.28

CD19	18	77219226	77227908	ENST00000591089.1	NFATC1	0.29	0.01	0.28
CD19	19	7400756	7417433	ENST00000576789.1	CTB-133G6.1	0.32	0.05	0.27
CD19	11	117872360	117887673	ENST00000525467.1	IL10RA	0.28	0.01	0.27
CD19	9	112727706	112739040	ENST00000434623.2	AKAP2	0.27	0.00	0.27
CD19	14	105524940	105537562	ENST00000546679.1	GPR132	0.32	0.05	0.27
CD19	8	101505352	101515105	ENST00000519316.1	ANKRD46	0.31	0.04	0.27
CD19	19	49827519	49845306	ENST00000600121.1	CD37	0.38	0.12	0.27
CD19	1	53096945	53108122	ENST00000424164.1	FAM159A	0.35	0.08	0.27
CD19	14	106282350	106287664	ENST00000482999.1	KIAA0125	0.27	0.00	0.27
CD19	15	75066062	75093388	ENST00000569321.1	CSK	0.51	0.25	0.27
CD19	1	31201550	31236569	ENST00000476492.1	LAPTM5	0.30	0.04	0.26
CD19	10	11184898	11223017	ENST00000537122.1	CELF2	0.32	0.06	0.26
CD19	6	32402294	32411438	ENST00000374982.5	HLA-DRA	0.36	0.10	0.26
CD19	15	86229664	86252170	ENST00000560482.1	AKAP13	0.31	0.05	0.26
CD19	8	11340662	11367244	ENST00000529894.1	BLK	0.26	0.00	0.26
CD19	11	35097683	35106244	ENST00000598940.1	AL356215.1	0.31	0.05	0.26
CD19	4	40174339	40216705	ENST00000503978.1	RHOH	0.30	0.04	0.26
CD19	19	42374270	42392833	ENST00000337665.4	ARHGEF1	0.44	0.19	0.25
CD19	8	56790857	56809092	ENST00000520220.1	LYN	0.35	0.10	0.25
CD19	11	61120450	61130574	ENST00000423772.2	TMEM138	0.45	0.20	0.25
CD19	10	49874683	49897623	ENST00000360890.2	WDFY4	0.25	0.00	0.25
CD19	22	24821855	24835079	ENST00000439591.1	ADORA2A	0.26	0.02	0.25
CD19	16	29346657	29372711	ENST00000356328.3	SNX29P2	0.24	0.00	0.24
CD19	17	3807403	3821299	ENST00000571637.1	P2RX1	0.34	0.10	0.24
CD19	19	35807029	35840479	ENST00000593704.1	CD22	0.26	0.02	0.24
CD19	9	134127597	134154499	ENST00000464831.1	FAM78A	0.35	0.11	0.24
CD19	6	149785200	149817360	ENST00000462655.1	ZC3H12D	0.28	0.04	0.24
CD19	11	128582503	128610944	ENST00000534087.1	FLI1	0.28	0.04	0.24
CD19	1	21618391	21627146	ENST00000527991.1	ECE1	0.26	0.02	0.24
CD19	11	72850513	72872020	ENST00000422375.1	FCHSD2	0.41	0.17	0.24
CD19	16	81803418	81877366	ENST00000569523.1	PLCG2	0.26	0.02	0.24
CD19	2	233923908	233972771	ENST00000467393.1	INPP5D	0.29	0.05	0.24
CD19	16	85921635	85951991	ENST00000569607.1	IRF8	0.29	0.05	0.24
CD19	17	74476430	74497452	ENST00000590288.1	RHBDF2	0.36	0.13	0.23
CD19	20	4789416	4805261	ENST00000379400.3	RASSF2	0.41	0.18	0.23
CD19	22	47159882	47174367	ENST00000406733.1	TBC1D22A	0.27	0.03	0.23
CD19	5	88114708	88142027	ENST00000509373.1	MEF2C	0.24	0.01	0.23
CD19	20	46038978	46050263	ENST00000262975.4	ZMYND8	0.24	0.01	0.23
CD19	19	2610475	2632474	ENST00000587867.1	GNG7	0.31	0.09	0.22
CD19	22	42304034	42337858	ENST00000472374.2	CENPM	0.44	0.22	0.22
CD19	6	32807846	32814107	ENST00000414474.1	PSMB9	0.52	0.30	0.22
CD19	5	1479672	1506754	ENST00000507282.1	LPCAT1	0.23	0.01	0.22
CD19	6	150937029	150965213	ENST00000367326.1	PLEKHG1	0.25	0.03	0.22
CD19	18	2956157	2983444	ENST00000581568.1	LPIN2	0.23	0.02	0.22
CD19	6	159459555	159485569	ENST00000338313.5	TAGAP	0.27	0.05	0.22
CD19	7	35764330	35771796	ENST00000396081.1	HERPUD2	0.22	0.00	0.22
CD19	7	35764330	35771796	ENST00000311350.3	HERPUD2	0.22	0.00	0.22
CD19	11	118739056	118768630	ENST00000292174.4	CXCR5	0.25	0.04	0.22
CD19	7	150100445	150107156	ENST00000474836.1	ZNF775	0.56	0.35	0.21
CD19	19	10712789	10716244	ENST00000407327.4	SLC44A2	0.80	0.58	0.21
CD19	16	21367569	21393569	ENST00000542817.1	NPIPL3	0.21	0.00	0.21
CD19	4	56812204	56816635	ENST00000257287.4	CEP135	0.73	0.52	0.21
CD19	17	38004143	38027814	ENST00000377940.3	ZPBP2	0.26	0.05	0.21
CD19	20	43204843	43247481	ENST00000349959.3	PKIG	0.24	0.03	0.21
CD19	14	89768030	89775675	ENST00000555658.1	FOXN3	0.30	0.09	0.21
CD19	14	69147805	69153516	ENST00000555997.1	ZFP36L1	0.23	0.03	0.21
CD19	3	5047573	5059145	ENST00000460806.1	BHLHE40	0.23	0.02	0.20
CD19	11	58967706	58992697	ENST00000361050.3	MPEG1	0.22	0.02	0.20
CD19	16	10959821	11016093	ENST00000572665.1	CIITA	0.28	0.07	0.20
CD19	1	150122093	150138999	ENST00000485470.1	PLEKHO1	0.34	0.13	0.20
CD19	20	55964442	55977216	ENST00000371219.2	RBM38	0.53	0.33	0.20
CD19	3	196350844	196371448	ENST00000426755.1	PIGX	0.34	0.14	0.20
CD19	17	45276453	45285339	ENST00000571981.1	MYL4	0.23	0.04	0.20
CD19	15	57571264	57599710	ENST00000560948.1	TCF12	0.24	0.04	0.19
CD19	22	18476769	18494405	ENST00000424046.1	MICAL3	0.32	0.12	0.19
CD19	1	206730430	206756416	ENST00000304534.8	RASSF5	0.25	0.05	0.19
CD19	15	74666045	74697205	ENST00000268053.6	CYP11A1	0.31	0.12	0.19

CD19	11	122930787	122942297	ENST00000531063.1	HSPA8	0.53	0.34	0.19
CD19	16	85965145	85983944	ENST00000569607.1	IRF8	0.21	0.02	0.19
CD19	9	126959034	126982108	ENST00000373603.1	NEK6	0.20	0.02	0.19
CD19	9	126959034	126982108	ENST00000540326.1	NEK6	0.20	0.02	0.19
CD19	18	60804706	60831957	ENST00000590515.1	BCL2	0.25	0.07	0.19
CD19	17	61992715	62032882	ENST00000584310.1	SCN4A	0.22	0.04	0.19
CD19	2	136871650	136898787	ENST00000466288.1	CXCR4	0.33	0.14	0.18
CD19	1	27984903	27990153	ENST00000339145.4	IFI6	0.51	0.32	0.18
CD19	22	37613897	37642449	ENST00000401529.3	RAC2	0.28	0.09	0.18
CD19	1	207078366	207106998	ENST00000487208.1	PIGR	0.23	0.05	0.18
CD19	10	11703130	11728646	ENST00000379237.1	USP6NL	0.23	0.05	0.18
CD19	6	32904722	32916498	ENST00000416244.2	HLA-DMB	0.27	0.09	0.18
CD19	14	64966362	64978672	ENST00000555321.1	ZBTB1	0.55	0.37	0.18
CD19	2	71290385	71301563	ENST00000524537.1	NAGK	0.39	0.21	0.18
CD19	3	58318904	58343543	ENST00000491164.1	PXK	0.25	0.07	0.18
CD19	5	163334703	163344988	ENST00000521838.1	MAT2B	0.20	0.03	0.18
CD19	6	41671523	41703442	ENST00000433032.1	TFEB	0.33	0.15	0.18
CD19	3	10232905	10242463	ENST00000256458.4	IRAK2	0.33	0.16	0.18
CD19	1	172348783	172369092	ENST00000465374.1	PIGC	0.21	0.03	0.17
CD19	2	128144112	128148979	ENST00000409179.2	MAP3K2	0.62	0.45	0.17
CD19	21	44817042	44836022	ENST00000478426.1	SIK1	0.22	0.05	0.17
CD19	12	48196588	48213374	ENST00000445237.2	HDAC7	0.40	0.23	0.17
CD19	11	67034303	67056724	ENST00000447274.2	ANKRD13D	0.40	0.23	0.17
CD19	22	22510876	22526241	ENST00000403807.3	VPREB1	0.23	0.07	0.17
CD19	9	139115225	139137596	ENST00000455222.1	QSOX2	0.22	0.05	0.17
CD19	7	138778458	138805019	ENST00000471652.1	ZC3HAV1	0.29	0.12	0.17
CD19	14	106023918	106072429	ENST00000431372.1	TMEM121	0.21	0.04	0.17
CD19	21	45556978	45601335	ENST00000598764.1	AP001055.1	0.28	0.12	0.17
CD19	3	122291690	122308644	ENST00000483793.1	PARP15	0.20	0.04	0.16
CD19	18	74764261	74781577	ENST00000580473.1	MBP	0.29	0.13	0.16
CD19	16	84618823	84644362	ENST00000561707.1	COTL1	0.20	0.04	0.16
CD19	9	35616119	35651731	ENST00000474403.1	SIT1	0.23	0.07	0.16
CD19	1	38937882	38948718	ENST00000474456.1	RRAGC	0.22	0.06	0.16
CD19	6	143160621	143189869	ENST00000367604.1	HIVEP2	0.22	0.06	0.16
CD19	6	143160621	143189869	ENST00000012134.2	HIVEP2	0.22	0.06	0.16
CD19	6	143160621	143189869	ENST00000474532.1	HIVEP2	0.22	0.06	0.16
CD19	3	107841403	107856868	ENST00000468021.1	IFT57	0.22	0.06	0.16
CD19	6	167362856	167374276	ENST00000496851.2	RNASET2	0.36	0.20	0.16
CD19	9	130665468	130681316	ENST00000479747.1	ST6GALNAC4	0.33	0.18	0.16
CD19	19	50917413	50938549	ENST00000357701.5	MYBPC2	0.42	0.26	0.16
CD19	13	46942847	46975976	ENST00000480935.1	KIAA0226L	0.24	0.08	0.16
CD19	2	238580170	238610065	ENST00000473815.1	LRRFIP1	0.35	0.19	0.16
CD19	12	92527263	92581890	ENST00000552315.1	BTG1	0.30	0.14	0.15
CD19	3	13034134	13065411	ENST00000473088.1	IQSEC1	0.30	0.15	0.15
CD19	11	73076425	73101853	ENST00000393580.2	RELT	0.21	0.06	0.15
CD19	11	82763568	82785588	ENST00000533276.1	RAB30	0.31	0.16	0.15
CD19	2	46761453	46803094	ENST00000473428.1	RHOQ	0.24	0.09	0.15
CD19	14	23018284	23039986	ENST00000557595.1	AE000662.92	0.25	0.10	0.15
CD19	12	7044970	7072648	ENST00000537533.1	PTPN6	0.36	0.21	0.15
CD19	4	82389749	82394691	ENST00000507538.1	RASGEF1B	0.74	0.59	0.14
CD19	12	113486672	113531373	ENST00000553140.1	DTX1	0.23	0.09	0.14
CD19	4	185452534	185460044	ENST00000393593.3	IRF2	0.39	0.25	0.14
CD19	12	9788063	9838708	ENST00000544322.1	CLEC2D	0.20	0.06	0.14
CD19	4	40289700	40319643	ENST00000310169.2	CHRNA9	0.21	0.06	0.14
CD19	17	38473833	38487686	ENST00000582914.1	RARA	0.58	0.44	0.14
CD19	7	142490667	142511468	ENST00000463701.1	PRSS1	0.26	0.12	0.14
CD19	1	111734737	111768995	ENST00000445067.2	CHI3L2	0.20	0.06	0.14
CD19	6	149077573	149093955	ENST00000367463.4	UST	0.21	0.07	0.14
CD19	2	231729691	231744201	ENST00000492029.1	ITM2C	0.33	0.19	0.14
CD19	6	144654443	144673365	ENST00000421035.1	UTRN	0.20	0.07	0.14
CD19	16	15728212	15738278	ENST00000396353.2	NDE1	0.42	0.28	0.14
CD19	10	90144149	90152479	ENST00000437752.1	RNLS	0.29	0.15	0.14
CD19	1	40838755	40871047	ENST00000539317.1	SMAP2	0.22	0.08	0.14
CD19	17	27064086	27073896	ENST00000584944.1	TRAF4	0.48	0.35	0.13
CD19	19	2040263	2096653	ENST00000395307.2	IZUMO4	0.32	0.19	0.13
CD19	9	3524176	3529109	ENST00000449190.1	RFX3	0.90	0.77	0.13
CD19	17	29814146	29838313	ENST00000578694.1	RAB11FIP4	0.24	0.11	0.13

CD19	13	41534432	41594336	ENST00000239882.3	ELF1	0.21	0.08	0.13
CD19	19	18278723	18292546	ENST00000600463.1	IFI30	0.23	0.10	0.13
CD19	6	106957303	106996547	ENST00000487681.1	AIM1	0.26	0.13	0.13
CD19	2	196511447	196525260	ENST00000418005.1	SLC39A10	0.39	0.26	0.13
CD19	17	75396031	75469717	ENST00000593189.1	9-Sep	0.28	0.15	0.12
CD19	14	50327610	50337580	ENST00000298310.5	NEMF	0.50	0.38	0.12
CD19	12	46657445	46665336	ENST00000546519.1	SLC38A1	0.57	0.45	0.12
CD19	16	11758829	11785379	ENST00000575349.1	TXNDC11	0.22	0.10	0.12
CD19	1	154914194	154929302	ENST00000490230.1	PBXIP1	0.29	0.16	0.12
CD19	1	2477758	2482788	ENST00000426449.1	TNFRSF14	0.72	0.59	0.12
CD19	2	54783925	54824402	ENST00000333896.5	SPTBN1	0.27	0.15	0.12
CD19	11	9621558	9642085	ENST00000527848.1	WEE1	0.21	0.09	0.12
CD19	15	45001549	45023195	ENST00000561237.1	TRIM69	0.36	0.24	0.12
CD19	20	44736001	44751095	ENST00000461171.1	CD40	0.21	0.09	0.12
CD19	12	110431794	110453837	ENST00000261739.4	ANKRD13A	0.28	0.16	0.12
CD19	2	202106787	202128005	ENST00000429881.1	CASP8	0.21	0.10	0.11
CD19	8	29937591	29951059	ENST00000521083.1	TMEM66	0.29	0.18	0.11
CD19	22	31680149	31688920	ENST00000443175.1	PIK3IP1	0.44	0.33	0.11
CD19	9	123686905	123707487	ENST00000540010.1	TRAF1	0.30	0.19	0.11
CD19	10	112618797	112633729	ENST00000444997.1	PDCD4	0.35	0.25	0.11
CD19	16	28921620	28948628	ENST00000567368.1	CD19	0.20	0.10	0.11
CD19	14	105116134	105145145	ENST00000330634.7	INF2	0.23	0.12	0.11
CD19	5	122109910	122117182	ENST00000514949.1	SNX2	0.41	0.31	0.11
CD19	18	2634081	2658907	ENST00000261598.8	SMCHD1	0.25	0.15	0.10
CD19	19	39887963	39904206	ENST00000438123.1	PLEKHG2	0.70	0.60	0.10
CD19	1	66795175	66818506	ENST00000526197.1	PDE4B	0.28	0.18	0.10
Colon	20	43968274	43977802	ENST00000537976.1	SDC4	0.77	0.29	0.48
Colon	1	2505891	2510796	ENST00000493183.1	FAM213B	0.61	0.15	0.46
Colon	17	57904024	57931778	ENST00000587470.1	VMP1	0.49	0.14	0.35
Colon	6	74224863	74234116	ENST00000455918.1	EEF1A1	0.90	0.58	0.32
Colon	7	27210777	27221249	ENST00000396344.4	HOXA10	0.76	0.45	0.30
Colon	17	37780840	37791262	ENST00000580029.1	PPP1R1B	0.49	0.19	0.30
Colon	10	3846234	3854755	ENST00000542957.1	KLF6	0.42	0.13	0.29
Colon	1	19966426	19978402	ENST00000427894.1	NBL1	0.54	0.25	0.28
Colon	15	75072089	75083506	ENST00000567571.1	CSK	0.80	0.52	0.28
Colon	5	79542438	79554102	ENST00000513907.1	SERINC5	0.51	0.26	0.25
Colon	17	76164003	76173458	ENST00000592456.1	SYNGR2	0.53	0.29	0.24
Colon	21	42931018	42955389	ENST00000454499.1	TMPRSS2	0.24	0.01	0.23
Colon	1	207111967	207121081	ENST00000491503.1	PIGR	0.23	0.00	0.23
Colon	13	28526778	28556061	ENST00000548877.1	CDX2	0.94	0.72	0.23
Colon	15	86121044	86133400	ENST00000560340.1	AKAP13	0.40	0.18	0.22
Colon	18	3446352	3459783	ENST00000472042.1	TGIF1	0.77	0.55	0.22
Colon	14	50465553	50471375	ENST00000529902.1	C14orf182	0.66	0.45	0.22
Colon	16	29830344	29838029	ENST00000570234.1	MVP	0.48	0.28	0.19
Colon	3	193848397	193860794	ENST00000476918.1	HES1	0.80	0.61	0.19
Colon	12	7051408	7057319	ENST00000538318.1	PTPN6	0.59	0.40	0.18
Colon	1	159888888	159896553	ENST00000397334.2	TAGLN2	0.63	0.45	0.18
Colon	17	7161661	7168259	ENST00000573745.1	CLDN7	0.63	0.45	0.18
Colon	6	31700974	31708664	ENST00000493662.2	MSH5-SAPCD1	0.87	0.70	0.18
Colon	20	52195018	52241000	ENST00000540425.1	ZNF217	0.41	0.24	0.17
Colon	3	49056900	49060957	ENST00000480392.1	NDUFAF3	0.84	0.67	0.17
Colon	12	56472347	56482109	ENST00000546748.1	ERBB3	0.44	0.27	0.17
Colon	11	130013608	130061419	ENST00000530376.1	ST14	0.22	0.05	0.16
Colon	2	200306225	200342782	ENST00000463386.1	SATB2	0.56	0.40	0.16
Colon	16	2028149	2036628	ENST00000567719.1	GFER	0.59	0.43	0.16
Colon	4	38662929	38689928	ENST00000436901.1	AC021860.1	0.45	0.29	0.16
Colon	8	126438172	126449618	ENST00000519576.1	TRIB1	0.52	0.36	0.16
Colon	1	1092807	1104591	ENST00000506177.1	TTLL10	0.35	0.19	0.16
Colon	19	49463688	49472130	ENST00000331825.6	FTL	0.54	0.39	0.15
Colon	1	37936771	37953471	ENST00000471012.1	ZC3H12A	0.49	0.34	0.15
Colon	7	150052314	150083105	ENST00000486297.1	ZNF775	0.36	0.21	0.15
Colon	2	173291273	173330716	ENST00000409080.1	ITGA6	0.25	0.10	0.15
Colon	19	13948788	13956320	ENST00000591727.1	NANOS3	0.69	0.55	0.15
Colon	11	307666	312937	ENST00000399815.2	IFITM2	0.70	0.55	0.14
Colon	11	118659296	118664159	ENST00000533239.1	DDX6	1.00	0.86	0.14
Colon	10	134259441	134282106	ENST00000392630.3	C10orf91	0.26	0.12	0.14
Colon	10	134259441	134282106	ENST00000321248.2	C10orf91	0.26	0.12	0.14

Colon	4	102266379	102270939	ENST00000529296.1	AP001816.1	0.94	0.80	0.14
Colon	1	150531037	150543263	ENST00000369035.2	C1orf138	0.51	0.37	0.14
Colon	17	27275334	27280852	ENST00000577182.1	PIPOX	1.00	0.86	0.14
Colon	1	149819766	149826428	ENST00000403683.1	HIST2H3A	0.28	0.14	0.14
Colon	11	71748625	71754435	ENST00000535947.1	NUMA1	0.81	0.67	0.13
Colon	5	96268784	96273156	ENST00000231368.5	LNPEP	0.95	0.81	0.13
Colon	19	1256417	1263739	ENST00000589161.1	CIRBP	0.65	0.52	0.13
Colon	11	64654606	64662582	ENST00000457202.1	EHD1	0.23	0.09	0.13
Colon	2	201980044	201998492	ENST00000460961.1	CFLAR	0.57	0.44	0.13
Colon	3	9436663	9444883	ENST00000406341.1	SETD5	0.78	0.65	0.13
Colon	17	4845115	4854686	ENST00000519300.1	ENO3	0.72	0.58	0.13
Colon	1	153503987	153511480	ENST00000462951.2	S100A6	0.39	0.26	0.13
Colon	10	90638872	90664402	ENST00000371924.1	STAMBPL1	0.20	0.07	0.13
Colon	9	140187219	140215940	ENST00000356628.2	NRARP	0.53	0.40	0.13
Colon	5	180668526	180674519	ENST00000514318.1	GNB2L1	0.82	0.70	0.13
Colon	2	174828021	174831622	ENST00000490182.1	SP3	1.00	0.88	0.12
Colon	1	1675407	1679081	ENST00000246421.4	SLC35E2	0.74	0.62	0.12
Colon	2	220109452	220119946	ENST00000392088.2	TUBA4A	0.53	0.40	0.12
Colon	12	53762866	53777584	ENST00000548560.1	SP1	0.41	0.29	0.12
Colon	7	1486269	1507327	ENST00000297508.7	MICALL2	0.35	0.23	0.12
Colon	19	1852750	1865843	ENST00000592313.1	KLF16	0.63	0.51	0.12
Colon	6	33538390	33559780	ENST00000374458.1	GGNBP1	0.27	0.16	0.12
Colon	4	40185249	40208107	ENST00000503978.1	RHOH	0.25	0.13	0.12
Colon	7	104643451	104656449	ENST00000474203.1	MLL5	0.62	0.51	0.12
Colon	8	103816742	103826153	ENST00000518697.1	AZIN1	0.54	0.43	0.12
Colon	2	151323963	151344439	ENST00000454202.1	RND3	0.28	0.17	0.11
Colon	1	169069278	169085972	ENST00000367813.3	ATP1B1	0.45	0.34	0.11
Colon	17	38213719	38232994	ENST00000577486.1	THRA	0.55	0.44	0.11
Colon	12	6559071	6563067	ENST00000543567.1	TAPBPL	0.75	0.64	0.11
Colon	17	4033794	4049258	ENST00000573984.1	CYB5D2	0.27	0.16	0.11
Colon	5	179244886	179250297	ENST00000360718.5	SQSTM1	0.68	0.57	0.11
Colon	7	100608082	100627662	ENST00000536621.1	MUC12	0.30	0.20	0.11
Colon	17	7459695	7466311	ENST00000429205.2	SENP3	0.74	0.63	0.11
Colon	7	27202748	27210249	ENST00000489695.1	HOXA9	0.95	0.85	0.10
Colon	6	32934747	32951807	ENST00000482838.1	BRD2	0.61	0.51	0.10
Colon	3	50373024	50380264	ENST00000490675.1	ZMYND10	0.49	0.39	0.10
Lung	20	23060957	23069729	ENST00000246006.4	CD93	0.23	0.01	0.21
Lung	17	7378645	7384073	ENST00000380599.4	ZBTB4	0.65	0.45	0.20
Lung	12	6441703	6453465	ENST00000538363.1	TNFRSF1A	0.61	0.45	0.16
Lung	2	66659175	66674430	ENST00000475239.1	MEIS1	0.78	0.63	0.16
Lung	22	46464333	46478321	ENST00000443490.1	MIRLET7	0.85	0.71	0.15
Lung	20	22536539	22565230	ENST00000319993.4	FOXA2	0.69	0.54	0.15
Lung	1	145434374	145443479	ENST00000486597.1	TXNIP	0.71	0.57	0.15
Lung	12	92526891	92540701	ENST00000552315.1	BTG1	0.71	0.57	0.14
Lung	14	105938037	105944737	ENST00000548309.1	CRIP2	0.80	0.66	0.14
Lung	5	43033834	43045550	ENST00000314890.3	ANXA2R	0.57	0.43	0.14
Lung	4	174427206	174460857	ENST00000505300.1	HAND2	0.74	0.60	0.14
Lung	9	35726029	35733751	ENST00000486056.1	CREB3	0.65	0.53	0.12
Lung	1	154939647	154948901	ENST00000473344.1	CKS1B	0.61	0.48	0.12
Lung	19	45244285	45262369	ENST00000473473.1	BCL3	0.46	0.34	0.12
Lung	12	119613625	119628282	ENST00000542496.1	HSPB8	0.22	0.10	0.12
Lung	1	2160113	2167961	ENST00000508416.1	SKI	0.66	0.55	0.12
Lung	16	56638742	56646907	ENST00000567300.1	MT2A	0.50	0.39	0.12
Lung	11	67803279	67809641	ENST00000533947.1	TCIRG1	0.45	0.34	0.11
Lung	19	13948720	13956318	ENST00000591727.1	NANOS3	0.66	0.55	0.11
Lung	11	305282	312843	ENST00000399815.2	IFITM2	0.50	0.40	0.10

Supplementary Table 3: Validation of tissue-specific DNA methylation at super-enhancers in normal tissue types.

	Super-Enhancer					HMR coverage (%)			DNA Methylation 450K		Significance	
Tissue	Chr.	Start	End	Transcript ID	Gene	Tissue	Others	Diff.	AVR Tissue	AVR Others	-log10(p-value)	Validated
Brain	10	126400866	126432679	ENST00000392754.3	FAM53B	0.40	0.19	0.21	0.27	0.32	8.42	TRUE
Brain	11	67173218	67188540	ENST00000531388.1	CARNS1	0.45	0.21	0.24	0.19	0.25	5.85	TRUE
Brain	1	205195003	205257756	ENST00000481950.1	TMCC2	0.23	0.09	0.14	0.23	0.27	4.99	TRUE
Brain	17	72226877	72248627	ENST00000526858.1	TTYH2	0.23	0.03	0.20	0.28	0.45	4.55	TRUE
Brain	1	205626881	205650899	ENST00000367145.3	SLC45A3	0.29	0.14	0.15	0.06	0.07	4.38	TRUE
Brain	22	37940770	37968154	ENST00000434728.1	CDC42EP1	0.50	0.25	0.25	0.20	0.26	4.33	TRUE
Brain	7	124359038	124407316	ENST00000303921.2	GPR37	0.51	0.05	0.46	0.23	0.30	4.25	TRUE
Brain	1	221879013	221917508	ENST00000477026.1	DUSP10	0.32	0.09	0.23	0.16	0.19	3.98	TRUE
Brain	17	27071068	27095625	ENST00000584059.1	FAM222B	0.29	0.14	0.15	0.12	0.16	3.98	TRUE
Brain	7	28724048	28800494	ENST00000468813.1	CREB5	0.27	0.04	0.23	0.26	0.35	3.87	TRUE
Brain	14	65146034	65196928	ENST00000556801.1	PLEKHG3	0.31	0.11	0.20	0.20	0.26	3.83	TRUE
Brain	19	51033029	51070726	ENST00000599957.1	LRRC4B	0.30	0.17	0.14	0.32	0.43	3.80	TRUE
Brain	6	41649397	41702285	ENST00000433032.1	TFEB	0.30	0.10	0.20	0.30	0.38	3.78	TRUE
Brain	21	48018786	48058971	ENST00000440086.1	PRMT2	0.35	0.09	0.26	0.27	0.32	3.64	TRUE
Brain	1	149967151	149985599	ENST00000417191.1	OTUD7B	0.27	0.11	0.16	0.15	0.17	3.55	TRUE
Brain	11	73357627	73375282	ENST00000544282.1	PLEKHB1	0.20	0.08	0.13	0.18	0.21	3.31	TRUE
Brain	10	81137824	81212605	ENST00000372333.3	ZCCHC24	0.43	0.09	0.33	0.23	0.27	3.26	TRUE
Brain	12	49579740	49630368	ENST00000552125.1	TUBA1C	0.23	0.09	0.13	0.18	0.24	3.22	TRUE
Brain	1	109889550	109945440	ENST00000471996.1	SORT1	0.26	0.05	0.20	0.23	0.30	3.11	TRUE
Brain	6	30844750	30864481	ENST00000514434.1	DDR1	0.45	0.18	0.27	0.28	0.37	3.10	TRUE
Brain	11	118463235	118534958	ENST00000528823.1	PHLDB1	0.23	0.10	0.13	0.26	0.31	3.09	TRUE
Brain	17	57405868	57485233	ENST00000582813.1	YPEL2	0.20	0.06	0.14	0.15	0.19	3.03	TRUE
Brain	1	214156400	214205391	ENST00000261454.4	PROX1	0.32	0.13	0.19	0.22	0.25	3.01	TRUE
Brain	4	77099124	77138142	ENST00000512895.1	FAM47E	0.30	0.14	0.16	0.16	0.19	3.01	TRUE
Brain	1	202090299	202115217	ENST00000272217.2	ARL8A	0.39	0.17	0.23	0.25	0.35	2.95	TRUE
Brain	7	141373362	141403139	ENST00000482493.1	KIAA1147	0.22	0.06	0.16	0.25	0.29	2.93	TRUE
Brain	13	67782754	67806323	ENST00000377861.3	PCDH9	0.59	0.20	0.39	0.18	0.23	2.91	TRUE
Brain	2	232526039	232554844	ENST00000466801.1	PTMA	0.34	0.23	0.12	0.18	0.20	2.87	TRUE
Brain	17	76857381	76931557	ENST00000262768.7	TIMP2	0.23	0.09	0.14	0.33	0.39	2.79	TRUE
Brain	1	160019557	160042341	ENST00000368089.3	KCNJ10	0.23	0.07	0.16	0.19	0.22	2.72	TRUE
Brain	5	173313778	173354104	ENST00000519467.1	CPEB4	0.34	0.08	0.25	0.17	0.23	2.70	TRUE
Brain	3	156845617	156856684	ENST00000467849.1	CCNL1	0.30	0.15	0.15	0.23	0.30	2.70	TRUE
Brain	16	57277838	57336233	ENST00000569059.1	PLLP	0.30	0.10	0.20	0.13	0.16	2.70	TRUE
Brain	10	111967550	111992938	ENST00000369612.1	MXI1	0.40	0.29	0.11	0.15	0.16	2.68	TRUE
Brain	2	164566271	164594418	ENST00000482917.1	FIGN	0.35	0.11	0.25	0.20	0.25	2.63	TRUE

Brain	15	45655307	45688573	ENST00000558118.1	GATM	0.24	0.05	0.19	0.20	0.28	2.62	TRUE
Brain	1	204796255	204866707	ENST00000514644.1	NFASC	0.21	0.03	0.18	0.30	0.37	2.60	TRUE
Brain	2	127806748	127912517	ENST00000409400.1	BIN1	0.36	0.06	0.30	0.21	0.26	2.53	TRUE
Brain	6	134488737	134505092	ENST00000525700.1	SGK1	0.60	0.27	0.34	0.24	0.29	2.51	TRUE
Brain	5	139012918	139146609	ENST00000505812.1	CXXC5	0.36	0.22	0.14	0.23	0.26	2.43	TRUE
Brain	9	140081775	140094696	ENST00000477345.1	TPRN	0.56	0.20	0.35	0.25	0.29	2.39	TRUE
Brain	14	77765872	77789479	ENST00000555093.1	GSTZ1	0.39	0.12	0.27	0.19	0.22	2.37	TRUE
Brain	5	646895	697805	ENST00000360578.5	TPPP	0.23	0.12	0.12	0.27	0.32	2.34	TRUE
Brain	3	134072004	134096230	ENST00000502491.1	AMOTL2	0.44	0.19	0.25	0.19	0.22	2.33	TRUE
Brain	17	40114067	40162277	ENST00000591153.1	DNAJC7	0.27	0.07	0.20	0.33	0.38	2.32	TRUE
Brain	9	130697475	130743184	ENST00000494606.1	FAM102A	0.61	0.17	0.44	0.27	0.32	2.32	TRUE
Brain	1	230932874	231004412	ENST00000366663.5	C1orf198	0.21	0.03	0.18	0.29	0.34	2.31	TRUE
Brain	10	72971329	73044360	ENST00000373192.4	UNC5B	0.21	0.09	0.12	0.20	0.23	2.30	TRUE
Brain	1	160047565	160068437	ENST00000460351.1	IGSF8	0.35	0.14	0.21	0.29	0.31	2.25	TRUE
Brain	12	26265040	26280746	ENST00000534829.1	SSPN	0.64	0.47	0.17	0.14	0.16	2.12	TRUE
Brain	1	109816196	109827024	ENST00000471740.1	PSRC1	0.36	0.17	0.19	0.16	0.18	2.11	TRUE
Brain	19	13093539	13211813	ENST00000588680.1	NFIX	0.47	0.21	0.26	0.29	0.34	2.10	TRUE
Brain	14	90845063	90876551	ENST00000556721.1	CALM1	0.35	0.20	0.15	0.09	0.10	2.08	TRUE
Brain	3	11024732	11080079	ENST00000495636.1	SLC6A1	0.23	0.06	0.17	0.20	0.24	2.07	TRUE
Brain	1	32792421	32824727	ENST00000574315.1	TSSK3	0.27	0.14	0.12	0.18	0.19	2.07	TRUE
Brain	17	17638844	17657613	ENST00000395774.1	RAI1	0.30	0.16	0.14	0.25	0.29	2.06	TRUE
Brain	19	17858364	17882158	ENST00000600393.1	FCHO1	0.22	0.11	0.11	0.21	0.24	2.01	TRUE
Brain	7	45111554	45132881	ENST00000490531.2	NACAD	0.22	0.07	0.15	0.27	0.31	1.99	TRUE
Brain	2	220142124	220157631	ENST00000460801.1	PTPRN	0.43	0.16	0.27	0.31	0.35	1.96	TRUE
Brain	11	134254603	134298147	ENST00000531510.1	B3GAT1	0.26	0.11	0.15	0.22	0.26	1.94	TRUE
Brain	6	163815822	163882069	ENST00000537883.1	QKI	0.79	0.13	0.66	0.17	0.18	1.89	TRUE
Brain	19	50985631	51019282	ENST00000598657.1	ASPDH	0.28	0.11	0.17	0.28	0.34	1.86	TRUE
Brain	1	155828272	155864174	ENST00000539162.1	SYT11	0.21	0.06	0.15	0.14	0.17	1.85	TRUE
Brain	1	205461954	205494873	ENST00000505932.1	CDK18	0.26	0.06	0.20	0.23	0.26	1.84	TRUE
Brain	4	154142334	154219000	ENST00000433687.1	TRIM2	0.38	0.05	0.32	0.31	0.33	1.80	TRUE
Brain	7	30174536	30225450	ENST00000455738.1	C7orf41	0.23	0.06	0.18	0.30	0.34	1.80	TRUE
Brain	9	124022263	124092639	ENST00000477553.1	GSN	0.26	0.12	0.15	0.30	0.34	1.77	TRUE
Brain	5	36599886	36615091	ENST00000509272.1	SLC1A3	0.50	0.11	0.38	0.17	0.20	1.76	TRUE
Brain	1	61541625	61624014	ENST00000496712.1	NFIA	0.24	0.11	0.13	0.12	0.14	1.76	TRUE
Brain	17	56589712	56620235	ENST00000582976.1	SEPT4	0.36	0.19	0.17	0.21	0.23	1.72	TRUE
Brain	10	124216441	124276238	ENST00000420892.1	HTRA1	0.27	0.07	0.20	0.30	0.34	1.64	TRUE
Brain	8	26427962	26519962	ENST00000474808.1	DPYSL2	0.45	0.10	0.35	0.33	0.37	1.59	TRUE
Brain	9	14299740	14327190	ENST00000493697.1	NFIB	0.57	0.42	0.15	0.23	0.27	1.56	TRUE
Brain	6	52225169	52265846	ENST00000360726.3	PAQR8	0.21	0.07	0.14	0.12	0.15	1.50	TRUE

Brain	2	145129528	145283099	ENST00000475115.1	ZEB2	0.39	0.08	0.30	0.21	0.24	1.50	TRUE
Brain	1	90285126	90311060	ENST00000527156.1	LRRC8D	0.33	0.14	0.18	0.05	0.06	1.48	TRUE
Brain	12	123317747	123395518	ENST00000371248.3	VPS37B	0.30	0.13	0.17	0.31	0.33	1.47	TRUE
Brain	1	160161970	160191690	ENST00000481831.1	DCAF8	0.25	0.09	0.17	0.16	0.19	1.44	TRUE
Brain	14	50976072	51001976	ENST00000441560.2	ATL1	0.36	0.11	0.26	0.14	0.15	1.34	TRUE
Brain	5	132105879	132116081	ENST00000492490.1	SEPT8	0.43	0.21	0.22	0.21	0.25	1.31	TRUE
Brain	22	46445235	46456789	ENST00000381051.2	MIRLET7	0.63	0.47	0.16	0.19	0.23	1.30	TRUE
Brain	20	31025912	31074699	ENST00000326071.4	C20orf112	0.35	0.16	0.19	0.11	0.13	1.27	FALSE
Brain	1	160068777	160077945	ENST00000448417.1	IGSF8	0.44	0.14	0.30	0.26	0.28	1.25	FALSE
Brain	17	42161429	42202224	ENST00000587135.1	HDAC5	0.23	0.10	0.14	0.17	0.18	1.24	FALSE
Brain	6	15245470	15269892	ENST00000397311.3	JARID2	0.36	0.25	0.11	0.08	0.08	1.24	FALSE
Brain	2	95678307	95745847	ENST00000349807.3	MAL	0.27	0.08	0.19	0.21	0.24	1.24	FALSE
Brain	5	126625320	126648566	ENST00000274473.6	MEGF10	0.30	0.06	0.24	0.27	0.32	1.16	FALSE
Brain	12	6639174	6665533	ENST00000396830.2	IFFO1	0.40	0.29	0.11	0.18	0.20	1.15	FALSE
Brain	20	45922895	45992027	ENST00000441977.1	ZMYND8	0.24	0.13	0.11	0.16	0.19	1.14	FALSE
Brain	10	102752997	102775915	ENST00000454422.1	LZTS2	0.44	0.32	0.13	0.20	0.22	1.09	FALSE
Brain	7	32078425	32118455	ENST00000464881.1	PDE1C	0.23	0.06	0.17	0.22	0.26	1.07	FALSE
Brain	1	110071934	110092553	ENST00000369851.4	GNAI3	0.31	0.18	0.13	0.02	0.03	1.05	FALSE
Brain	10	64127809	64167185	ENST00000421210.1	ZNF365	0.23	0.03	0.20	0.24	0.26	1.04	FALSE
Brain	17	48914258	48946511	ENST00000509385.1	TOB1	0.28	0.17	0.11	0.17	0.18	1.01	FALSE
Brain	15	43800300	43824728	ENST00000300231.5	MAP1A	0.28	0.16	0.12	0.17	0.18	1.01	FALSE
Brain	1	33789227	33859751	ENST00000468406.1	PHC2	0.25	0.11	0.14	0.30	0.33	0.95	FALSE
Brain	6	69337682	69367340	ENST00000370598.1	BAI3	0.33	0.16	0.17	0.09	0.09	0.83	FALSE
Brain	20	35445944	35496093	ENST00000357779.3	SOGA1	0.30	0.11	0.20	0.30	0.31	0.82	FALSE
Brain	9	133698900	133750533	ENST00000318560.5	ABL1	0.29	0.08	0.21	0.33	0.36	0.81	FALSE
Brain	15	64974641	64996542	ENST00000559665.2	OAZ2	0.36	0.11	0.24	0.16	0.17	0.80	FALSE
Brain	2	9768796	9795405	ENST00000460093.1	YWHAQ	0.21	0.09	0.11	0.14	0.15	0.75	FALSE
Brain	3	181403893	181455295	ENST00000325404.1	SOX2	0.84	0.56	0.28	0.22	0.22	0.72	FALSE
Brain	11	111845328	111865086	ENST00000530645.1	DIXDC1	0.33	0.09	0.24	0.32	0.34	0.70	FALSE
Brain	6	128798636	128843173	ENST00000368202.4	PTPRK	0.20	0.09	0.11	0.08	0.08	0.67	FALSE
Brain	13	97862565	97936838	ENST00000445661.2	MBNL2	0.24	0.09	0.15	0.21	0.23	0.67	FALSE
Brain	17	38258962	38270871	ENST00000246672.3	NR1D1	0.42	0.31	0.11	0.15	0.16	0.64	FALSE
Brain	22	46457577	46466999	ENST00000396008.2	MIRLET7	0.94	0.50	0.44	0.31	0.35	0.64	FALSE
Brain	5	124064380	124097267	ENST00000512940.1	ZNF608	0.41	0.24	0.17	0.31	0.37	0.62	FALSE
Brain	12	1752842	1776949	ENST00000545747.1	WNT5B	0.24	0.13	0.11	0.29	0.31	0.37	FALSE
Brain	2	193010308	193063844	ENST00000409056.3	TMEFF2	0.21	0.06	0.15	0.22	0.23	0.37	FALSE
Brain	3	69130585	69168856	ENST00000485444.1	ARL6IP5	0.22	0.12	0.10	0.18	0.19	0.35	FALSE
Brain	5	115856316	115912087	ENST00000512156.1	SEMA6A	0.23	0.08	0.14	0.16	0.17	0.33	FALSE
Brain	12	48167031	48182319	ENST00000599515.1	AC004466.1	0.40	0.10	0.31	0.04	0.04	0.32	FALSE

Brain	10	73574974	73621362	ENST00000394934.1	PSAP	0.21	0.05	0.15	0.19	0.20	0.29	FALSE
Brain	21	43639119	43683392	ENST00000340588.4	ABCG1	0.22	0.11	0.11	0.19	0.20	0.28	FALSE
Brain	9	135976307	136024639	ENST00000372062.3	RALGDS	0.33	0.16	0.17	0.23	0.24	0.27	FALSE
Brain	19	54921528	54950915	ENST00000467939.1	TTYH1	0.38	0.18	0.20	0.20	0.22	0.24	FALSE
Brain	9	8840393	8880342	ENST00000481079.1	PTPRD	0.24	0.08	0.16	0.22	0.23	0.22	FALSE
Brain	4	134065738	134085565	ENST00000511112.1	PCDH10	0.73	0.42	0.30	0.16	0.15	0.16	FALSE
Brain	15	37371561	37406086	ENST00000559129.1	MEIS2	0.46	0.33	0.12	0.24	0.22	0.15	FALSE
Brain	3	170135527	170167574	ENST00000488989.1	CLDN11	0.34	0.07	0.27	0.32	0.30	0.13	FALSE
Brain	7	86272604	86301105	ENST00000421579.1	GRM3	0.22	0.09	0.13	0.12	0.11	0.12	FALSE
Brain	17	38216903	38234316	ENST00000577486.1	THRA	0.63	0.48	0.15	0.21	0.21	0.09	FALSE
Brain	4	96452170	96474433	ENST00000504962.1	UNC5C	0.48	0.15	0.33	0.12	0.11	0.03	FALSE
Brain	19	30713867	30786928	ENST00000591488.1	ZNF536	0.21	0.11	0.10	0.15	0.14	0.02	FALSE
Brain	10	24989499	25014855	ENST00000463892.1	ARHGAP21	0.33	0.22	0.12	0.15	0.16	-0.02	FALSE
Brain	14	51524492	51564122	ENST00000360392.4	TRIM9	0.20	0.07	0.13	0.22	0.21	-0.09	FALSE
Brain	17	68152783	68183190	ENST00000243457.3	KCNJ2	0.38	0.11	0.27	0.09	0.08	-0.10	FALSE
Brain	1	65712720	65745163	ENST00000483402.1	DNAJC6	0.34	0.10	0.24	0.24	0.21	-0.16	FALSE
Brain	11	6400761	6441884	ENST00000533407.1	APBB1	0.21	0.07	0.15	0.09	0.09	-0.24	FALSE
Brain	1	156353833	156405678	ENST00000495000.1	C1orf61	0.29	0.18	0.11	0.24	0.21	-0.27	FALSE
Brain	17	79345155	79376776	ENST00000307745.7	BAHCC1	0.54	0.37	0.17	0.18	0.17	-0.61	FALSE
Brain	21	34399873	34453980	ENST00000498799.1	OLIG1	0.28	0.18	0.11	0.19	0.17	-0.63	FALSE
Brain	11	119211319	119250680	ENST00000527843.1	USP2	0.22	0.10	0.12	0.19	0.17	-1.23	FALSE
Brain	10	88108134	88126246	ENST00000327946.7	GRID1	0.29	0.19	0.11	0.20	0.16	-1.70	FALSE
Breast	10	121410843	121447500	ENST00000450186.1	BAG3	0.21	0.06	0.15	0.26	0.39	18.11	TRUE
Breast	10	95184875	95242959	ENST00000488645.1	MYOF	0.21	0.04	0.17	0.27	0.42	15.89	TRUE
Breast	11	65678179	65686589	ENST00000530188.1	C11orf68	0.30	0.14	0.16	0.32	0.39	15.28	TRUE
Breast	1	207221596	207227576	ENST00000367079.2	PFKFB2	0.61	0.48	0.12	0.08	0.12	15.17	TRUE
Breast	2	106009478	106028025	ENST00000447958.1	FHL2	0.21	0.08	0.13	0.14	0.25	14.92	TRUE
Breast	11	6339234	6344196	ENST00000530979.1	PRKCDBP	0.61	0.34	0.26	0.14	0.25	14.91	TRUE
Breast	1	156065471	156100930	ENST00000392353.3	LMNA	0.56	0.18	0.38	0.16	0.23	14.72	TRUE
Breast	11	66821179	66830989	ENST00000532559.1	RHOD	0.33	0.16	0.17	0.17	0.26	14.21	TRUE
Breast	19	1154329	1184542	ENST00000587655.1	SBNO2	0.28	0.10	0.18	0.30	0.46	14.12	TRUE
Breast	11	114162169	114180527	ENST00000545255.1	NNMT	0.26	0.03	0.24	0.30	0.58	13.61	TRUE
Breast	2	112249648	112262948	ENST00000371162.4	AC108463.3	0.27	0.08	0.19	0.03	0.21	13.38	TRUE
Breast	19	13948994	13959464	ENST00000591727.1	NANOS3	0.68	0.56	0.13	0.19	0.30	13.12	TRUE
Breast	11	62306073	62328249	ENST00000531324.1	AHNAK	0.61	0.34	0.27	0.17	0.28	13.12	TRUE
Breast	2	87744763	87759323	ENST00000359481.4	PLGLB2	0.21	0.09	0.11	0.03	0.18	13.06	TRUE
Breast	1	6637843	6670728	ENST00000496707.1	KLHL21	0.28	0.17	0.12	0.16	0.25	12.95	TRUE
Breast	14	69241271	69268143	ENST00000408913.2	ZFP36L1	0.42	0.32	0.10	0.09	0.13	12.42	TRUE
Breast	20	51581498	51597746	ENST00000371497.4	TSHZ2	0.45	0.16	0.29	0.17	0.26	12.06	TRUE

Breast	8	62622640	62634995	ENST00000379449.6	ASPH	0.38	0.21	0.17	0.18	0.23	11.50	TRUE
Breast	4	169752417	169771288	ENST00000507735.1	PALLD	0.28	0.16	0.12	0.10	0.14	11.46	TRUE
Breast	1	16156416	16179578	ENST00000375759.3	SPEN	0.49	0.37	0.12	0.08	0.11	11.33	TRUE
Breast	1	181055984	181089589	ENST00000367577.4	IER5	0.32	0.13	0.19	0.16	0.19	11.21	TRUE
Breast	8	145008274	145031982	ENST00000527816.1	PLEC	0.62	0.34	0.27	0.21	0.28	11.19	TRUE
Breast	14	105432978	105441663	ENST00000555122.1	AHNAK2	0.43	0.23	0.20	0.14	0.23	10.98	TRUE
Breast	15	75930589	75963120	ENST00000569152.1	SNX33	0.27	0.16	0.11	0.11	0.16	10.81	TRUE
Breast	15	68548590	68582156	ENST00000566739.1	FEM1B	0.25	0.12	0.13	0.17	0.25	10.74	TRUE
Breast	20	43963799	43990114	ENST00000537976.1	SDC4	0.33	0.13	0.21	0.11	0.16	10.63	TRUE
Breast	2	36579464	36605312	ENST00000473403.1	CRIM1	0.55	0.19	0.35	0.21	0.32	10.63	TRUE
Breast	12	125388867	125425378	ENST00000542416.1	UBC	0.39	0.28	0.11	0.12	0.15	10.54	TRUE
Breast	12	10868156	10877012	ENST00000540747.1	CSDA	0.79	0.29	0.50	0.08	0.15	10.38	TRUE
Breast	6	111906089	111928358	ENST00000528599.1	TRAF3IP2	0.33	0.15	0.18	0.18	0.25	10.38	TRUE
Breast	17	2294949	2311496	ENST00000571836.2	MNT	0.62	0.42	0.20	0.12	0.14	10.21	TRUE
Breast	10	33226394	33252825	ENST00000439974.3	ITGB1	0.30	0.19	0.10	0.09	0.12	10.06	TRUE
Breast	14	55568260	55575863	ENST00000553493.1	LGALS3	0.32	0.13	0.19	0.17	0.41	10.04	TRUE
Breast	11	57045039	57094324	ENST00000527207.1	TNKS1BP1	0.23	0.09	0.13	0.11	0.20	9.99	TRUE
Breast	1	27830256	27900464	ENST00000487743.2	AHDC1	0.37	0.09	0.28	0.33	0.43	9.98	TRUE
Breast	16	56638741	56649586	ENST00000567300.1	MT2A	0.46	0.27	0.19	0.20	0.31	9.81	TRUE
Breast	22	36718824	36814131	ENST00000216181.5	MYH9	0.34	0.16	0.18	0.18	0.23	9.73	TRUE
Breast	1	27183345	27192842	ENST00000339276.4	SFN	0.33	0.22	0.11	0.29	0.37	9.65	TRUE
Breast	1	201404474	201474025	ENST00000532460.1	CSRP1	0.22	0.10	0.12	0.19	0.24	9.49	TRUE
Breast	3	187453456	187468930	ENST00000496823.1	BCL6	0.81	0.63	0.19	0.09	0.11	9.49	TRUE
Breast	11	8828385	8837469	ENST00000531237.1	ST5	0.63	0.12	0.51	0.32	0.48	9.25	TRUE
Breast	2	239193425	239200323	ENST00000431832.1	PER2	0.73	0.36	0.37	0.20	0.28	9.19	TRUE
Breast	19	47598365	47618174	ENST00000594526.1	SAE1	0.46	0.30	0.16	0.13	0.16	9.18	TRUE
Breast	11	9586185	9600407	ENST00000524612.1	WEE1	0.46	0.26	0.21	0.09	0.13	8.91	TRUE
Breast	14	77489392	77513614	ENST00000238647.3	IRF2BPL	0.67	0.37	0.29	0.17	0.24	8.75	TRUE
Breast	5	131593100	131603174	ENST00000418373.1	PDLIM4	0.60	0.08	0.51	0.29	0.40	8.73	TRUE
Breast	11	61732568	61749363	ENST00000601917.1	AP003733.1	0.38	0.24	0.14	0.09	0.11	8.53	TRUE
Breast	1	45270631	45276266	ENST00000482715.1	BTBD19	0.65	0.27	0.38	0.33	0.40	8.47	TRUE
Breast	19	42771661	42788152	ENST00000575839.1	CIC	0.57	0.44	0.13	0.15	0.17	8.26	TRUE
Breast	12	52608969	52643707	ENST00000544024.1	KRT86	0.21	0.06	0.15	0.27	0.36	8.01	TRUE
Breast	7	869464	876882	ENST00000469755.1	SUN1	0.22	0.07	0.15	0.29	0.44	7.97	TRUE
Breast	2	46523740	46563334	ENST00000475822.1	EPAS1	0.23	0.13	0.10	0.13	0.17	7.61	TRUE
Breast	20	17537085	17560729	ENST00000449141.2	DSTN	0.27	0.12	0.16	0.15	0.20	7.60	TRUE
Breast	9	130280649	130347137	ENST00000373314.3	FAM129B	0.20	0.10	0.10	0.29	0.37	7.59	TRUE
Breast	22	30792614	30822843	ENST00000407550.3	MTFP1	0.36	0.17	0.19	0.11	0.13	7.58	TRUE
Breast	12	109225745	109251401	ENST00000548522.1	SSH1	0.24	0.09	0.15	0.16	0.24	7.09	TRUE

Breast	14	23292133	23323062	ENST00000547596.1	MMP14	0.33	0.15	0.17	0.13	0.19	7.09	TRUE
Breast	1	27320120	27340181	ENST00000289166.5	FAM46B	0.33	0.18	0.16	0.14	0.17	7.04	TRUE
Breast	1	8062975	8088860	ENST00000487559.1	ERRFI1	0.25	0.14	0.10	0.16	0.21	6.92	TRUE
Breast	1	154941228	154949531	ENST00000473344.1	CKS1B	0.82	0.52	0.31	0.14	0.19	6.71	TRUE
Breast	19	41220087	41229010	ENST00000263370.2	ITPKC	0.69	0.55	0.14	0.09	0.11	6.65	TRUE
Breast	7	41734099	41745805	ENST00000442711.1	INHBA	0.40	0.26	0.14	0.13	0.19	6.64	TRUE
Breast	15	42562799	42569003	ENST00000567421.1	GANC	0.91	0.51	0.40	0.06	0.08	6.58	TRUE
Breast	5	142773722	142785691	ENST00000502500.1	NR3C1	0.95	0.51	0.44	0.10	0.15	6.55	TRUE
Breast	1	16274460	16281222	ENST00000494020.1	ZBTB17	0.21	0.07	0.14	0.32	0.48	6.16	TRUE
Breast	1	155097310	155110100	ENST00000484027.1	SLC50A1	0.63	0.51	0.12	0.09	0.11	6.11	TRUE
Breast	11	118779500	118810391	ENST00000534788.1	UPK2	0.63	0.45	0.18	0.14	0.20	5.75	TRUE
Breast	17	27134005	27140821	ENST00000582059.1	FAM222B	0.45	0.35	0.10	0.05	0.08	5.73	TRUE
Breast	22	46467114	46488203	ENST00000360737.3	MIRLET7	1.00	0.47	0.53	0.25	0.45	5.64	TRUE
Breast	1	33207559	33241755	ENST00000373480.1	KIAA1522	0.22	0.10	0.12	0.22	0.27	5.58	TRUE
Breast	22	46448058	46467049	ENST00000381051.2	MIRLET7	1.00	0.50	0.50	0.20	0.30	5.57	TRUE
Breast	7	55085115	55095372	ENST00000463948.1	EGFR	0.50	0.21	0.29	0.19	0.24	5.29	TRUE
Breast	11	65238595	65276583	ENST00000309775.7	AP000769.1	0.63	0.43	0.20	0.14	0.18	5.25	TRUE
Breast	10	82213990	82238676	ENST00000372156.1	TSPAN14	0.25	0.14	0.11	0.13	0.17	5.20	TRUE
Breast	17	46122636	46152031	ENST00000581319.1	NFE2L1	0.28	0.11	0.17	0.13	0.15	5.18	TRUE
Breast	11	57528367	57568939	ENST00000534647.1	CTNND1	0.31	0.11	0.20	0.16	0.30	5.02	TRUE
Breast	17	17723596	17746027	ENST00000435530.2	SREBF1	0.36	0.25	0.11	0.14	0.16	4.99	TRUE
Breast	9	124030410	124052489	ENST00000545652.1	GSN	0.40	0.26	0.15	0.21	0.23	4.78	TRUE
Breast	3	141083866	141089271	ENST00000507657.1	ZBTB38	0.39	0.08	0.32	0.17	0.34	4.74	TRUE
Breast	17	17578482	17616584	ENST00000471135.2	RAI1	0.26	0.15	0.10	0.18	0.21	4.67	TRUE
Breast	11	12694725	12726400	ENST00000334310.6	TEAD1	0.27	0.17	0.10	0.16	0.19	4.63	TRUE
Breast	12	6432600	6452223	ENST00000538363.1	TNFRSF1A	0.50	0.30	0.19	0.05	0.08	4.48	TRUE
Breast	17	7736868	7749068	ENST00000570632.1	KDM6B	0.92	0.77	0.15	0.07	0.08	4.31	TRUE
Breast	18	3446318	3460082	ENST00000472042.1	TGIF1	0.68	0.56	0.12	0.06	0.07	4.19	TRUE
Breast	20	19954385	20001261	ENST00000481837.1	NAA20	0.22	0.09	0.13	0.15	0.18	4.18	TRUE
Breast	1	19247868	19283180	ENST00000416166.1	IFFO2	0.29	0.14	0.15	0.21	0.23	3.72	TRUE
Breast	3	29321550	29334286	ENST00000456853.1	RBMS3	0.65	0.21	0.45	0.05	0.07	3.66	TRUE
Breast	6	138172161	138196002	ENST00000433680.1	TNFAIP3	0.29	0.14	0.14	0.07	0.10	3.62	TRUE
Breast	12	59300121	59315174	ENST00000548968.1	LRIG3	0.39	0.18	0.21	0.07	0.09	3.48	TRUE
Breast	18	3245538	3270102	ENST00000584539.1	MYL12B	0.43	0.25	0.18	0.06	0.08	3.41	TRUE
Breast	3	9436165	9466747	ENST00000468208.1	SETD5	0.30	0.18	0.12	0.11	0.13	3.16	TRUE
Breast	11	65185034	65198304	ENST00000531151.1	FRMD8	1.00	0.88	0.12	0.10	0.14	2.99	TRUE
Breast	18	46449301	46483832	ENST00000586093.1	SMAD7	0.47	0.32	0.15	0.15	0.18	2.93	TRUE
Breast	9	14298144	14323961	ENST00000493697.1	NFIB	0.71	0.42	0.28	0.22	0.27	2.74	TRUE
Breast	3	193849125	193860857	ENST00000476918.1	HES1	0.79	0.61	0.18	0.07	0.08	2.64	TRUE

Breast	7	42257859	42278946	ENST00000428534.1	GLI3	0.33	0.17	0.15	0.20	0.20	2.56	TRUE
Breast	21	36252652	36264673	ENST00000399237.2	RUNX1	0.93	0.58	0.35	0.13	0.17	2.54	TRUE
Breast	19	13123147	13172690	ENST00000358552.3	NFIX	0.50	0.29	0.20	0.32	0.35	2.35	TRUE
Breast	10	74002370	74038384	ENST00000471240.1	DDIT4	0.27	0.17	0.10	0.11	0.12	2.32	TRUE
Breast	4	95366945	95386196	ENST00000511767.1	PDLIM5	0.24	0.11	0.12	0.08	0.10	2.31	TRUE
Breast	19	16177568	16192210	ENST00000588483.1	TPM4	0.43	0.26	0.16	0.16	0.18	2.23	TRUE
Breast	6	10398835	10421638	ENST00000486038.1	TFAP2A	0.81	0.68	0.13	0.19	0.22	2.02	TRUE
Breast	2	43444985	43456496	ENST00000282388.3	ZFP36L2	1.00	0.88	0.12	0.05	0.06	1.99	TRUE
Breast	15	93425946	93432559	ENST00000555520.1	CHD2	0.87	0.61	0.26	0.01	0.02	1.91	TRUE
Breast	7	36426266	36435508	ENST00000418118.1	ANLN	0.36	0.23	0.12	0.06	0.07	1.86	TRUE
Breast	1	100109973	100129273	ENST00000263174.4	PALMD	0.24	0.03	0.21	0.16	0.23	1.86	TRUE
Breast	14	74207241	74227902	ENST00000421708.1	ELMSAN1	0.58	0.41	0.18	0.12	0.13	1.80	TRUE
Breast	19	49375182	49379790	ENST00000600406.1	PPP1R15A	0.78	0.60	0.18	0.07	0.09	1.63	TRUE
Breast	20	10634437	10656669	ENST00000254958.4	JAG1	0.49	0.22	0.27	0.06	0.06	1.58	TRUE
Breast	1	59245181	59252471	ENST00000371222.2	JUN	0.86	0.61	0.25	0.05	0.05	1.42	TRUE
Breast	4	103736345	103751180	ENST00000503282.1	UBE2D3	0.48	0.36	0.11	0.05	0.06	1.39	TRUE
Breast	20	34326330	34331841	ENST00000493853.1	RBM39	0.91	0.74	0.17	0.05	0.05	1.38	TRUE
Breast	10	106080462	106112294	ENST00000458723.1	ITPRIP	0.31	0.19	0.12	0.19	0.19	1.27	FALSE
Breast	16	23152633	23161835	ENST00000219689.7	USP31	0.37	0.24	0.12	0.02	0.03	1.01	FALSE
Breast	4	146653244	146658422	ENST00000510096.1	C4orf51	0.68	0.29	0.39	0.04	0.05	0.98	FALSE
Breast	6	74224616	74234296	ENST00000455918.1	EEF1A1	0.79	0.59	0.21	0.07	0.08	0.87	FALSE
Breast	6	136605683	136612552	ENST00000528229.1	BCLAF1	0.54	0.42	0.12	0.03	0.04	0.87	FALSE
Breast	6	121756401	121769000	ENST00000282561.3	GJA1	0.38	0.18	0.19	0.15	0.20	0.80	FALSE
Breast	15	50644604	50649357	ENST00000560896.1	GABPB1	0.96	0.80	0.15	0.04	0.04	0.79	FALSE
Breast	16	15735275	15740014	ENST00000396353.2	NDE1	0.64	0.54	0.10	0.04	0.04	0.76	FALSE
Breast	10	74051120	74097380	ENST00000473051.1	DNAJB12	0.27	0.14	0.13	0.13	0.13	0.68	FALSE
Breast	5	1881509	1890183	ENST00000513692.1	IRX4	0.97	0.78	0.19	0.19	0.22	0.63	FALSE
Breast	7	115849775	115868787	ENST00000485009.1	TES	0.21	0.11	0.10	0.05	0.06	0.60	FALSE
Breast	2	12841318	12863362	ENST00000405331.3	TRIB2	0.35	0.19	0.17	0.09	0.11	0.26	FALSE
Breast	9	33157786	33167403	ENST00000379731.4	B4GALT1	0.92	0.42	0.50	0.14	0.14	-0.02	FALSE
Breast	10	112252889	112273435	ENST00000468749.1	DUSP5	0.37	0.21	0.16	0.24	0.26	-0.15	FALSE
Breast	5	67510759	67574308	ENST00000520675.1	PIK3R1	0.27	0.10	0.16	0.08	0.07	-0.34	FALSE
Breast	3	71104592	71120542	ENST00000497553.1	FOXP1	0.41	0.28	0.13	0.13	0.12	-0.62	FALSE
Breast	5	2739262	2759651	ENST00000502957.1	IRX2	0.92	0.80	0.11	0.23	0.20	-1.21	FALSE
Breast	5	140887653	140909039	ENST00000468119.1	DIAPH1	0.23	0.11	0.12	0.32	0.26	-2.92	FALSE
Breast	8	116659885	116682409	ENST00000451156.1	TRPS1	0.42	0.17	0.26	0.20	0.15	-3.13	FALSE
Breast	12	109082915	109097018	ENST00000547170.1	CORO1C	0.31	0.21	0.11	0.24	0.14	-14.35	FALSE
CD19	17	56394652	56423683	ENST00000583624.1	BZRAP1	0.46	0.16	0.31	0.20	0.41	39.73	TRUE
CD19	1	111401311	111424334	ENST00000476408.1	CD53	0.29	0.00	0.29	0.12	0.41	37.85	TRUE

CD19	16	85965145	85983944	ENST00000569607.1	IRF8	0.21	0.02	0.19	0.30	0.57	37.13	TRUE
CD19	6	13258837	13278907	ENST00000481706.1	PHACTR1	0.29	0.01	0.28	0.19	0.58	36.71	TRUE
CD19	2	233923908	233972771	ENST00000467393.1	INPP5D	0.29	0.05	0.24	0.19	0.42	35.52	TRUE
CD19	1	31201550	31236569	ENST00000476492.1	LAPTM5	0.30	0.04	0.26	0.31	0.56	31.68	TRUE
CD19	22	50627294	50632183	ENST00000395829.1	TRABD	0.69	0.28	0.41	0.15	0.42	30.04	TRUE
CD19	18	74764261	74781577	ENST00000580473.1	MBP	0.29	0.13	0.16	0.28	0.54	29.54	TRUE
CD19	10	11184898	11223017	ENST00000537122.1	CELF2	0.32	0.06	0.26	0.13	0.29	28.19	TRUE
CD19	6	32807846	32814107	ENST00000414474.1	PSMB9	0.52	0.30	0.22	0.16	0.23	25.92	TRUE
CD19	1	167584400	167603373	ENST00000537350.1	RCSD1	0.37	0.08	0.29	0.20	0.36	25.14	TRUE
CD19	1	150122093	150138999	ENST00000485470.1	PLEKHO1	0.34	0.13	0.20	0.10	0.21	24.45	TRUE
CD19	9	134127597	134154499	ENST00000464831.1	FAM78A	0.35	0.11	0.24	0.16	0.30	24.20	TRUE
CD19	1	206730430	206756416	ENST00000304534.8	RASSF5	0.25	0.05	0.19	0.16	0.27	22.59	TRUE
CD19	13	41534432	41594336	ENST00000239882.3	ELF1	0.21	0.08	0.13	0.09	0.31	21.93	TRUE
CD19	12	57869667	57876031	ENST00000552249.1	ARHGAP9	0.56	0.14	0.42	0.26	0.40	21.40	TRUE
CD19	14	61937696	61947119	ENST00000536400.1	PRKCH	0.33	0.04	0.29	0.04	0.29	19.40	TRUE
CD19	7	50344148	50370755	ENST00000426121.1	IKZF1	0.41	0.07	0.35	0.20	0.42	19.07	TRUE
CD19	21	44817042	44836022	ENST00000478426.1	SIK1	0.22	0.05	0.17	0.30	0.58	18.82	TRUE
CD19	11	128582503	128610944	ENST00000534087.1	FLI1	0.28	0.04	0.24	0.14	0.36	17.88	TRUE
CD19	3	122291690	122308644	ENST00000483793.1	PARP15	0.20	0.04	0.16	0.13	0.29	17.63	TRUE
CD19	17	43299369	43312992	ENST00000591434.1	FMNL1	0.55	0.16	0.39	0.17	0.30	17.34	TRUE
CD19	22	37613897	37642449	ENST00000401529.3	RAC2	0.28	0.09	0.18	0.07	0.12	17.11	TRUE
CD19	2	238580170	238610065	ENST00000473815.1	LRRFIP1	0.35	0.19	0.16	0.13	0.24	16.12	TRUE
CD19	20	55964442	55977216	ENST00000371219.2	RBM38	0.53	0.33	0.20	0.15	0.24	15.94	TRUE
CD19	21	45660565	45668530	ENST00000400377.3	ICOSLG	0.72	0.38	0.34	0.08	0.14	15.33	TRUE
CD19	14	105116134	105145145	ENST00000330634.7	INF2	0.23	0.12	0.11	0.14	0.28	15.28	TRUE
CD19	1	26609716	26620076	ENST00000319041.6	SH3BGRL3	0.40	0.03	0.37	0.24	0.41	14.66	TRUE
CD19	1	53096945	53108122	ENST00000424164.1	FAM159A	0.35	0.08	0.27	0.19	0.26	13.94	TRUE
CD19	22	24821855	24835079	ENST00000439591.1	ADORA2A	0.26	0.02	0.25	0.32	0.60	13.11	TRUE
CD19	19	2610475	2632474	ENST00000587867.1	GNG7	0.31	0.09	0.22	0.10	0.17	12.71	TRUE
CD19	13	46942847	46975976	ENST00000480935.1	KIAA0226L	0.24	0.08	0.16	0.07	0.10	12.28	TRUE
CD19	20	4789416	4805261	ENST00000379400.3	RASSF2	0.41	0.18	0.23	0.22	0.36	12.12	TRUE
CD19	4	56812204	56816635	ENST00000257287.4	CEP135	0.73	0.52	0.21	0.11	0.14	12.05	TRUE
CD19	8	56790857	56809092	ENST00000520220.1	LYN	0.35	0.10	0.25	0.13	0.21	11.96	TRUE
CD19	6	159459555	159485569	ENST00000338313.5	TAGAP	0.27	0.05	0.22	0.13	0.25	11.68	TRUE
CD19	17	29814146	29838313	ENST00000578694.1	RAB11FIP4	0.24	0.11	0.13	0.14	0.26	11.44	TRUE
CD19	19	35807029	35840479	ENST00000593704.1	CD22	0.26	0.02	0.24	0.22	0.56	11.25	TRUE
CD19	19	13204800	13217457	ENST00000590120.1	LYL1	0.94	0.34	0.60	0.21	0.44	11.15	TRUE
CD19	11	67034303	67056724	ENST00000447274.2	ANKRD13D	0.40	0.23	0.17	0.12	0.23	10.45	TRUE
CD19	11	73076425	73101853	ENST00000393580.2	RELT	0.21	0.06	0.15	0.13	0.18	10.21	TRUE

CD19	11	9621558	9642085	ENST00000527848.1	WEE1	0.21	0.09	0.12	0.06	0.11	9.86	TRUE
CD19	9	35616119	35651731	ENST00000474403.1	SIT1	0.23	0.07	0.16	0.15	0.23	9.35	TRUE
CD19	15	74666045	74697205	ENST00000268053.6	CYP11A1	0.31	0.12	0.19	0.26	0.34	8.30	TRUE
CD19	1	111734737	111768995	ENST00000445067.2	CHI3L2	0.20	0.06	0.14	0.09	0.12	8.24	TRUE
CD19	6	41671523	41703442	ENST00000433032.1	TFEB	0.33	0.15	0.18	0.18	0.23	8.08	TRUE
CD19	6	32904722	32916498	ENST00000416244.2	HLA-DMB	0.27	0.09	0.18	0.16	0.31	7.98	TRUE
CD19	1	66795175	66818506	ENST00000526197.1	PDE4B	0.28	0.18	0.10	0.13	0.25	7.81	TRUE
CD19	16	85921635	85951991	ENST00000569607.1	IRF8	0.29	0.05	0.24	0.22	0.37	7.79	TRUE
CD19	3	196350844	196371448	ENST00000426755.1	PIGX	0.34	0.14	0.20	0.12	0.18	7.65	TRUE
CD19	9	130665468	130681316	ENST00000479747.1	ST6GALNAC4	0.33	0.18	0.16	0.09	0.13	7.17	TRUE
CD19	6	31547188	31556914	ENST00000464044.1	LST1	0.57	0.18	0.40	0.19	0.25	6.59	TRUE
CD19	6	149785200	149817360	ENST00000462655.1	ZC3H12D	0.28	0.04	0.24	0.17	0.35	6.58	TRUE
CD19	12	46657445	46665336	ENST00000546519.1	SLC38A1	0.57	0.45	0.12	0.10	0.17	6.45	TRUE
CD19	11	58967706	58992697	ENST00000361050.3	MPEG1	0.22	0.02	0.20	0.29	0.50	6.35	TRUE
CD19	1	207078366	207106998	ENST00000487208.1	PIGR	0.23	0.05	0.18	0.20	0.38	6.25	TRUE
CD19	12	48196588	48213374	ENST00000445237.2	HDAC7	0.40	0.23	0.17	0.11	0.15	6.14	TRUE
CD19	2	54783925	54824402	ENST00000333896.5	SPTBN1	0.27	0.15	0.12	0.08	0.13	6.13	TRUE
CD19	2	197013308	197048452	ENST00000449152.1	STK17B	0.42	0.07	0.34	0.07	0.12	5.81	TRUE
CD19	2	71290385	71301563	ENST00000524537.1	NAGK	0.39	0.21	0.18	0.07	0.10	5.74	TRUE
CD19	6	32402294	32411438	ENST00000374982.5	HLA-DRA	0.36	0.10	0.26	0.24	0.36	5.34	TRUE
CD19	19	42374270	42392833	ENST00000337665.4	ARHGEF1	0.44	0.19	0.25	0.15	0.23	5.12	TRUE
CD19	10	49874683	49897623	ENST00000360890.2	WDFY4	0.25	0.00	0.25	0.30	0.59	5.11	TRUE
CD19	17	74476430	74497452	ENST00000590288.1	RHBDF2	0.36	0.13	0.23	0.27	0.38	4.87	TRUE
CD19	1	154914194	154929302	ENST00000490230.1	PBXIP1	0.29	0.16	0.12	0.16	0.21	4.81	TRUE
CD19	20	44736001	44751095	ENST00000461171.1	CD40	0.21	0.09	0.12	0.06	0.15	4.79	TRUE
CD19	3	13034134	13065411	ENST00000473088.1	IQSEC1	0.30	0.15	0.15	0.16	0.23	4.63	TRUE
CD19	11	72850513	72872020	ENST00000422375.1	FCHSD2	0.41	0.17	0.24	0.10	0.13	4.54	TRUE
CD19	19	18278723	18292546	ENST00000600463.1	IFI30	0.23	0.10	0.13	0.15	0.25	4.49	TRUE
CD19	2	136871650	136898787	ENST00000466288.1	CXCR4	0.33	0.14	0.18	0.06	0.08	4.31	TRUE
CD19	11	61120450	61130574	ENST00000423772.2	TMEM138	0.45	0.20	0.25	0.21	0.23	4.29	TRUE
CD19	15	75066062	75093388	ENST00000569321.1	CSK	0.51	0.25	0.27	0.13	0.18	4.20	TRUE
CD19	16	11758829	11785379	ENST00000575349.1	TXNDC11	0.22	0.10	0.12	0.10	0.11	4.16	TRUE
CD19	16	10959821	11016093	ENST00000572665.1	CIITA	0.28	0.07	0.20	0.11	0.23	4.05	TRUE
CD19	15	57571264	57599710	ENST00000560948.1	TCF12	0.24	0.04	0.19	0.16	0.22	4.02	TRUE
CD19	14	106313303	106330974	ENST00000482999.1	KIAA0125	0.51	0.00	0.51	0.31	0.59	4.00	TRUE
CD19	19	2040263	2096653	ENST00000395307.2	IZUMO4	0.32	0.19	0.13	0.18	0.24	3.98	TRUE
CD19	16	81803418	81877366	ENST00000569523.1	PLCG2	0.26	0.02	0.24	0.24	0.26	3.96	TRUE
CD19	8	56751994	56758932	ENST00000519728.1	LYN	0.54	0.24	0.30	0.04	0.08	3.70	TRUE
CD19	2	231729691	231744201	ENST00000492029.1	ITM2C	0.33	0.19	0.14	0.24	0.34	3.70	TRUE

CD19	10	112618797	112633729	ENST00000444997.1	PDCD4	0.35	0.25	0.11	0.08	0.11	3.69	TRUE
CD19	14	105524940	105537562	ENST00000546679.1	GPR132	0.32	0.05	0.27	0.29	0.48	3.56	TRUE
CD19	6	106957303	106996547	ENST00000487681.1	AIM1	0.26	0.13	0.13	0.11	0.17	3.39	TRUE
CD19	1	38937882	38948718	ENST00000474456.1	RRAGC	0.22	0.06	0.16	0.19	0.32	3.28	TRUE
CD19	22	42304034	42337858	ENST00000472374.2	CENPM	0.44	0.22	0.22	0.22	0.28	3.26	TRUE
CD19	2	202106787	202128005	ENST00000429881.1	CASP8	0.21	0.10	0.11	0.15	0.20	3.18	TRUE
CD19	17	27064086	27073896	ENST00000584944.1	TRAF4	0.48	0.35	0.13	0.12	0.13	3.14	TRUE
CD19	12	7044970	7072648	ENST00000537533.1	PTPN6	0.36	0.21	0.15	0.20	0.27	3.12	TRUE
CD19	4	82389749	82394691	ENST00000507538.1	RASGEF1B	0.74	0.59	0.14	0.08	0.09	3.09	TRUE
CD19	12	92527263	92581890	ENST00000552315.1	BTG1	0.30	0.14	0.15	0.07	0.07	3.06	TRUE
CD19	16	28921620	28948628	ENST00000567368.1	CD19	0.20	0.10	0.11	0.20	0.30	2.96	TRUE
CD19	4	185452534	185460044	ENST00000393593.3	IRF2	0.39	0.25	0.14	0.15	0.19	2.90	TRUE
CD19	22	18476769	18494405	ENST00000424046.1	MICAL3	0.32	0.12	0.19	0.18	0.31	2.86	TRUE
CD19	15	86229664	86252170	ENST00000560482.1	AKAP13	0.31	0.05	0.26	0.24	0.38	2.74	TRUE
CD19	17	3807403	3821299	ENST00000571637.1	P2RX1	0.34	0.10	0.24	0.30	0.47	2.74	TRUE
CD19	15	45001549	45023195	ENST00000561237.1	TRIM69	0.36	0.24	0.12	0.09	0.13	2.68	TRUE
CD19	2	46761453	46803094	ENST00000473428.1	RHOQ	0.24	0.09	0.15	0.05	0.06	2.66	TRUE
CD19	12	9788063	9838708	ENST00000544322.1	CLEC2D	0.20	0.06	0.14	0.15	0.17	2.63	TRUE
CD19	6	167362856	167374276	ENST00000496851.2	RNASET2	0.36	0.20	0.16	0.11	0.12	2.16	TRUE
CD19	7	142490667	142511468	ENST00000463701.1	PRSS1	0.26	0.12	0.14	0.12	0.14	2.14	TRUE
CD19	7	86840957	86850205	ENST00000455575.1	TMEM243	0.59	0.31	0.28	0.07	0.08	2.08	TRUE
CD19	1	2477758	2482788	ENST00000426449.1	TNFRSF14	0.72	0.59	0.12	0.11	0.14	2.03	TRUE
CD19	7	150100445	150107156	ENST00000474836.1	ZNF775	0.56	0.35	0.21	0.15	0.21	1.88	TRUE
CD19	4	40289700	40319643	ENST00000310169.2	CHRNA9	0.21	0.06	0.14	0.08	0.15	1.71	TRUE
CD19	8	134509931	134514822	ENST00000520020.1	ST3GAL1	0.60	0.12	0.48	0.25	0.29	1.71	TRUE
CD19	17	75396031	75469717	ENST00000593189.1	SEPT9	0.28	0.15	0.12	0.27	0.31	1.48	TRUE
CD19	11	58340596	58348101	ENST00000389919.4	ZFP91-CNTF	0.83	0.52	0.31	0.07	0.07	1.29	FALSE
CD19	9	3524176	3529109	ENST00000449190.1	RFX3	0.90	0.77	0.13	0.06	0.07	1.25	FALSE
CD19	7	138778458	138805019	ENST00000471652.1	ZC3HAV1	0.29	0.12	0.17	0.10	0.12	1.22	FALSE
CD19	2	232255961	232260646	ENST00000287590.5	B3GNT7	0.54	0.13	0.41	0.32	0.37	1.15	FALSE
CD19	19	50917413	50938549	ENST00000357701.5	MYBPC2	0.42	0.26	0.16	0.22	0.24	1.12	FALSE
CD19	14	23018284	23039986	ENST00000557595.1	AE000662.92	0.25	0.10	0.15	0.05	0.05	1.04	FALSE
CD19	12	110431794	110453837	ENST00000261739.4	ANKRD13A	0.28	0.16	0.12	0.07	0.07	0.71	FALSE
CD19	14	50327610	50337580	ENST00000298310.5	NEMF	0.50	0.38	0.12	0.04	0.04	0.70	FALSE
CD19	3	58318904	58343543	ENST00000491164.1	PXK	0.25	0.07	0.18	0.02	0.03	0.61	FALSE
CD19	17	38004143	38027814	ENST00000377940.3	ZPBP2	0.26	0.05	0.21	0.32	0.34	0.52	FALSE
CD19	8	29937591	29951059	ENST00000521083.1	TMEM66	0.29	0.18	0.11	0.04	0.04	0.15	FALSE
CD19	19	10712789	10716244	ENST00000407327.4	SLC44A2	0.80	0.58	0.21	0.16	0.17	0.14	FALSE
CD19	2	196511447	196525260	ENST00000418005.1	SLC39A10	0.39	0.26	0.13	0.03	0.03	0.00	FALSE

CD19	5	122109910	122117182	ENST00000514949.1	SNX2	0.41	0.31	0.11	0.08	0.07	-0.04	FALSE
CD19	16	15728212	15738278	ENST00000396353.2	NDE1	0.42	0.28	0.14	0.04	0.04	-0.07	FALSE
CD19	19	39887963	39904206	ENST00000438123.1	PLEKHG2	0.70	0.60	0.10	0.08	0.07	-0.13	FALSE
CD19	18	2634081	2658907	ENST00000261598.8	SMCHD1	0.25	0.15	0.10	0.10	0.09	-0.24	FALSE
CD19	22	31680149	31688920	ENST00000443175.1	PIK3IP1	0.44	0.33	0.11	0.08	0.08	-0.27	FALSE
CD19	17	38473833	38487686	ENST00000582914.1	RARA	0.58	0.44	0.14	0.16	0.15	-0.31	FALSE
CD19	11	122930787	122942297	ENST00000531063.1	HSPA8	0.53	0.34	0.19	0.15	0.15	-0.44	FALSE
CD19	11	82763568	82785588	ENST00000533276.1	RAB30	0.31	0.16	0.15	0.05	0.05	-0.74	FALSE
CD19	2	128144112	128148979	ENST00000409179.2	MAP3K2	0.62	0.45	0.17	0.14	0.12	-0.84	FALSE
CD19	14	64966362	64978672	ENST00000555321.1	ZBTB1	0.55	0.37	0.18	0.11	0.09	-1.91	FALSE
CD19	1	40838755	40871047	ENST00000539317.1	SMAP2	0.22	0.08	0.14	0.10	0.07	-2.81	FALSE
CD19	1	27984903	27990153	ENST00000339145.4	IFI6	0.51	0.32	0.18	0.14	0.10	-3.04	FALSE
Colon	5	79542438	79554102	ENST00000513907.1	SERINC5	0.51	0.26	0.25	0.13	0.18	14.54	TRUE
Colon	10	90638872	90664402	ENST00000371924.1	STAMBPL1	0.20	0.07	0.13	0.09	0.13	13.17	TRUE
Colon	15	75072089	75083506	ENST00000567571.1	CSK	0.80	0.52	0.28	0.08	0.11	10.57	TRUE
Colon	10	134259441	134282106	ENST00000392630.3	C10orf91	0.26	0.12	0.14	0.26	0.32	9.61	TRUE
Colon	1	37936771	37953471	ENST00000471012.1	ZC3H12A	0.49	0.34	0.15	0.16	0.19	9.15	TRUE
Colon	19	1256417	1263739	ENST00000589161.1	CIRBP	0.65	0.52	0.13	0.15	0.18	8.78	TRUE
Colon	19	1852750	1865843	ENST00000592313.1	KLF16	0.63	0.51	0.12	0.07	0.09	8.29	TRUE
Colon	3	49056900	49060957	ENST00000480392.1	NDUFAF3	0.84	0.67	0.17	0.10	0.11	8.01	TRUE
Colon	17	27275334	27280852	ENST00000577182.1	PIPOX	1.00	0.86	0.14	0.08	0.09	7.92	TRUE
Colon	16	2028149	2036628	ENST00000567719.1	GFER	0.59	0.43	0.16	0.16	0.19	7.14	TRUE
Colon	11	130013608	130061419	ENST00000530376.1	ST14	0.22	0.05	0.16	0.21	0.26	7.07	TRUE
Colon	6	33538390	33559780	ENST00000374458.1	GGNBP1	0.27	0.16	0.12	0.13	0.15	6.84	TRUE
Colon	2	220109452	220119946	ENST00000392088.2	TUBA4A	0.53	0.40	0.12	0.07	0.09	6.77	TRUE
Colon	9	140187219	140215940	ENST00000356628.2	NRARP	0.53	0.40	0.13	0.24	0.29	5.89	TRUE
Colon	7	150052314	150083105	ENST00000486297.1	ZNF775	0.36	0.21	0.15	0.14	0.16	5.67	TRUE
Colon	14	50465553	50471375	ENST00000529902.1	C14orf182	0.66	0.45	0.22	0.11	0.12	5.40	TRUE
Colon	6	74224863	74234116	ENST00000455918.1	EEF1A1	0.90	0.58	0.32	0.10	0.12	5.13	TRUE
Colon	6	31700974	31708664	ENST00000493662.2	MSH5-SAPCD1	0.87	0.70	0.18	0.11	0.12	4.92	TRUE
Colon	1	1092807	1104591	ENST00000506177.1	TTLL10	0.35	0.19	0.16	0.29	0.34	4.42	TRUE
Colon	7	1486269	1507327	ENST00000297508.7	MICALL2	0.35	0.23	0.12	0.09	0.11	4.22	TRUE
Colon	17	4845115	4854686	ENST00000519300.1	ENO3	0.72	0.58	0.13	0.11	0.12	3.71	TRUE
Colon	16	29830344	29838029	ENST00000570234.1	MVP	0.48	0.28	0.19	0.28	0.35	3.49	TRUE
Colon	1	153503987	153511480	ENST00000462951.2	S100A6	0.39	0.26	0.13	0.07	0.09	3.38	TRUE
Colon	4	102266379	102270939	ENST00000529296.1	AP001816.1	0.94	0.80	0.14	0.05	0.06	3.03	TRUE
Colon	7	100608082	100627662	ENST00000536621.1	MUC12	0.30	0.20	0.11	0.27	0.29	2.91	TRUE
Colon	1	159888888	159896553	ENST00000397334.2	TAGLN2	0.63	0.45	0.18	0.09	0.10	2.80	TRUE
Colon	1	149819766	149826428	ENST00000403683.1	HIST2H3A	0.28	0.14	0.14	0.07	0.08	2.76	TRUE

Colon	7	27202748	27210249	ENST00000489695.1	HOXA9	0.95	0.85	0.10	0.19	0.23	2.73	TRUE
Colon	7	27210777	27221249	ENST00000396344.4	HOXA10	0.76	0.45	0.30	0.26	0.29	2.60	TRUE
Colon	19	13948788	13956320	ENST00000591727.1	NANOS3	0.69	0.55	0.15	0.24	0.29	2.55	TRUE
Colon	7	104643451	104656449	ENST00000474203.1	MLL5	0.62	0.51	0.12	0.06	0.07	2.40	TRUE
Colon	1	150531037	150543263	ENST00000369035.2	C1orf138	0.51	0.37	0.14	0.15	0.16	2.35	TRUE
Colon	1	19966426	19978402	ENST00000427894.1	NBL1	0.54	0.25	0.28	0.15	0.17	2.18	TRUE
Colon	5	180668526	180674519	ENST00000514318.1	GNB2L1	0.82	0.70	0.13	0.07	0.08	2.01	TRUE
Colon	17	57904024	57931778	ENST00000587470.1	VMP1	0.49	0.14	0.35	0.25	0.33	1.96	TRUE
Colon	11	118659296	118664159	ENST00000533239.1	DDX6	1.00	0.86	0.14	0.05	0.06	1.96	TRUE
Colon	12	7051408	7057319	ENST00000538318.1	PTPN6	0.59	0.40	0.18	0.14	0.14	1.93	TRUE
Colon	20	52195018	52241000	ENST00000540425.1	ZNF217	0.41	0.24	0.17	0.22	0.23	1.74	TRUE
Colon	6	32934747	32951807	ENST00000482838.1	BRD2	0.61	0.51	0.10	0.05	0.06	1.58	TRUE
Colon	17	7161661	7168259	ENST00000573745.1	CLDN7	0.63	0.45	0.18	0.12	0.13	1.40	TRUE
Colon	15	86121044	86133400	ENST00000560340.1	AKAP13	0.40	0.18	0.22	0.20	0.26	1.38	TRUE
Colon	2	201980044	201998492	ENST00000460961.1	CFLAR	0.57	0.44	0.13	0.12	0.13	1.25	FALSE
Colon	2	174828021	174831622	ENST00000490182.1	SP3	1.00	0.88	0.12	0.07	0.07	1.19	FALSE
Colon	17	4033794	4049258	ENST00000573984.1	CYB5D2	0.27	0.16	0.11	0.07	0.07	1.17	FALSE
Colon	20	43968274	43977802	ENST00000537976.1	SDC4	0.77	0.29	0.48	0.18	0.19	1.08	FALSE
Colon	2	173291273	173330716	ENST00000409080.1	ITGA6	0.25	0.10	0.15	0.17	0.18	0.90	FALSE
Colon	3	9436663	9444883	ENST00000406341.1	SETD5	0.78	0.65	0.13	0.09	0.10	0.86	FALSE
Colon	5	96268784	96273156	ENST00000231368.5	LNPEP	0.95	0.81	0.13	0.03	0.03	0.76	FALSE
Colon	8	103816742	103826153	ENST00000518697.1	AZIN1	0.54	0.43	0.12	0.11	0.11	0.75	FALSE
Colon	8	126438172	126449618	ENST00000519576.1	TRIB1	0.52	0.36	0.16	0.06	0.05	0.64	FALSE
Colon	17	38213719	38232994	ENST00000577486.1	THRA	0.55	0.44	0.11	0.15	0.16	0.64	FALSE
Colon	18	3446352	3459783	ENST00000472042.1	TGIF1	0.77	0.55	0.22	0.09	0.09	0.62	FALSE
Colon	1	1675407	1679081	ENST00000246421.4	SLC35E2	0.74	0.62	0.12	0.03	0.04	0.54	FALSE
Colon	3	193848397	193860794	ENST00000476918.1	HES1	0.80	0.61	0.19	0.08	0.08	0.47	FALSE
Colon	2	200306225	200342782	ENST00000463386.1	SATB2	0.56	0.40	0.16	0.16	0.17	0.46	FALSE
Colon	17	7459695	7466311	ENST00000429205.2	SENP3	0.74	0.63	0.11	0.20	0.19	0.26	FALSE
Colon	1	169069278	169085972	ENST00000367813.3	ATP1B1	0.45	0.34	0.11	0.13	0.12	0.19	FALSE
Colon	12	6559071	6563067	ENST00000543567.1	TAPBPL	0.75	0.64	0.11	0.06	0.05	-0.04	FALSE
Colon	12	56472347	56482109	ENST00000546748.1	ERBB3	0.44	0.27	0.17	0.15	0.14	-0.05	FALSE
Colon	11	307666	312937	ENST00000399815.2	IFITM2	0.70	0.55	0.14	0.16	0.16	-0.08	FALSE
Colon	3	50373024	50380264	ENST00000490675.1	ZMYND10	0.49	0.39	0.10	0.16	0.14	-0.13	FALSE
Colon	13	28526778	28556061	ENST00000548877.1	CDX2	0.94	0.72	0.23	0.23	0.23	-0.27	FALSE
Colon	2	151323963	151344439	ENST00000454202.1	RND3	0.28	0.17	0.11	0.12	0.11	-0.59	FALSE
Colon	4	38662929	38689928	ENST00000436901.1	AC021860.1	0.45	0.29	0.16	0.14	0.13	-0.59	FALSE
Colon	19	49463688	49472130	ENST00000331825.6	FTL	0.54	0.39	0.15	0.19	0.18	-0.82	FALSE
Colon	5	179244886	179250297	ENST00000360718.5	SQSTM1	0.68	0.57	0.11	0.07	0.06	-1.19	FALSE

Colon	11	71748625	71754435	ENST00000535947.1	NUMA1	0.81	0.67	0.13	0.14	0.12	-1.54	FALSE
Colon	12	53762866	53777584	ENST00000548560.1	SP1	0.41	0.29	0.12	0.11	0.10	-1.97	FALSE
Colon	11	64654606	64662582	ENST00000457202.1	EHD1	0.23	0.09	0.13	0.14	0.12	-5.94	FALSE
Lung	4	174427206	174460857	ENST00000505300.1	HAND2	0.74	0.60	0.14	0.24	0.31	9.72	TRUE
Lung	2	66659175	66674430	ENST00000475239.1	MEIS1	0.78	0.63	0.16	0.20	0.28	9.54	TRUE
Lung	1	2160113	2167961	ENST00000508416.1	SKI	0.66	0.55	0.12	0.17	0.26	5.43	TRUE
Lung	20	22536539	22565230	ENST00000319993.4	FOXA2	0.69	0.54	0.15	0.17	0.22	2.36	TRUE
Lung	16	56638742	56646907	ENST00000567300.1	MT2A	0.50	0.39	0.12	0.22	0.27	2.26	TRUE
Lung	12	6441703	6453465	ENST00000538363.1	TNFRSF1A	0.61	0.45	0.16	0.05	0.08	1.37	TRUE
Lung	12	119613625	119628282	ENST00000542496.1	HSPB8	0.22	0.10	0.12	0.14	0.19	1.36	TRUE
Lung	11	305282	312843	ENST00000399815.2	IFITM2	0.50	0.40	0.10	0.16	0.17	1.10	FALSE
Lung	1	154939647	154948901	ENST00000473344.1	CKS1B	0.61	0.48	0.12	0.15	0.17	0.69	FALSE
Lung	22	46464333	46478321	ENST00000443490.1	MIRLET7	0.85	0.71	0.15	0.20	0.23	0.32	FALSE
Lung	11	67803279	67809641	ENST00000533947.1	TCIRG1	0.45	0.34	0.11	0.10	0.12	0.29	FALSE
Lung	17	7378645	7384073	ENST00000380599.4	ZBTB4	0.65	0.45	0.20	0.22	0.24	0.06	FALSE
Lung	5	43033834	43045550	ENST00000314890.3	ANXA2R	0.57	0.43	0.14	0.20	0.21	-0.09	FALSE
Lung	19	45244285	45262369	ENST00000473473.1	BCL3	0.46	0.34	0.12	0.08	0.09	-0.21	FALSE
Lung	12	92526891	92540701	ENST00000552315.1	BTG1	0.71	0.57	0.14	0.12	0.11	-0.21	FALSE
Lung	14	105938037	105944737	ENST00000548309.1	CRIP2	0.80	0.66	0.14	0.16	0.17	-0.32	FALSE
Lung	1	145434374	145443479	ENST00000486597.1	TXNIP	0.71	0.57	0.15	0.10	0.10	-0.61	FALSE
Lung	19	13948720	13956318	ENST00000591727.1	NANOS3	0.66	0.55	0.11	0.27	0.28	-1.91	FALSE
Lung	9	35726029	35733751	ENST00000486056.1	CREB3	0.65	0.53	0.12	0.13	0.12	-2.30	FALSE

Supplementary Table 4: Hypermethylated super-enhancers in cancer samples (δ HMR > 25%).

	Super-Enhancer			HMR coverage (%)			
**Cancer type**	**Chr.**	**Start**	**End**	**Normal**	**Cancer**	**δ**	**Gene symbol**
Primary breast tumor (468PT)	7	27134577	27144793	0.95	0.22	0.73	HOXA2
Primary breast tumor (468PT)	5	1881509	1890183	0.97	0.29	0.67	IRX4
Primary breast tumor (468PT)	3	29321550	29334286	0.65	0.00	0.65	RBMS3
Primary breast tumor (468PT)	5	142773722	142785691	0.95	0.32	0.63	NR3C1
Primary breast tumor (468PT)	11	8828385	8837469	0.63	0.00	0.63	ST5
Primary breast tumor (468PT)	22	46467114	46488203	1.00	0.39	0.61	MIRLET7
Primary breast tumor (468PT)	5	2739262	2759651	0.92	0.31	0.60	IRX2
Primary breast tumor (468PT)	5	131593100	131603174	0.60	0.00	0.60	PDLIM4
Primary breast tumor (468PT)	22	46448058	46467049	1.00	0.50	0.50	MIRLET7
Primary breast tumor (468PT)	7	27175056	27185979	0.61	0.17	0.44	HOXA5
Primary breast tumor (468PT)	21	36252652	36264673	0.93	0.50	0.42	RUNX1
Primary breast tumor (468PT)	1	45270631	45276266	0.65	0.25	0.41	BTBD19
Primary breast tumor (468PT)	1	33792562	33815439	0.49	0.10	0.39	PHC2
Primary breast tumor (468PT)	10	122913683	122920721	0.38	0.00	0.38	WDR11
Primary breast tumor (468PT)	20	51581498	51597746	0.45	0.08	0.37	TSHZ2
Primary breast tumor (468PT)	14	74207241	74227902	0.58	0.22	0.36	MIR4505
Primary breast tumor (468PT)	12	92527863	92539838	0.63	0.28	0.35	BTG1
Primary breast tumor (468PT)	1	154941228	154949531	0.82	0.49	0.34	MIR4258
Primary breast tumor (468PT)	7	41734099	41745805	0.40	0.07	0.34	INHBA
Primary breast tumor (468PT)	11	6339234	6344196	0.61	0.28	0.32	PRKCDBP
Primary breast tumor (468PT)	1	144706472	144710218	0.32	0.00	0.32	PDE4DIP
Primary breast tumor (468PT)	1	27830256	27900464	0.37	0.05	0.32	WASF2
Primary breast tumor (468PT)	15	93425946	93432559	0.87	0.56	0.31	CHD2
Primary breast tumor (468PT)	20	34326330	34331841	0.91	0.61	0.30	RBM39
Primary breast tumor (468PT)	15	50644604	50649357	0.96	0.66	0.29	GABPB1
Primary breast tumor (468PT)	12	6432600	6452223	0.50	0.21	0.29	TNFRSF1A
Primary breast tumor (468PT)	3	71104592	71120542	0.41	0.12	0.28	FOXP1
Primary breast tumor (468PT)	1	157964217	157991843	0.50	0.22	0.28	KIRREL
Primary breast tumor (468PT)	1	144981791	144985538	0.28	0.00	0.28	PDE4DIP
Primary breast tumor (468PT)	14	69402525	69445588	0.35	0.08	0.28	ACTN1
Primary breast tumor (468PT)	19	42771661	42788152	0.57	0.30	0.27	ERF
Primary breast tumor (468PT)	7	74264047	74268456	0.26	0.00	0.26	GTF2IRD2
Primary breast tumor (468PT)	10	126406768	126435377	0.34	0.08	0.26	FAM53B
Breast tumor metastasis (468LN)	5	2739262	2759651	0.92	0.13	0.79	IRX2

Breast tumor metastasis (468LN)	5	1881509	1890183	0.97	0.21	0.76	IRX4
Breast tumor metastasis (468LN)	7	27134577	27144793	0.95	0.21	0.74	HOXA2
Breast tumor metastasis (468LN)	1	45270631	45276266	0.65	0.00	0.65	BTBD19
Breast tumor metastasis (468LN)	5	131593100	131603174	0.60	0.00	0.60	PDLIM4
Breast tumor metastasis (468LN)	2	239193425	239200323	0.73	0.15	0.59	PER2
Breast tumor metastasis (468LN)	12	6479020	6487672	0.57	0.00	0.57	LTBR
Breast tumor metastasis (468LN)	9	33157786	33167403	0.92	0.36	0.56	B4GALT1
Breast tumor metastasis (468LN)	18	3622231	3626476	0.52	0.00	0.52	DLGAP1
Breast tumor metastasis (468LN)	16	54959639	54972666	0.93	0.43	0.51	IRX5
Breast tumor metastasis (468LN)	7	27175056	27185979	0.61	0.11	0.49	HOXA5
Breast tumor metastasis (468LN)	7	5457613	5470399	0.98	0.49	0.49	TNRC18
Breast tumor metastasis (468LN)	4	146653244	146658422	0.68	0.21	0.47	MMAA
Breast tumor metastasis (468LN)	3	193849125	193860857	0.79	0.34	0.46	HES1
Breast tumor metastasis (468LN)	12	10868156	10877012	0.79	0.35	0.44	CSDA
Breast tumor metastasis (468LN)	1	153579895	153590703	0.49	0.06	0.43	S100A14
Breast tumor metastasis (468LN)	1	59245181	59252471	0.86	0.44	0.43	JUN
Breast tumor metastasis (468LN)	11	62306073	62328249	0.61	0.20	0.42	AHNAK
Breast tumor metastasis (468LN)	3	71104592	71120542	0.41	0.00	0.41	FOXP1
Breast tumor metastasis (468LN)	10	122913683	122920721	0.38	0.00	0.38	WDR11
Breast tumor metastasis (468LN)	6	121756401	121769000	0.38	0.00	0.38	GJA1
Breast tumor metastasis (468LN)	4	128702346	128706700	0.55	0.17	0.38	HSPA4L
Breast tumor metastasis (468LN)	20	10634437	10656669	0.49	0.11	0.37	JAG1
Breast tumor metastasis (468LN)	6	35692791	35701120	0.55	0.18	0.37	FKBP5
Breast tumor metastasis (468LN)	22	46448058	46467049	1.00	0.63	0.37	MIRLET7
Breast tumor metastasis (468LN)	11	8828385	8837469	0.63	0.26	0.37	ST5
Breast tumor metastasis (468LN)	3	29321550	29334286	0.65	0.29	0.36	RBMS3
Breast tumor metastasis (468LN)	19	13123147	13172690	0.50	0.14	0.36	TRMT1
Breast tumor metastasis (468LN)	14	77489392	77513614	0.67	0.31	0.35	C14orf4
Breast tumor metastasis (468LN)	1	27830256	27900464	0.37	0.02	0.35	WASF2
Breast tumor metastasis (468LN)	12	115105999	115123428	0.60	0.24	0.35	TBX3
Breast tumor metastasis (468LN)	11	66821179	66830989	0.33	0.00	0.33	RHOD
Breast tumor metastasis (468LN)	11	65238595	65276583	0.63	0.31	0.32	MALAT1
Breast tumor metastasis (468LN)	1	144706472	144710218	0.32	0.00	0.32	PDE4DIP
Breast tumor metastasis (468LN)	15	93425946	93432559	0.87	0.56	0.31	CHD2
Breast tumor metastasis (468LN)	7	55085115	55095372	0.50	0.19	0.31	EGFR
Breast tumor metastasis (468LN)	1	37936765	37953189	0.42	0.11	0.31	ZC3H12A
Breast tumor metastasis (468LN)	4	77506454	77513253	0.31	0.00	0.31	STBD1
Breast tumor metastasis (468LN)	7	41734099	41745805	0.40	0.10	0.31	INHBA
Breast tumor metastasis (468LN)	8	145008274	145031982	0.62	0.32	0.30	PLEC1

Breast tumor metastasis (468LN)	17	75274996	75288850	0.30	0.01	0.29	SEPT9
Breast tumor metastasis (468LN)	6	10398835	10421638	0.81	0.52	0.29	TFAP2A
Breast tumor metastasis (468LN)	12	6432600	6452223	0.50	0.21	0.29	TNFRSF1A
Breast tumor metastasis (468LN)	1	144981791	144985538	0.28	0.00	0.28	PDE4DIP
Breast tumor metastasis (468LN)	10	74051120	74097380	0.27	0.00	0.27	DDIT4
Breast tumor metastasis (468LN)	22	36718824	36814131	0.34	0.07	0.27	MYH9
Breast tumor metastasis (468LN)	8	116659885	116682409	0.42	0.15	0.27	TRPS1
Breast tumor metastasis (468LN)	6	30843042	30858880	0.32	0.05	0.27	DDR1
Breast tumor metastasis (468LN)	5	95291493	95300367	0.41	0.14	0.27	ELL2
Breast tumor metastasis (468LN)	5	121515651	121520476	0.26	0.00	0.26	ZNF474
Breast tumor metastasis (468LN)	3	187453456	187468930	0.81	0.56	0.26	BCL6
Breast tumor metastasis (468LN)	2	36579464	36605312	0.55	0.29	0.26	CRIM1
Breast tumor metastasis (468LN)	12	46653450	46664706	0.32	0.07	0.25	SLC38A1
Breast tumor metastasis (468LN)	19	12888234	12905680	0.68	0.43	0.25	JUNB
Lung adenocarcinoma (H1437)	5	2738590	2759482	0.83	0.10	0.74	IRX2
Lung adenocarcinoma (H1437)	5	172655376	172676296	0.74	0.17	0.58	NKX2-5
Lung adenocarcinoma (H1437)	3	128199015	128216666	0.79	0.25	0.53	GATA2
Lung adenocarcinoma (H1437)	4	174427206	174460857	0.74	0.25	0.48	HAND2
Lung adenocarcinoma (H1437)	1	145434374	145443479	0.71	0.24	0.48	TXNIP
Lung adenocarcinoma (H1437)	2	66659175	66674430	0.78	0.33	0.45	MEIS1
Lung adenocarcinoma (H1437)	19	13122379	13134630	0.43	0.04	0.39	NFIX
Lung adenocarcinoma (H1437)	1	145448015	145457936	0.37	0.00	0.37	TXNIP
Lung adenocarcinoma (H1437)	17	7378645	7384073	0.65	0.29	0.36	ZBTB4
Lung adenocarcinoma (H1437)	14	105938037	105944737	0.80	0.46	0.34	CRIP2
Lung adenocarcinoma (H1437)	9	35726029	35733751	0.65	0.34	0.31	CREB3
Lung adenocarcinoma (H1437)	22	46445515	46454339	0.63	0.34	0.29	MIRLET7
Lung adenocarcinoma (H1437)	22	46464333	46478321	0.85	0.58	0.27	MIRLET7
Lung adenocarcinoma (H1437)	10	77154286	77170840	0.72	0.45	0.26	ZNF503
Lung squamous cell carcinoma (H157)	4	174427206	174460857	0.74	0.07	0.67	HAND2
Lung squamous cell carcinoma (H157)	5	2738590	2759482	0.83	0.20	0.63	IRX2
Lung squamous cell carcinoma (H157)	3	128199015	128216666	0.79	0.16	0.63	GATA2
Lung squamous cell carcinoma (H157)	2	8815972	8826211	0.96	0.37	0.59	ID2
Lung squamous cell carcinoma (H157)	2	66659175	66674430	0.78	0.26	0.52	MEIS1
Lung squamous cell carcinoma (H157)	14	36971679	36995027	1.00	0.51	0.49	SFTA3
Lung squamous cell carcinoma (H157)	1	145434374	145443479	0.71	0.23	0.48	TXNIP
Lung squamous cell carcinoma (H157)	12	92526891	92540701	0.71	0.27	0.43	BTG1
Lung squamous cell carcinoma (H157)	19	13122379	13134630	0.43	0.00	0.43	NFIX
Lung squamous cell carcinoma (H157)	12	6441703	6453465	0.61	0.23	0.39	TNFRSF1A
Lung squamous cell carcinoma (H157)	11	118659939	118664216	0.92	0.54	0.38	DDX6

Lung squamous cell carcinoma (H157)	10	77154286	77170840	0.72	0.33	0.38	ZNF503
Lung squamous cell carcinoma (H157)	17	7378645	7384073	0.65	0.28	0.37	ZBTB4
Lung squamous cell carcinoma (H157)	1	145448015	145457936	0.37	0.00	0.37	TXNIP
Lung squamous cell carcinoma (H157)	12	115105978	115131359	0.52	0.15	0.36	TBX3
Lung squamous cell carcinoma (H157)	2	201724772	201732443	0.74	0.39	0.35	Y_RNA
Lung squamous cell carcinoma (H157)	17	38222943	38234141	0.48	0.13	0.35	THRA
Lung squamous cell carcinoma (H157)	12	7032586	7039696	0.64	0.30	0.34	ATN1
Lung squamous cell carcinoma (H157)	9	35726029	35733751	0.65	0.33	0.32	CREB3
Lung squamous cell carcinoma (H157)	22	46464333	46478321	0.85	0.53	0.32	MIRLET7
Lung squamous cell carcinoma (H157)	16	54956715	54973307	0.73	0.41	0.32	IRX5
Lung squamous cell carcinoma (H157)	14	105938037	105944737	0.80	0.48	0.32	CRIP2
Lung squamous cell carcinoma (H157)	17	7459853	7466333	0.71	0.44	0.26	SENP3
Lung squamous cell carcinoma (H157)	22	46445515	46454339	0.63	0.37	0.26	MIRLET7
Lung squamous cell carcinoma (H157)	5	42990572	43020949	0.39	0.13	0.26	ANXA2R
Lung squamous cell carcinoma (H157)	3	48504418	48515502	0.26	0.00	0.26	SHISA5
Small cell lung cancer (H1672)	11	65182856	65196355	0.89	0.13	0.76	NEAT1
Small cell lung cancer (H1672)	14	36971679	36995027	1.00	0.31	0.69	SFTA3
Small cell lung cancer (H1672)	12	6441703	6453465	0.61	0.00	0.61	TNFRSF1A
Small cell lung cancer (H1672)	19	13948720	13956318	0.66	0.13	0.52	MIR23
Small cell lung cancer (H1672)	20	22536539	22565230	0.69	0.17	0.51	FOXA2
Small cell lung cancer (H1672)	4	174427206	174460857	0.74	0.23	0.50	HAND2
Small cell lung cancer (H1672)	3	128199015	128216666	0.79	0.29	0.50	GATA2
Small cell lung cancer (H1672)	17	38222943	38234141	0.48	0.00	0.48	THRA
Small cell lung cancer (H1672)	22	46464333	46478321	0.85	0.38	0.47	MIRLET7
Small cell lung cancer (H1672)	14	105938037	105944737	0.80	0.33	0.47	CRIP2
Small cell lung cancer (H1672)	11	118777304	118801687	0.71	0.25	0.46	UPK2
Small cell lung cancer (H1672)	2	66659175	66674430	0.78	0.33	0.45	MEIS1
Small cell lung cancer (H1672)	22	46445515	46454339	0.63	0.19	0.45	MIRLET7
Small cell lung cancer (H1672)	12	114806250	114852976	0.45	0.02	0.43	TBX5
Small cell lung cancer (H1672)	16	54956715	54973307	0.73	0.33	0.40	IRX5
Small cell lung cancer (H1672)	6	30646634	30659820	0.63	0.23	0.39	NRM
Small cell lung cancer (H1672)	12	92526891	92540701	0.71	0.33	0.38	BTG1
Small cell lung cancer (H1672)	11	67803279	67809641	0.45	0.09	0.37	TCIRG1
Small cell lung cancer (H1672)	1	145448015	145457936	0.37	0.00	0.37	TXNIP
Small cell lung cancer (H1672)	9	35726029	35733751	0.65	0.29	0.36	CREB3
Small cell lung cancer (H1672)	17	7378645	7384073	0.65	0.29	0.36	ZBTB4
Small cell lung cancer (H1672)	17	79475627	79487805	0.63	0.28	0.36	ACTG1
Small cell lung cancer (H1672)	5	2738590	2759482	0.83	0.48	0.35	IRX2
Small cell lung cancer (H1672)	11	118659939	118664216	0.92	0.57	0.35	DDX6

Small cell lung cancer (H1672)	2	201724772	201732443	0.74	0.40	0.35	Y_RNA
Small cell lung cancer (H1672)	19	1256391	1263727	0.50	0.16	0.34	CIRBP
Small cell lung cancer (H1672)	19	13122379	13134630	0.43	0.10	0.34	NFIX
Small cell lung cancer (H1672)	1	153537691	153541885	0.41	0.10	0.32	S100A2
Small cell lung cancer (H1672)	1	1364851	1372426	0.46	0.14	0.32	VWA1
Small cell lung cancer (H1672)	18	3591992	3606653	0.46	0.15	0.31	DLGAP1
Small cell lung cancer (H1672)	22	38708087	38715695	0.57	0.27	0.31	CSNK1E
Small cell lung cancer (H1672)	6	31694582	31706840	0.45	0.14	0.30	CLIC1
Small cell lung cancer (H1672)	19	1247145	1256325	0.68	0.38	0.30	MIDN
Small cell lung cancer (H1672)	19	41219787	41228823	0.64	0.35	0.29	ITPKC
Small cell lung cancer (H1672)	10	77154286	77170840	0.72	0.43	0.29	ZNF503
Small cell lung cancer (H1672)	1	145434374	145443479	0.71	0.42	0.29	TXNIP
Small cell lung cancer (H1672)	7	1569896	1580027	0.40	0.11	0.28	MAFK
Small cell lung cancer (H1672)	9	14302438	14323420	0.55	0.27	0.28	NFIB
Small cell lung cancer (H1672)	12	115105978	115131359	0.52	0.24	0.28	TBX3
Small cell lung cancer (H1672)	10	88726022	88733482	0.37	0.10	0.27	AGAP11
Small cell lung cancer (H1672)	5	172655376	172676296	0.74	0.48	0.26	NKX2-5
Small cell lung cancer (H1672)	16	56638742	56646907	0.50	0.25	0.25	MT2A
Small cell lung cancer (H1672)	9	14340900	14353877	0.32	0.06	0.25	NFIB
Primary colon tumor (Colon_P))	17	46631233	46657163	0.43	0.09	0.34	HOXB3
Primary colon tumor (Colon_P)	7	27178057	27189568	0.69	0.39	0.30	HOXA6
Primary colon tumor (Colon_P)	5	134361067	134376085	0.64	0.36	0.29	PITX1
Primary colon metastasis (Colon_M)	7	27178057	27189568	0.69	0.36	0.33	HOXA6
Primary colon metastasis (Colon_M)	17	46631233	46657163	0.43	0.11	0.32	HOXB3
Primary colon metastasis (Colon_M)	5	134361067	134376085	0.64	0.36	0.28	PITX1
Primary colon metastasis (Colon_M)	15	75072089	75083506	0.80	0.53	0.27	CSK
Glioblastoma (U87MG)	1	161160323	161173936	0.80	0.07	0.73	NDUFS2
Glioblastoma (U87MG)	6	163815822	163882069	0.79	0.14	0.65	QKI
Glioblastoma (U87MG)	7	29516606	29530680	0.63	0.00	0.63	CHN2
Glioblastoma (U87MG)	13	78291692	78329377	0.59	0.00	0.59	SLAIN1
Glioblastoma (U87MG)	9	130697475	130743184	0.61	0.07	0.55	FAM102A
Glioblastoma (U87MG)	18	74684916	74780601	0.53	0.00	0.53	MBP
Glioblastoma (U87MG)	17	79097124	79111085	0.59	0.09	0.50	AATK
Glioblastoma (U87MG)	9	131142171	131157912	0.49	0.00	0.49	MIR219-2
Glioblastoma (U87MG)	11	61520065	61534843	0.53	0.04	0.49	MYRF
Glioblastoma (U87MG)	10	14577374	14624047	0.46	0.00	0.46	FAM107B
Glioblastoma (U87MG)	3	33685210	33704614	0.45	0.00	0.45	CLASP2
Glioblastoma (U87MG)	13	67782754	67806323	0.59	0.14	0.45	PCDH9
Glioblastoma (U87MG)	22	38361166	38471689	0.39	0.01	0.39	SOX10

Glioblastoma (U87MG)	17	36570707	36622668	0.54	0.16	0.39	AC124789.1
Glioblastoma (U87MG)	6	134488737	134505092	0.60	0.22	0.38	SGK1
Glioblastoma (U87MG)	11	67173218	67188540	0.45	0.06	0.38	CARNS1
Glioblastoma (U87MG)	10	81137824	81212605	0.43	0.05	0.38	ZCCHC24
Glioblastoma (U87MG)	8	26427962	26519962	0.45	0.08	0.37	DPYSL2
Glioblastoma (U87MG)	7	22212818	22261220	0.37	0.00	0.37	RAPGEF5
Glioblastoma (U87MG)	3	181403893	181455295	0.84	0.47	0.36	SOX2
Glioblastoma (U87MG)	17	38216903	38234316	0.63	0.27	0.36	THRA
Glioblastoma (U87MG)	18	60417206	60470491	0.35	0.00	0.35	PHLPP1
Glioblastoma (U87MG)	12	48167031	48182319	0.40	0.06	0.35	AC004466.1
Glioblastoma (U87MG)	9	140081775	140094696	0.56	0.21	0.35	TPRN
Glioblastoma (U87MG)	3	170135527	170167574	0.34	0.00	0.34	CLDN11
Glioblastoma (U87MG)	3	72424289	72464834	0.34	0.00	0.34	RYBP
Glioblastoma (U87MG)	2	145129528	145283099	0.39	0.05	0.34	ZEB2-AS1
Glioblastoma (U87MG)	10	88421359	88444547	0.33	0.00	0.33	LDB3
Glioblastoma (U87MG)	5	36599886	36615091	0.50	0.17	0.33	SLC1A3
Glioblastoma (U87MG)	22	37940770	37968154	0.50	0.18	0.32	CDC42EP1
Glioblastoma (U87MG)	2	127806748	127912517	0.36	0.04	0.32	BIN1
Glioblastoma (U87MG)	4	154142334	154219000	0.38	0.06	0.32	Y_RNA
Glioblastoma (U87MG)	6	163659272	163687676	0.36	0.04	0.32	snoU13
Glioblastoma (U87MG)	19	13093539	13211813	0.47	0.16	0.31	NFIX
Glioblastoma (U87MG)	1	202090299	202115217	0.39	0.08	0.31	ARL8A
Glioblastoma (U87MG)	16	19841311	19898686	0.35	0.04	0.31	GPRC5B
Glioblastoma (U87MG)	14	29226576	29255674	0.61	0.30	0.30	RP11-966I7.2
Glioblastoma (U87MG)	10	126400866	126432679	0.40	0.10	0.30	FAM53B
Glioblastoma (U87MG)	17	17638844	17657613	0.30	0.00	0.30	RAI1-AS1
Glioblastoma (U87MG)	15	64974641	64996542	0.36	0.06	0.30	RP11-330L19.4
Glioblastoma (U87MG)	16	75266933	75302422	0.51	0.22	0.29	BCAR1
Glioblastoma (U87MG)	2	232526039	232554844	0.34	0.05	0.29	Metazoa_SRP
Glioblastoma (U87MG)	13	107142225	107162158	0.29	0.00	0.29	EFNB2
Glioblastoma (U87MG)	17	68152783	68183190	0.38	0.09	0.29	KCNJ2
Glioblastoma (U87MG)	10	102752997	102775915	0.44	0.16	0.28	LZTS2
Glioblastoma (U87MG)	14	77765872	77789479	0.39	0.10	0.28	GSTZ1
Glioblastoma (U87MG)	8	120648911	120687423	0.41	0.13	0.28	ENPP2
Glioblastoma (U87MG)	18	13610336	13640017	0.53	0.25	0.28	LDLRAD4
Glioblastoma (U87MG)	17	79345155	79376776	0.54	0.27	0.27	MIR4740
Glioblastoma (U87MG)	4	115547254	115584118	0.27	0.00	0.27	MIR577
Glioblastoma (U87MG)	16	15234031	15244187	0.27	0.00	0.27	Y_RNA
Glioblastoma (U87MG)	5	132105879	132116081	0.43	0.16	0.27	8-Sep

Glioblastoma (U87MG)	10	650150	686281	0.31	0.04	0.26	RP11-809C18.3
Glioblastoma (U87MG)	9	135976307	136024639	0.33	0.07	0.26	RALGDS
Glioblastoma (U87MG)	11	111845328	111865086	0.33	0.07	0.25	DIXDC1
Glioblastoma (U87MG)	10	22858862	22973344	0.25	0.00	0.25	PIP4K2A
Glioblastoma (U87MG)	1	198871424	198907549	0.48	0.23	0.25	RP11-31E23.1

Supplementary Table 5: Hypomethylated super-enhancers in cancer samples (δ HMR > 25%).

	Super-Enhancer			HMR coverage (%)			
**Tissue**	**Chr.**	**Start**	**End**	**Normal**	**Cancer**	**δ**	**Gene symbol**
Primary breast tumor (468PT)	2	91785127	91788546	0.00	1.00	1.00	AC233263.1
Primary breast tumor (468PT)	7	107947705	107953802	0.00	1.00	1.00	NRCAM
Primary breast tumor (468PT)	16	72320261	72327455	0.00	1.00	1.00	PMFBP1
Primary breast tumor (468PT)	3	74578493	74588238	0.00	1.00	1.00	CNTN3
Primary breast tumor (468PT)	10	118176539	118201896	0.00	0.99	0.99	PNLIPRP3
Primary breast tumor (468PT)	11	131923942	131950946	0.00	0.98	0.98	NTM
Primary breast tumor (468PT)	20	22354832	22372608	0.00	0.96	0.96	FOXA2
Primary breast tumor (468PT)	20	22354832	22372608	0.00	0.96	0.96	FOXA2
Primary breast tumor (468PT)	8	32082261	32138267	0.02	0.97	0.94	NRG1
Primary breast tumor (468PT)	8	32082261	32138267	0.02	0.97	0.94	NRG1
Primary breast tumor (468PT)	8	32082261	32138267	0.02	0.97	0.94	NRG1
Primary breast tumor (468PT)	8	32082261	32138267	0.02	0.97	0.94	NRG1
Primary breast tumor (468PT)	8	32082261	32138267	0.02	0.97	0.94	NRG1
Primary breast tumor (468PT)	8	32082261	32138267	0.02	0.97	0.94	NRG1
Primary breast tumor (468PT)	5	167229136	167249797	0.06	1.00	0.94	TENM2
Primary breast tumor (468PT)	4	166884826	166896752	0.00	0.93	0.93	TLL1
Primary breast tumor (468PT)	11	41071460	41077058	0.00	0.91	0.91	LRRC4C
Primary breast tumor (468PT)	2	17760510	17781417	0.06	0.97	0.91	VSNL1
Primary breast tumor (468PT)	21	28941125	28955796	0.00	0.90	0.90	ADAMTS5
Primary breast tumor (468PT)	15	84511818	84524836	0.06	0.97	0.90	ADAMTSL3
Primary breast tumor (468PT)	16	82659814	82694376	0.05	0.94	0.89	CDH13
Primary breast tumor (468PT)	8	32160365	32202004	0.05	0.93	0.87	NRG1
Primary breast tumor (468PT)	8	32160365	32202004	0.05	0.93	0.87	NRG1
Primary breast tumor (468PT)	8	32160365	32202004	0.05	0.93	0.87	NRG1
Primary breast tumor (468PT)	8	32160365	32202004	0.05	0.93	0.87	NRG1
Primary breast tumor (468PT)	8	32160365	32202004	0.05	0.93	0.87	NRG1
Primary breast tumor (468PT)	8	32160365	32202004	0.05	0.93	0.87	NRG1
Primary breast tumor (468PT)	12	29884879	29936970	0.04	0.90	0.86	TMTC1
Primary breast tumor (468PT)	3	74647878	74690393	0.05	0.91	0.86	CNTN3
Primary breast tumor (468PT)	11	122026659	122068967	0.07	0.93	0.86	BLID
Primary breast tumor (468PT)	12	19149492	19185438	0.00	0.85	0.85	PLEKHA5
Primary breast tumor (468PT)	12	19149492	19185438	0.00	0.85	0.85	PLEKHA5
Primary breast tumor (468PT)	12	19149492	19185438	0.00	0.85	0.85	PLEKHA5
Primary breast tumor (468PT)	12	19149492	19185438	0.00	0.85	0.85	PLEKHA5

Primary breast tumor (468PT)	5	61211861	61217024	0.00	0.83	0.83	C5orf64
Primary breast tumor (468PT)	11	109930034	109968654	0.06	0.87	0.81	ZC3H12C
Primary breast tumor (468PT)	10	108182232	108202133	0.00	0.81	0.81	SORCS1
Primary breast tumor (468PT)	5	167124831	167172069	0.01	0.81	0.80	TENM2
Primary breast tumor (468PT)	12	18411546	18429687	0.00	0.80	0.80	PIK3C2G
Primary breast tumor (468PT)	4	177902138	177945974	0.00	0.76	0.76	VEGFC
Primary breast tumor (468PT)	3	115502074	115525187	0.08	0.84	0.75	LSAMP
Primary breast tumor (468PT)	8	49607646	49623337	0.07	0.82	0.74	EFCAB1
Primary breast tumor (468PT)	8	123139023	123153617	0.09	0.81	0.72	HAS2
Primary breast tumor (468PT)	7	55760378	55777116	0.00	0.68	0.68	VOPP1
Primary breast tumor (468PT)	8	32403683	32442699	0.19	0.86	0.66	NRG1
Primary breast tumor (468PT)	20	1789115	1811959	0.13	0.77	0.64	SIRPA
Primary breast tumor (468PT)	21	39831004	39862029	0.02	0.65	0.63	ERG
Primary breast tumor (468PT)	17	5729361	5740922	0.00	0.61	0.61	WSCD1
Primary breast tumor (468PT)	3	62571130	62594277	0.00	0.53	0.53	CADPS
Primary breast tumor (468PT)	5	1930374	1976494	0.12	0.63	0.50	IRX4
Primary breast tumor (468PT)	10	92593455	92620169	0.06	0.55	0.49	HTR7
Primary breast tumor (468PT)	11	114162169	114180527	0.26	0.75	0.49	NNMT
Primary breast tumor (468PT)	6	121756401	121769000	0.38	0.86	0.48	GJA1
Primary breast tumor (468PT)	1	162875345	162886688	0.00	0.45	0.45	C1orf110
Primary breast tumor (468PT)	1	162875345	162886688	0.00	0.45	0.45	C1orf110
Primary breast tumor (468PT)	5	149314305	149321676	0.00	0.38	0.38	PDE6A
Primary breast tumor (468PT)	5	149314305	149321676	0.00	0.38	0.38	PDE6A
Primary breast tumor (468PT)	3	193545523	193550492	0.00	0.37	0.37	OPA1
Primary breast tumor (468PT)	17	39674641	39706091	0.17	0.52	0.35	KRT19
Primary breast tumor (468PT)	2	143614960	143638844	0.02	0.35	0.34	KYNU
Primary breast tumor (468PT)	10	65310940	65315929	0.00	0.33	0.33	REEP3
Primary breast tumor (468PT)	10	65310940	65315929	0.00	0.33	0.33	REEP3
Primary breast tumor (468PT)	15	83945999	83992250	0.08	0.41	0.33	BNC1
Primary breast tumor (468PT)	17	57902558	57931624	0.25	0.56	0.31	VMP1
Primary breast tumor (468PT)	7	130643572	130647502	0.19	0.49	0.30	MKLN1
Primary breast tumor (468PT)	1	218648751	218673670	0.00	0.29	0.29	C1orf143
Primary breast tumor (468PT)	7	18547133	18551508	0.00	0.29	0.29	HDAC9
Primary breast tumor (468PT)	17	18621223	18626374	0.00	0.29	0.29	TRIM16L
Primary breast tumor (468PT)	11	15986587	15993130	0.00	0.29	0.29	SOX6
Primary breast tumor (468PT)	13	73629841	73641096	0.29	0.57	0.28	KLF5
Primary breast tumor (468PT)	1	8062975	8088860	0.25	0.51	0.27	ERRFI1
Primary breast tumor (468PT)	1	160982545	160992918	0.21	0.46	0.25	F11R
Breast tumor metastasis (468LN)	2	91785127	91788546	0.00	1.00	1.00	AC233263.1

Breast tumor metastasis (468LN)	2	213706261	213711784	0.00	1.00	1.00	IKZF2
Breast tumor metastasis (468LN)	3	74578493	74588238	0.00	1.00	1.00	CNTN3
Breast tumor metastasis (468LN)	20	22354832	22372608	0.00	1.00	1.00	FOXA2
Breast tumor metastasis (468LN)	20	22354832	22372608	0.00	1.00	1.00	FOXA2
Breast tumor metastasis (468LN)	4	166884826	166896752	0.00	1.00	1.00	TLL1
Breast tumor metastasis (468LN)	17	5729361	5740922	0.00	0.99	0.99	WSCD1
Breast tumor metastasis (468LN)	13	106796195	106810591	0.00	0.97	0.97	EFNB2
Breast tumor metastasis (468LN)	4	74869808	74873810	0.00	0.96	0.96	CXCL5
Breast tumor metastasis (468LN)	3	62571130	62594277	0.00	0.95	0.95	CADPS
Breast tumor metastasis (468LN)	1	115150842	115162363	0.00	0.95	0.95	DENND2C
Breast tumor metastasis (468LN)	5	66176502	66195546	0.00	0.94	0.94	MAST4
Breast tumor metastasis (468LN)	5	167229136	167249797	0.06	1.00	0.94	TENM2
Breast tumor metastasis (468LN)	5	167124831	167172069	0.01	0.93	0.93	TENM2
Breast tumor metastasis (468LN)	11	41071460	41077058	0.00	0.91	0.91	LRRC4C
Breast tumor metastasis (468LN)	16	82089108	82117271	0.00	0.91	0.91	HSD17B2
Breast tumor metastasis (468LN)	11	93740640	93763165	0.05	0.96	0.90	HEPHL1
Breast tumor metastasis (468LN)	3	74647878	74690393	0.05	0.93	0.88	CNTN3
Breast tumor metastasis (468LN)	12	18411546	18429687	0.00	0.88	0.88	PIK3C2G
Breast tumor metastasis (468LN)	15	84511818	84524836	0.06	0.94	0.88	ADAMTSL3
Breast tumor metastasis (468LN)	12	52886804	52920914	0.07	0.95	0.88	KRT5
Breast tumor metastasis (468LN)	19	46683563	46726215	0.07	0.94	0.86	IGFL1
Breast tumor metastasis (468LN)	5	73526769	73548658	0.00	0.86	0.86	ARHGEF28
Breast tumor metastasis (468LN)	12	19149492	19185438	0.00	0.85	0.85	PLEKHA5
Breast tumor metastasis (468LN)	12	19149492	19185438	0.00	0.85	0.85	PLEKHA5
Breast tumor metastasis (468LN)	12	19149492	19185438	0.00	0.85	0.85	PLEKHA5
Breast tumor metastasis (468LN)	12	19149492	19185438	0.00	0.85	0.85	PLEKHA5
Breast tumor metastasis (468LN)	11	119990825	120009715	0.09	0.94	0.85	TRIM29
Breast tumor metastasis (468LN)	18	10411107	10423068	0.04	0.89	0.85	APCDD1
Breast tumor metastasis (468LN)	18	38445567	38458816	0.00	0.84	0.84	PIK3C3
Breast tumor metastasis (468LN)	5	169370816	169407860	0.09	0.92	0.83	FAM196B
Breast tumor metastasis (468LN)	6	12313555	12330214	0.04	0.87	0.83	EDN1
Breast tumor metastasis (468LN)	5	61211861	61217024	0.00	0.83	0.83	C5orf64
Breast tumor metastasis (468LN)	8	32082261	32138267	0.02	0.84	0.82	NRG1
Breast tumor metastasis (468LN)	8	32082261	32138267	0.02	0.84	0.82	NRG1
Breast tumor metastasis (468LN)	8	32082261	32138267	0.02	0.84	0.82	NRG1
Breast tumor metastasis (468LN)	8	32082261	32138267	0.02	0.84	0.82	NRG1
Breast tumor metastasis (468LN)	8	32082261	32138267	0.02	0.84	0.82	NRG1
Breast tumor metastasis (468LN)	8	32082261	32138267	0.02	0.84	0.82	NRG1
Breast tumor metastasis (468LN)	7	18547133	18551508	0.00	0.82	0.82	HDAC9

Breast tumor metastasis (468LN)	4	13908076	13924205	0.00	0.81	0.81	BOD1L1
Breast tumor metastasis (468LN)	6	143645048	143661635	0.00	0.81	0.81	AL031320.1
Breast tumor metastasis (468LN)	2	70570838	70616677	0.00	0.80	0.80	FAM136A
Breast tumor metastasis (468LN)	9	91379222	91406810	0.00	0.80	0.80	C9orf47
Breast tumor metastasis (468LN)	7	55000692	55038342	0.02	0.81	0.79	EGFR
Breast tumor metastasis (468LN)	4	74454199	74487257	0.06	0.85	0.79	RASSF6
Breast tumor metastasis (468LN)	2	121929305	121969683	0.03	0.81	0.78	TFCP2L1
Breast tumor metastasis (468LN)	15	71567004	71589795	0.10	0.88	0.78	THSD4
Breast tumor metastasis (468LN)	17	69398086	69438343	0.00	0.77	0.77	AC007461.1
Breast tumor metastasis (468LN)	12	70358689	70362371	0.00	0.77	0.77	MYRFL
Breast tumor metastasis (468LN)	8	32160365	32202004	0.05	0.81	0.76	NRG1
Breast tumor metastasis (468LN)	8	32160365	32202004	0.05	0.81	0.76	NRG1
Breast tumor metastasis (468LN)	8	32160365	32202004	0.05	0.81	0.76	NRG1
Breast tumor metastasis (468LN)	8	32160365	32202004	0.05	0.81	0.76	NRG1
Breast tumor metastasis (468LN)	8	32160365	32202004	0.05	0.81	0.76	NRG1
Breast tumor metastasis (468LN)	8	32160365	32202004	0.05	0.81	0.76	NRG1
Breast tumor metastasis (468LN)	5	1930374	1976494	0.12	0.88	0.76	IRX4
Breast tumor metastasis (468LN)	20	1445625	1487649	0.07	0.83	0.76	SIRPB2
Breast tumor metastasis (468LN)	10	108182232	108202133	0.00	0.76	0.76	SORCS1
Breast tumor metastasis (468LN)	5	66283790	66335622	0.08	0.83	0.75	MAST4
Breast tumor metastasis (468LN)	7	22893734	22901595	0.14	0.88	0.74	TOMM7
Breast tumor metastasis (468LN)	6	80234368	80256281	0.23	0.95	0.73	LCA5
Breast tumor metastasis (468LN)	22	27868674	27873807	0.23	0.95	0.72	MN1
Breast tumor metastasis (468LN)	21	39831004	39862029	0.02	0.72	0.71	ERG
Breast tumor metastasis (468LN)	6	54710175	54759167	0.04	0.74	0.70	FAM83B
Breast tumor metastasis (468LN)	3	123517889	123561019	0.03	0.72	0.69	MYLK
Breast tumor metastasis (468LN)	8	128672774	128685618	0.05	0.71	0.66	MYC
Breast tumor metastasis (468LN)	3	188984945	189053159	0.01	0.66	0.65	TPRG1
Breast tumor metastasis (468LN)	2	17760510	17781417	0.06	0.69	0.64	VSNL1
Breast tumor metastasis (468LN)	13	25237454	25247430	0.18	0.81	0.64	ATP12A
Breast tumor metastasis (468LN)	7	36690919	36727628	0.02	0.65	0.63	AOAH
Breast tumor metastasis (468LN)	3	189540773	189562008	0.08	0.69	0.62	TP63
Breast tumor metastasis (468LN)	3	189540773	189562008	0.08	0.69	0.62	TP63
Breast tumor metastasis (468LN)	3	189540773	189562008	0.08	0.69	0.62	TP63
Breast tumor metastasis (468LN)	3	189540773	189562008	0.08	0.69	0.62	TP63
Breast tumor metastasis (468LN)	3	189540773	189562008	0.08	0.69	0.62	TP63
Breast tumor metastasis (468LN)	17	39734929	39745765	0.08	0.69	0.61	KRT14
Breast tumor metastasis (468LN)	3	9208190	9218379	0.08	0.68	0.61	SRGAP3
Breast tumor metastasis (468LN)	8	66840219	66887130	0.06	0.66	0.61	DNAJC5B

Breast tumor metastasis (468LN)	8	66840219	66887130	0.06	0.66	0.61	DNAJC5B
Breast tumor metastasis (468LN)	5	148155468	148172554	0.00	0.60	0.60	ADRB2
Breast tumor metastasis (468LN)	3	111296776	111315059	0.00	0.59	0.59	ZBED2
Breast tumor metastasis (468LN)	5	64484499	64507100	0.00	0.59	0.59	ADAMTS6
Breast tumor metastasis (468LN)	5	52540140	52559537	0.03	0.62	0.59	MOCS2
Breast tumor metastasis (468LN)	11	12130227	12162030	0.10	0.67	0.57	MICAL2
Breast tumor metastasis (468LN)	1	94425779	94461050	0.03	0.60	0.56	ABCA4
Breast tumor metastasis (468LN)	15	83945999	83992250	0.08	0.65	0.56	BNC1
Breast tumor metastasis (468LN)	6	107163126	107200634	0.01	0.57	0.56	QRSL1
Breast tumor metastasis (468LN)	1	209527622	209609482	0.05	0.61	0.56	CAMK1G
Breast tumor metastasis (468LN)	9	124030410	124052489	0.40	0.95	0.55	GSN
Breast tumor metastasis (468LN)	3	44923928	44941628	0.03	0.58	0.54	TGM4
Breast tumor metastasis (468LN)	8	128402145	128416279	0.12	0.66	0.54	POU5F1B
Breast tumor metastasis (468LN)	7	130643572	130647502	0.19	0.73	0.53	MKLN1
Breast tumor metastasis (468LN)	10	82213990	82238676	0.25	0.78	0.53	TSPAN14
Breast tumor metastasis (468LN)	11	58340393	58348640	0.48	1.00	0.52	ZFP91-CNTF
Breast tumor metastasis (468LN)	2	1708289	1747387	0.12	0.62	0.50	PXDN
Breast tumor metastasis (468LN)	10	6762937	6792166	0.11	0.61	0.50	PRKCQ
Breast tumor metastasis (468LN)	4	100367626	100385121	0.00	0.50	0.50	ADH7
Breast tumor metastasis (468LN)	12	109114581	109129684	0.20	0.69	0.49	CORO1C
Breast tumor metastasis (468LN)	17	34104001	34120836	0.05	0.54	0.49	MMP28
Breast tumor metastasis (468LN)	7	134297576	134309091	0.00	0.48	0.48	BPGM
Breast tumor metastasis (468LN)	2	70745929	70782514	0.07	0.54	0.47	TGFA
Breast tumor metastasis (468LN)	2	208102152	208124172	0.08	0.55	0.47	KLF7
Breast tumor metastasis (468LN)	18	20393690	20449701	0.01	0.48	0.47	RBBP8
Breast tumor metastasis (468LN)	1	162875345	162886688	0.00	0.47	0.47	C1orf110
Breast tumor metastasis (468LN)	1	162875345	162886688	0.00	0.47	0.47	C1orf110
Breast tumor metastasis (468LN)	8	29196390	29211042	0.48	0.94	0.46	DUSP4
Breast tumor metastasis (468LN)	12	52820050	52849388	0.03	0.49	0.46	KRT6B
Breast tumor metastasis (468LN)	9	118011886	118026827	0.00	0.45	0.45	1-Dec
Breast tumor metastasis (468LN)	5	33299342	33322096	0.04	0.49	0.44	TARS
Breast tumor metastasis (468LN)	4	15893942	15940788	0.01	0.45	0.44	FGFBP1
Breast tumor metastasis (468LN)	16	68669444	68711125	0.14	0.58	0.44	CDH3
Breast tumor metastasis (468LN)	19	33840783	33869285	0.07	0.49	0.42	CEBPG
Breast tumor metastasis (468LN)	11	12085413	12114013	0.03	0.45	0.42	MICAL2
Breast tumor metastasis (468LN)	2	99524648	99538593	0.14	0.56	0.42	KIAA1211L
Breast tumor metastasis (468LN)	3	131851786	131856052	0.02	0.44	0.42	CPNE4
Breast tumor metastasis (468LN)	7	48120763	48135456	0.12	0.54	0.42	UPP1
Breast tumor metastasis (468LN)	22	25338358	25391181	0.11	0.52	0.41	TMEM211

Breast tumor metastasis (468LN)	6	143716698	143732923	0.04	0.44	0.40	AL031320.1
Breast tumor metastasis (468LN)	1	221611253	221636061	0.04	0.43	0.40	DUSP10
Breast tumor metastasis (468LN)	10	118176539	118201896	0.00	0.39	0.39	PNLIPRP3
Breast tumor metastasis (468LN)	18	28809492	28837065	0.06	0.44	0.38	DSG1
Breast tumor metastasis (468LN)	10	24737918	24757794	0.37	0.76	0.38	KIAA1217
Breast tumor metastasis (468LN)	20	51581498	51597746	0.45	0.82	0.37	TSHZ2
Breast tumor metastasis (468LN)	17	39674641	39706091	0.17	0.54	0.37	KRT19
Breast tumor metastasis (468LN)	4	11615367	11638574	0.00	0.37	0.37	HS3ST1
Breast tumor metastasis (468LN)	7	41136094	41161972	0.04	0.41	0.37	C7orf10
Breast tumor metastasis (468LN)	6	17863327	17868787	0.00	0.36	0.36	KIF13A
Breast tumor metastasis (468LN)	8	32403683	32442699	0.19	0.55	0.36	NRG1
Breast tumor metastasis (468LN)	11	128362001	128396377	0.21	0.57	0.36	ETS1
Breast tumor metastasis (468LN)	11	18328431	18352028	0.10	0.45	0.36	GTF2H1
Breast tumor metastasis (468LN)	X	105509168	105546610	0.00	0.35	0.35	MUM1L1
Breast tumor metastasis (468LN)	12	59433027	59479164	0.05	0.40	0.34	LRIG3
Breast tumor metastasis (468LN)	20	56261686	56292224	0.14	0.47	0.33	PMEPA1
Breast tumor metastasis (468LN)	5	168586808	168610831	0.05	0.38	0.33	SLIT3
Breast tumor metastasis (468LN)	11	12183685	12222874	0.04	0.36	0.33	MICAL2
Breast tumor metastasis (468LN)	21	30003125	30011412	0.00	0.33	0.33	N6AMT1
Breast tumor metastasis (468LN)	21	30003125	30011412	0.00	0.33	0.33	N6AMT1
Breast tumor metastasis (468LN)	21	30003125	30011412	0.00	0.33	0.33	N6AMT1
Breast tumor metastasis (468LN)	6	53475369	53500722	0.04	0.36	0.33	GCLC
Breast tumor metastasis (468LN)	5	95554865	95565313	0.00	0.32	0.32	PCSK1
Breast tumor metastasis (468LN)	11	114162169	114180527	0.26	0.58	0.32	NNMT
Breast tumor metastasis (468LN)	4	186225739	186230197	0.00	0.31	0.31	SNX25
Breast tumor metastasis (468LN)	2	206514186	206569665	0.11	0.41	0.30	NRP2
Breast tumor metastasis (468LN)	18	59613226	59621595	0.06	0.36	0.30	RNF152
Breast tumor metastasis (468LN)	11	15986587	15993130	0.00	0.30	0.30	SOX6
Breast tumor metastasis (468LN)	5	86394511	86425151	0.00	0.30	0.30	AC008394.1
Breast tumor metastasis (468LN)	17	805322	839104	0.10	0.40	0.29	NXN
Breast tumor metastasis (468LN)	21	40386166	40424480	0.08	0.37	0.29	PSMG1
Breast tumor metastasis (468LN)	6	116684394	116720580	0.05	0.34	0.29	DSE
Breast tumor metastasis (468LN)	6	111906089	111928358	0.33	0.61	0.28	TRAF3IP2
Breast tumor metastasis (468LN)	21	44720362	44794103	0.13	0.41	0.28	SIK1
Breast tumor metastasis (468LN)	3	111518531	111530936	0.08	0.35	0.27	PHLDB2
Breast tumor metastasis (468LN)	3	111518531	111530936	0.08	0.35	0.27	PHLDB2
Breast tumor metastasis (468LN)	21	44907375	44943674	0.06	0.33	0.27	SIK1
Breast tumor metastasis (468LN)	13	73984215	74005603	0.00	0.27	0.27	KLF5
Breast tumor metastasis (468LN)	9	111295882	111325768	0.00	0.27	0.27	ACTL7B

Breast tumor metastasis (468LN)	11	93860386	93869783	0.22	0.48	0.26	PANX1
Breast tumor metastasis (468LN)	10	99443070	99451242	0.24	0.49	0.25	AVPI1
Lung adenocarcinoma (H1437)	12	108664152	108729705	0.02	0.99	0.97	CMKLR1
Lung adenocarcinoma (H1437)	12	2321941	2506285	0.01	0.96	0.96	CACNA1C
Lung adenocarcinoma (H1437)	6	164505820	164531835	0.01	0.92	0.91	QKI
Lung adenocarcinoma (H1437)	3	16923413	16959538	0.07	0.94	0.86	PLCL2
Lung adenocarcinoma (H1437)	12	2160784	2308190	0.03	0.89	0.86	CACNA1C
Lung adenocarcinoma (H1437)	11	128560125	128616737	0.09	0.94	0.85	FLI1
Lung adenocarcinoma (H1437)	8	12937916	12990887	0.08	0.92	0.84	DLC1
Lung adenocarcinoma (H1437)	1	153579237	153590776	0.04	0.86	0.81	S100A14
Lung adenocarcinoma (H1437)	14	23898802	23913794	0.00	0.80	0.80	MYH7
Lung adenocarcinoma (H1437)	10	11188477	11246900	0.03	0.83	0.80	CELF2
Lung adenocarcinoma (H1437)	16	49857837	49893960	0.17	0.95	0.78	ZNF423
Lung adenocarcinoma (H1437)	17	32547489	32583972	0.05	0.82	0.77	CCL2
Lung adenocarcinoma (H1437)	11	67775074	67783359	0.10	0.84	0.74	UNC93B1
Lung adenocarcinoma (H1437)	14	23870473	23890571	0.00	0.72	0.72	MYH6
Lung adenocarcinoma (H1437)	5	175084363	175123938	0.05	0.76	0.71	HRH2
Lung adenocarcinoma (H1437)	6	45914072	45984711	0.02	0.72	0.70	CLIC5
Lung adenocarcinoma (H1437)	11	124745703	124772028	0.07	0.76	0.68	ROBO4
Lung adenocarcinoma (H1437)	15	68564041	68574856	0.24	0.91	0.68	FEM1B
Lung adenocarcinoma (H1437)	20	43963996	43977803	0.09	0.75	0.66	SDC4
Lung adenocarcinoma (H1437)	3	14834008	14933715	0.04	0.66	0.62	FGD5
Lung adenocarcinoma (H1437)	12	4377579	4414053	0.22	0.79	0.57	CCND2
Lung adenocarcinoma (H1437)	10	17256938	17276860	0.22	0.77	0.55	VIM
Lung adenocarcinoma (H1437)	11	74858188	74908024	0.00	0.54	0.54	SLCO2B1
Lung adenocarcinoma (H1437)	12	114806250	114852976	0.45	0.93	0.48	TBX5
Lung adenocarcinoma (H1437)	1	8063878	8088662	0.20	0.68	0.48	ERRFI1
Lung adenocarcinoma (H1437)	17	79337961	79404678	0.22	0.70	0.47	BAHCC1
Lung adenocarcinoma (H1437)	1	151960740	151974470	0.24	0.65	0.40	S100A10
Lung adenocarcinoma (H1437)	6	139690764	139698884	0.53	0.94	0.40	CITED2
Lung adenocarcinoma (H1437)	7	150642159	150687879	0.17	0.57	0.40	KCNH2
Lung adenocarcinoma (H1437)	16	637852	644850	0.41	0.79	0.39	RAB40C
Lung adenocarcinoma (H1437)	16	29165405	29212441	0.01	0.39	0.38	SNX29P2
Lung adenocarcinoma (H1437)	22	50323334	50364887	0.25	0.61	0.37	PIM3
Lung adenocarcinoma (H1437)	3	53269245	53309568	0.07	0.42	0.35	TKT
Lung adenocarcinoma (H1437)	1	153537691	153541885	0.41	0.76	0.34	S100A2
Lung adenocarcinoma (H1437)	2	145236716	145282659	0.29	0.63	0.34	ZEB2
Lung adenocarcinoma (H1437)	9	132241137	132264724	0.28	0.61	0.33	NTMT1
Lung adenocarcinoma (H1437)	6	82458588	82464329	0.56	0.89	0.33	FAM46A

Lung adenocarcinoma (H1437)	17	48131999	48143308	0.25	0.58	0.33	ITGA3
Lung adenocarcinoma (H1437)	17	79300771	79323073	0.29	0.61	0.32	TMEM105
Lung adenocarcinoma (H1437)	3	64669880	64677423	0.57	0.88	0.32	ADAMTS9
Lung adenocarcinoma (H1437)	3	71625820	71634865	0.45	0.77	0.32	FOXP1
Lung adenocarcinoma (H1437)	21	47373962	47413579	0.16	0.48	0.31	COL6A1
Lung adenocarcinoma (H1437)	5	180668274	180675859	0.53	0.84	0.31	GNB2L1
Lung adenocarcinoma (H1437)	17	80223721	80257686	0.16	0.47	0.31	CSNK1D
Lung adenocarcinoma (H1437)	16	86504285	86554076	0.52	0.83	0.31	FOXF1
Lung adenocarcinoma (H1437)	22	19830490	19844157	0.25	0.55	0.30	C22orf29
Lung adenocarcinoma (H1437)	16	19117268	19151258	0.11	0.40	0.29	CTD-2349B8.1
Lung adenocarcinoma (H1437)	1	153504009	153510224	0.41	0.70	0.29	S100A6
Lung adenocarcinoma (H1437)	3	9437015	9444925	0.71	1.00	0.29	SETD5
Lung adenocarcinoma (H1437)	12	52536940	52547179	0.09	0.37	0.29	KRT80
Lung adenocarcinoma (H1437)	12	7032586	7039696	0.64	0.92	0.28	ATN1
Lung adenocarcinoma (H1437)	2	127412308	127428737	0.14	0.42	0.27	GYPC
Lung adenocarcinoma (H1437)	6	74224953	74234140	0.53	0.79	0.26	EEF1A1
Lung adenocarcinoma (H1437)	10	33226742	33251655	0.24	0.50	0.26	ITGB1
Lung adenocarcinoma (H1437)	19	13956569	13963420	0.41	0.66	0.25	NANOS3
Lung squamous cell carcinoma (H157)	14	23898802	23913794	0.00	0.97	0.97	MYH7
Lung squamous cell carcinoma (H157)	1	164717131	164764470	0.01	0.98	0.97	PBX1
Lung squamous cell carcinoma (H157)	15	92456353	92524948	0.01	0.98	0.97	SLCO3A1
Lung squamous cell carcinoma (H157)	12	2321941	2506285	0.01	0.98	0.97	CACNA1C
Lung squamous cell carcinoma (H157)	12	108664152	108729705	0.02	0.99	0.97	CMKLR1
Lung squamous cell carcinoma (H157)	7	51322792	51385835	0.03	0.98	0.95	COBL
Lung squamous cell carcinoma (H157)	22	33196018	33254838	0.06	1.00	0.94	TIMP3
Lung squamous cell carcinoma (H157)	6	11345367	11394452	0.01	0.95	0.94	NEDD9
Lung squamous cell carcinoma (H157)	2	218653998	218760935	0.01	0.95	0.93	TNS1
Lung squamous cell carcinoma (H157)	3	14834008	14933715	0.04	0.97	0.93	FGD5
Lung squamous cell carcinoma (H157)	22	32924539	32976379	0.01	0.93	0.91	SYN3
Lung squamous cell carcinoma (H157)	12	2160784	2308190	0.03	0.94	0.91	CACNA1C
Lung squamous cell carcinoma (H157)	9	71319153	71371192	0.06	0.97	0.91	PIP5K1B
Lung squamous cell carcinoma (H157)	22	34206955	34272052	0.03	0.94	0.91	LARGE
Lung squamous cell carcinoma (H157)	3	123047106	123169548	0.02	0.93	0.91	ADCY5
Lung squamous cell carcinoma (H157)	22	33011575	33041335	0.07	0.97	0.90	SYN3
Lung squamous cell carcinoma (H157)	11	117730279	117757325	0.09	0.99	0.90	FXYD6
Lung squamous cell carcinoma (H157)	10	116233610	116285340	0.00	0.89	0.89	ABLIM1
Lung squamous cell carcinoma (H157)	11	19719061	19814464	0.05	0.94	0.89	NAV2
Lung squamous cell carcinoma (H157)	5	175084363	175123938	0.05	0.94	0.89	HRH2
Lung squamous cell carcinoma (H157)	15	92394913	92441672	0.06	0.95	0.89	SLCO3A1

Lung squamous cell carcinoma (H157)	1	175138842	175191990	0.07	0.95	0.88	KIAA0040
Lung squamous cell carcinoma (H157)	3	16923413	16959538	0.07	0.95	0.88	PLCL2
Lung squamous cell carcinoma (H157)	6	45914072	45984711	0.02	0.89	0.87	CLIC5
Lung squamous cell carcinoma (H157)	9	677510	756481	0.04	0.90	0.86	KANK1
Lung squamous cell carcinoma (H157)	4	23855744	23906109	0.05	0.91	0.86	PPARGC1A
Lung squamous cell carcinoma (H157)	10	11188477	11246900	0.03	0.89	0.86	CELF2
Lung squamous cell carcinoma (H157)	10	97137580	97321838	0.02	0.87	0.85	SORBS1
Lung squamous cell carcinoma (H157)	1	208373068	208418917	0.11	0.96	0.85	PLXNA2
Lung squamous cell carcinoma (H157)	6	112519021	112620635	0.03	0.88	0.84	LAMA4
Lung squamous cell carcinoma (H157)	10	116297903	116338177	0.00	0.83	0.83	ABLIM1
Lung squamous cell carcinoma (H157)	11	11556367	11614464	0.00	0.82	0.82	GALNT18
Lung squamous cell carcinoma (H157)	1	201326267	201371297	0.03	0.84	0.81	LAD1
Lung squamous cell carcinoma (H157)	14	23870473	23890571	0.00	0.81	0.81	MYH6
Lung squamous cell carcinoma (H157)	2	218814905	218887533	0.05	0.85	0.80	TNS1
Lung squamous cell carcinoma (H157)	1	208057100	208099004	0.05	0.83	0.79	CD34
Lung squamous cell carcinoma (H157)	13	113612236	113642719	0.07	0.84	0.78	MCF2L
Lung squamous cell carcinoma (H157)	6	46859103	46921360	0.04	0.81	0.77	GPR116
Lung squamous cell carcinoma (H157)	11	128560125	128616737	0.09	0.86	0.77	FLI1
Lung squamous cell carcinoma (H157)	2	1701232	1756057	0.07	0.83	0.76	PXDN
Lung squamous cell carcinoma (H157)	2	206546300	206580305	0.14	0.90	0.76	NRP2
Lung squamous cell carcinoma (H157)	3	46885844	46916759	0.08	0.83	0.75	MYL3
Lung squamous cell carcinoma (H157)	10	45484961	45497498	0.13	0.87	0.74	ZNF22
Lung squamous cell carcinoma (H157)	16	49857837	49893960	0.17	0.91	0.74	ZNF423
Lung squamous cell carcinoma (H157)	9	73070671	73110216	0.03	0.77	0.74	KLF9
Lung squamous cell carcinoma (H157)	3	13031469	13061226	0.11	0.85	0.73	IQSEC1
Lung squamous cell carcinoma (H157)	1	203236433	203297636	0.16	0.87	0.71	BTG2
Lung squamous cell carcinoma (H157)	22	18913160	18924266	0.07	0.78	0.71	PRODH
Lung squamous cell carcinoma (H157)	2	42273101	42300251	0.08	0.78	0.70	PKDCC
Lung squamous cell carcinoma (H157)	1	167585748	167668005	0.01	0.71	0.69	RCSD1
Lung squamous cell carcinoma (H157)	10	116356071	116457594	0.02	0.70	0.68	ABLIM1
Lung squamous cell carcinoma (H157)	6	164505820	164531835	0.01	0.69	0.67	QKI
Lung squamous cell carcinoma (H157)	15	64259521	64312980	0.00	0.66	0.66	DAPK2
Lung squamous cell carcinoma (H157)	3	123371190	123441485	0.03	0.67	0.64	MYLK
Lung squamous cell carcinoma (H157)	6	167010067	167054751	0.08	0.71	0.63	RPS6KA2
Lung squamous cell carcinoma (H157)	1	16338365	16348730	0.11	0.73	0.63	CLCNKA
Lung squamous cell carcinoma (H157)	11	113906599	114092628	0.05	0.67	0.62	ZBTB16
Lung squamous cell carcinoma (H157)	14	100196476	100226407	0.15	0.75	0.61	EML1
Lung squamous cell carcinoma (H157)	17	32547489	32583972	0.05	0.64	0.59	CCL2
Lung squamous cell carcinoma (H157)	11	58938916	58992734	0.05	0.64	0.59	MPEG1

Lung squamous cell carcinoma (H157)	2	159810283	159870494	0.06	0.61	0.55	TANC1
Lung squamous cell carcinoma (H157)	5	148793738	148806368	0.07	0.62	0.55	IL17B
Lung squamous cell carcinoma (H157)	17	53342089	53373406	0.13	0.67	0.54	HLF
Lung squamous cell carcinoma (H157)	3	138066340	138119923	0.04	0.57	0.53	MRAS
Lung squamous cell carcinoma (H157)	10	88420822	88454560	0.01	0.55	0.53	LDB3
Lung squamous cell carcinoma (H157)	7	150642159	150687879	0.17	0.69	0.52	KCNH2
Lung squamous cell carcinoma (H157)	3	128718996	128746653	0.14	0.66	0.52	EFCC1
Lung squamous cell carcinoma (H157)	9	73168217	73230803	0.02	0.54	0.52	KLF9
Lung squamous cell carcinoma (H157)	5	141183253	141261839	0.14	0.65	0.51	PCDH1
Lung squamous cell carcinoma (H157)	2	233923287	233954214	0.11	0.62	0.51	INPP5D
Lung squamous cell carcinoma (H157)	1	26386415	26449049	0.04	0.55	0.51	PDIK1L
Lung squamous cell carcinoma (H157)	15	66905412	66955707	0.04	0.55	0.51	RP11-321F6.1
Lung squamous cell carcinoma (H157)	19	33751162	33802757	0.14	0.64	0.50	CEBPA
Lung squamous cell carcinoma (H157)	22	37562253	37596293	0.18	0.67	0.49	C1QTNF6
Lung squamous cell carcinoma (H157)	1	41939307	41981913	0.03	0.52	0.49	EDN2
Lung squamous cell carcinoma (H157)	16	46764273	46798191	0.03	0.51	0.49	MYLK3
Lung squamous cell carcinoma (H157)	1	47676896	47710031	0.21	0.70	0.49	TAL1
Lung squamous cell carcinoma (H157)	15	65346572	65383634	0.08	0.56	0.48	KBTBD13
Lung squamous cell carcinoma (H157)	3	53174020	53206578	0.08	0.55	0.47	PRKCD
Lung squamous cell carcinoma (H157)	12	114806250	114852976	0.45	0.90	0.45	TBX5
Lung squamous cell carcinoma (H157)	10	45452698	45465905	0.11	0.55	0.45	RASSF4
Lung squamous cell carcinoma (H157)	15	85359756	85408023	0.01	0.46	0.45	ALPK3
Lung squamous cell carcinoma (H157)	12	109547560	109572829	0.04	0.49	0.44	ACACB
Lung squamous cell carcinoma (H157)	5	148650125	148713310	0.02	0.46	0.44	AFAP1L1
Lung squamous cell carcinoma (H157)	2	220473619	220511327	0.05	0.49	0.44	SLC4A3
Lung squamous cell carcinoma (H157)	3	126068503	126077592	0.29	0.73	0.44	KLF15
Lung squamous cell carcinoma (H157)	16	29165405	29212441	0.01	0.45	0.43	SNX29P2
Lung squamous cell carcinoma (H157)	12	52288433	52321075	0.05	0.47	0.43	ACVRL1
Lung squamous cell carcinoma (H157)	5	148513364	148558772	0.02	0.44	0.42	ABLIM3
Lung squamous cell carcinoma (H157)	6	11194797	11293348	0.06	0.48	0.42	NEDD9
Lung squamous cell carcinoma (H157)	11	62646517	62690197	0.11	0.52	0.41	CHRM1
Lung squamous cell carcinoma (H157)	15	57840518	57983809	0.01	0.42	0.41	GCOM1
Lung squamous cell carcinoma (H157)	2	30440179	30497935	0.08	0.48	0.40	LBH
Lung squamous cell carcinoma (H157)	1	94485792	94536633	0.03	0.43	0.40	ABCA4
Lung squamous cell carcinoma (H157)	5	142177195	142264107	0.00	0.40	0.39	ARHGAP26
Lung squamous cell carcinoma (H157)	16	57653421	57683065	0.02	0.41	0.39	GPR56
Lung squamous cell carcinoma (H157)	2	159936457	160087427	0.01	0.40	0.39	TANC1
Lung squamous cell carcinoma (H157)	8	12610121	12624953	0.34	0.73	0.38	LONRF1
Lung squamous cell carcinoma (H157)	12	117479472	117535649	0.01	0.39	0.38	TESC

Lung squamous cell carcinoma (H157)	16	58484914	58536190	0.08	0.46	0.38	NDRG4
Lung squamous cell carcinoma (H157)	20	19737283	19767891	0.13	0.50	0.38	AL121761.2
Lung squamous cell carcinoma (H157)	10	73469788	73536258	0.05	0.42	0.37	C10orf54
Lung squamous cell carcinoma (H157)	3	156802993	156855459	0.06	0.43	0.37	CCNL1
Lung squamous cell carcinoma (H157)	6	13383397	13489697	0.04	0.41	0.37	AL583828.1
Lung squamous cell carcinoma (H157)	9	116236515	116397394	0.04	0.41	0.37	RGS3
Lung squamous cell carcinoma (H157)	1	55007351	55053202	0.05	0.41	0.36	ACOT11
Lung squamous cell carcinoma (H157)	16	66392035	66422229	0.05	0.41	0.36	CDH5
Lung squamous cell carcinoma (H157)	17	4379415	4447094	0.09	0.44	0.35	MYBBP1A
Lung squamous cell carcinoma (H157)	10	105494540	105555321	0.01	0.36	0.34	SH3PXD2A
Lung squamous cell carcinoma (H157)	11	74858188	74908024	0.00	0.34	0.34	SLCO2B1
Lung squamous cell carcinoma (H157)	10	71158153	71242818	0.05	0.38	0.33	TSPAN15
Lung squamous cell carcinoma (H157)	1	151960740	151974470	0.24	0.57	0.33	S100A10
Lung squamous cell carcinoma (H157)	22	36541286	36558381	0.18	0.51	0.33	APOL3
Lung squamous cell carcinoma (H157)	1	2056832	2083663	0.06	0.38	0.32	PRKCZ
Lung squamous cell carcinoma (H157)	6	33659980	33715998	0.04	0.37	0.32	IP6K3
Lung squamous cell carcinoma (H157)	20	24972518	25040392	0.09	0.40	0.31	ACSS1
Lung squamous cell carcinoma (H157)	17	56583934	56616821	0.16	0.47	0.31	4-Sep
Lung squamous cell carcinoma (H157)	14	89852016	89897880	0.11	0.42	0.31	FOXN3-AS1
Lung squamous cell carcinoma (H157)	9	129783892	129898008	0.01	0.32	0.31	ANGPTL2
Lung squamous cell carcinoma (H157)	1	31839527	31868307	0.05	0.36	0.31	FABP3
Lung squamous cell carcinoma (H157)	12	6221157	6351408	0.03	0.34	0.30	CD9
Lung squamous cell carcinoma (H157)	17	48981354	49028680	0.06	0.36	0.30	SPAG9
Lung squamous cell carcinoma (H157)	3	46731656	46739274	0.17	0.47	0.30	ALS2CL
Lung squamous cell carcinoma (H157)	14	75724170	75782540	0.16	0.46	0.30	FOS
Lung squamous cell carcinoma (H157)	3	125978012	126033776	0.04	0.33	0.29	KLF15
Lung squamous cell carcinoma (H157)	17	72422406	72460049	0.19	0.47	0.28	GPRC5C
Lung squamous cell carcinoma (H157)	15	68564041	68574856	0.24	0.52	0.28	FEM1B
Lung squamous cell carcinoma (H157)	19	2567152	2637706	0.08	0.35	0.28	GNG7
Lung squamous cell carcinoma (H157)	17	21178575	21194211	0.42	0.70	0.28	MAP2K3
Lung squamous cell carcinoma (H157)	16	53534624	53554437	0.15	0.41	0.26	AKTIP
Lung squamous cell carcinoma (H157)	11	72851228	72894684	0.09	0.36	0.26	FCHSD2
Lung squamous cell carcinoma (H157)	4	89203835	89219912	0.17	0.43	0.26	PPM1K
Lung squamous cell carcinoma (H157)	8	145021828	145029514	0.53	0.78	0.25	PLEC
Small cell lung cancer (H1672)	17	32547489	32583972	0.05	0.95	0.90	CCL2
Small cell lung cancer (H1672)	11	58938916	58992734	0.05	0.95	0.90	MPEG1
Small cell lung cancer (H1672)	14	23898802	23913794	0.00	0.89	0.89	MYH7
Small cell lung cancer (H1672)	16	88262997	88280525	0.07	0.94	0.88	BANP
Small cell lung cancer (H1672)	14	23870473	23890571	0.00	0.82	0.82	MYH6

Small cell lung cancer (H1672)	12	10862096	10876772	0.16	0.97	0.81	CSDA
Small cell lung cancer (H1672)	1	145208293	145266278	0.07	0.87	0.80	NOTCH2NL
Small cell lung cancer (H1672)	19	48218393	48248060	0.12	0.87	0.75	EHD2
Small cell lung cancer (H1672)	7	100727881	100783471	0.09	0.84	0.74	SERPINE1
Small cell lung cancer (H1672)	16	29259027	29284865	0.01	0.74	0.73	SNX29P2
Small cell lung cancer (H1672)	11	44746277	44803353	0.04	0.77	0.72	TSPAN18
Small cell lung cancer (H1672)	12	4377579	4414053	0.22	0.94	0.72	CCND2
Small cell lung cancer (H1672)	19	2567152	2637706	0.08	0.78	0.71	GNG7
Small cell lung cancer (H1672)	11	44584904	44654491	0.04	0.74	0.69	CD82
Small cell lung cancer (H1672)	X	64886373	64932289	0.26	0.96	0.69	MSN
Small cell lung cancer (H1672)	18	19743086	19793872	0.19	0.88	0.69	GATA6
Small cell lung cancer (H1672)	21	47373962	47413579	0.16	0.85	0.68	COL6A1
Small cell lung cancer (H1672)	7	116138842	116200757	0.10	0.76	0.66	CAV1
Small cell lung cancer (H1672)	19	6055959	6111185	0.10	0.70	0.60	RFX2
Small cell lung cancer (H1672)	8	11532909	11620420	0.32	0.90	0.59	C8orf49
Small cell lung cancer (H1672)	1	151960740	151974470	0.24	0.76	0.52	S100A10
Small cell lung cancer (H1672)	10	46950950	46984234	0.12	0.63	0.52	SYT15
Small cell lung cancer (H1672)	1	51433106	51444971	0.49	1.00	0.51	CDKN2C
Small cell lung cancer (H1672)	21	46711726	46750687	0.07	0.57	0.50	POFUT2
Small cell lung cancer (H1672)	21	46711726	46750687	0.07	0.57	0.50	POFUT2
Small cell lung cancer (H1672)	21	46711726	46750687	0.07	0.57	0.50	POFUT2
Small cell lung cancer (H1672)	2	241925890	241950654	0.12	0.61	0.49	SNED1
Small cell lung cancer (H1672)	12	10364085	10373949	0.26	0.73	0.47	GABARAPL1
Small cell lung cancer (H1672)	1	144923313	145010556	0.02	0.48	0.46	AL590452.1
Small cell lung cancer (H1672)	16	3102086	3157272	0.10	0.56	0.46	ZSCAN10
Small cell lung cancer (H1672)	19	18401834	18406232	0.52	0.97	0.45	JUND
Small cell lung cancer (H1672)	16	86504285	86554076	0.52	0.96	0.45	FOXF1
Small cell lung cancer (H1672)	11	62646517	62690197	0.11	0.55	0.44	CHRM1
Small cell lung cancer (H1672)	19	45244285	45262369	0.46	0.89	0.43	BCL3
Small cell lung cancer (H1672)	11	1770660	1805042	0.08	0.51	0.43	CTSD
Small cell lung cancer (H1672)	19	2462980	2496458	0.29	0.70	0.42	GADD45B
Small cell lung cancer (H1672)	19	33751162	33802757	0.14	0.55	0.41	CEBPA
Small cell lung cancer (H1672)	3	46885844	46916759	0.08	0.48	0.40	MYL3
Small cell lung cancer (H1672)	13	114821455	114835116	0.12	0.52	0.40	RASA3
Small cell lung cancer (H1672)	7	143071647	143093995	0.11	0.51	0.40	EPHA1
Small cell lung cancer (H1672)	13	110937717	111086117	0.06	0.46	0.40	COL4A2
Small cell lung cancer (H1672)	22	37562253	37596293	0.18	0.56	0.38	C1QTNF6
Small cell lung cancer (H1672)	17	81037486	81068828	0.21	0.59	0.38	METRNL
Small cell lung cancer (H1672)	13	114863283	114898583	0.15	0.52	0.37	RASA3

Small cell lung cancer (H1672)	12	53602263	53626034	0.27	0.62	0.36	RARG
Small cell lung cancer (H1672)	11	6338518	6348300	0.22	0.56	0.34	PRKCDBP
Small cell lung cancer (H1672)	16	88809516	88852538	0.11	0.43	0.32	PIEZO1
Small cell lung cancer (H1672)	19	45347579	45383849	0.17	0.49	0.32	PVRL2
Small cell lung cancer (H1672)	9	132161805	132179421	0.11	0.42	0.32	NTMT1
Small cell lung cancer (H1672)	16	29165405	29212441	0.01	0.33	0.31	SNX29P2
Small cell lung cancer (H1672)	1	2475405	2484449	0.45	0.76	0.31	TNFRSF14
Small cell lung cancer (H1672)	10	134321138	134365757	0.10	0.41	0.31	INPP5A
Small cell lung cancer (H1672)	6	139690764	139698884	0.53	0.84	0.31	CITED2
Small cell lung cancer (H1672)	8	11663395	11764350	0.05	0.35	0.30	CTSB
Small cell lung cancer (H1672)	10	17256938	17276860	0.22	0.52	0.30	VIM
Small cell lung cancer (H1672)	20	24972518	25040392	0.09	0.39	0.30	ACSS1
Small cell lung cancer (H1672)	6	30880966	30905424	0.07	0.37	0.30	SFTA2
Small cell lung cancer (H1672)	22	39616903	39707444	0.13	0.42	0.29	AL031590.1
Small cell lung cancer (H1672)	3	128718996	128746653	0.14	0.43	0.29	EFCC1
Small cell lung cancer (H1672)	1	12192038	12247305	0.08	0.36	0.28	TNFRSF1B
Small cell lung cancer (H1672)	13	110868117	110924162	0.03	0.30	0.27	COL4A2
Small cell lung cancer (H1672)	13	111155320	111212932	0.02	0.29	0.27	COL4A2
Small cell lung cancer (H1672)	3	9437015	9444925	0.71	0.97	0.26	SETD5
Small cell lung cancer (H1672)	6	3053230	3070286	0.27	0.52	0.25	RIPK1
Primary colon tumor (Colon_P)	11	64654606	64662582	0.23	0.87	0.64	EHD1
Primary colon tumor (Colon_P)	11	34644271	34676099	0.09	0.70	0.61	EHF
Primary colon tumor (Colon_P)	17	75277484	75285351	0.20	0.78	0.57	9-Sep
Primary colon tumor (Colon_P)	1	1092807	1104591	0.35	0.90	0.56	TTLL10
Primary colon tumor (Colon_P)	12	6478956	6487347	0.24	0.78	0.54	LTBR
Primary colon tumor (Colon_P)	19	35605339	35613006	0.23	0.76	0.53	FXYD3
Primary colon tumor (Colon_P)	1	207111967	207121081	0.23	0.74	0.51	PIGR
Primary colon tumor (Colon_P)	17	46021666	46045798	0.16	0.64	0.48	CDK5RAP3
Primary colon tumor (Colon_P)	9	140082154	140094655	0.20	0.67	0.47	TPRN
Primary colon tumor (Colon_P)	1	153579813	153590695	0.05	0.52	0.47	S100A14
Primary colon tumor (Colon_P)	1	234949066	234963582	0.06	0.52	0.46	IRF2BP2
Primary colon tumor (Colon_P)	1	234949066	234963582	0.06	0.52	0.46	IRF2BP2
Primary colon tumor (Colon_P)	11	72485443	72499721	0.10	0.56	0.46	STARD10
Primary colon tumor (Colon_P)	1	155900806	155917642	0.20	0.63	0.44	RXFP4
Primary colon tumor (Colon_P)	16	70091792	70101175	0.34	0.75	0.41	RP11-419C5.2
Primary colon tumor (Colon_P)	15	40634401	40644052	0.05	0.44	0.39	PHGR1
Primary colon tumor (Colon_P)	22	37406895	37441393	0.08	0.47	0.39	MPST
Primary colon tumor (Colon_P)	17	56404613	56418558	0.34	0.73	0.38	BZRAP1
Primary colon tumor (Colon_P)	8	145725241	145730866	0.32	0.70	0.38	GPT

Primary colon tumor (Colon_P)	1	167049094	167060421	0.09	0.46	0.38	GPA33
Primary colon tumor (Colon_P)	10	3816041	3829259	0.31	0.68	0.38	KLF6
Primary colon tumor (Colon_P)	21	43876507	43895539	0.03	0.39	0.37	SLC37A1
Primary colon tumor (Colon_P)	1	3369826	3378616	0.30	0.66	0.37	ARHGEF16
Primary colon tumor (Colon_P)	22	46479313	46489352	0.06	0.43	0.36	FLJ27365
Primary colon tumor (Colon_P)	7	130570945	130647038	0.16	0.51	0.36	MKLN1
Primary colon tumor (Colon_P)	1	202072763	202088434	0.10	0.43	0.33	GPR37L1
Primary colon tumor (Colon_P)	17	7161661	7168259	0.63	0.95	0.32	CLDN7
Primary colon tumor (Colon_P)	16	31137728	31147935	0.17	0.47	0.31	PRSS8
Primary colon tumor (Colon_P)	1	46632072	46655214	0.08	0.38	0.30	TSPAN1
Primary colon tumor (Colon_P)	20	62312602	62332044	0.16	0.45	0.29	TNFRSF6B
Primary colon tumor (Colon_P)	19	49115902	49124026	0.39	0.67	0.29	SPHK2
Primary colon tumor (Colon_P)	15	45726853	45749919	0.09	0.38	0.29	C15orf48
Primary colon tumor (Colon_P)	2	173291273	173330716	0.25	0.53	0.28	ITGA6
Primary colon tumor (Colon_P)	20	62153361	62177846	0.18	0.46	0.28	PTK6
Primary colon tumor (Colon_P)	2	200306225	200342782	0.56	0.84	0.28	SATB2
Primary colon tumor (Colon_P)	20	25200574	25236386	0.08	0.36	0.28	PYGB
Primary colon tumor (Colon_P)	21	40159118	40212366	0.18	0.44	0.27	ETS2
Primary colon tumor (Colon_P)	1	226052374	226087213	0.19	0.46	0.27	LEFTY1
Primary colon tumor (Colon_P)	3	58642829	58682800	0.06	0.32	0.26	FAM3D
Primary colon tumor (Colon_P)	16	574288	586492	0.27	0.52	0.26	SOLH
Primary colon tumor (Colon_P)	4	6747543	6755479	0.18	0.44	0.26	KIAA0232
Primary colon tumor (Colon_P)	14	50465553	50471375	0.66	0.92	0.25	C14orf182
Primary colon metastasis (Colon_M)	15	40634401	40644052	0.05	0.73	0.67	PHGR1
Primary colon metastasis (Colon_M)	11	64654606	64662582	0.23	0.87	0.64	EHD1
Primary colon metastasis (Colon_M)	16	70091792	70101175	0.34	0.96	0.61	RP11-419C5.2
Primary colon metastasis (Colon_M)	17	75277484	75285351	0.20	0.80	0.60	9-Sep
Primary colon metastasis (Colon_M)	11	34644271	34676099	0.09	0.64	0.55	EHF
Primary colon metastasis (Colon_M)	17	56404613	56418558	0.34	0.87	0.53	BZRAP1
Primary colon metastasis (Colon_M)	7	130570945	130647038	0.16	0.66	0.51	MKLN1
Primary colon metastasis (Colon_M)	7	50410776	50436416	0.00	0.49	0.49	IKZF1
Primary colon metastasis (Colon_M)	9	140082154	140094655	0.20	0.68	0.49	TPRN
Primary colon metastasis (Colon_M)	1	153579813	153590695	0.05	0.53	0.49	S100A14
Primary colon metastasis (Colon_M)	11	72485443	72499721	0.10	0.57	0.47	STARD10
Primary colon metastasis (Colon_M)	1	234949066	234963582	0.06	0.50	0.45	IRF2BP2
Primary colon metastasis (Colon_M)	1	234949066	234963582	0.06	0.50	0.45	IRF2BP2
Primary colon metastasis (Colon_M)	10	3816041	3829259	0.31	0.75	0.44	KLF6
Primary colon metastasis (Colon_M)	16	637814	644973	0.45	0.89	0.44	RAB40C
Primary colon metastasis (Colon_M)	5	169688532	169740245	0.01	0.45	0.43	LCP2

Primary colon metastasis (Colon_M)	22	46479313	46489352	0.06	0.49	0.42	FLJ27365
Primary colon metastasis (Colon_M)	1	155900806	155917642	0.20	0.62	0.42	RXFP4
Primary colon metastasis (Colon_M)	4	6747543	6755479	0.18	0.60	0.41	KIAA0232
Primary colon metastasis (Colon_M)	1	1092807	1104591	0.35	0.75	0.41	TTLL10
Primary colon metastasis (Colon_M)	7	142493655	142510845	0.11	0.51	0.40	PRSS1
Primary colon metastasis (Colon_M)	1	3369826	3378616	0.30	0.69	0.39	ARHGEF16
Primary colon metastasis (Colon_M)	7	50345823	50370722	0.06	0.45	0.39	IKZF1
Primary colon metastasis (Colon_M)	2	219149842	219160653	0.44	0.82	0.38	TMBIM1
Primary colon metastasis (Colon_M)	1	202072763	202088434	0.10	0.47	0.37	GPR37L1
Primary colon metastasis (Colon_M)	3	156836974	156855523	0.13	0.49	0.36	CCNL1
Primary colon metastasis (Colon_M)	8	145725241	145730866	0.32	0.68	0.35	GPT
Primary colon metastasis (Colon_M)	16	31137728	31147935	0.17	0.51	0.35	PRSS8
Primary colon metastasis (Colon_M)	13	27523605	27558934	0.13	0.47	0.34	USP12
Primary colon metastasis (Colon_M)	17	46021666	46045798	0.16	0.50	0.34	CDK5RAP3
Primary colon metastasis (Colon_M)	6	41031558	41042616	0.34	0.67	0.33	NFYA
Primary colon metastasis (Colon_M)	1	113240401	113250840	0.29	0.62	0.32	RHOC
Primary colon metastasis (Colon_M)	18	72918561	72932349	0.40	0.72	0.32	TSHZ1
Primary colon metastasis (Colon_M)	17	7161661	7168259	0.63	0.94	0.31	CLDN7
Primary colon metastasis (Colon_M)	3	9938065	9959891	0.17	0.47	0.31	IL17RC
Primary colon metastasis (Colon_M)	17	81030950	81060926	0.29	0.59	0.30	METRNL
Primary colon metastasis (Colon_M)	2	200306225	200342782	0.56	0.86	0.30	SATB2
Primary colon metastasis (Colon_M)	19	49115902	49124026	0.39	0.68	0.30	SPHK2
Primary colon metastasis (Colon_M)	1	211499374	211527884	0.09	0.38	0.30	TRAF5
Primary colon metastasis (Colon_M)	12	6478956	6487347	0.24	0.53	0.29	LTBR
Primary colon metastasis (Colon_M)	12	89739271	89785574	0.15	0.44	0.29	DUSP6
Primary colon metastasis (Colon_M)	20	62312602	62332044	0.16	0.45	0.29	TNFRSF6B
Primary colon metastasis (Colon_M)	11	307666	312937	0.70	0.98	0.28	IFITM2
Primary colon metastasis (Colon_M)	5	175959707	175981606	0.36	0.64	0.28	CDHR2
Primary colon metastasis (Colon_M)	21	43876507	43895539	0.03	0.31	0.28	SLC37A1
Primary colon metastasis (Colon_M)	19	54970768	54983186	0.35	0.63	0.28	LENG9
Primary colon metastasis (Colon_M)	1	226052374	226087213	0.19	0.47	0.27	LEFTY1
Primary colon metastasis (Colon_M)	15	31546396	31565926	0.03	0.31	0.27	KLF13
Primary colon metastasis (Colon_M)	14	55558308	55599128	0.10	0.37	0.27	LGALS3
Primary colon metastasis (Colon_M)	2	145252863	145282644	0.47	0.74	0.27	ZEB2
Primary colon metastasis (Colon_M)	1	46632072	46655214	0.08	0.34	0.26	TSPAN1
Primary colon metastasis (Colon_M)	15	63333068	63347067	0.46	0.71	0.26	TPM1
Primary colon metastasis (Colon_M)	15	45726853	45749919	0.09	0.35	0.26	C15orf48
Primary colon metastasis (Colon_M)	11	1865282	1903941	0.12	0.38	0.26	LSP1
Primary colon metastasis (Colon_M)	14	50465553	50471375	0.66	0.92	0.25	C14orf182

Primary colon metastasis (Colon_M)	22	37406895	37441393	0.08	0.33	0.25	MPST
Primary colon metastasis (Colon_M)	2	47563284	47609335	0.08	0.33	0.25	EPCAM
Primary colon metastasis (Colon_M)	17	76402998	76414113	0.00	0.25	0.25	PGS1
Glioblastoma (U87MG)	15	77985239	78036242	0.02	1.00	0.98	LINGO1
Glioblastoma (U87MG)	1	112417478	112468665	0.03	1.00	0.97	KCND3
Glioblastoma (U87MG)	17	9811786	9891020	0.02	0.99	0.97	GAS7
Glioblastoma (U87MG)	11	89364627	89408904	0.00	0.97	0.97	NOX4
Glioblastoma (U87MG)	4	16653926	16697144	0.00	0.97	0.96	LDB2
Glioblastoma (U87MG)	11	7592529	7626823	0.06	1.00	0.94	PPFIBP2
Glioblastoma (U87MG)	9	19549453	19728789	0.03	0.97	0.94	SLC24A2
Glioblastoma (U87MG)	9	19549453	19728789	0.03	0.97	0.94	SLC24A2
Glioblastoma (U87MG)	6	97413825	97477513	0.03	0.96	0.93	KLHL32
Glioblastoma (U87MG)	17	9903813	9965252	0.05	0.98	0.93	GAS7
Glioblastoma (U87MG)	2	21473055	21505937	0.06	0.99	0.93	TDRD15
Glioblastoma (U87MG)	3	35676346	35724308	0.05	0.97	0.92	ARPP21
Glioblastoma (U87MG)	15	32065846	32164147	0.06	0.98	0.91	OTUD7A
Glioblastoma (U87MG)	11	119942989	119964740	0.06	0.97	0.91	TRIM29
Glioblastoma (U87MG)	9	137967864	138040365	0.09	0.99	0.90	OLFM1
Glioblastoma (U87MG)	4	7342499	7398832	0.09	0.99	0.90	SORCS2
Glioblastoma (U87MG)	10	1224882	1259633	0.08	0.97	0.89	ADARB2
Glioblastoma (U87MG)	11	83518536	83549646	0.00	0.89	0.89	DLG2
Glioblastoma (U87MG)	20	58177200	58286056	0.10	0.99	0.89	PHACTR3
Glioblastoma (U87MG)	7	154558028	154588504	0.08	0.97	0.89	DPP6
Glioblastoma (U87MG)	8	143575390	143626966	0.10	0.97	0.88	BAI1
Glioblastoma (U87MG)	11	49190901	49236799	0.04	0.92	0.88	FOLH1
Glioblastoma (U87MG)	5	175104513	175139823	0.03	0.90	0.88	HRH2
Glioblastoma (U87MG)	19	57200811	57239668	0.06	0.93	0.87	ZNF835
Glioblastoma (U87MG)	11	7650333	7699694	0.06	0.93	0.87	CYB5R2
Glioblastoma (U87MG)	1	110693399	110756870	0.07	0.94	0.87	KCNC4
Glioblastoma (U87MG)	11	117399960	117681020	0.12	0.99	0.87	DSCAML1
Glioblastoma (U87MG)	1	111123465	111163650	0.13	1.00	0.87	KCNA2
Glioblastoma (U87MG)	1	4712562	4775202	0.12	0.99	0.87	AJAP1
Glioblastoma (U87MG)	11	83939015	84004173	0.03	0.89	0.86	DLG2
Glioblastoma (U87MG)	4	42605460	42661658	0.12	0.98	0.86	ATP8A1
Glioblastoma (U87MG)	4	6440647	6473745	0.14	1.00	0.86	PPP2R2C
Glioblastoma (U87MG)	11	119843908	119906650	0.11	0.97	0.86	TRIM29
Glioblastoma (U87MG)	4	7296865	7329947	0.06	0.91	0.85	SORCS2
Glioblastoma (U87MG)	4	7296865	7329947	0.06	0.91	0.85	SORCS2
Glioblastoma (U87MG)	11	117721606	117754344	0.17	1.00	0.83	FXYD6

Glioblastoma (U87MG)	20	37432328	37531273	0.11	0.94	0.82	PPP1R16B
Glioblastoma (U87MG)	5	743055	802933	0.08	0.90	0.82	ZDHHC11B
Glioblastoma (U87MG)	11	64379909	64428733	0.07	0.89	0.82	NRXN2
Glioblastoma (U87MG)	11	133874209	133916196	0.12	0.93	0.81	JAM3
Glioblastoma (U87MG)	11	133874209	133916196	0.12	0.93	0.81	JAM3
Glioblastoma (U87MG)	6	97344914	97390757	0.16	0.96	0.80	KLHL32
Glioblastoma (U87MG)	11	114090121	114132225	0.07	0.86	0.80	NNMT
Glioblastoma (U87MG)	7	51292392	51394212	0.18	0.97	0.80	COBL
Glioblastoma (U87MG)	2	60731415	60797670	0.15	0.95	0.80	BCL11A
Glioblastoma (U87MG)	19	29973373	30022034	0.17	0.96	0.79	VSTM2B
Glioblastoma (U87MG)	3	118677505	118754932	0.10	0.89	0.79	IGSF11
Glioblastoma (U87MG)	9	19747340	19790384	0.15	0.94	0.78	SLC24A2
Glioblastoma (U87MG)	11	131430276	131505034	0.15	0.92	0.78	NTM
Glioblastoma (U87MG)	13	113650879	113757822	0.08	0.85	0.77	AL137002.1
Glioblastoma (U87MG)	15	77895466	77967694	0.09	0.84	0.75	LINGO1
Glioblastoma (U87MG)	17	72226877	72248627	0.23	0.98	0.75	TTYH2
Glioblastoma (U87MG)	1	160019557	160042341	0.23	0.98	0.75	KCNJ10
Glioblastoma (U87MG)	9	8840393	8880342	0.24	0.98	0.74	PTPRD
Glioblastoma (U87MG)	9	19457142	19518307	0.09	0.83	0.74	ACER2
Glioblastoma (U87MG)	20	45187487	45213480	0.22	0.96	0.74	SLC13A3
Glioblastoma (U87MG)	8	9753618	9790735	0.15	0.89	0.74	MSRA
Glioblastoma (U87MG)	11	113926954	114070258	0.08	0.81	0.73	ZBTB16
Glioblastoma (U87MG)	11	134254603	134298147	0.26	0.99	0.73	B3GAT1
Glioblastoma (U87MG)	13	25710619	25792315	0.15	0.86	0.71	AMER2
Glioblastoma (U87MG)	4	36164927	36295153	0.11	0.82	0.71	DTHD1
Glioblastoma (U87MG)	3	11024732	11080079	0.23	0.94	0.71	SLC6A1
Glioblastoma (U87MG)	11	64441886	64486253	0.13	0.83	0.70	NRXN2
Glioblastoma (U87MG)	8	27089725	27139876	0.09	0.77	0.67	STMN4
Glioblastoma (U87MG)	5	141125726	141169237	0.03	0.70	0.67	ARAP3
Glioblastoma (U87MG)	6	166689003	166721330	0.20	0.87	0.67	PRR18
Glioblastoma (U87MG)	10	88016137	88053122	0.07	0.73	0.66	GRID1
Glioblastoma (U87MG)	8	143531641	143555608	0.30	0.96	0.65	BAI1
Glioblastoma (U87MG)	12	110034532	110070399	0.16	0.81	0.65	MVK
Glioblastoma (U87MG)	16	934803	971974	0.12	0.76	0.64	LMF1
Glioblastoma (U87MG)	10	88108134	88126246	0.29	0.94	0.64	GRID1
Glioblastoma (U87MG)	16	5005930	5093187	0.14	0.77	0.63	ALG1
Glioblastoma (U87MG)	10	98094383	98136176	0.18	0.80	0.62	OPALIN
Glioblastoma (U87MG)	16	1064221	1098971	0.24	0.85	0.61	SSTR5
Glioblastoma (U87MG)	17	3402742	3440702	0.12	0.70	0.58	TRPV3

Glioblastoma (U87MG)	1	204796255	204866707	0.21	0.79	0.58	NFASC
Glioblastoma (U87MG)	2	105022517	105068208	0.24	0.82	0.58	POU3F3
Glioblastoma (U87MG)	19	31043362	31082430	0.06	0.62	0.56	ZNF536
Glioblastoma (U87MG)	19	51033029	51070726	0.30	0.86	0.56	LRRC4B
Glioblastoma (U87MG)	3	39487852	39573984	0.34	0.88	0.54	MOBP
Glioblastoma (U87MG)	17	26265479	26324131	0.04	0.57	0.54	LYRM9
Glioblastoma (U87MG)	7	86272604	86301105	0.22	0.75	0.53	GRM3
Glioblastoma (U87MG)	3	10505723	10550300	0.05	0.56	0.51	ATP2B2
Glioblastoma (U87MG)	20	24441648	24488844	0.15	0.66	0.51	SYNDIG1
Glioblastoma (U87MG)	6	110640550	110689914	0.02	0.53	0.51	METTL24
Glioblastoma (U87MG)	21	43639119	43683392	0.22	0.71	0.49	ABCG1
Glioblastoma (U87MG)	15	89891804	89952836	0.35	0.82	0.47	POLG
Glioblastoma (U87MG)	6	69337682	69367340	0.33	0.79	0.46	BAI3
Glioblastoma (U87MG)	1	175825389	175858645	0.12	0.57	0.45	RFWD2
Glioblastoma (U87MG)	11	72284488	72365197	0.06	0.49	0.43	PDE2A
Glioblastoma (U87MG)	11	1405512	1442659	0.20	0.63	0.43	BRSK2
Glioblastoma (U87MG)	3	133460192	133484110	0.24	0.67	0.42	TF
Glioblastoma (U87MG)	1	156353833	156405678	0.29	0.70	0.41	C1orf61
Glioblastoma (U87MG)	15	45655307	45688573	0.24	0.65	0.41	GATM
Glioblastoma (U87MG)	14	78431807	78458522	0.14	0.54	0.41	ADCK1
Glioblastoma (U87MG)	1	204888687	205049666	0.13	0.51	0.38	CNTN2
Glioblastoma (U87MG)	2	164566271	164594418	0.35	0.72	0.36	FIGN
Glioblastoma (U87MG)	X	103025990	103049724	0.64	1.00	0.36	PLP1
Glioblastoma (U87MG)	1	200978848	201012524	0.14	0.49	0.35	KIF21B
Glioblastoma (U87MG)	12	113534226	113581503	0.11	0.46	0.35	RASAL1
Glioblastoma (U87MG)	11	131522182	131565052	0.19	0.53	0.34	NTM
Glioblastoma (U87MG)	8	53318133	53342376	0.29	0.62	0.34	ST18
Glioblastoma (U87MG)	13	113365298	113413184	0.12	0.45	0.33	ATP11A
Glioblastoma (U87MG)	7	37232585	37330045	0.11	0.44	0.32	ELMO1
Glioblastoma (U87MG)	4	17510272	17521459	0.21	0.53	0.32	CLRN2
Glioblastoma (U87MG)	8	65275409	65295224	0.24	0.55	0.31	BHLHE22
Glioblastoma (U87MG)	15	84214391	84255934	0.08	0.38	0.30	SH3GL3
Glioblastoma (U87MG)	3	42724031	42791782	0.06	0.36	0.30	CCDC13
Glioblastoma (U87MG)	1	20755054	20821566	0.19	0.48	0.30	CAMK2N1
Glioblastoma (U87MG)	21	34399873	34453980	0.28	0.57	0.29	OLIG1
Glioblastoma (U87MG)	11	133987173	134023589	0.17	0.46	0.28	JAM3
Glioblastoma (U87MG)	19	50985631	51019282	0.28	0.53	0.25	ASPDH

Supplementary Table 6: GO enrichment analysis of hypermethylated super-enhancers (GOTERM_BP_5, p<0.01).

GO term	# genes	% genes	Enrichment	p-value*	Sample**
Regulation of gene expression	16	57,1	3,6	8,7E-6	468PT
Regulation of transcription	16	57,1	4,0	9,1E-6	468PT
Regulation of macromolecule biosynthetic process	16	57,1	3,7	1,0E-5	468PT
Regulation of cellular biosynthetic process	16	57,1	3,5	1,1E-5	468PT
Regulation of nucleobase, nucleoside, nucleotide and nucleic acid metabolic process	16	57,1	3,7	1,4E-5	468PT
Regulation of transcription, DNA-dependent	13	46,4	4,7	3,0E-5	468PT
Regulation of RNA metabolic process	13	46,4	4,6	3,3E-5	468PT
Regulation of RNA metabolic process	18	35,3	3,4	6,1E-4	468LN
Vasculature development	8	15,7	10,9	7,9E-4	468LN
Regulation of transcription, DNA-dependent	18	35,3	3,5	8,9E-4	468LN
Blood vessel development	8	15,7	11,1	9,0E-4	468LN
Blood vessel morphogenesis	7	13,7	11,3	2,9E-3	468LN
Gland development	6	11,8	15,2	3,0E-3	468LN
Chordate embryonic development	8	15,7	8,3	3,2E-3	468LN
Angiogenesis	6	11,8	13,8	4,0E-3	468LN
Regulation of transcription	19	37,3	2,5	4,6E-3	468LN
Regulation of cellular biosynthetic process	20	39,2	2,3	6,0E-3	468LN
Regulation of gene expression	16	76,2	3,6	6,5E-6	H157
Regulation of transcription	16	76,2	4,0	6,8E-6	H157
Regulation of macromolecule biosynthetic process	16	76,2	3,7	7,6E-6	H157
Regulation of cellular biosynthetic process	16	76,2	3,5	8,1E-6	H157
Regulation of nucleobase, nucleoside, nucleotide and nucleic acid metabolic process	16	76,2	3,7	1,0E-5	H157
Regulation of transcription, DNA-dependent	12	57,1	4,4	2,4E-4	H157
Regulation of RNA metabolic process	12	57,1	4,3	2,5E-4	H157
Regulation of transcription	19	51,4	3,1	3,1E-4	H1672
Regulation of gene expression	19	51,4	2,8	3,2E-4	H1672
Regulation of transcription, DNA-dependent	15	40,5	3,6	3,4E-4	H1672
Positive Regulation of RNA metabolic process	9	24,3	7,9	3,6E-4	H1672
Regulation of macromolecule biosynthetic process	19	51,4	2,8	3,8E-4	H1672
Positive Regulation of transcription, DNA-dependent	9	24,3	8,0	3,9E-4	H1672
Regulation of RNA metabolic process	15	40,5	3,5	3,9E-4	H1672
Regulation of cellular biosynthetic process	19	51,4	2,7	4,2E-4	H1672
Regulation of nucleobase, nucleoside, nucleotide and nucleic acid metabolic process	19	51,4	2,9	5,2E-4	H1672
Positive Regulation of transcription	9	24,3	6,8	8,0E-4	H1672
Positive Regulation of gene expression	9	24,3	6,6	9,0E-4	H1672

Positive Regulation of nucleobase, nucleoside, nucleotide and nucleic acid metabolic process	9	24,3	6,1	1,4E-3	H1672
Positive Regulation of nitrogen compound metabolic process	9	24,3	5,9	1,6E-3	H1672
Positive Regulation of macromolecule biosynthetic process	9	24,3	5,8	1,6E-3	H1672
Positive Regulation of cellular biosynthetic process	9	24,3	5,6	2,1E-3	H1672
Positive Regulation of biosynthetic process	9	24,3	5,5	2,2E-3	H1672
Regulation of striated muscle tissue development	4	10,8	33,9	3,5E-3	H1672
Positive Regulation of macromolecule metabolic process	9	24,3	4,5	8,2E-3	H1672
Tissue development	8	21,6	5,1	8,8E-3	H1672
Positive Regulation of cellular metabolic process	9	24,3	4,3	9,3E-3	H1672

*** Bonferroni corrected**
**** Only samples with significantly enriched GO terms**

Supplementary Table 7: Validation of hypermethylated super-enhancers in cancer samples (HumanMethylation450 BeadChip) .

	Super-enhancer					Validation set		WGBS	CNV
**Cancer type**	**ID**	**Gene**	**Chr.**	**Start**	**End**	**δ AVR**	**FDR**	**δ HMR**	**FDR**
Breast cancer	1_MACS_peak_2257_lociStitched	CKS1B	1	154941228	154949531	0.05	9.25E-08	0.34	9.97E-01
Breast cancer	2_MACS_peak_23111_lociStitched	RBMS3	3	29321550	29334286	0.06	9.30E-06	0.65	9.97E-01
Breast cancer	2_MACS_peak_23764_lociStitched	FOXP1	3	71104592	71120542	0.01	4.33E-01	0.28	9.97E-01
Breast cancer	1_MACS_peak_27137_lociStitched	IRX4	5	1881509	1890183	0.08	1.20E-04	0.67	9.97E-01
Breast cancer	3_MACS_peak_27146_lociStitched	IRX2	5	2739262	2759651	0.03	4.58E-02	0.60	9.97E-01
Breast cancer	1_MACS_peak_28648_lociStitched	NR3C1	5	142773722	142785691	0.09	8.80E-12	0.63	9.97E-01
Breast cancer	1_MACS_peak_31912_lociStitched	INHBA	7	41734099	41745805	0.06	1.94E-06	0.34	9.97E-01
Breast cancer	6_MACS_peak_5342_lociStitched	FAM53B	10	126406768	126435377	0.01	1.84E-01	0.26	9.97E-01
Breast cancer	1_MACS_peak_5527_lociStitched	PRKCDBP	11	6339234	6344196	0.10	1.04E-08	0.32	9.97E-01
Breast cancer	3_MACS_peak_7389_lociStitched	TNFRSF1A	12	6432600	6452223	0.01	5.72E-03	0.29	9.97E-01
Breast cancer	2_MACS_peak_8659_lociStitched	BTG1	12	92527863	92539838	0.03	1.27E-05	0.35	9.97E-01
Breast cancer	4_MACS_peak_10930_lociStitched	ELMSAN1	14	74207241	74227902	-0.01	7.93E-02	0.36	9.97E-01
Breast cancer	1_MACS_peak_11720_lociStitched	GABPB1	15	50644604	50649357	0.00	4.10E-02	0.29	2.33E-01
Breast cancer	MACS_peak_12476	CHD2	15	93425946	93432559	0.01	2.01E-03	0.31	9.97E-01
Breast cancer	3_MACS_peak_17254_lociStitched	CIC	19	42771661	42788152	0.03	5.75E-12	0.27	9.97E-01
Breast cancer	1_MACS_peak_21219_lociStitched	RBM39	20	34326330	34331841	0.00	5.85E-04	0.30	9.97E-01
Breast cancer	2_MACS_peak_21515_lociStitched	TSHZ2	20	51581498	51597746	0.06	7.91E-08	0.37	9.97E-01
Breast cancer	1_MACS_peak_21859_lociStitched	RUNX1	21	36252652	36264673	0.04	2.53E-03	0.42	9.97E-01
Breast cancer	MACS_peak_22783	MIRLET7	22	46467114	46488203	0.16	2.61E-07	0.61	2.33E-01
Colorectal cancer	3_MACS_peak_30801_lociStitched	PITX1	5	134361067	134376085	0.09	2.24E-06	0.29	8.18E-01
Glioblastoma	13_MACS_peak_22858_lociStitched	BIN1	2	127806748	127912517	0.06	1.66E-02	0.32	5.54E-01
Glioblastoma	15_MACS_peak_22991_lociStitched	ZEB2	2	145129528	145283099	0.00	8.77E-01	0.34	9.23E-01
Glioblastoma	6_MACS_peak_23868_lociStitched	PTMA	2	232526039	232554844	0.07	6.33E-04	0.29	9.23E-01
Glioblastoma	8_MACS_peak_29107_lociStitched	SOX2	3	181403893	181455295	0.01	8.20E-01	0.36	4.22E-01
Glioblastoma	1_MACS_peak_31287_lociStitched	SLC1A3	5	36599886	36615091	0.00	8.95E-01	0.33	3.74E-02
Glioblastoma	MACS_peak_32260	SEPT8	5	132105879	132116081	0.05	1.29E-02	0.27	9.71E-01
Glioblastoma	2_MACS_peak_34748_lociStitched	SGK1	6	134488737	134505092	0.06	2.19E-02	0.38	9.73E-01
Glioblastoma	12_MACS_peak_35205_lociStitched	QKI	6	163815822	163882069	0.01	8.17E-01	0.65	9.71E-01
Glioblastoma	5_MACS_peak_40731_lociStitched	RALGDS	9	135976307	136024639	0.02	1.30E-01	0.26	9.23E-01
Glioblastoma	1_MACS_peak_40902_lociStitched	TPRN	9	140081775	140094696	0.05	3.59E-03	0.35	9.23E-01
Glioblastoma	11_MACS_peak_5386_lociStitched	ZCCHC24	10	81137824	81212605	0.04	6.60E-03	0.38	9.23E-01
Glioblastoma	3_MACS_peak_5703_lociStitched	LZTS2	10	102752997	102775915	0.04	3.35E-02	0.28	4.22E-01
Glioblastoma	3_MACS_peak_7435_lociStitched	CARNS1	11	67173218	67188540	0.07	5.40E-05	0.38	9.23E-01
Glioblastoma	MACS_peak_9242	AC004466.1	12	48167031	48182319	0.00	7.19E-01	0.35	9.23E-01
Glioblastoma	3_MACS_peak_11368_lociStitched	PCDH9	13	67782754	67806323	0.01	8.20E-01	0.45	5.54E-01

Glioblastoma	1_MACS_peak_12563_lociStitched	GSTZ1	14	77765872	77789479	0.02	2.56E-01	0.28	9.58E-01
Glioblastoma	4_MACS_peak_13804_lociStitched	OAZ2	15	64974641	64996542	0.02	2.43E-02	0.30	9.23E-01
Glioblastoma	MACS_peak_17165	THRA	17	38216903	38234316	0.01	7.56E-01	0.36	9.23E-01
Glioblastoma	4_MACS_peak_17901_lociStitched	KCNJ2	17	68152783	68183190	0.00	7.48E-01	0.29	9.23E-01
Glioblastoma	5_MACS_peak_18355_lociStitched	BAHCC1	17	79345155	79376776	-0.01	2.32E-01	0.27	9.23E-01
Glioblastoma	3_MACS_peak_26411_lociStitched	CDC42EP1	22	37940770	37968154	0.06	5.93E-04	0.32	9.23E-01
Lung adenocarcinoma	MACS_peak_3232	TXNIP	1	145434374	145443479	0.00	8.52E-01	0.48	5.29E-01
Lung adenocarcinoma	3_MACS_peak_30019_lociStitched	MEIS1	2	66659175	66674430	0.14	4.25E-18	0.45	no CNV
Lung adenocarcinoma	2_MACS_peak_39322_lociStitched	GATA2	3	128199015	128216666	0.08	2.96E-08	0.53	no CNV
Lung adenocarcinoma	4_MACS_peak_42669_lociStitched	HAND2	4	174427206	174460857	0.12	3.22E-12	0.48	no CNV
Lung adenocarcinoma	5_MACS_peak_42987_lociStitched	IRX2	5	2738590	2759482	0.07	1.60E-05	0.74	5.29E-01
Lung adenocarcinoma	5_MACS_peak_45552_lociStitched	NKX2-5	5	172655376	172676296	0.09	4.62E-08	0.58	no CNV
Lung adenocarcinoma	MACS_peak_54700	CREB3	9	35726029	35733751	-0.01	2.73E-02	0.31	NA
Lung adenocarcinoma	3_MACS_peak_7162_lociStitched	ZNF503-AS2	10	77154286	77170840	0.02	2.08E-02	0.26	no CNV
Lung adenocarcinoma	MACS_peak_17492	CRIP2	14	105938037	105944737	0.00	6.84E-01	0.34	no CNV
Lung adenocarcinoma	MACS_peak_22262	ZBTB4	17	7378645	7384073	0.01	1.38E-01	0.36	no CNV
Lung adenocarcinoma	MACS_peak_36631	MIRLET7	22	46445515	46454339	0.02	1.82E-01	0.29	NA
Lung adenocarcinoma	MACS_peak_36635	MIRLET7	22	46464333	46478321	0.03	1.82E-02	0.27	NA
Lung squamous cell carcinoma	MACS_peak_3232	TXNIP	1	145434374	145443479	0.02	1.32E-01	0.48	no CNV
Lung squamous cell carcinoma	2_MACS_peak_28599_lociStitched	ID2	2	8815972	8826211	0.00	5.29E-01	0.59	9.86E-07
Lung squamous cell carcinoma	3_MACS_peak_30019_lociStitched	MEIS1	2	66659175	66674430	0.11	4.14E-13	0.52	1.91E-01
Lung squamous cell carcinoma	3_MACS_peak_32032_lociStitched	CLK1	2	201724772	201732443	0.02	5.41E-06	0.35	4.78E-02
Lung squamous cell carcinoma	2_MACS_peak_37947_lociStitched	SHISA5	3	48504418	48515502	0.01	7.22E-01	0.26	2.92E-01
Lung squamous cell carcinoma	2_MACS_peak_39322_lociStitched	GATA2	3	128199015	128216666	0.08	5.59E-07	0.63	1.91E-01
Lung squamous cell carcinoma	4_MACS_peak_42669_lociStitched	HAND2	4	174427206	174460857	0.13	6.38E-14	0.67	NA
Lung squamous cell carcinoma	5_MACS_peak_42987_lociStitched	IRX2	5	2738590	2759482	0.15	6.38E-14	0.63	NA
Lung squamous cell carcinoma	4_MACS_peak_43416_lociStitched	ANXA2R	5	42990572	43020949	0.09	2.87E-07	0.26	2.79E-02
Lung squamous cell carcinoma	MACS_peak_54700	CREB3	9	35726029	35733751	-0.02	4.14E-03	0.32	1.15E-01
Lung squamous cell carcinoma	3_MACS_peak_7162_lociStitched	ZNF503-AS2	10	77154286	77170840	0.04	1.91E-05	0.38	1.91E-01
Lung squamous cell carcinoma	1_MACS_peak_11040_lociStitched	DDX6	11	118659939	118664216	0.01	5.77E-01	0.38	3.75E-01
Lung squamous cell carcinoma	MACS_peak_11710	TNFRSF1A	12	6441703	6453465	0.02	1.82E-01	0.39	NA
Lung squamous cell carcinoma	MACS_peak_11762	ATN1	12	7032586	7039696	0.00	5.35E-01	0.34	NA
Lung squamous cell carcinoma	5_MACS_peak_13097_lociStitched	BTG1	12	92526891	92540701	0.00	9.18E-01	0.43	NA
Lung squamous cell carcinoma	6_MACS_peak_13707_lociStitched	TBX3	12	115105978	115131359	0.08	3.09E-10	0.36	NA
Lung squamous cell carcinoma	7_MACS_peak_15952_lociStitched	NKX2-1	14	36971679	36995027	0.13	7.34E-12	0.49	2.92E-01
Lung squamous cell carcinoma	MACS_peak_17492	CRIP2	14	105938037	105944737	0.00	4.53E-01	0.32	no CNV
Lung squamous cell carcinoma	4_MACS_peak_20804_lociStitched	IRX5	16	54956715	54973307	0.09	1.03E-11	0.32	NA
Lung squamous cell carcinoma	MACS_peak_22262	ZBTB4	17	7378645	7384073	0.02	2.86E-03	0.37	NA
Lung squamous cell carcinoma	MACS_peak_22267	SENP3	17	7459853	7466333	0.01	1.78E-01	0.26	NA

Lung squamous cell carcinoma	MACS_peak_23136	THRA	17	38222943	38234141	0.06	3.97E-05	0.35	no CNV
Lung squamous cell carcinoma	MACS_peak_36631	MIRLET7	22	46445515	46454339	0.01	4.99E-01	0.26	NA
Lung squamous cell carcinoma	MACS_peak_36635	MIRLET7	22	46464333	46478321	0.03	3.82E-02	0.32	NA
Small cell lung cancer	MACS_peak_42	VWA1	1	1364851	1372426	0.02	4.71E-01	0.32	9.12E-01
Small cell lung cancer	MACS_peak_3232	TXNIP	1	145434374	145443479	0.01	5.20E-01	0.29	2.81E-01
Small cell lung cancer	MACS_peak_3436	S100A2	1	153537691	153541885	-0.03	3.10E-02	0.32	4.02E-01
Small cell lung cancer	3_MACS_peak_30019_lociStitched	MEIS1	2	66659175	66674430	0.19	1.70E-24	0.45	9.12E-01
Small cell lung cancer	3_MACS_peak_32032_lociStitched	CLK1	2	201724772	201732443	0.01	2.01E-03	0.35	4.52E-01
Small cell lung cancer	2_MACS_peak_39322_lociStitched	GATA2	3	128199015	128216666	0.09	8.82E-07	0.50	9.12E-01
Small cell lung cancer	4_MACS_peak_42669_lociStitched	HAND2	4	174427206	174460857	0.17	1.74E-15	0.50	9.12E-01
Small cell lung cancer	5_MACS_peak_42987_lociStitched	IRX2	5	2738590	2759482	0.13	3.90E-10	0.35	9.12E-01
Small cell lung cancer	5_MACS_peak_45552_lociStitched	NKX2-5	5	172655376	172676296	0.14	2.02E-11	0.26	7.99E-01
Small cell lung cancer	2_MACS_peak_46644_lociStitched	NRM	6	30646634	30659820	0.04	8.43E-04	0.39	1.42E-02
Small cell lung cancer	2_MACS_peak_46715_lociStitched	CLIC1	6	31694582	31706840	0.02	7.73E-02	0.30	8.38E-01
Small cell lung cancer	1_MACS_peak_49158_lociStitched	MAFK	7	1569896	1580027	0.01	6.46E-01	0.28	9.12E-01
Small cell lung cancer	4_MACS_peak_54476_lociStitched	NFIB	9	14302438	14323420	0.01	3.41E-01	0.28	1.57E-01
Small cell lung cancer	4_MACS_peak_54483_lociStitched	NFIB	9	14340900	14353877	0.06	7.73E-02	0.25	9.12E-01
Small cell lung cancer	MACS_peak_54700	CREB3	9	35726029	35733751	-0.01	4.66E-01	0.36	9.12E-01
Small cell lung cancer	3_MACS_peak_7162_lociStitched	ZNF503-AS2	10	77154286	77170840	0.04	7.31E-05	0.29	7.99E-01
Small cell lung cancer	MACS_peak_7364	C10orf116	10	88726022	88733482	0.01	6.46E-01	0.27	2.39E-01
Small cell lung cancer	MACS_peak_9994	FRMD8	11	65182856	65196355	0.02	1.82E-01	0.76	4.52E-01
Small cell lung cancer	MACS_peak_10139	TCIRG1	11	67803279	67809641	0.01	6.09E-01	0.37	1.57E-01
Small cell lung cancer	1_MACS_peak_11040_lociStitched	DDX6	11	118659939	118664216	0.01	1.50E-01	0.35	9.12E-01
Small cell lung cancer	2_MACS_peak_11044_lociStitched	UPK2	11	118777304	118801687	0.00	8.56E-01	0.46	6.46E-01
Small cell lung cancer	MACS_peak_11710	TNFRSF1A	12	6441703	6453465	0.02	2.30E-02	0.61	5.80E-01
Small cell lung cancer	5_MACS_peak_13097_lociStitched	BTG1	12	92526891	92540701	0.00	9.62E-01	0.38	9.81E-01
Small cell lung cancer	9_MACS_peak_13696_lociStitched	TBX5	12	114806250	114852976	0.14	6.63E-10	0.43	7.99E-01
Small cell lung cancer	6_MACS_peak_13707_lociStitched	TBX3	12	115105978	115131359	0.07	9.04E-07	0.28	9.12E-01
Small cell lung cancer	7_MACS_peak_15952_lociStitched	NKX2-1	14	36971679	36995027	0.09	2.49E-05	0.69	9.12E-01
Small cell lung cancer	MACS_peak_17492	CRIP2	14	105938037	105944737	-0.01	5.23E-01	0.47	9.81E-01
Small cell lung cancer	4_MACS_peak_20804_lociStitched	IRX5	16	54956715	54973307	0.08	2.07E-09	0.40	9.12E-01
Small cell lung cancer	1_MACS_peak_20826_lociStitched	MT2A	16	56638742	56646907	0.04	3.34E-02	0.25	9.12E-01
Small cell lung cancer	MACS_peak_22262	ZBTB4	17	7378645	7384073	0.05	6.28E-07	0.36	7.24E-01
Small cell lung cancer	MACS_peak_23136	THRA	17	38222943	38234141	0.03	1.15E-01	0.48	2.39E-01
Small cell lung cancer	1_MACS_peak_24752_lociStitched	ACTG1	17	79475627	79487805	-0.01	9.09E-02	0.36	9.12E-01
Small cell lung cancer	3_MACS_peak_24980_lociStitched	DLGAP1	18	3591992	3606653	-0.01	6.37E-01	0.31	3.82E-01
Small cell lung cancer	MACS_peak_26165	MIDN	19	1247145	1256325	0.02	9.48E-03	0.30	no CNV
Small cell lung cancer	MACS_peak_26166	CIRBP	19	1256391	1263727	-0.01	5.54E-01	0.34	NA
Small cell lung cancer	MACS_peak_27682	ITPKC	19	41219787	41228823	-0.02	1.21E-02	0.29	no CNV

Small cell lung cancer	6_MACS_peak_33435_lociStitched	FOXA2	20	22536539	22565230	0.09	5.88E-06	0.51	8.38E-01
Small cell lung cancer	MACS_peak_36245	CSNK1E	22	38708087	38715695	0.00	8.64E-01	0.31	9.81E-01
Small cell lung cancer	MACS_peak_36631	MIRLET7	22	46445515	46454339	-0.02	2.34E-01	0.45	9.12E-01
Small cell lung cancer	MACS_peak_36635	MIRLET7	22	46464333	46478321	-0.01	7.51E-01	0.47	7.88E-01

Supplementary Table 8: Hypomethylated cancer-related super-enhancers in colorectal cancer cells.

Enhancer					% DNA hypomethylation			450K (AVR)			CNV	CNV	#TFBS
Chr.	Start	End	Enhancer ID (Hnisz et al., 2013)	Gene symbol	Normal	Tumor	Delta	Normal	Tumor	-log10	FDR	WGBS	FOXQ1
1	1079431	1101911	7_MACS_peak_34_lociStitched	MIR200b	0.21	0.51	-0.31	0.56	0.36	-10.82	NA	loss	18
1	1365609	1378027	3_MACS_peak_73_lociStitched	VWA1	0.28	0.75	-0.47	NA	NA	NA	NA	loss	0
1	45270646	45276647	MACS_peak_1200	PLK3	0.18	0.52	-0.34	0.22	0.34	3.63	NA	NA	7
2	70310047	70317059	2_MACS_peak_15169_lociStitched	PCBP1	0.54	0.95	-0.40	0.54	0.40	-9.97	NA	NA	0
3	49934498	49944642	2_MACS_peak_18795_lociStitched	MST1R	0.09	0.57	-0.48	0.40	0.22	-8.11	0.79	NA	7
7	130563226	130576863	MACS_peak_24364	MIR29a/b	0.00	0.27	-0.27	0.74	0.36	-8.83	NA	NA	7
7	130577071	130604705	MACS_peak_24365	MIR29a/b	0.21	0.95	-0.74	0.55	0.31	-5.29	0.71	NA	11
8	128744519	128756323	2_MACS_peak_25390_lociStitched	MYC	0.53	0.83	-0.30	0.46	0.31	-2.65	0.71	NA	5
10	101664881	101695667	4_MACS_peak_3541_lociStitched	DNMBP	0.02	0.30	-0.28	NA	NA	NA	0.71	NA	22
11	309864	321533	2_MACS_peak_3964_lociStitched	IFITM3	0.69	0.94	-0.25	0.40	0.39	-0.20	NA	NA	2
12	7069196	7075991	MACS_peak_5450	MIR200c	0.39	0.73	-0.34	0.68	0.31	-9.54	NA	NA	4
12	13022807	13063891	9_MACS_peak_5515_lociStitched	GPRC5A	0.05	0.38	-0.33	0.44	0.44	-0.11	NA	NA	35
14	69245317	69265057	3_MACS_peak_7661_lociStitched	ZFP36L1	0.47	0.80	-0.33	NA	NA	NA	0.64	NA	9
14	105552749	105561461	1_MACS_peak_8032_lociStitched	GPR123	0.53	0.81	-0.28	NA	NA	NA	NA	loss	4
16	1137094	1142491	MACS_peak_8969	C1QTNF8	0.41	0.71	-0.30	NA	NA	NA	NA	loss	6
16	89622859	89633240	MACS_peak_10212	SNORD68	0.39	0.67	-0.28	0.51	0.51	-0.38	NA	NA	6
17	56428488	56496439	10_MACS_peak_11452_lociStitched	RNF43	0.11	0.58	-0.47	0.52	0.44	-5.29	NA	NA	51
17	75275055	75285678	1_MACS_peak_11905_lociStitched	SEPT9	0.27	0.73	-0.46	NA	NA	NA	NA	NA	5
17	79301530	79306049	MACS_peak_12086	TMEM105	0.24	0.56	-0.31	0.52	0.60	1.54	NA	loss	0
20	62305260	62334757	6_MACS_peak_17235_lociStitched	ARFRP1	0.11	0.37	-0.27	0.59	0.19	-6.28	0.71	loss	15
22	38138277	38150317	MACS_peak_18004	TRIOBP	0.20	0.45	-0.25	NA	NA	NA	0.71	NA	8
22	38176901	38182015	MACS_peak_18012	PDCL3	0.22	0.60	-0.38	0.64	0.31	-6.46	NA	NA	3
22	46464429	46478530	MACS_peak_18258	MIRLET7	0.53	0.99	-0.46	NA	NA	NA	NA	NA	3

Supplementary Table 9: Large hypomethylated regions in primary and metastatic colorectal cancer samples.

Sample type	Chr.	Start	End	Size (bp)	Meth. level	Gene symbol	Distance TSS	% HMR normal colon
Primary tumor	1	218336382	218349940	13558	0.09	TGFB2	168736	0.28
Primary tumor	13	73628949	73641647	12698	0.15	KLF5	0	0.30
Primary tumor	9	74373398	74385279	11881	0.10	TMEM2	0	0.28
Primary tumor	2	91663698	91675203	11505	0.10	IGKV1OR2-118	3693	0.46
Primary tumor	8	117884649	117895539	10890	0.13	RAD21	0	0.25
Primary tumor	2	90448882	90459317	10435	0.09	AC233263.3	53454	0.19
Primary tumor	21	34437234	34447500	10266	0.19	OLIG1	0	0.36
Primary tumor	5	127414083	127424041	9958	0.06	SLC12A2	0	0.36
Primary tumor	1	207921099	207930539	9440	0.13	CD46	0	0.20
Primary tumor	1	203289774	203299097	9323	0.16	U6	1547	0.40
Primary tumor	20	25227101	25236365	9264	0.14	PYGB	0	0.31
Primary tumor	2	173291608	173300419	8811	0.12	ITGA6	0	0.42
Primary tumor	22	37410887	37419122	8235	0.19	MPST	0	0.23
Primary tumor	11	46933497	46941707	8210	0.11	LRP4	0	0.39
Primary tumor	15	63795181	63803381	8200	0.04	USP3	0	0.47
Primary tumor	21	33244343	33252482	8139	0.09	HUNK	0	0.43
Primary tumor	18	55463636	55471500	7864	0.13	ATP8B1	0	0.32
Primary tumor	2	230577345	230585201	7856	0.11	DNER	0	0.36
Primary tumor	10	32338334	32346117	7783	0.12	KIF5B	0	0.45
Primary tumor	6	124122411	124130163	7752	0.18	NKAIN2	0	0.42
Primary tumor	12	33044638	33052291	7653	0.13	PKP2	0	0.30
Primary tumor	5	94615687	94623331	7644	0.11	MCTP1	0	0.39
Primary tumor	1	207222956	207230560	7604	0.13	PFKFB2	0	0.45
Primary tumor	6	128835158	128842574	7416	0.06	PTPRK	0	0.39
Primary tumor	7	17337977	17345308	7331	0.07	AHR	0	0.32
Primary tumor	14	55591597	55598891	7294	0.14	LGALS3	0	0.26
Metastasis	3	41231323	41265376	34053	0.14	CTNNB1	0	0.15
Metastasis	5	127414053	127440949	26896	0.10	SLC12A2	0	0.13
Metastasis	22	46459859	46480180	20321	0.14	MIRLET7	0	0.43
Metastasis	9	29192909	29213215	20306	0.18	LINGO2	41208	0.20
Metastasis	5	94614719	94634334	19615	0.17	MCTP1	0	0.15
Metastasis	21	40174290	40193387	19097	0.18	ETS2	0	0.46
Metastasis	17	63540803	63558867	18064	0.12	AXIN2	0	0.35
Metastasis	2	90442156	90460214	18058	0.08	AC233263.3	52557	0.27
Metastasis	3	87025252	87041710	16458	0.19	VGLL3	0	0.10

Metastasis	4	75229835	75245790	15955	0.20	EREG	0	0.20
Metastasis	2	91667887	91682638	14751	0.08	IGKV1OR2-118	0	0.30
Metastasis	1	218336537	218350719	14182	0.09	TGFB2	167958	0.26
Metastasis	14	94639297	94652604	13307	0.16	PPP4R4	0	0.31
Metastasis	9	74372950	74385279	12329	0.07	TMEM2	0	0.27
Metastasis	12	89739454	89751618	12164	0.05	DUSP6	0	0.43
Metastasis	13	73628491	73640649	12158	0.09	KLF5	0	0.31
Metastasis	17	56404994	56417144	12150	0.10	MIR4736	0	0.40
Metastasis	21	47062453	47074317	11864	0.14	PCBP3	0	0.18
Metastasis	1	207919635	207930507	10872	0.09	CD46	0	0.17
Metastasis	22	46433823	46444602	10779	0.18	MIRLET7	0	0.16
Metastasis	2	132767266	132777949	10683	0.17	RP1-156L9.1	15532	0.07
Metastasis	5	88172728	88183358	10630	0.18	CTC-454M9.1	0	0.49
Metastasis	11	46931142	46941707	10565	0.10	LRP4	0	0.30
Metastasis	11	25440840	25450991	10151	0.18	AC015820.1	107833	0.00
Metastasis	8	106324990	106335123	10133	0.12	ZFPM2	0	0.26
Metastasis	14	66972981	66982851	9870	0.08	GPHN	0	0.29
Metastasis	1	142928205	142937914	9709	0.20	RP11-423O2.7	18802	0.00
Metastasis	21	33243902	33253577	9675	0.11	HUNK	0	0.36
Metastasis	21	10105053	10114480	9427	0.20	CR381653.1	66688	0.16
Metastasis	3	79060095	79069497	9402	0.09	ROBO1	0	0.43
Metastasis	1	203289774	203299097	9323	0.13	U6	1547	0.40
Metastasis	1	11530768	11540086	9318	0.12	PTCHD2	0	0.18
Metastasis	2	41704857	41713858	9001	0.12	AC010739.1	109668	0.00
Metastasis	1	37500826	37509794	8968	0.19	GRIK3	1096	0.46
Metastasis	14	88781784	88790680	8896	0.16	KCNK10	0	0.27
Metastasis	1	151960764	151969653	8889	0.10	AL591893.1	0	0.50
Metastasis	6	69935822	69944658	8836	0.18	BAI3	0	0.13
Metastasis	7	102787637	102796362	8725	0.15	RP11-401L13.5	0	0.20
Metastasis	2	106007479	106016181	8702	0.18	FHL2	0	0.15
Metastasis	7	26191459	26200010	8551	0.08	NFE2L3	0	0.37
Metastasis	2	230577164	230585636	8472	0.11	DNER	0	0.34
Metastasis	13	67803752	67812201	8449	0.13	PCDH9	0	0.28
Metastasis	3	89155675	89164093	8418	0.13	EPHA3	0	0.39
Metastasis	13	78265037	78273411	8374	0.04	MIR3665	0	0.45
Metastasis	18	53249062	53257434	8372	0.10	TCF4	0	0.38
Metastasis	9	177326	185696	8370	0.16	CBWD1	0	0.24
Metastasis	8	92610199	92618527	8328	0.19	7SK	3006	0.29
Metastasis	15	79596231	79604549	8318	0.15	TMED3	0	0.19

Metastasis	12	33044638	33052875	8237	0.10	PKP2	0	0.28
Metastasis	12	89912441	89920652	8211	0.08	GALNT4	0	0.31
Metastasis	20	5583918	5592121	8203	0.08	GPCPD1	0	0.42
Metastasis	7	30322505	30330689	8184	0.06	MIR550A1	0	0.32
Metastasis	1	235092612	235100792	8180	0.15	RP11-443B7.1	0	0.31
Metastasis	14	65167106	65175127	8021	0.12	PLEKHG3	0	0.39
Metastasis	10	32338291	32346311	8020	0.07	Y_RNA	0	0.44
Metastasis	15	63795181	63803166	7985	0.04	USP3	0	0.49
Metastasis	12	12937531	12945460	7929	0.13	APOLD1	0	0.15
Metastasis	11	94276085	94283992	7907	0.06	FUT4	0	0.23
Metastasis	8	117884817	117892656	7839	0.07	MIR3610	0	0.32
Metastasis	3	12328878	12336686	7808	0.09	PPARG	0	0.44
Metastasis	5	129239340	129247107	7767	0.10	CHSY3	0	0.48
Metastasis	1	207222956	207230681	7725	0.10	PFKFB2	0	0.45
Metastasis	7	77320936	77328644	7708	0.12	RSBN1L	0	0.33
Metastasis	10	11055794	11063495	7701	0.07	CELF2	0	0.32
Metastasis	20	31170742	31178437	7695	0.09	RP11-410N8.4	0	0.41
Metastasis	1	46595249	46602845	7596	0.08	RP4-533D7.5	0	0.42
Metastasis	2	69607934	69615506	7572	0.11	GFPT1	0	0.29
Metastasis	12	71832196	71839719	7523	0.05	LGR5	0	0.41
Metastasis	2	91995135	92002654	7519	0.05	AC127391.1	3143	0.15
